# Glycan Array Evaluation of Synthetic Epitopes between the Capsular Polysaccharides from *Streptococcus pneumoniae* 19F and 19A

**DOI:** 10.1021/acschembio.1c00347

**Published:** 2021-09-01

**Authors:** Laura Morelli, Luigi Lay, Darielys Santana-Mederos, Yury Valdes-Balbin, Vicente Verez Bencomo, Angela van Diepen, Cornelis H. Hokke, Fabrizio Chiodo, Federica Compostella

**Affiliations:** † Department of Medical Biotechnology and Translational Medicine, University of Milan, Via Saldini 50, 20133 Milano, Italy; ‡ Department of Chemistry, University of Milan, Via Golgi 19, 20133 Milano, Italy; § Finlay Vaccine Institute, 200 and 21 Street, 11600 Havana, Cuba; ∥ Department of Parasitology, Leiden University Medical Center, Albinusdreef 2, 2333 ZA Leiden, The Netherlands; ⊥ Italian National Research Council (CNR), Institute of Biomolecular Chemistry (ICB), Via Campi Flegrei 34, 80078 Pozzuoli, Italy

## Abstract

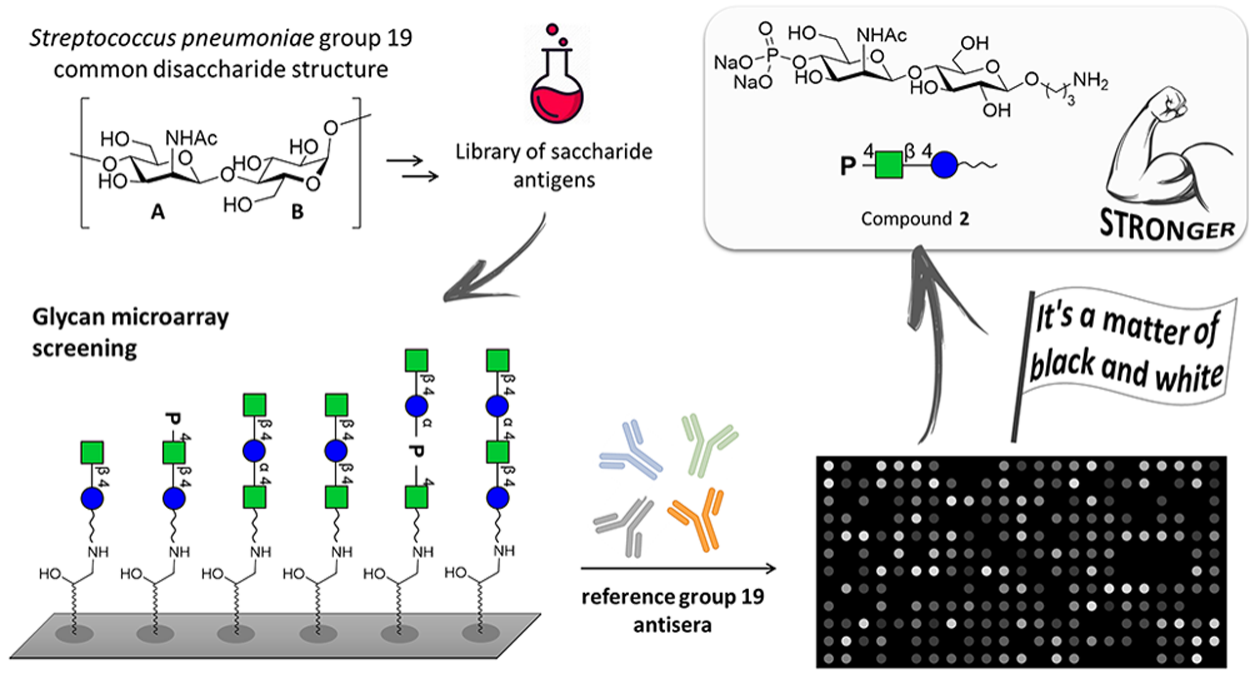

Vaccination represents the most effective way to prevent invasive pneumococcal diseases. The glycoconjugate vaccines licensed so far are obtained from capsular polysaccharides (CPSs) of the most virulent serotypes. Protection is largely limited to the specific vaccine serotypes, and the continuous need for broader coverage to control the outbreak of emerging serotypes is pushing the development of new vaccine candidates. Indeed, the development of efficacious vaccine formulation is complicated by the high number of bacterial serotypes with different CPSs. In this context, to simplify vaccine composition, we propose the design of new saccharide fragments containing chemical structures shared by different serotypes as cross-reactive and potentially cross-protective common antigens. In particular, we focused on *Streptococcus pneumoniae* (Sp) 19A and 19F. The CPS repeating units of Sp 19F and 19A are very similar and share a common structure, the disaccharide ManNAc-β-(1→4)-Glc (A-B). Herein, we describe the synthesis of a small library of compounds containing different combinations of the common 19F/19A disaccharide. The six new compounds were tested with a glycan array to evaluate their recognition by antibodies in reference group 19 antisera and factor reference antisera (reacting against 19F or 19A). The disaccharide A-B, phosphorylated at the upstream end, emerged as a hit from the glycan array screening because it is strongly recognized by the group 19 antisera and by the 19F and 19A factor antisera, with similar intensity compared with the CPSs used as controls. Our data give a strong indication that the phosphorylated disaccharide A-B can be considered a common epitope among different Sp 19 serotypes.

## Introduction

The Gram-positive bacterium *Streptococcus pneumoniae* (Sp) is a major cause of otitis media, bacteremia, and meningitis. In addition, Sp is the leading cause of community-acquired pneumonia despite the worldwide administration of pneumococcal conjugate vaccines.^1,2^ A recent analysis by UNICEF estimates that pneumonia kills one child every 39 s.^3^ Sp accounts for approximately 100 serotypes, defined by the different serotype-specific capsular polysaccharide structures (CPSs). The CPSs are the most important virulence factor of the bacterium and are an optimal target for vaccine design and development.^4^ The pneumococci are common inhabitants of the upper and lower respiratory tract microbial community. Most serotypes are causes of morbidity, but only a few are responsible for the majority of invasive pneumococcal diseases (IPDs).^5^ The incidence is more severe in the youngest and oldest portion of the population and independent of the level of economic development of the patients’ country. Nasopharyngeal colonization, the first usually asymptomatic step in the development of an invasive disease, is also considered a crucial determinant at the basis of horizontal dissemination of the pathogen within the community.^6^ Recently, the composition of the lung microbiota has been linked to lung carcinogenesis and to the establishment of lung metastasis, adding new clinical perspectives for the use and impact of *S. pneumoniae* vaccines.^7^ Vaccination represents the most effective way to prevent individual invasive disease, hinder primary intranasal colonization, reduce nasopharyngeal carriage, and prevent pneumococcal infections and carriage throughout the community. Extensive vaccination programs with pneumococcal polysaccharide (PPVs) and conjugate (PCVs) vaccines have effectively reduced the disease burden, although important limitations remain. The most relevant limitation is caused by the large structural diversity of capsular polysaccharides, which constitutes a major challenge for eliminating pneumococcal disease. Vaccines include only the CPSs from the serotypes causing the majority of the IPDs in the world or in a specific geographic area. Protection is serotype-specific, and in most of the cases, commercial vaccines are unable to protect against serotypes not included in the vaccine (nonvaccine serotypes), because the antigenicity of the capsule is type-specific. Furthermore, Sp host colonization is known to evolve under the pressure of the host environment^8^ and can generate novel antigenic diversity by recombination, with the generation of diverse capsular polysaccharide species over time.^9^ One way to overcome the limitations of licensed vaccines is to increase the valency, i.e., the number of vaccine serotypes in the PCV formulations. Fifteen- and twenty-valent vaccine candidates (20vPnC-Pfizer and V114-Merck) are under examination for marketing or license authorizations.^10,11^ They demonstrated safety and immunogenicity profiles comparable to those of the licensed 13-valent vaccine (PCV13-Pfizer).^12−14^ In addition, two 24-valent formulations, one of which exploits a new site-specific conjugation technology, are under preclinical evaluation.^15,16^ However, due to the global variation in serotype prevalence, the search for new vaccine candidates and approaches that elicit broader protection is important considering the efforts involved in vaccine development.^17^ Ideal candidates should be protective against a broader range of pneumococcal serotypes, with the possibility of the addition in the vaccine formulation of emerging new clinical isolates. Several alternatives have been studied to develop novel vaccine candidates with a broader coverage, for example, by using inactivated whole cell vaccine strains or pneumococcal proteins, such as pneumococcal surface protein A (PspA), pneumolysin (Ply), pneumococcal surface protein C (PspC), and pneumococcal surface adhesin A (PsaA).^18,19^ To date, a highly cross protective protein antigen has not been validated in human trials, and the sugar-based approach is at the moment the unique available option for *S. pneumoniae*.

Synthetic saccharide fragments of Sp 6B CPS, conjugated to the protein keyhole limpet hemocyanin (KLH), proved to be able to induce the production of antibodies that are protective not only toward the 6B serotype but also toward 6A.^20^ The cross-reactivity is thought to be due to a shared (sub)structure, common to 6A and 6B serotypes in the saccharide fragments, opening the idea of a common epitope structure.^21^

**Figure 1:**
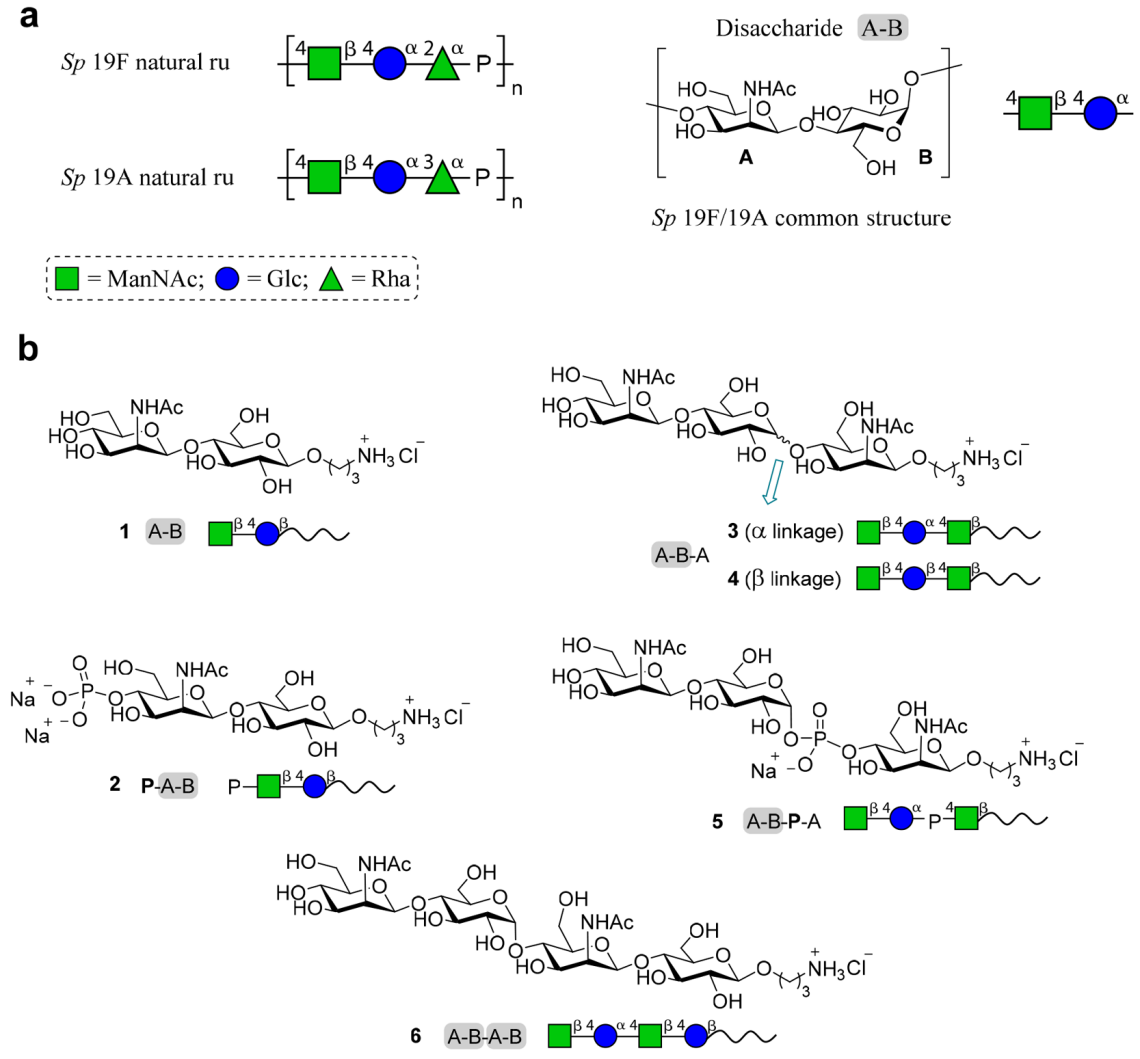
(a) Natural repeating units of *S. pneumoniae* 19F and 19A CPSs highlighting their common structures, the disaccharide A-B. (b) Synthesized oligomers related to different combinations of the common disaccharide A-B [ManNAc-β-(1→4)-Glc].

In the context of cross-reactive and potentially cross-protective saccharide fragments common to different bacterial CPSs, we addressed the rational design of new saccharide fragments containing chemical structures shared by different serotypes of *S. pneumoniae*. In particular, we focused on Sp 19F and 19A, which are the most common IPD-causing serotypes in young people.^22,23^ Sp 19F and 19A CPSs consist of very similar repeating units, differing only in the position of one glycosidic linkage. However, antibody cross-protection between the two serotypes has not been demonstrated, as established by the increase in the number of infections caused by serotype 19A after vaccination with pneumococcal vaccines containing only serotype 19F.^24^ The weak antibody cross-protection is probably due to conformational differences between the two polysaccharide chains.^25^ The polysaccharide repeating unit (RU) structures consist of a linear trisaccharide containing an *N*-acetyl-d-mannosamine unit (A) linked through a β-(1→4) bond to a d-glucose residue (B) that in turn is linked to an l-rhamnose unit through an α-(1→2) bond in Sp 19F or an α-(1→3) bond in Sp 19A. The RU are linked to each other via α-(1→4) phosphodiester bridges (Figure 1a).^26^ They share the common structure of the ManNAc-β-(1→4)-Glc disaccharide. Herein, we describe the synthesis of six different saccharides, containing different overlapping combinations of the common disaccharide subunit. We evaluated their recognition by antibodies in reference sera from immunized rabbits using a microarray format. Results showed that phosphorylated disaccharide **2** (Figure 1b) is strongly recognized by antibodies in both reference sera of rabbits immunized against either Sp 19F or Sp 19A. In addition, sera against the Sp group 19 (including serotypes 19F and 19A as well as 19B and 19C) also showed a strong recognition regarding disaccharide **2**. Our study could improve our understanding of cross recognition, setting the stage for the development of broadly protective interventions.

## Results and Discussion

A small library of rationally designed non-natural saccharide antigens has been synthesized (Figure 1b). The synthesized structures contain different combinations of the common disaccharide A-B [ManNAc-β-(1→4)-Glc] and do not represent frameshifts of the Sp 19A or 19F capsular polysaccharides. The synthesized compounds were rationally designed taking into account the possible existence of non-natural conformational epitopes (discontinuous residues) as Sp19A/Sp19F common epitopes.^27^ Each compound was functionalized at the reducing end with an aminopropyl linker to allow conjugation to carrier proteins^28,29^ or preparation of multivalent systems.^30−33^

Figure 1b shows that the new compounds differ for the length of the saccharide motif, from the shorter basic disaccharides **1** and **2** to tetrasaccharide **6**, and/or the presence of the phosphate group. Negatively charged phosphates are part of the repeating units found in the polysaccharides, and they can play an important role in carbohydrate activity and antibody recognition.^34^ On this basis, we planned to evaluate the antibody binding characteristics when the phosphate is (i) absent (compounds **1**, **3**, **4**, and **6**), (ii) present at the upstream end, as in compound **2**, or (iii) a bridge between two saccharides (trisaccharide **5**). The presence of *N*-acetyl-d-mannosamine (A) is a characteristic of Sp 19A and 19F polysaccharide chains, potentially endowed with an immunodominant role during biological recognition. Thus, to elongate the parent disaccharide A-B to the trisaccharide structures, we have planned to conjugate an additional unit of *N*-acetyl-d-mannosamine at the reducing end of the disaccharide in the presence (compound **5**) or absence (compound **3** or **4**) of the phosphate group. In addition, compounds **3** and **4** differ in the stereochemistry of the glycosidic linkage connecting the disaccharide A-B to the additional mannosamine unit, to underline the role of the stereochemistry in activity.

**Scheme 1:**
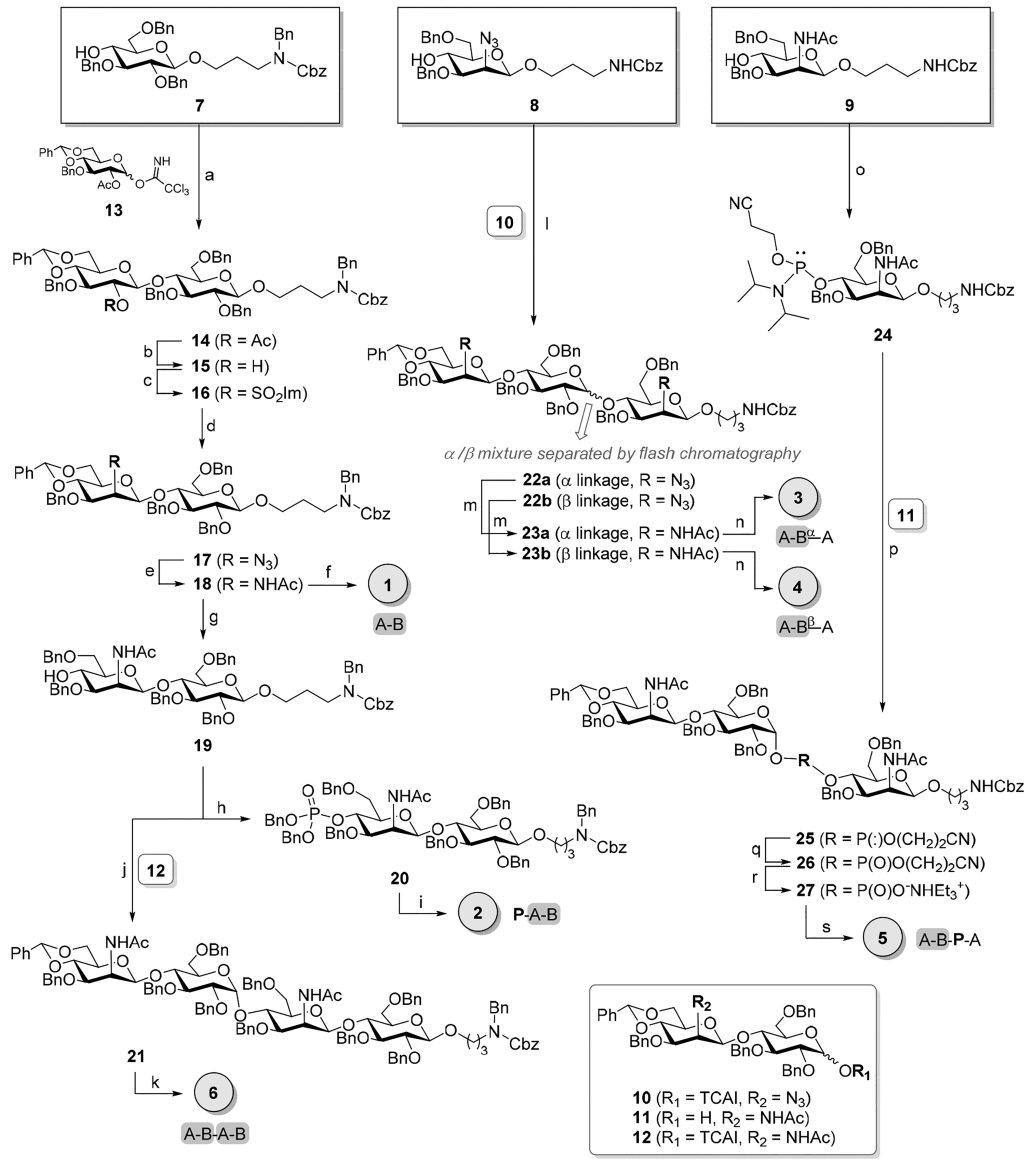
Synthetic Approach to Target Compounds **1–6** Reagents and conditions: (a) TESOTf, DCM, 4 Å molecular sieves (MS), −20 °C, 74%; (b) MeONa, DCM/MeOH, 93%; (c) SO_2_Im_2_, NaH_60%_, DMF, −40 °C, 75%; (d) NaN_3_, DMF dry, 80 °C, 90%; (e) Zn, CuSO_4_·5H_2_O, THF/Ac_2_O/AcOH, 66%; (f) Pd(OH)_2_/C, H_2_, EtOAc/MeOH/HCl, 99%; (g) Et_3_SiH, BF_3_·Et_2_O, DCM, 0 °C, 4 Å MS, 80%; (h) (BnO)_2_PN(iPr)_2_, tetrazole, DCM, then mCPBA, −20 to 0 °C, 80%; (i) Pd/C, H_2_, MeOH/H_2_O, 80%; (j) TMSOTf, DCM, 4 Å MS, 0 °C to room temperature (rt), 55%; (k) Pd(OH)_2_/C, H_2_, EtOAc/MeOH/HCl, 95%; (l) TESOTf, DCM, 4 Å MS, −20 °C, 85%; (m) Zn, CuSO_4_·5H_2_O, THF/Ac_2_O/AcOH, 30–47%; (n) Pd(OH)_2_/C, H_2_, EtOAc/MeOH/HCl, quant; (o) Cl(iPr_2_N)P(OCH_2_CH_3_CN), DIPEA, DCM, 90%; (p) DCI, DCM, 95%; (q) *t*BuOOH, ACN, 0 °C to rt, 73% (55% α); (r) TEA, DCM, 4 days, 80%; (s) Pd/C, H_2_, MeOH/H_2_O, 90%. DIPEA, *N*,*N*-diisopropylethylamine; DCI, 4,5-dicyanoimidazole; ACN, acetonitrile.

The set of saccharides has been obtained through a convergent approach (Scheme 1) based on the initial preparation of partially protected saccharides. In particular, building blocks **7–9** are the glucosyl and mannosyl acceptors designed as the aminopropyl-functionalized primers for the synthesis of all of the compounds. In addition, three differently protected A-B disaccharides, compounds **10–12**, were designed as the upstream moieties for oligomer elongation and synthesized as reported in section 1 of the Supporting Information from known protected glucosides.

We started with the synthesis of compounds **1**, **2**, and tetrasaccharide **6** (Scheme 1). Building block **7** was glycosylated on a gram scale with known glucosyl trichloroacetimidate donor **13**(35) under the catalysis of triethylsilyl triflate, to give disaccharide **14** in 74% yield. Then, the glucosyl moiety at the upstream end was subjected to *gluco* to *manno* epimerization through three consecutive manipulations at C-2′: first deacetylation under Zémplen conditions, then activation with sulfonyldiimidazole, followed by nucleophilic substitution with sodium azide to give disaccharide **17** in 60% overall yield.^29^ Subsequently, azido reduction with the zinc–copper couple, in the presence of acetic anhydride, allowed us to obtain acetamide **18** in 66% yield. On one hand, disaccharide **18** was subjected to hydrogenolysis to give quantitatively compound **1**, the first derivative of the library. On the other hand, compound **18** was selectively deprotected at C-4 of the mannosyl unit through a regioselective reductive opening of the benzylidene acetal to give disaccharide **19** in good yield (80%). Disaccharide **19** was used as the precursor of phosphorylated disaccharide **2**, the second compound of the library, and as the acceptor in the glycosylation toward tetrasaccharide **6**. We initially introduced the phosphate group at the upstream end of **19**, through phosphitylation with dibenzyl *N,N*-diisopropylphosphoramidite in the presence of 1*H*-tetrazole, followed by *m*-CPBA oxidation, to give dibenzylphosphate **20** in 80% yield.^36^ Hydrogenolysis of **20** gave phosphorylated disaccharide **2**. Then, the synthesis of tetrasaccharide **6** was accomplished by a TMSOTf-promoted glycosylation between disaccharide acceptor **19** and trichloroacetamidate donor **12**. Fully protected tetrasaccharide **21** was obtained in 55% yield in exclusively α-configuration of the newly formed glycosidic bond (*J*_1″,2″_ = 3.7 Hz). Compound **21** was then deprotected to give the desired tetrasaccharide **6** in 95% yield.

We then turned our attention to the synthesis of the trisaccharide targets (Scheme 1). Mannosyl acceptor **8** was glycosylated in 85% yield with disaccharide donor **10** under the promotion of triethylsilyl triflate. The glycosylation provided a 2:1 α/β mixture of trisaccharides, **22a** and **22b**, which were easily separated by flash column chromatography. Then, the two anomers were reacted separately in the next steps to yield trisaccharides **3** and **4**, which differ in the anomeric configuration of the newly formed glycosidic bond. Initial conversion of the two azido groups into *N*-acetamides, followed by hydrogenolysis, gave the desired compounds **3** and **4**.

The last compound of the library is trisaccharide **5**, which displays a phosphate moiety as the connection between one disaccharide A-B unit and a ManNAc (A) residue (Scheme 1). In this case, mannosyl acceptor **9** was used instead of **8** as the primer for the preparation of the trisaccharide fragment. This corrective strategy was introduced to avoid the simultaneous reduction of multiple azide groups, which could cause a significant decrease in the chemical yield at the late stages of the synthesis. The phosphoramidite methodology was chosen for the construction of the phosphate bridge.^37−40^ At first, we decided to phosphitylate the anomeric hydroxyl group of disaccharide **11**, but disappointingly, any attempt to couple the resulting anomeric phosphoramidite to buiding block **9** was unsuccessful. We then used the reverse approach and phosphitylated the 4-OH of mannoside **9** with 2-cyanoethyl *N*,*N*-diisopropyl-chlorophosphoramidite in the presence of diisopropylethylamine (DIPEA). Phosphoramidite **24** was obtained in 90% yield as a 1:1 mixture of *R* and *S* diastereomers [^31^P NMR (CDCl_3_) δ 150.77, 149.99]. 4,5-Dicyanoimidazole (DCI)-mediated coupling^41^ of phosphoramidite **24** to a 5:1 α/β anomeric mixture of disaccharide **11** gave successfully in very high yield glycosyl phosphite triester **25** as a complex and inseparable mixture of diasteromers (α/β anomers, *R*/*S* diastereomers at P). The purified intermediate glycosyl phosphite triester **25** was oxidized with *tert*-butyl hydroxyperoxide to the corresponding phosphate in a separable α/β mixture (73% yield, 55% α-anomer). The α-anomer of **26** was treated with triethylamine to provide compound **27** in 80% yield. The final fully deprotected compound **5** was obtained in 90% yield after hydrogenolysis.

### Binding Studies with *S. pneumoniae* Antisera

**Figure 2:**
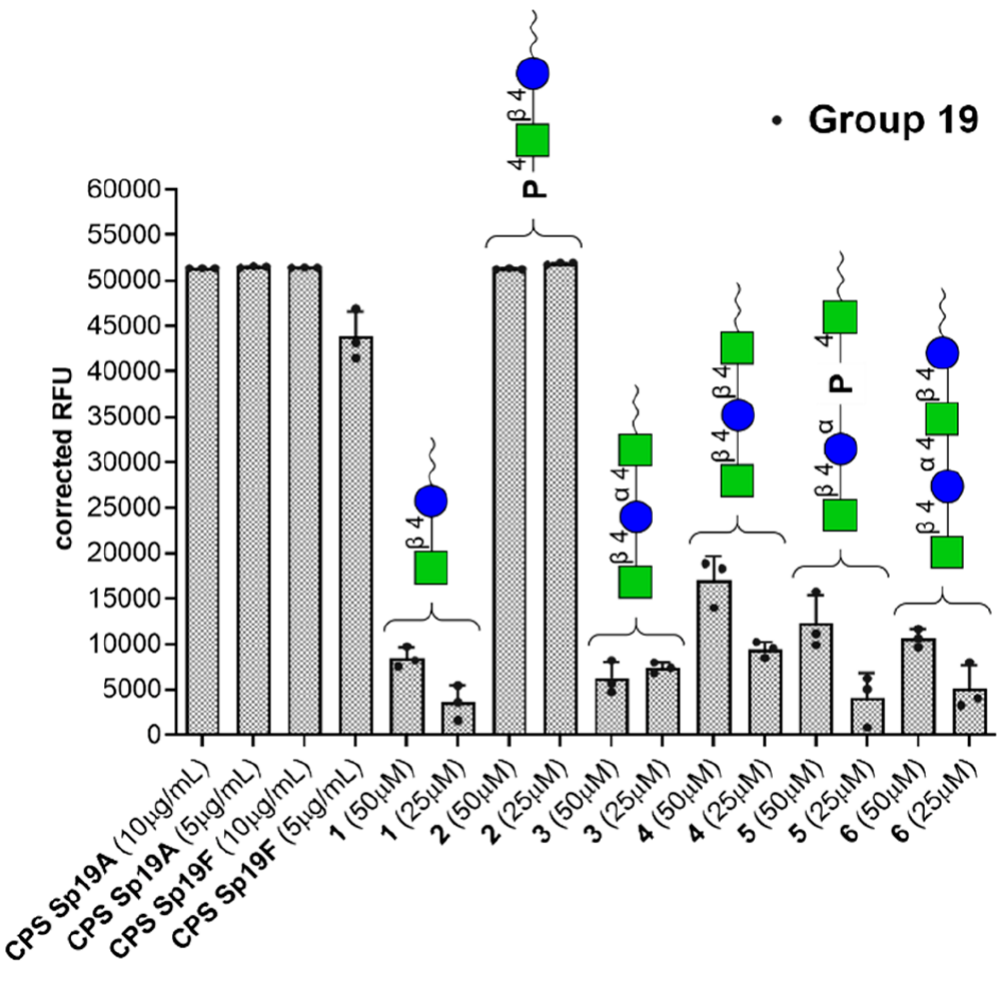
IgG binding of reference group 19 antisera recognizing the common epitope to all CPSs belonging to Sp group 19. The vertical axis represents the averaged serum IgG binding as relative fluorescence units (corrected over background). The horizontal axis shows the different synthetic structures printed on the glycan microarray. CPSs from Sp 19A and Sp 19F were used as positive controls as both contain the group 19 common epitope. Each bar corresponds to the median value from three replicates (represented as individual values with the circles) of the IgG binding to the printed Sp 19 common epitopes. Raw data of glycan array analyses are reported in Figure SI-3.

The newly synthesized fragments were printed on epoxysilane-coated glass slides, and their interaction with immunoglobulins in *S. pneumoniae* antisera was studied and evaluated by a microarray as previously described.^42−44^ Carbohydrate microarrays have been explored in the past several years as a high-throughput screening method to study the interactions of carbohydrates with different carbohydrate-binding receptors^45,46^ and antibodies in different biological fluids (including plasma and sera). For example, carbohydrate microarrays have been used to evaluate lectin binding and anticarbohydrate antibodies in the context of cancer and vaccine development.^47−51^ In this study, a glycan microarray was constructed by exploiting covalent immobilization of glycan fragments and controls according to previously established procedures.^42,43^ The six newly synthesized fragments were printed on the slide, together with native Sp 19F and 19A CPSs as controls. Natural and non-natural saccharide fragments were incubated first with pneumococcal reference group 19 antisera from rabbits immunized with the whole bacteria (Figure 2). The presence of specific IgG binding was detected by means of secondary fluorescent anti-rabbit IgG antibodies.

**Figure 3:**
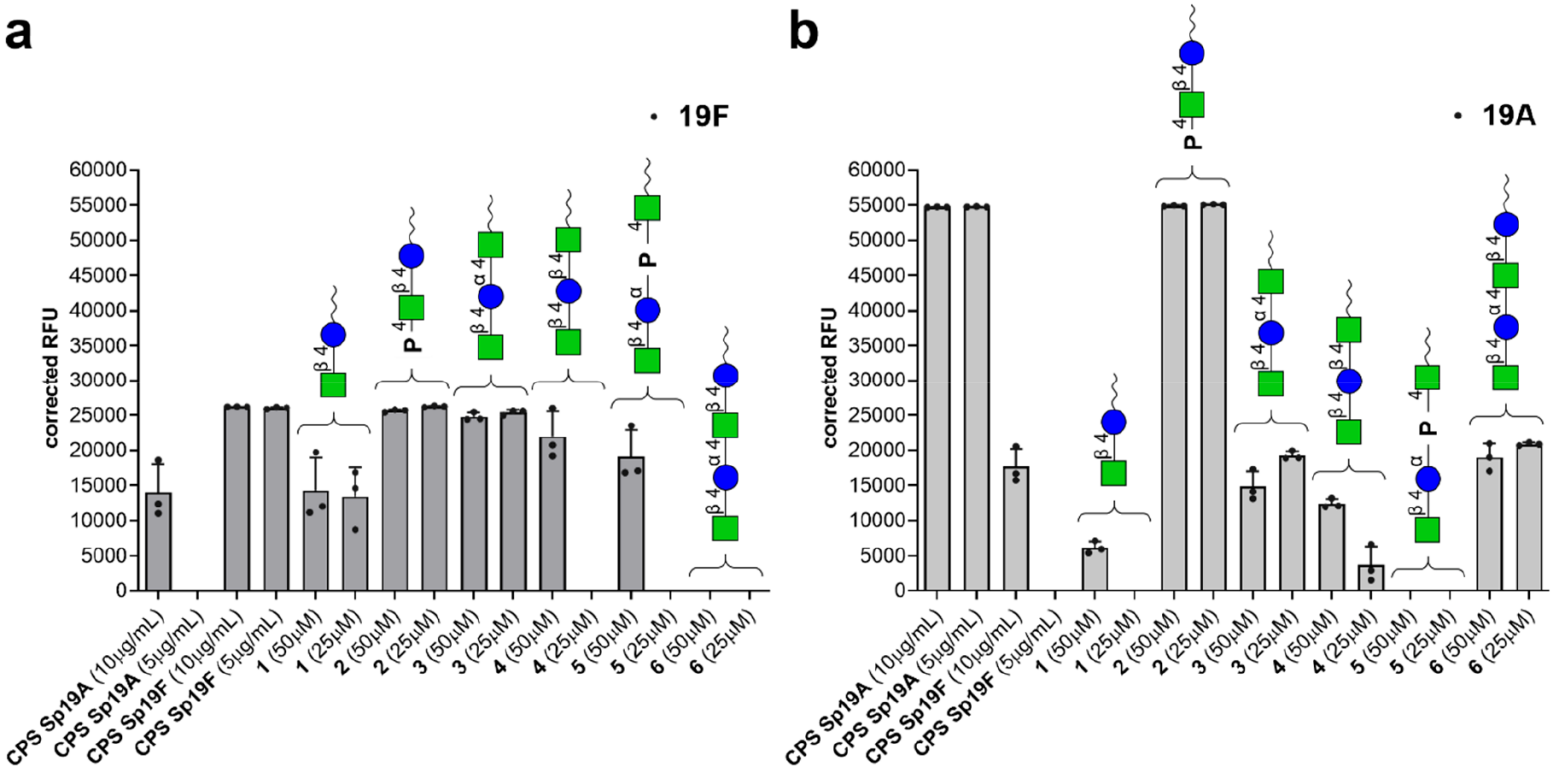
IgG in infected reference sera can recognize different synthetic Sp 19 common epitopes printed on the glycan microarray. The vertical axis represents the averaged serum IgG binding as relative fluorescence units (corrected over background). The horizontal axis shows the different synthetic structures printed on the glycan microarray. CPSs from Sp 19F and Sp 19A were used as positive or negative controls, depending on the tested sera. Each bar corresponds to the median value from three replicates (represented as individual values with the circles) of the IgG binding to the printed Sp 19 common epitopes. (a) IgG binding of reference sera from rabbits immunized with Sp 19F. (b) IgG binding of reference sera from rabbits immunized with Sp 19A. Raw data of glycan array analyses are reported in Figure SI-3.

The glycan microarray experiment was used to qualitatively identify glycan epitopes. In particular, we were interested in identifying synthetic epitopes that can be targeted simultaneously by antibodies raised by different pneumococcal serotypes. On this basis, we set up a first microarray analysis of the response of synthetic compounds **1–6** to reference sera. The group 19 antisera recognize all of the CPSs belonging to group 19. The data (Figure 2) showed that molecule **2** is the sole compound to be strongly recognized by the reference group 19 antisera, with similar intensities compared with the corresponding native 19F and 19A CPSs used as positive control. While disaccharide compound **1** showed weak binding to these sera, the presence of the phosphate in compound **2** added a structural element that can resemble the structure of a more complete common epitope. Compound **2** was thus further investigated for its role as a cross-reactive epitope. We then incubated the natural and non-natural saccharide fragments with two different pneumococcal factor reference antisera (Figure 3). It should be noted that factor antisera are obtained by immunization of rabbits with whole Sp 19F or Sp 19A bacterium and further purified to remove or reduce the antibodies recognizing the common epitopes. Interestingly, Figure 3a shows that anti-19F serotype-specific antibodies contain reduced but detectable antigroup 19 antibodies as observed by the reaction with 19A CPS. The highly reactive antibodies recognize structures having ManNAc-β-(1→4)-Glc present in molecules **2** and **3**. In addition, the presence of the phosphate in compound **2** improves the binding of sera compared with that of molecule **1**. Figure 3b shows that anti-19A antisera have detectable anti-group 19 antibodies as ascertained by the reaction with 19F CPS. It is noteworthy that the serotype-specific antiserum recognizes very strongly molecule **2**. Molecule **3** also showed a moderate cross-reactivity between the two different sera, even if the intensity did not reach the maximum in the case of Sp 19A antisera. Besides residual activities in each experiment, none of the other synthetic compounds showed significant binding activity in both arrays, thus excluding their role as conserved epitopes. Our data provide strong support to the identification of compound **2** as one of the common epitopes between Sp 19F and Sp 19A.

The substructure represented by molecule **2** is a structural motif also found in the natural polysaccharides of the other two serotypes of Sp group 19, Sp19B and Sp19C (see Figure SI-2). The main structural difference between group 19 CPSs is that Sp19F and Sp19A are linear polymers, while Sp19B and Sp19C are mono- and dibranched polysaccharides, respectively, where the A-B disaccharide is part of the linear main chain. On this basis, we set out to perform an additional microarray analysis of the response of the same set of compounds toward sera obtained by immunizing rabbits with 19B and 19C serotypes (see Figure SI-1a). Again, relevant antibody binding to molecule **2** was observed, with anti-19B/19C sera, as well as a good binding to molecule **4** [ManNAc-β-(1→4)-Glc-β-(1→4)-ManNAc], which expresses conserved patterns between 19B and 19C (see Figure SI-2). The other synthesized fragments showed weaker or no reactivity. On the contrary, no significant binding to the printed structures was observed on the glycan array with specific anti-19C sera (see Figure SI-1b). This last result highlights the role of the structural differences between 19B and 19C especially on their inner epitope accessibility and branches. At the same time, it allows us to exclude nonspecific binding to compound **2** in the previous set of experiments.

These results further support molecule **2** as a hypothetical Sp19 common epitope for the design of a new generation of anti-Sp vaccines.

## Conclusions

In the design of a new generation of anti-Sp vaccines, the idea of identifying conserved glycan epitopes among different Sp serotypes could be a valid option. Our study shows that a phosphorylated simple disaccharide can be considered as one common carbohydrate epitope shared among different Sp 19 serotypes. Our glycan array-based evaluation of the synthesized bacterial saccharides showed their capacity to be recognized by antibodies elicited by different serotypes. This evidence provides a basis for additional studies aimed at the identification of other common saccharide epitopes. For example, the lead phosphorylated disaccharide **2** could be elongated at the nonreducing end through the phosphate bridge by a rhamnose residue, giving rise to a longer structure still common to Sp 19A and 19F serotypes. In support of our approach, interestingly, a recent report by Sanapala et al. demonstrated that a synthetic carbohydrate antigen constituted by the covalent combination of two minimal Sp natural repeating units induced protective antibodies against multiple serotypes *in vivo*.^52^ These results and approaches could help in the design and formulation of a new generation of Sp carbohydrate-based vaccines, exploring synthetic common epitopes between different serotypes.

## Methods

### Immobilization of the Synthesized Fragment on Glass Slides

Solutions of the synthesized fragments were prepared from a 0.1 mM stock solution in Milli-Q and diluted with NEXTERION Spot buffer with 10% DMSO to final concentrations of 50 and 25 μM. Then, 50 μL of each solution was placed in a 384-well plate (Scienion) that was stored at −20 °C. The synthesized fragments were printed onto NEXTERION Slide E, an epoxysilane-functionalized glass slide, by contact printing using an Omnigrid 100 microarray printer (Genomic Solutions) equipped with SMP3 pins (0.7 nL at each contact).^53^ Dot spacing was set as 290 and 245 μm (*X*, *Y*). The printed slides were incubated overnight at room temperature (rt) at sufficient humidity to prevent spots from drying. Slides were allowed for the covalent binding of the studied compounds (**1–6**) via reaction with primary amine on the spacer at the reducing end.

### Serum Binding Experiments

A silicone gasket was placed in the microarray slide, allowing the proper separation between the different arrays. The remaining unreacted epoxysilane groups were blocked and quenched with 2% BSA and 50 mM ethanolamine in PBS for 60 min at rt. The slides were then washed with PBS, and each microarray was incubated with the specific diluted rabbit antiserum in PBS, 0.01% Tween 20, and 1% BSA (antisera diluted 1:200) for 60 min at rt while being shaken. Factor antisera produced by immunizing rabbits with killed whole bacterial cells were purchased from SSI Diagnostica A/S: factor antiserum 19b (reacts with 19F), factor antiserum 19c (reacts with 19A), factor antiserum 7h (reacts with 19B and 19C), factor antiserum 19f (reacts with 19C), and the group 19 antiserum (reacts with 19A, 19B, 19C, and 19F). After being washed with PBS, 0.05% Tween 20, and PBS, the slides were incubated with fluorescently labeled anti-rabbit IgG and goat anti-rabbit IgG Fc (DyLight 550) (Abcam, ab96984) (diluted 1:1000 in PBS and 0.01% Tween 20) for 30 min at rt while being shaken. After a final rinse with PBS, 0.05% Tween 20, and Milli-Q, the slides were dried and kept in the dark until they were scanned.

### Scanning and Data Analysis

A G2565BA scanner (Agilent Technologies) was used to scan the slides for fluorescence using two lasers (532 and 633 nm). Data and images were analyzed using GenePix Pro version 7.0 (Molecular Devices). The fluorescent spots were aligned and resized using round features with no composite pixel intensity (CPI) threshold as previously described.^54^ Background-subtracted median intensities were averaged and processed, and median values of negative controls included on each array were subtracted.

## Supporting Information Available

Figures SI-1 and SI-2, raw data of glycan array analyses (Figure SI-3), description of the synthetic strategy for the preparation of compounds **7–9** and **10–12**, detailed experimental procedures and characterization of all compounds, and NMR spectra of the compounds (PDF)
