# Supplementary material for: Glycan Array Evaluation of Synthetic Epitopes between the Capsular Polysaccharides from Streptococcus pneumoniae 19F and 19A
Source: ACS Chem Biol. 2021 Sep 1;16(9):1671–9. doi: 10.1021/acschembio.1c00347 (PMC8453487; doi:10.1021/acschembio.1c00347)

Glycan array evaluation of synthetic epitopes between the capsular polysaccharides from *Streptococcus pneumoniae* 19F and 19A

Laura Morelli,[1] Luigi Lay,[2] Darielys Santana-Mederos,[3] Yury Valdes-Balbin,[3] Vicente Verez Bencomo,[3] Angela van Diepen,[4] Cornelis H. Hokke,[4] Fabrizio Chiodo,[* # 4,5] and Federica Compostella[* # 1]

[1] Department of Medical Biotechnology and Translational Medicine, University of Milan, Via Saldini 50, 20133 Milano, Italy

[2] Department of Chemistry, University of Milan, Via Golgi 19, 20133 Milano Italy

[3] Finlay Vaccine Institute, 200 and 21 Street, 11600 Havana, Cuba

[4] Department of Parasitology, Leiden University Medical Center, Albinusdreef 2, 2333 ZA Leiden, The Netherlands

[5] Italian National Research Council (CNR), Institute of Biomolecular Chemistry (ICB), Via Campi Flegrei 34, 80078 Pozzuoli, Italy

[#] F.Ch. and F.Co. are co-last and co-corresponding

Supporting Information

Table of Contents

1	Supporting data to microarray experiments	S3
2	Experimental Part: General Information	S5
3	Experimental Part: General Procedures	S6
3.1	Zemplén de-*O*-acetylation (Procedure A)	S6
3.2	Activation with sulfonyl-diimidazole (Procedure B)	S6
3.3	*Gluco*-*Manno* epimerization by nucleophilic substitution with NaN_3 (Procedure C)	S6
3.4	Conversion of Azide to Acetamide with Zinc-Cu couple (Procedure D)	S6
3.5	Conversion of Azide to Acetamide with 1,3-propanedithiol followed by acetylation (Procedure E)	S7
3.6	*O*-Allyl group deprotection (Procedure F)	S7
3.7	Hydrogenolysis of benzyl-type protecting groups (Procedure G)	S7
4	Synthesis and characterization of strategically protected building blocks	S8
4.1	Synthesis of glucosyl acceptor **7**	S8
4.2	Synthesis of mannosyl acceptors **8** and **9**	S10
4.3	Synthesis of Disaccharides **10**, **11** and **12**	S19
4.3.1	Synthesis of glucosyl acceptor **43**	S20
4.3.2	Synthesis of trichloroacetimidate **13**	S22
5	Synthesis and characterization of disaccharides	S32
6	Synthesis and characterization of tetrasaccharides	S38
7	Synthesis and characterization of trisaccharides	S40
8	References	S50
9	NMR spectra of synthetic compounds	S52

1 Supporting data to microarray experiments

The six newly synthesized saccharide fragments were printed on the slide together with native Sp 19A and 19F CPSs as negative controls. The compounds were incubated using reference sera obtained from rabbits infected with Sp 19B/19C or 19C.

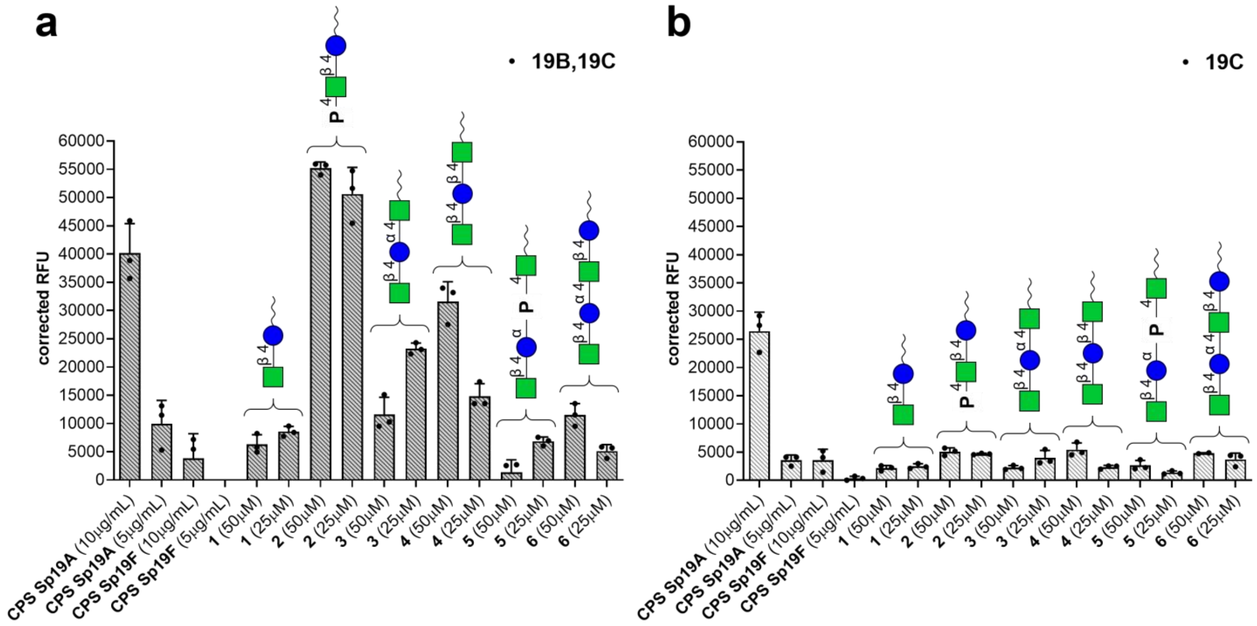

Figure SI-1. IgG binding of reference sera from animal rabbits immunized with: a) Sp 19 serotypes B and C (Factor 7h by SSI Diagnostica A/S Denmark); b) Sp 19 serotype C (Factor 19f by SSI Diagnostica A/S Denmark). The vertical axis represents the averaged serum IgG binding as relative fluorescence units (corrected over background). The horizontal axis shows the different synthetic structures printed on the glycan microarray. CPSs from Sp 19A and Sp 19F were used as controls. Each bar corresponds to the median value from three replicates (represented as individual values with the circles) of the IgG binding to the printed Sp 19 epitopes.

19F →4)-β-D-Man*p*NAc-(1→4)-α-D-Glc*p*-(1→2)-α-L-Rha*p*-(1→*P*→

19A →4)-β-D-Man*p*NAc-(1→4)-α-D-Glc*p*-(1→3)-α-L-Rha*p*-(1→*P*→

19B →4)-β-D-Man*p*NAc-(1→4)-α-D-Glc*p*-(1→4)-β-D-Man*p*NAc-(1→4)-[β-D-Rib*f*-(1→4)-α-L-Rha*p*-(1→3)]-α-L-Rha*p*-(1→*P*→

19C →4)-β-D-Man*p*NAc-(1→4)-α-D-Glc*p*-(1→4)-β-D-Man*p*NAc-(1→4)-[β-D-Rib*f*-(1→4)-α-L-Rha*p*-(1→3)]-[β-D-Glc*p*-(1→6)]-α-L-Rha*p*-(1→*P*→

Figure SI-2. Chemical structures of the CPSs of *Streptococcus pneumoniae* group 19 serotypes.

	Table format: Grouped	Group A 19F,19A,19B,19C			Group B 19F		
		A:Y1	A:Y2	A:Y3	B:Y1	B:Y2	B:Y3
1	CPS Sp19A (10µg/mL)	51358.710000	51347.710000	51359.710000	11036.000000	18635.000000	12378.000000
2	CPS Sp19A (5µg/mL)	51405.710000	51559.710000	51530.710000	0.000000	0.000000	0.000000
3	CPS Sp19F (10µg/mL)	51401.710000	51427.710000	51413.710000	26212.000000	26229.000000	26197.000000
4	CPS Sp19F (5µg/mL)	46878.710000	41447.710000	43167.710000	25881.000000	26144.000000	26070.000000
5	**1** (50µM)	9713.710000	8187.710000	7542.710000	11174.000000	19727.000000	12018.000000
6	**1** (25µM)	1616.710000	3580.710000	5416.710000	8734.000000	14624.000000	16858.000000
7	**2** (50µM)	51216.710000	51221.710000	51343.710000	25435.000000	25719.000000	25805.000000
8	**2** (25µM)	51739.710000	51939.710000	51925.710000	26164.000000	26248.000000	26349.000000
9	**3** (50µM)	4700.710000	5628.710000	8192.710000	24491.000000	24435.000000	25462.000000
10	**3** (25µM)	6849.710000	7375.710000	7956.710000	25643.000000	25624.000000	24986.000000
11	**4** (50µM)	18833.710000	18290.710000	13975.710000	26012.000000	20765.000000	19179.000000
12	**4** (25µM)	9501.710000	8476.710000	10197.710000	0.000000	0.000000	0.000000
13	**5** (50µM)	9884.710000	15758.710000	11116.710000	16837.000000	17087.000000	23512.000000
14	**5** (25µM)	6213.710000	5030.710000	818.000000	0.000000	0.000000	0.000000
15	**6** (50µM)	11615.710000	10614.710000	9613.710000	0.000000	0.000000	0.000000
16	**6** (25µM)	7991.710000	4017.710000	3266.710000	0.000000	0.000000	0.000000

	Table format: Grouped	Group D 19A		
		D:Y1	D:Y2	D:Y3
1	CPS Sp19A (10µg/mL)	54767.36000	54704.36000	54719.36000
2	CPS Sp19A (5µg/mL)	54763.36000	54751.36000	54822.36000
3	CPS Sp19F (10µg/mL)	20572.36000	15734.36000	16686.36000
4	CPS Sp19F (5µg/mL)	0.00000	0.00000	0.00000
5	**1** (50µM)	5922.36000	7093.36000	5387.36000
6	**1** (25µM)	0.00000	0.00000	0.00000
7	**2** (50µM)	54859.36000	55018.36000	54888.36000
8	**2** (25µM)	55088.36000	55085.36000	55148.36000
9	**3** (50µM)	17281.36000	13110.36000	14099.36000
10	**3** (25µM)	18893.36000	18940.36000	19953.36000
11	**4** (50µM)	11954.36000	12074.36000	13181.36000
12	**4** (25µM)	1511.36000	6601.36000	2903.36000
13	**5** (50µM)	0.00000	0.00000	0.00000
14	**5** (25µM)	0.00000	0.00000	0.00000
15	**6** (50µM)	17028.36000	20974.36000	19001.36000
16	**6** (25µM)	20874.36000	20686.36000	21118.36000

Figure SI-3. Individual raw data values from the relative fluorescent units (IgG in the tested sera) corrected over background signal (printed PBS).

2 Experimental Part: General Information

All chemicals were purchased from Sigma Aldrich (now Millipore Sigma, owned by Merck KGaA) and used without any further purification unless otherwise described. All the reactions were performed under Argon atmosphere and using dry solvents, unless otherwise indicated. The procedures are reported at the best optimized scales. The synthetic routes to compounds **35** and **47** were repeated twice in order to ensure the availability of the required amounts of substrates. Dichloromethane (DCM), triethylamine (TEA) and diisopropylamine (DIPEA) were freshly distilled from CaH_2 prior to use. Tetrahydrofuran (THF) was dried and simultaneously deperoxidated[1] by filtering over a pad of basic Al_2O_3, and stored over activated 4 Å molecular sieves (MS). Dimethylformamide (DMF) and methanol were dried over activated molecular sieves. NaH 60% dispersion in mineral oil was washed with hexane three times before use (only in small scale synthesis for safety reasons).

Thin Layer Chromatography (TLC) was performed by using Silica gel on Merck TLC-PET foils precoated with a fluorescent indicator. All reactions were followed by TLC-analysis: compounds were visualized, when appropriate, by UV light (254 nm) or detected by dipping into a *cerium molybdate*[2] or *ethanolic sulphuric acid*[3] or *ninhydrin*[4] staining solution followed by heating.

Organic solvents were removed under reduced pressure by rotary evaporation (water bath temperature = 45°C). Compounds were purified by flash column chromatography, following Still procedure,[5] using silica gel as stationary phase. Manual procedure involved the use of a high-purity grade silica gel by Merck (SiO_2, high-purity grade (9385), pore size 60 Å, 230-400 mesh particle size) and, generally, an isocratic elution. Automated flash chromatography was widely employed in medium/large scale synthesis by the use of Biotage® Isolera™ Prime purification System (SNAP cartridges, gradient elution).

The purity of all synthetized compound was verified by nuclear magnetic resonance (NMR) analysis. NMR characterization was carried out using a 500 MHz Bruker FT-NMR AVANCE DRX500 spectrometer (pulsed field gradient, reverse broadband probe) at a sample temperature of 298K. ^{1}H-NMR spectra were acquired at 500.13 MHz, ^{31}P at 202.46 MHz and ^{13}C at 125.77 MHz. NMR chemical shifts (δ) are reported in ppm. Bidimensional experiments (^{1}H-^{1}H COSY, ^{1}H-^{1}H TOCSY, ^{1}H-^{13}C HSQC and ^{1}H-^{31}P HMBC) were collected to better assign NMR signals. ^{31}P-NMR spectra are referenced to external standards (triphenylphosphine in $CDCl_3$, and H_3PO_4 in D_2O).

High Resolution Mass Spectrometry (HRMS) was carried out on a high definition hybrid quadrupole/time-of-flight (QTof) mass spectrometer (Synapt G2Si system by Waters) equipped with electron-spry ionization (ESI) probe. A Thermo Quest Finnigan LCQ^{TM}DECA ion trap mass spectrometer, equipped with a Finnigan ESI interface, was used to perform Low Resolution Mass Spectrometry (LRMS). Optical rotation was measured at room temperature with a Perkin-Elmer 241 polarimeter (589 nm, D line from Na lamp).

3 Experimental Part: General Procedures

3.1 Zemplén de-*O*-acetylation (Procedure A)[6,7]

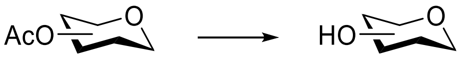

A 0.4 M (or 0.1 M) solution of sodium methoxide in methanol was added (0.25 eq. for each acetyl group, until basic pH) to a stirred solution of the sugar (1 mmol) in dichloromethane (10 mL per disaccharide mmol or 15 mL per monosaccharide mmol). Deacetylation was confirmed by TLC or NMR analysis, then the reaction was neutralized by an acidic ion-exchange resin (Dowex® 50WX8, H^+ form), filtered and concentrated.

3.2 Activation with sulfonyl-diimidazole (Procedure B)[8]

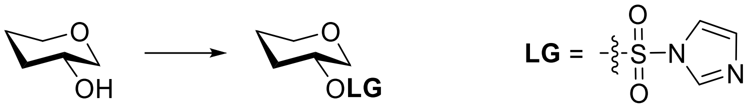

NaH (10 mmol) was added to a stirred solution of the sugar (1 mmol) in DMF (10 mL) at room temperature. After 1 h, the suspension was cooled to −40 °C and 1,1′-sulfonyl-diimidazole (5 mmol) in DMF (10 mL) was added. After 1 h the reaction was quenched with methanol and allowed to warm to room temperature. The reaction mixture was diluted with ethyl acetate and washed two times with brine, and the combined aqueous layers were extracted with ethyl acetate. Then, the combined organic layers were dried over sodium sulfate, filtered, and evaporated. The crude product was purified by flash chromatography.

3.3 Gluco-Manno epimerization by nucleophilic substitution with NaN_3 (Procedure C)[8]

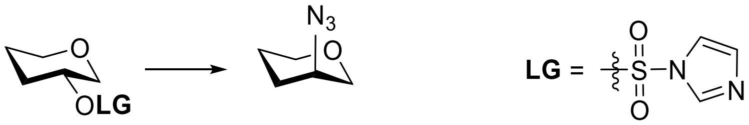

Sodium azide (10 mmol) was added to a stirred solution of 2-*O*-activated-glucoside (1 mmol) in DMF (20 mL), and the resulting mixture was heated at 80 °C. After 6 h, the reaction mixture was cooled at room temperature, diluted with water, and extracted with ethyl acetate (3 times). The combined organic extracts were washed with a 1:1 water/brine mixture, dried over sodium sulfate, filtered, and concentrated. Flash chromatography of the crude gave the resulting pure azido mannoside.

3.4 Conversion of Azide to Acetamide with Zinc-Cu couple (Procedure D)[8]

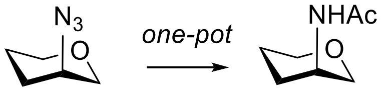

To a solution of the azido glycoside (1 mmol) in THF/Ac_2O/AcOH (3:2:1, 30 mL) freshly activated zinc (35 mmol, activated with aq. 2% $CuSO_4$ and washed 3 times with THF to remove the excess of

aqueous residues) was added by a spatula. As soon as TLC analysis revealed starting material consumption, the excess of zinc was removed by filtration through a Celite pad, washing with ethyl acetate. The organic was washed twice with satd $NaHCO_3$, and the combined aqueous phases were extracted with ethyl acetate. The combined organic layers were finally washed with brine, then dried over sodium sulfate, filtered, and concentrated. The residue was purified by flash chromatography.

3.5 Conversion of Azide to Acetamide with 1,3-propanedithiol followed by acetylation (Procedure E)[9–11]

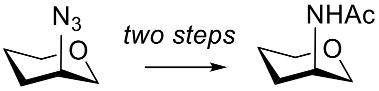

To a solution of the azido glycoside (1 mmol) in methanol (20 mL) 1,3-Propanedithiol (14 mmol) and freshly distilled DIPEA (14 mmol) were added in two equal portions within 24 h. After 72h, the reaction mixture (turned from a clear solution to a turbid white suspension) was concentrated under a stream of air with the aid of a Drechsel apparatus refilled with bleach to trap and neutralize the smelly thiol vapours. The crude was dissolved in pyridine (10 mL), then acetic anhydride (20 mmol) was added dropwise. After 24 h at room temperature, the reaction mixture was diluted with dichloromethane, filtered over a Celite pad, and then washed once with aqueous HCl (1N solution) and twice with brine. The organic layer was dried over sodium sulfate, filtered, and concentrated. The crude product was chromatographed on silica gel.

3.6 O-Allyl group deprotection (Procedure F)[12]

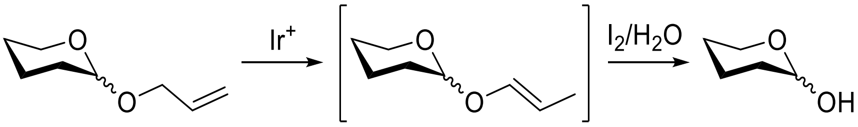

A red suspension of (1,5-cyclooctadiene)bis(methyldiphenylphosphine)iridium(I) hexafluorophosphate (0.02 mmol) in THF (6 mL) was degassed and stirred at room temperature under a hydrogen atmosphere for 10 min. The light-yellow clear solution of the activated catalyst was added to a solution of the allyl glycoside (1 mmol) in THF (14 mL, 0.05M final concentration). As soon as TLC analysis showed a complete conversion of the allyl ether into the 1-propenyl ether (usually after 3h), hydrolysis of the latter was performed by adding a solution of iodine (1.7 mmol) in THF/water (4:1, 30 mL). After 2-3 h, the reaction mixture was diluted with ethyl acetate and washed twice with freshly prepared aq 5% solution of $Na_2S_2O_5$. The aqueous phases were extracted with ethyl acetate, then the combined organic layers were washed with satd $NaHCO_3$ and brine, dried over sodium sulfate, filtered, and concentrated under reduced pressure. The residue was purified by flash column chromatography to afford the free anomeric hydroxyl group as an α/β mixture.

3.7 Hydrogenolysis of benzyl-type protecting groups (Procedure G)[13]

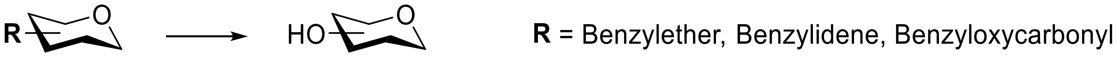

The sugar (0.05 mmol) dissolved in a 1:1:1 EtOAc/MeOH/0.1M HCl tri-solvent mixture (5 mL) was hydrogenolyzed in the presence of Pd/C or $Pd(OH)_2$/C (1:1 w/w, substrate/catalyst). As soon as TLC (or NMR) analysis indicated complete conversion, the reaction mixture was filtered over a Celite pad, washed with water (HPLC grade), and the filtrate was concentrated to 1 mL, and then lyophilized to give a white solid.

4 Synthesis and characterization of strategically protected building blocks

4.1 Synthesis of glucosyl acceptor 7

The glucosyl acceptor donor **7** was obtained in five steps, modifying the Wang procedure[14] according to our standard labs protocols. Glucose pentaacetate **28** was initially glycosylated with *N*-(carbobenzyloxy)-3-aminopropanol to give glucoside **29**, which was then deacetylated to compound **30**. Subsequent 4,6-*O*-benzylidene protection gave **31**, which was then benzylated to compound **32**. Lastly, the regioselective reductive opening of the benzylidene acetal gave acceptor **7** in satisfactory yield.

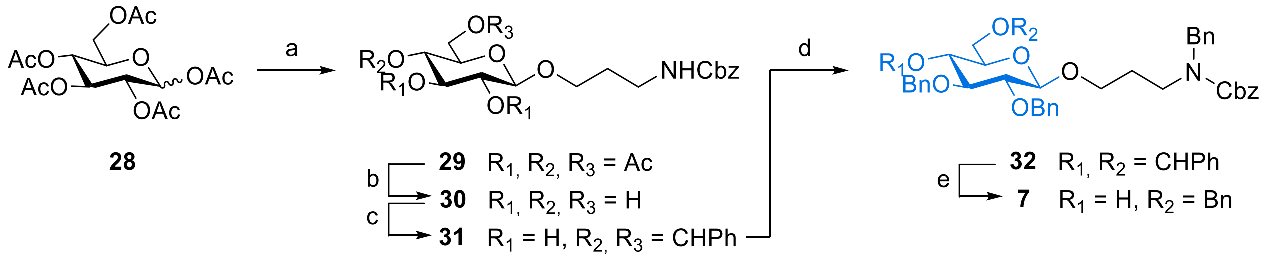

Reagents and conditions: a) *N*-Cbz-3-aminopropanol, $BF_3{\cdot}Et_2O$, DCM, **41%**; b) NaOMe/MeOH, DCM, **95%**; c) $PhCH(OMe)_2$, PTSA, CH_3CN, **75%**; d) BnBr, NaH, DMF, **83%**; e) Et_3SiH, $BF_3{\cdot}Et_2O$, DCM, 4Å MS, **63%**.
PTSA = *p*-Toluenesulfonic acid

N-(Carbobenzyloxy)-3-aminopropyl 2,3,4,6-tetra-*O*-Acetyl-β-D-glucopyranoside (29)

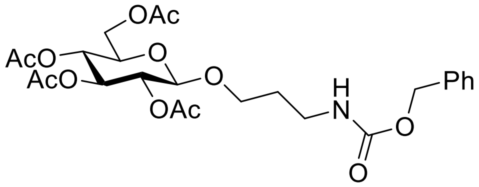

Chemical Formula: $C_{25}H_{33}NO_{12}$
Exact Mass: 539,20
Molecular Weight: 539,53

To a stirred solution of glucose pentaacetate **28** (3 g, 7.71 mmol) and *N*-CBz-aminopropanol (2.4 g, 11.57 mmol) in dichloromethane (26 mL, 0.8 M), a 1:1 solution of boron trifluoride diethyl etherate (9.5 mL, 77.1 mmol) in dichloromethane was added dropwise *via* a dropping funnel in two equal portions within 5 h of each other. After 18 h, the reaction was diluted with dichloromethane (200 mL), washed with satd $NaHCO_3$ (3 x 200 mL, until basic pH) and brine (200 mL). The aqueous phases were extracted with ethyl acetate (400 mL) and the resulting organic layer was washed with brine (400 mL). Then the combined organic phases were dried over sodium sulfate, filtered and

evaporated. The crude product was purified by flash chromatography (hexane/ethyl acetate gradient, 60:40 to 40:60) to give **29** (2.0 g, 41%) as pale-yellow oil.

The spectroscopic data are in agreement with those reported in literature.[14]

N-(Carbobenzyloxy)-3-aminopropyl β-D-glucopyranoside (30)

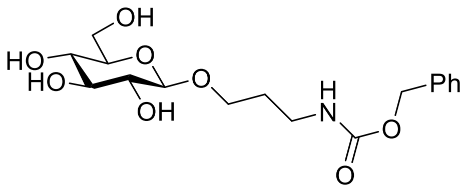

Chemical Formula: $C_{17}H_{25}NO_8$
Exact Mass: 371,16
Molecular Weight: 371,39

To a stirred solution of **29** (2.0 g, 3.70 mmol) in methanol (40 mL, 0.1 M) at 0 °C, a 0.2 M solution of sodium methoxide in methanol (18.5 mL, 3.7 mmol) was added dropwise, then the reaction was gradually warmed to room temperature. Deacetylation was confirmed by TLC analysis (hexane/ethyl acetate 1:1 for **29**, dichloromethane/methanol 85:15 for the product). After 1 h, the reaction was neutralized by addiction of an acidic ion-exchange resin (Dowex® 50WX8, H^+ form), then filtered and concentrated. The residue (1.2 g) was used in the next step without any further purification.

The spectroscopic data are in agreement with those reported in literature.[14]

N-(Carbobenzyloxy)-3-aminopropyl 4,6-*O*-benzylidene-β-D-glucopyranoside (31)

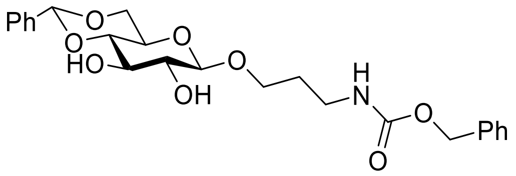

Chemical Formula: $C_{24}H_{29}NO_8$
Exact Mass: 459,19
Molecular Weight: 459,50

To a solution of crude **30** (1.2 g) in acetonitrile (30 mL, 0.1 M), benzaldehyde dimethyl acetal (1.20 mL, 8.07 mmol) and *p*-toluenesulfonic acid (0.12 g, 0.65 mmol) were added. The reaction was monitored by TLC: disappearance of the starting material was followed with dichloromethane/methanol 9:1, and product formation with hexane/ethyl acetate 3:7. After 1 h, the reaction was quenched by addition of TEA and concentrated. Flash column purification (hexane/ethyl acetate gradient, 40:60 to 30:70) gave benzylidene **31** (1.28 g, 75% over two steps) as an amorphous white solid.

The spectroscopic data are in agreement with those reported in literature.[14]

N-Benzyl-*N*-(carbobenzyloxy)-3-aminopropyl 2,3-di-*O*-benzyl-4,6-*O*-benzylidene-β-D-glucopyranoside (32)

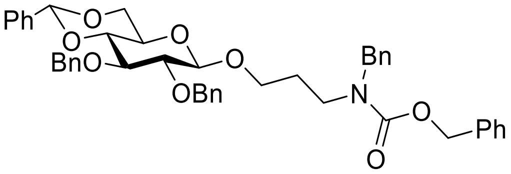

Chemical Formula: $C_{45}H_{47}NO_8$
Exact Mass: 729,33
Molecular Weight: 729,87

NaH (60% in oil, 1.06 g, 27.8 mmol) was added to a stirred solution of **31** (1.28 g, 2.78 mmol) in DMF (25 mL, 0.1 M) at room temperature. After 15 min, benzyl bromide (1.9 mL, 16.1 mmol) was added and the disappearance of starting material was followed by TLC analysis (dichloromethane/methanol 9:1, for product formation hexane/ethyl acetate 3:7). The mixture was quenched by carefully addition of methanol, then diluted with 1 N HCl (80 mL) and extracted with ethyl acetate (3×70 mL). The combined organics were washed with brine (2×70 mL), dried over Na_2SO_4 and evaporated. Flash chromatography of the crude (hexane/ethyl acetate, 80:20) gave **32** (1.68 g, 83%) as a yellow oil.

The spectroscopic data are in agreement with those reported in literature.[14]

***N*-Benzyl-*N*-(carbobenzyloxy)-3-aminopropyl 2,3,6-tri-*O*-benzyl-β-D-glucopyranoside (7)**

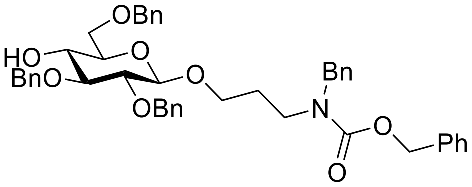

Chemical Formula: $C_{45}H_{49}NO_8$
Exact Mass: 731,35
Molecular Weight: 731,89

A suspension of compound **32** (0.50 g, 0.68 mmol)* and 4Å MS (500 mg) in dichloromethane (17 mL, 0.04M) was stirred for 10 min at room temperature, then triethylsilane (1 mL, 6.80 mmol) was added and the mixture was stirred for additional 30 min. Subsequently, $BF_3·Et_2O$ (0.42 mL, 3.40 mmol) was slowly added dropwise to the reaction mixture. After 3 h, the reaction was quenched with 0.5 mL of TEA, diluted with dichloromethane, filtered over a Celite pad and concentrated *in vacuo*. The residue was purified by flash chromatography (hexane/ethyl acetate, 70:30) to afford **7** (0.32 g, 65%) as a colourless oil.

The spectroscopic data are in agreement with those reported in literature.[14]

*Experimental evidences support that 0.5 g is the best scale for this reaction. The reaction was repeated three times to afford the necessary amount of compound **7**.

4.2 Synthesis of mannosyl acceptors 8 and 9

N-(carbobenzyloxy)-3-aminopropyl mannopyranosides **8** and **9** were obtained through a common synthetic route, starting from benzylation of glucose diacetonide **33,** followed by hydrolysis of the diacetonide and peracetylation of **35** to afford 2,4,6-tri-*O*-Acetyl-3-*O*-benzyl-β-D-glucopyranoside **36**. Glycosylation of **36** with *N*-(carbobenzyloxy)-3-aminopropanol, followed by Zemplén de-*O*-acetylation and 4,6-*O*-benzylidene protection gave glucoside **39,** which presents the 2-*O* position unprotected. Activation of the latter with sulfonylimidazole, followed by displacement with sodium azide allowed the epimerization from the *gluco* derivative **40** to the *manno* glycoside **41**. Then,

regioselective reductive opening of the benzylidene acetal of 2-azido mannoside **41** gave **8**. Conversely, azide reduction of **41** by 1,3-propanedithiol, followed by acetylation, afforded 2-*N*-acetyl-mannosamine **42**, which was subjected to benzylidene reductive cleavage to give the mannosyl acceptor **9**.

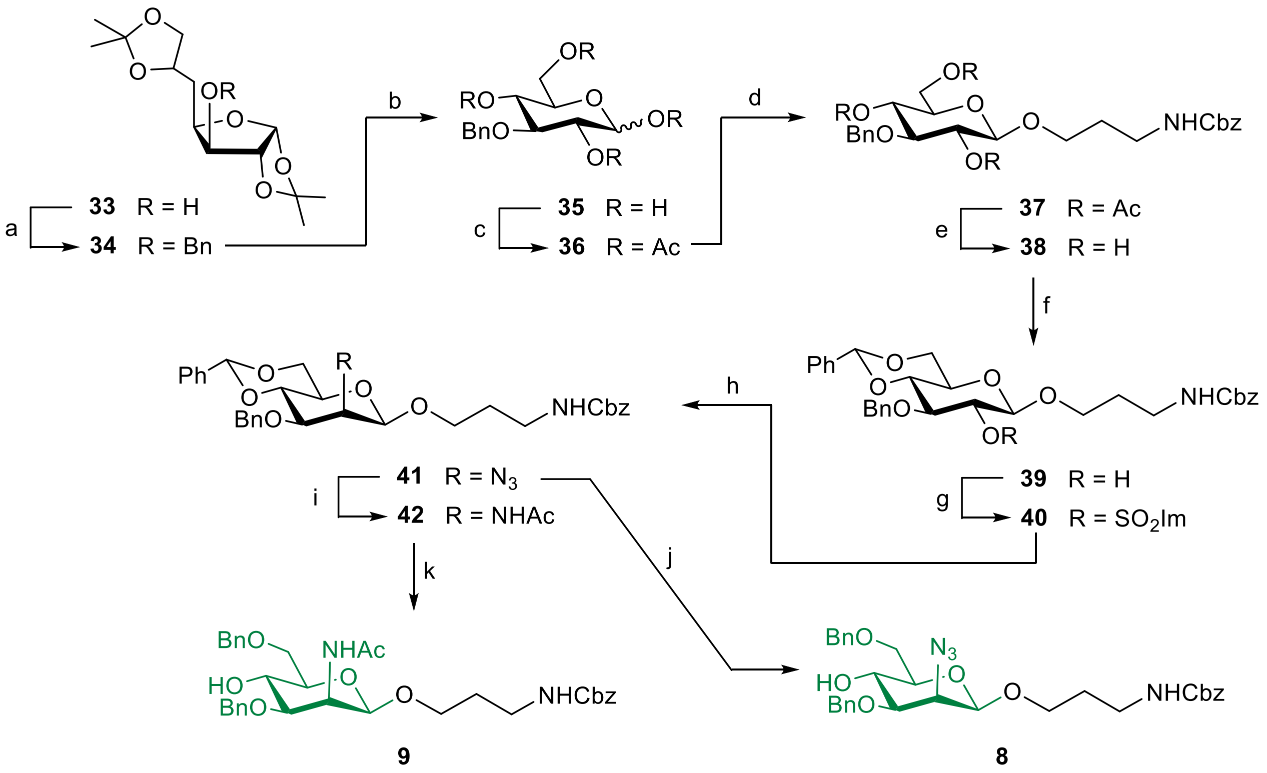

Reagents and conditions: a) BnBr, NaOH aq (50%), DMSO, **95%**; b) Dowex H^+, 1:3 EtOH/H_2O; c) Ac_2O, DMAP, Py, **80%** *over two steps*; d) *N*-Cbz-3-aminopropanol, $BF_3{\cdot}Et_2O$, DCM, **45%**; e) NaOMe/MeOH, DCM; f) $PhCH(OMe)_2$, *p*TSA, CH_3CN, **81%** *over two steps*; g) SO_2Im_2, NaH, DMF, -40°C, **81%**; h) NaN_3, DMF dry, 80°C, **70%**; i) Et_3SiH, $BF_3{\cdot}Et_2O$, DCM, 4Å MS, **79%**; j) propandithiol, DIPEA, MeOH, *then* Ac_2O, Pyr, **93%**; k) Et_3SiH, $BF_3{\cdot}Et_2O$, DCM, 4Å MS, **85%**.
DBU = 1,8-Diazabicyclo-[5.4.0]undec-7-ene; DMAP = 4-(Dimethylamino)pyridine; DIPEA = *N*,*N*-Diisopropylethylamine; *p*TSA = *p*-Toluenesulfonic acid

3-*O*-Benzyl-1,2:5,6-di-*O*-isopropylidene-α-D-glucopyranoside (34)

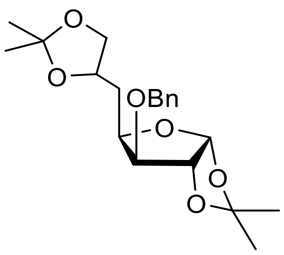

Chemical Formula: $C_{20}H_{28}O_6$
Exact Mass: 364,19
Molecular Weight: 364,44

To a solution of **33** (10 g, 38.42 mmol) in dimethyl sulfoxide (50 mL, 0.8M) a 50% aq. solution of sodium hydroxide (5.25 mL, 65.31 mmol) was added dropwise, then the reaction mixture was cooled at 0°C and freshly distilled benzyl bromide (6.85 mL, 57.63 mmol) was added. After 3 h of stirring (TLC analysis, hexane/ethyl acetate 7:3), the reaction was diluted with water (600 mL) and

extracted with ethyl acetate (4 x 300 mL). The combined organic layers were dried over Na_2SO_4 and evaporated. Flash chromatography of the crude (petroleum ether/ethyl acetate, 80:20) gave **34** (12.76 g, 95%) as a pale-yellow oil.

The spectroscopic data are in agreement with those reported in literature.[15,16]

3-*O*-Benzyl-D-glucopyranose (35)

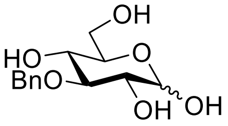

Chemical Formula: $C_{13}H_{18}O_6$
Exact Mass: 270,11
Molecular Weight: 270,28

To a stirred solution of **34** (12.75 g, 36.41 mmol) in 1:3 ethanol/water (120 mL, 0.3 M) Dowex® 50WX8 H^+ form resin (5 g) was added and the reaction was heated at reflux temperature (100°C). After 8 h, the reaction was cooled and allowed to stir overnight at room temperature. Another portion of resin was added (1 g) and after further stirring at reflux for 4 h the reaction was cooled to room temperature, filtered over a Büchner (washing with a 1:1 ethanol/water solution), and concentrated (using toluene to form an azeotrope with water). The crude product **35** (9.87 g, α/β 0.15:0.85) was used in the next step without any further purification.

The spectroscopic data are in agreement with those reported in literature.[15,17,18]

1,2,4,6-Tetra-*O*-acetyl-3-*O*-benzyl-β-D-glucopyranoside (36)

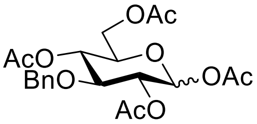

Chemical Formula: $C_{21}H_{26}O_{10}$
Exact Mass: 438,15
Molecular Weight: 438,43

To a stirred solution of **35** (9.87 g, 36.52 mmol) in pyridine (73 mL, 0.5 M) at 0 °C, acetic anhydride (34.5 mL, 365 mmol) was slowly added *via* a dropping funnel. After 24 h (TLC analysis, hexane/ethyl acetate 1:1), the reaction was diluted dichloromethane (200 mL), washed with 1N HCl until pH 5 (2 x 100 mL), then the combined aqueous layers were extracted with dichloromethane (100 mL). The combined organic layers were washed with satd $NaHCO_3$ (3 x 200 mL, until basic pH) and brine (150 mL), then dried over Na_2SO_4 and evaporated. Flash chromatography of the crude (petroleum ether/ethyl acetate gradient, 80:20 to 70:30) gave **36** (12.32 g, 77%, α/β 0.1:0.9) as a pale-yellow oil.

The spectroscopic data are in agreement with those reported in literature.[15]

***N*-(Carbobenzyloxy)-3-aminopropyl 2,4,6-tri-*O*-Acetyl-3-*O*-benzyl-β-D-glucopyranoside (37)**

To a stirred solution under argon of compound **36** (5.5 g, 12.54 mmol) and *N*-CBz-aminopropanol (2.15 g, 15.05 mmol) in dichloromethane (42 mL), boron trifluoride diethyl etherate (8.5 mL, 68.4 mmol) was added dropwise *via* a dropping funnel in two equal portions within 4 h of each other. One hour after the last addition of acid, the reaction was diluted with dichloromethane (200 mL), washed with satd $NaHCO_3$ (2 x 200 mL) and brine (200 mL). The aqueous phases were extracted

with ethyl acetate (400 mL) and the resulting organic layer was washed with brine (400 mL). Then, the combined organic phases were dried over sodium sulfate, filtered and evaporated. The crude product was purified by flash chromatography (hexane/ethyl acetate gradient, 60:40 to 40:60) to give **37** (3.23 g, 45%, recovered starting material (**36**) = 0.88 g) as a colourless oil.

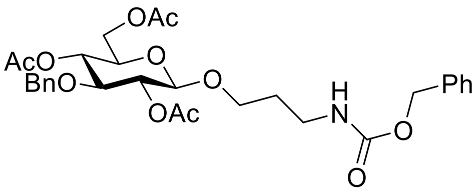

Chemical Formula: $C_{30}H_{37}NO_{11}$
Exact Mass: 587,24
Molecular Weight: 587,62

R_f = 0.32 (hexane/ethyl acetate 1:1)

$[\alpha]_D^{20}$ = -21.03 (*c* 1 in $CHCl_3$)

^{1}H NMR ($CDCl_3$) δ 7.43 – 7.18 (m, 10H, arom.), 5.19 – 5.09 (m, 3H, NCOOC*H*$_2$Ph, H-4), 5.09 – 5.02 (m, 1H, H-2), 4.66 – 4.56 (m, 2H, OC*H*$_2$Ph), 4.44 (d, $J_{1\text{-}2}$ = 7.8 Hz, 1H, H-1), 4.25 – 4.11 (m, 2H, H-6a, H-6b), 3.95 – 3.86 (m, 1H, OC*H*HCH$_2$CH$_2$N), 3.71 (t, $J_{3\text{-}2}$ = $J_{3\text{-}4}$ = 9.3 Hz, 1H, H-3), 3.64 – 3.55 (m, 2H, OCH*H*CH$_2$CH$_2$N, H-5), 3.29 (m, 2H, OCH$_2$CH$_2$C*H*$_2$N), 2.06 (br s, 3H, OCOC*H*$_3$), 2.01 (s, 3H, OCOC*H*$_3$), 1.99 (s, 3H, OCOC*H*$_3$), 1.87 – 1.70 (m, 2H, OCH$_2$C*H*$_2$CH$_2$N).

^{13}C NMR ($CDCl_3$) δ 170.78 (O*C*OCH$_3$), 169.32 (2C, O*C*OCH$_3$), 156.48 (N*C*OOCH$_2$Ph), 137.76 (quat.), 136.72 (quat.), 128.48 – 127.77 (10C, arom.), 100.84 (C-1), 79.98 (C-3), 73.80 (O*C*H$_2$Ph), 72.46 (C-2), 72.16 (C-5), 69.62 (C-4), 67.17 (O*C*H$_2$CH$_2$CH$_2$N), 66.53 (O*C*H$_2$Ph), 62.26 (C-6), 38.20 (OCH$_2$CH$_2$*C*H$_2$N), 29.52 (OCH$_2$*C*H$_2$CH$_2$N), 20.80 (OCO*C*H$_3$), 20.74 , 20.72 (OCO*C*H$_3$).

HRMS (ESI+): *m/z* for $C_{30}H_{37}NO_{11}Na$ calcd 610.2264 $[M+Na]^+$, found 610.2266.

N-(Carbobenzyloxy)-3-aminopropyl 3-*O*-benzyl-β-D-glucopyranoside (38)

A 0.2 M solution of sodium methoxide in methanol (13.5 mL) was added to a solution of **37** (3.2 g, 5.45 mmol) in methanol (54 mL) at 0 °C. The reaction was stirred for 24 h at room temperature (disappearance of the starting material was followed by TLC, hexane/ethyl acetate 1:1, and product formation with dichloromethane/methanol 9:1), then neutralized with an ion exchange resin (Dowex® 50WX8, H^+ form), filtered and concentrated. Glucopyranoside **38** (2.41 g, crude) was recovered as light-yellow oil, without any further purification.

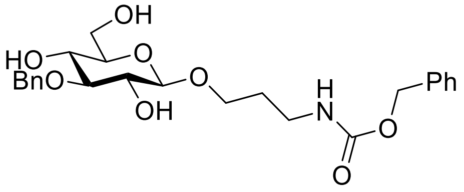

Chemical Formula: $C_{24}H_{31}NO_8$
Exact Mass: 461,20
Molecular Weight: 461,51

R_f = 0.52 (dichloromethane/methanol 9:1)

$[\alpha]_D^{20}$ = -13.66 (c 1 in CH_3OH)

^{1}H NMR (MeOD) δ 7.50 – 7.20 (m, 10H, arom.), 5.09 (br s, 2H, NCOOCH_2Ph), 4.97 – 4.81 (m, 2H, OCH_2Ph), 4.28 (d, $J_{1\text{-}2}$ = 7.3 Hz, 1H), 4.00 – 3.93 (m, 1H, OC*H*HCH_2CH_2N), 3.93 – 3.85 (m, 1H, H-6a), 3.72 – 3.58 (m, 2H, H-6b, OCH*H*CH_2CH_2N), 3.49 – 3.21 (m, 6H, H-4, H-3, H-2, OCH_2CH_2CH_2N, H-5), 1.88 – 1.75 (m, 2H, OCH_2CH_2CH_2N).

^{13}C NMR (MeOD) δ 157.57 (N*C*OOCH_2Ph), 139.04 (2C, quat.), 128.05 – 127.06 (10C, arom.), 103.03 (C-1), 84.89 (C-3), 76.50 (C-5), 74.54 (O*C*H_2Ph), 73.90 (C-2), 70.06 (C-4), 66.75 (O*C*H_2CH_2CH_2N), 65.97 (NCOO*C*H_2Ph), 61.35 (C-6), 37.44 (OCH_2CH_2*C*H_2N), 29.50 (OCH_2*C*H_2CH_2N).

HRMS (ESI+): *m/z* for $C_{24}H_{31}NO_8Na$ calcd 484.1947 $[M+Na]^+$, found 484.1945.

***N*-(Carbobenzyloxy)-3-aminopropyl 3-*O*-benzyl-4,6-*O*-benzylidene-β-D-glucopyranoside (39)**

To a solution of crude glucopyranose **38** (2.41 g, 5.45 mmol) dissolved in acetonitrile (52 mL), benzaldehyde dimethyl acetal (1.25 mL, 13.08 mmol) and *p*-toluenesulfonic acid (0.12 g, 1.05 mmol) were added. The disappearance of the starting material was monitored by TLC, dichloromethane/methanol 9:1, and product formation with hexane/ethyl acetate 6:4. After 1h, the reaction was quenched by addition of TEA and concentrated. Flash chromatography purification (hexane/ethyl acetate, 60:40) gave benzylidene **39** (2.4 g, 81% over two steps) as an amorphous white solid.

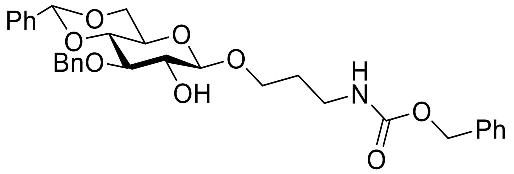

Chemical Formula: $C_{31}H_{35}NO_8$
Exact Mass: 549,24
Molecular Weight: 549,62

R_f = 0.32 (hexane/ethyl acetate 6:4)

$[\alpha]_D^{20}$ = -33.3 (c 1 in $CHCl_3$)

^{1}H NMR ($CDCl_3$) δ 7.64 – 7.16 (m, 15H, arom.), 5.59 (s, 1H, C*H*Ph), 5.23 – 5.15 (m, 1H, N*H*Cbz), 5.13 (s, 2H, NCOOCH_2Ph), 4.99 (d, J = 11.7 Hz, 1H, OC*H*HPh), 4.85 (d, J = 11.7 Hz, 1H, OCH*H*Ph), 4.40 (d, $J_{1\text{-}2}$ = 7.7 Hz, 1H, H-1), 4.36 (dd, $J_{6a\text{-}6b}$ = 10.5, $J_{6a\text{-}5}$ = 5.0 Hz, 1H, H-6a), 4.05 – 3.97 (m, 1H, OC*H*HCH_2CH_2N), 3.80 (t, $J_{6b\text{-}6a}$ = 10.5 Hz, 1H), 3.76 – 3.62 (m, 3H, OCH*H*CH_2CH_2N, H-4, H-3), 3.62 – 3.49 (m, 2H, H-2, OCH_2CH_2C*H*HN), 3.49 – 3.42 (m, 1H, H-5), 3.34 – 3.25 (m, 1H, OCH_2CH_2CH*H*N), 1.93 – 1.74 (m, 2H, OCH_2CH_2CH_2N).

^{13}C NMR ($CDCl_3$) δ 156.72 (N*C*OOCH_2Ph), 138.42 – 136.61 (3C, quat.), 129.01 – 126.02 (15C, arom.), 103.40 (C-1), 101.25 (*C*HPh), 81.25 (C-4), 80.35 (C-3), 74.60 (O*C*H_2Ph), 74.36 (C-2),

68.69 (C-6), 67.78 (O*C*$H_2$$CH_2$$CH_2$N), 66.73 (NCOO*C*$H_2$Ph), 66.43 (C-5), 38.07 (O$CH_2$$CH_2$*C*$H_2$N), 29.57 (O$CH_2$*C*$H_2$$CH_2$N).

HRMS (ESI+): *m/z* for $C_{31}H_{35}NO_8Na$ calcd 572.2260 $[M+Na]^+$, found 572.2265.

***N*-(Carbobenzyloxy)-3-aminopropyl 3-*O*-benzyl-4,6-*O*-benzylidene-2-*O*-(*N*-imidazole-1-sulfonyl)-β-D-glucopyranoside (40)**

NaH (60% in oil, 1.76 g, 44.00 mmol) was added to a stirred solution of compound **39** (2.4 g, 4.40 mmol) in DMF (50 mL) at room temperature. After 1 h, the suspension was cooled to −40 °C and a solution of 1,1′-sulfonyl-diimidazole (4.36 g, 22.00 mmol) in DMF (40 mL) was added. After 5 h, the reaction was quenched with methanol and allowed to warm to room temperature. The reaction mixture was diluted with ethyl acetate (100 mL), washed with brine (2 x 100 mL), and the combined aqueous layers were extracted with ethyl acetate (200 mL). Then, the combined organic layers were dried over sodium sulfate, filtered and evaporated. Automated flash chromatography of the crude using Biotage Isolera Prime purification system (SNAP 100g column, hexane/ethyl acetate gradient, 60:40 to 20:80) gave **40** (2.43 g, 81%) as a white solid.

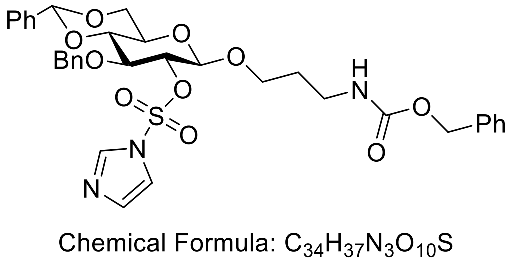

Chemical Formula: $C_{34}H_{37}N_3O_{10}S$
Exact Mass: 679,22
Molecular Weight: 679,74

R_f = 0.25 (hexane/ethyl acetate 4:6)

$[\alpha]_D^{20}$ = - 26.4 (*c* 1 in $CHCl_3$)

^{1}H NMR ($CDCl_3$) δ 7.99 (s, 1H, Imid.), 7.55 – 7.19 (m, 16H, 15H arom., Imid.), 7.05 (s, 1H, Imid.), 5.57 (s, 1H, C*H*Ph), 5.22 (br t, 1H, N*H*Cbz), 5.13 (s, 2H, NCOOC*H_2*Ph), 4.84 (d, *J* = 11.2 Hz, 1H, OC*H*HPh), 4.71 – 4.58 (m, 2H, OC*H*HPh, H-2), 4.50 (d, $J_{1\text{-}2}$ = 7.8 Hz, 1H, H-1), 4.36 (dd, $J_{6a\text{-}6b}$ = 10.5, $J_{6a\text{-}5}$ = 5.0 Hz, 1H, H-6a), 3.85 – 3.69 (m, 4H, OC*H*H$CH_2$$CH_2$N, H-6b, H-3, H-4), 3.55 – 3.47 (m, 1H, OCH*H*$CH_2$$CH_2$N), 3.46 – 3.37 (m, 1H, H-5), 3.31 – 3.21 (m, 2H, O$CH_2$$CH_2$C*$H_2$*N), 1.75 (m, 2H, O$CH_2$C*$H_2$*$CH_2$N).

^{13}C NMR ($CDCl_3$) δ 156.51 (N*C*OOCH$_2$Ph), 137.10 (2C, quat.), 136.97 (quat.), 136.69 (*C*H, Imid.), 130.27 (*C*H, Imid.), 129.25 – 125.96 (15C, arom.), 118.39 (*C*H, Imid.), 101.45 (*C*HPh), 100.28 (C-1), 85.14 (C-2), 81.64 (C-4), 76.84 (C-3), 74.60 (O*C*H_2Ph), 68.33 (O*C*$H_2$$CH_2$$CH_2$N), 68.10 (C-6), 66.58 (NCOO*C*H_2Ph), 66.17 (C-5), 37.75 (O$CH_2$$CH_2$*C*$H_2$N), 29.37 (O$CH_2$*C*$H_2$$CH_2$N).

HRMS (ESI+): *m/z* for $C_{34}H_{37}N_3O_{10}NaS$ calcd 702.2097 $[M+Na]^+$, found 702.2092.

***N*-(Carbobenzyloxy)-3-aminopropyl 2-azido-3-*O*-benzyl-4,6-*O*-benzylidene-2-deoxy-β-D-glucopyranoside (41)**

Sodium azide (1.6 g, 24.70 mmol) was added to a stirred solution of compound **40** (2.4 g, 3.53 mmol) in DMF (36 mL), and the resulting mixture was heated at 80 °C. After 6 h, the reaction mixture was cooled to room temperature, diluted with dichloromethane (50 mL) and washed with brine (2 x 30 mL). The aqueous phases were extracted with dichloromethane (100 mL) and the combined organic extracts were dried over sodium sulfate, filtered and concentrated. Automated flash chromatography of the crude by Biotage Isolera Prime purification System (SNAP 100g column, hexane/ethyl acetate gradient, 80:20 to 40:60) gave **41** (1.45 g, 70%, recovered starting material (**40**) = 183 mg) as a white solid.

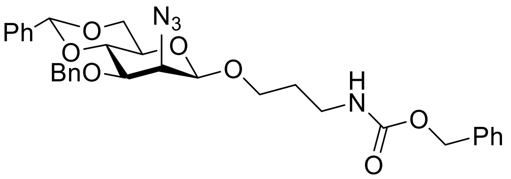

Chemical Formula: $C_{31}H_{34}N_4O_7$
Exact Mass: 574,24
Molecular Weight: 574,63

R_f = 0.28 (hexane/ethyl acetate 6:4)

$[\alpha]_D^{20}$ = -62.4 (*c* 1 in $CHCl_3$)

^{1}H NMR ($CDCl_3$) δ 7.59 – 7.21 (m, 15H, arom.), 5.61 (s, 1H, C*H*Ph), 5.19 – 5.04 (m, 3H, N*H*Cbz, NCOOC*H*$_2$Ph), 4.90 (d, *J* = 12.4 Hz, 1H, OC*H*HPh), 4.78 (d, *J* = 12.4 Hz, 1H, OCH*H*Ph), 4.56 (br s, 1H, H-1), 4.32 (dd, $J_{6a\text{-}6b}$ = 10.5, $J_{6a\text{-}5}$ = 4.9 Hz, 1H, H-6a), 4.08 – 3.93 (m, 3H, H-4, H-2, OC*H*HCH$_2$CH$_2$N), 3.88 (t, $J_{6b\text{-}6a}$ = 10.5 Hz, 1H, H-6b), 3.75 (dd, $J_{3\text{-}4}$ = 9.6, $J_{3\text{-}2}$ = 3.6 Hz, 1H, H-3), 3.66 – 3.55 (m, 1H, OCH*H*CH$_2$CH$_2$N), 3.46 – 3.25 (m, 3H, OCH2CH2C*H*$_2$N, H-5), 1.95 – 1.71 (m, 2H, OCH$_2$C*H*$_2$CH$_2$N).

^{13}C NMR ($CDCl_3$) δ 156.48 (N*C*OOCH$_2$Ph), 137.74 – 136.70 (3C, quat.), 129.03 – 126.02 (15C, arom.), 101.58 (*C*HPh), 100.25 (C-1), 78.55 (C-4), 76.29 (C-3), 72.93 (O*C*H$_2$Ph), 68.39 (C-6), 67.78 (O*C*H$_2$CH$_2$CH$_2$N), 67.32 (C-5), 66.59 (NCOO*C*H$_2$Ph), 63.41 (C-2), 38.26 (OCH$_2$CH$_2$*C*H$_2$N), 29.63 (OCH$_2$*C*H$_2$CH$_2$N).

HRMS (ESI+): *m/z* for $C_{31}H_{34}N_4O_7Na$ calcd 597.2325 $[M+Na]^+$, found 597.2328.

N-(Carbobenzyloxy)-3-aminopropyl 2-Azido-3,6-di-*O*-benzyl-2-deoxy-β-D-mannopyranoside (8)

A suspension of compound **41** (0.40 g, 0.69 mmol) and 4Å MS (500 mg) in dichloromethane (23 mL, 0.03M) was stirred for 10 min at room temperature, then triethylsilane (1.1 mL, 6.90 mmol) was added and the mixture was stirred for additional 30 min. Subsequently, $BF_3{\cdot}Et_2O$ (0.42 mL, 3.45 mmol) was very slowly added to the reaction mixture. After 2 h, the reaction was quenched with 0.5 mL of TEA, diluted with dichloromethane, filtered over a Celite pad and concentrated *in vacuo*. The residue was purified by flash chromatography (hexane/ethyl acetate, 60:40) to afford **8** (0.31 g, 79%) as a colourless oil.

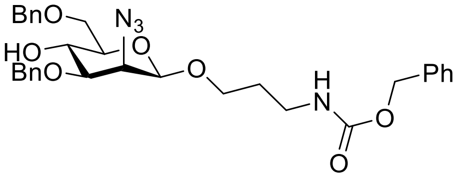

Chemical Formula: $C_{31}H_{36}N_4O_7$
Exact Mass: 576,26
Molecular Weight: 576,65

R_f = 0.34 (hexane/ethyl acetate 1:1)

$[\alpha]_D^{20}$ = -93.08 (*c* 1 in $CHCl_3$)

^{1}H NMR ($CDCl_3$) δ 7.41 - 7.24 (m, 15H, arom.), 5.38 (br s, 1H, N*H*COOCH$_2$Ph), 5.08 (br s, 2H, NCOOC*H$_2$*Ph), 4.75 (d, *J* = 11.8 Hz, 1H, OC*H$_2$*Ph), 4.63 (d, *J* = 11.8 Hz, 1H, OC*H$_2$*Ph), 4.58 – 4.50 (m, 2H, OC*H$_2$*Ph), 4.47 (br s, 1H, H-1), 4.0 – 3.87 (m, 2H, H-2, OC*H*HCH$_2$CH$_2$N), 3.82 – 3.73 (m, 2H, H-4, H-6a), 3.73 – 3.58 (m, 2H, H-6b, OCH*H*CH$_2$CH$_2$N), 3.48 – 3.34 (m, 3H, H-3, H-5, OCH$_2$CH$_2$C*H*HN), 3.34 – 3.23 (m, 1H, OCH$_2$CH$_2$CH*H*N), 1.92 – 1.69 (m, 2H, OCH$_2$C*H$_2$*CH$_2$N).

^{13}C NMR ($CDCl_3$) δ 156.70 (NH*C*OCH$_3$), 137.95 – 136.93 (3C, quat.), 128.87 – 127.90 (15C, arom.), 99.89 (C-1), 80.51 (C-3), 75.01 (C-5), 73.77 and 72.21 (2C, O*C*H$_2$Ph), 70.41 (C-6), 68.37 (C-4), 67.47 (O*C*H$_2$CH$_2$CH$_2$N), 66.66 (NCOO*C*H$_2$Ph), 61.21 (C-2), 38.17 (OCH$_2$CH$_2$*C*H$_2$N), 29.80 (OCH$_2$*C*H$_2$CH$_2$N).

HRMS (ESI+): *m/z* for $C_{31}H_{36}N_4O_7Na$ calcd 599.2482 $[M+Na]^+$, found 599.2484; *m/z* for $C_{31}H_{36}N_2O_7Na$ calcd 571.2420 $[M+Na-N_2]^+$, found 571.2424.

_N_-(Carbobenzyloxy)-3-aminopropyl 2-Acetamido-3-_O_-benzyl-4,6-_O_-benzylidene-2-deoxy-β-D-mannopyranoside (42)

1,3-Propanedithiol (0.6 mL, 12.2 mmol) and freshly distilled DIPEA (1.05 mL, 12.20 mmol) were added to a solution under Ar of compound **41** (1.00 g, 1.74 mmol) in anhydrous MeOH (6 mL). The reaction mixture (a clear solution) was stirred at room temperature for 24 h, then an additional amount of 1,3-propanedithiol (0.6 mL, 12.2 mmol) and ed DIPEA (1.05 mL, 12.2 mmol) were added. After 72h, the reaction mixture (turbid white suspension) was concentrated under a stream of air with the aid of a Drechsel apparatus refilled with bleach to trap and neutralize smelly thiols vapors. The crude was dissolved in pyridine (12 mL), and acetic anhydride (4 mL, 42.30 mmol) was added dropwise. After 24 h at room temperature, the reaction mixture was diluted with 10 mL of dichloromethane, filtered over a Celite pad and washed with aqueous HCl (1N solution, 100mL) and brine (2 x 100 mL). The organic layer was dried over sodium sulfate, filtered and concentrated. The crude product was purified with flash chromatography (hexane/ethyl acetate gradient, 30:70 to 10:90) to give **42** (0.96 g, 93%) as a white solid.

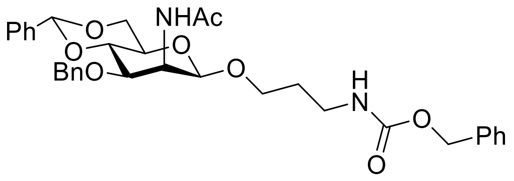

Chemical Formula: $C_{33}H_{38}N_2O_8$
Exact Mass: 590,26
Molecular Weight: 590,67

R_f = 0.25 (hexane/ethyl acetate 2:8)

$[\alpha]_D^{20}$ = -56.0 (*c* 1 in $CHCl_3$)

^{1}H NMR ($CDCl_3$) δ 7.57 – 7.20 (m, 15H, arom.), 5.86 (d, *J* = 9.2 Hz, 1H, N*H*Ac), 5.56 (s, 1H, C*H*Ph), 5.31 (br t, 1H, N*H*Cbz), 5.18 – 5.05 (m, 2H, NCOOC*H*$_2$Ph), 4.85 (br d, 1H, H-2), 4.76 (d, *J* = 12.2 Hz, 1H, OC*H*HPh), 4.63 (d, *J* = 12.2 Hz, 1H, OCH*H*Ph), 4.58 (s, 1H, H-1), 4.31 (dd, $J_{6a,6b}$ = 10.4, $J_{6a,5}$ = 4.8 Hz, 1H, H-6a), 3.93 – 3.83 (m, 1H, OC*H*HCH$_2$CH$_2$N), 3.80 – 3.69 (m, 3H, H-3, H-4, H-6b), 3.66 – 3.53 (m, 1H, OCH*H*CH$_2$CH$_2$N), 3.48 – 3.38 (m, 1H, H-5), 3.38 – 3.22 (m, 2H, OCH$_2$CH$_2$C*H*$_2$N), 2.00 (s, 3H, NHCOC*H*$_3$), 1.84 – 1.71 (m, 2H, OCH$_2$C*H*$_2$CH$_2$N).

^{13}C NMR ($CDCl_3$) δ 170.91 (NH*C*OCH$_3$), 156.50 (N*C*OOCH$_2$Ph), 137.76 – 136.70 (3C, quat.), 129.04 – 126.05 (15C, arom.), 101.61 (*C*HPh), 100.14 (C-1), 79.00 (C-4), 75.23 (C-3), 71.61 (O*C*H$_2$Ph), 68.77 (C-6), 67.83 (O*C*H$_2$CH$_2$CH$_2$N), 66.92 (C-5), 66.62 (NCOO*C*H$_2$Ph), 49.85 (C-2), 38.77 (OCH$_2$CH$_2$*C*H$_2$N), 29.46 (OCH$_2$*C*H$_2$CH$_2$N), 23.32 (NHCO*C*H$_3$).

HRMS (ESI+): *m/z* for $C_{33}H_{38}N_2O_8Na$ calcd 613.2526 $[M+Na]^+$, found 613.2529.

***N*-(Carbobenzyloxy)-3-aminopropyl 2-Acetamido-3,6-di-*O*-benzyl-2-deoxy-β-D-mannopyranoside (9)**

To a suspension of compound **42** (0.94 g, 1.59 mmol) and 4 Å MS (1 g) in dichloromethane (30 mL), triethylsilane (2.6 mL, 16.0 mmol) was added. After 30 min, a solution of $BF_3 \cdot Et_2O$ (1.00 mL, 8.00 mmol) in dichloromethane (1 mL) was added dropwise at room temperature. After 4 h, the reaction was quenched with triethylamine, filtered over celite, and concentrated under reduced pressure. The residue was purified by flash chromatography (hexane/ethyl acetate gradient, 20:80 to 100% ethyl acetate) to afford **9** (0.80 g, 85%) as a white solid.

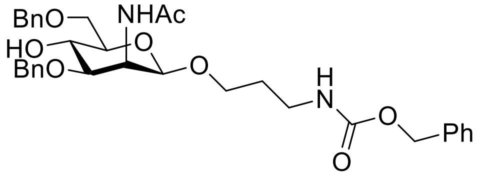

Chemical Formula: $C_{33}H_{40}N_2O_8$
Exact Mass: 592,28
Molecular Weight: 592,69

R_f = 0.35 (hexane/ethyl acetate 1:9)

$[\alpha]_D^{20}$ = - 59.9 (*c* 1 in CH_3OH)

^{1}H NMR (500 MHz, CD_3OD) δ 7.46 – 7.21 (m, 15H, arom.), 5.17 – 5.02 (m, 2H, NCOOC*H*$_2$Ph), 4.81 (d, *J* = 11.1 Hz, 1H, OC*H*HPh), 4.77 (*b*d, 1H, H-2), 4.66 – 4.53 (m, 3H, H-1, OC*H*$_2$Ph), 4.49 (d, *J* = 11.1 Hz, 1H, OCH*H*Ph), 3.91 – 3.77 (m, 3H, OC*H*HCH$_2$CH$_2$N, H-6a, H-6b), 3.69 – 3.55 (m, 2H, OCH*H*CH$_2$CH$_2$N, H-4), 3.51 (dd, $J_{3,4}$ = 9.4, $J_{3,2}$ = 4.2 Hz, 1H, H-3), 3.44 – 3.36 (m, 1H, H-5), 3.30 – 3.17 (m, 2H, OCH$_2$CH$_2$C*H*$_2$N), 1.98 (s, 3H, NHCOC*H*$_3$), 1.81 – 1.71 (m, 2H, OCH$_2$C*H*$_2$CH$_2$N).

^{13}C NMR (CD_3OD) δ 172.54 (NH*C*OCH$_3$), 157.39 (N*C*OOCH$_2$Ph), 138.23 – 137.02 (3C, quat.), 128.13 – 127.20 (15C, arom.), 99.60 (C-1), 80.06 (C-3), 75.66 (C-5), 73.28 (O*C*H$_2$Ph), 70.80 (O*C*H$_2$Ph), 69.17 (C-6), 66.45 (O*C*H$_2$CH$_2$CH$_2$N), 66.10 (C-4), 66.00 (NCOO*C*H$_2$Ph), 49.54 (C-2), 37.49 (OCH$_2$CH$_2$*C*H$_2$N), 29.32 (OCH$_2$*C*H$_2$CH$_2$N), 21.26 (NHCO*C*H$_3$).

HRMS (ESI+): *m/z* for $C_{33}H_{40}N_2O_8Na$ calcd 615.2682 $[M+Na]^+$, found 615.2683.

4.3 Synthesis of Disaccharides 10, 11 and 12

The A-B disaccharides **10**, **11** and **12** were obtained as follows through a common synthetic route.

The glycosylation reaction between glucosyl trichloroacetimidate donor **13**[19] and 4-OH glucosyl acceptor **43**[20] gave disaccharide **44** which, after *O*-deacetylation (**45**) and activation with sulfonyl diimidazole (**46**), was epimerized via nucleophilic substitution with sodium azide providing disaccharide **47** with the desired *manno* β-(1→4) configuration. Trichloroacetimidate **10** was obtained from **47** in good overall yield by removal of the *O*-allyl group (**48**) followed by activation of the anomeric position with trichloroacetonitrile (**10**). On the other side, disaccharides **11** and **12** were synthetized from **47** by initial azide reduction to give compound **49**. Then, hydrolysis of the allyl group gave disaccharide **11**, which was in turn activated with trichloroacetonitrile to disaccharide donor **12**.

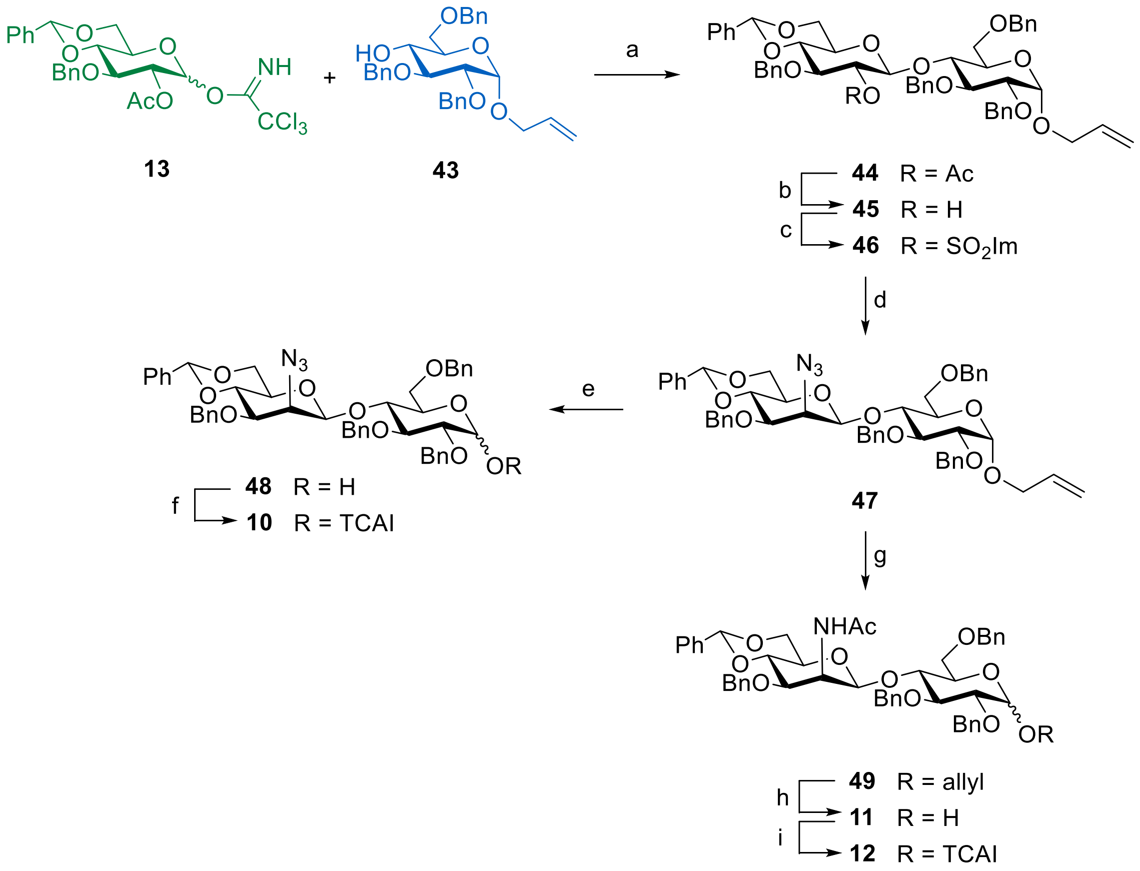

Reagents and conditions: a) TESOTf, DCM, 4Å MS, -20°C, **98%**; b) NaOMe/MeOH, DCM; c) SO_2Im_2, $NaH_{60\%}$, DMF, -40°C, **86%** *over two steps*; d) NaN_3, DMF, 80°C, **89%**; e) Ir cat., THF, H_2 then I_2, THF/H_2O, **86%**; f) CCl_3CN, DBU, DCM, **97%**; g) Zn, $CuSO_4{\cdot}5H_2O$, THF/Ac_2O/AcOH, **56%**; h) Ir cat., THF, H_2 then I_2, THF/H_2O, **94%**; i) CCl_3CN, DBU, DCM, **88%**.
TCAI = -(HN=)C-CCl_3, trichloroacetimidate; DBU = 1,8-Diazabicyclo[5.4.0]undec-7-ene;
Ir cat.= (1,5-Cyclooctadiene)bis(methyldiphenylphosphine)iridium(I) hexafluorophosphate

4.3.1 Synthesis of glucosyl acceptor 43

Based on literature procedures[20–24] modified according to our standard lab protocols, known allyl 2,3,6-tri-*O*-benzyl-α-D-glucopyranoside **43** was prepared in four steps from α-D-glucose **50** in 33% overall yield. D-Glucose **50** was first allylated to α/β allyl D-glucopyranoside **51**, which was then treated with benzaldehyde dimethylacetal in the presence of an acid catalysis to afford 4,6-*O*-benzylidene acetal **52** in 61% yield over two steps. Compound **52** was then benzylated to glucopyranoside **53**, which was obtained in a 1:1 α/β mixture of anomers. The pure alpha anomer **53α** was separated by repeated flash chromatographies and subjected to regioselective reductive opening of the benzylidene acetal, mediated by Et_3SiH/TFA, to afford the desired 4-OH glucosyl acceptor **43** in 80% yield.

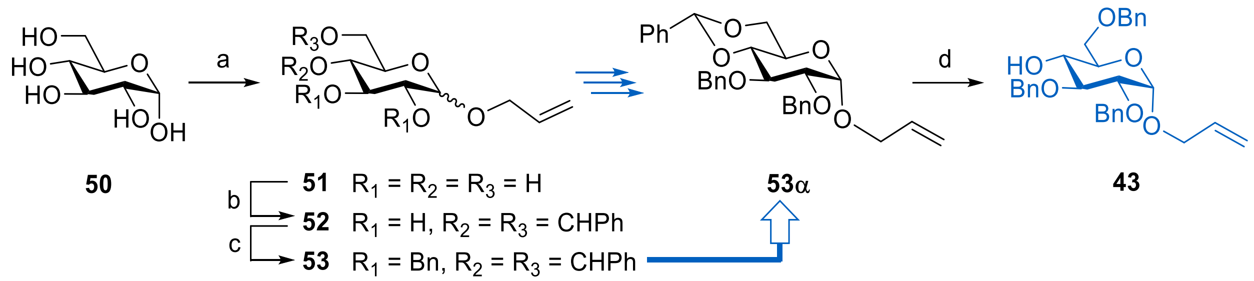

Reagents and conditions: a) allyl alcohol, Amberlite IRA-120 H^+, 95°C; b) $PhCH(OMe)_2$, *p*TSA, DMF, 40°C, **61%** *over two steps*; c) BnBr, $NaOH_{50\%}$, DMSO, **64%**; d) Et_3SiH, TFA, DCM, 0°C to r.t, **80%**. *p*TSA = *p*-Toluenesulfonic Acid; DMSO = Dimethyl sulfoxide; TFA = Trifluoroacetic acid; ⇨ = *flash chromatography*

Allyl 4,6-*O*-benzylidene-D-glucopyranoside (52)

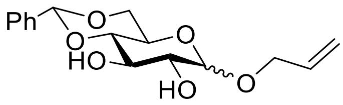

Chemical Formula: $C_{16}H_{20}O_6$
Exact Mass: 308,13
Molecular Weight: 308,33

A suspension of commercially available D-glucose **50** (10,50 g, 58.00 mmol), Amberlite IR-120 H^+ resin (10.50 g) and allylic alcohol (60 mL) was heated at reflux temperature (97 °C). After 30 min, the reaction mixture turned to a clear orange solution and was monitored by TLC analysis (ethyl acetate/isopropanol/water 3:3:0.3). After 5 h, the heterogeneous mixture was filtered and concentrated. Crude **51** (15.8 g, caramel coloured amorphous solid) was dissolved in DMF, then benzaldehyde dimethyl acetal (16 mL, 0.16 mol) and *p*-toluenesulfonic acid (1.10 g, 5.80 mmol) were added. The flask was connected to the water pump through a junction, and the reaction was stirred at reduced pressure at 40 °C to remove methanol (TLC analysis: ethyl acetate/isopropanol 8:2 for **51**, and petroleum ether/ethyl acetate 3:7 for the product). After 5 h, the reaction was quenched with satd $NaHCO_3$ (150 mL) and extracted with chloroform (4 x 100 mL). The combined organic phases were washed with water, then dried over sodium sulfate, filtered and evaporated. The crude product was purified by flash chromatography (petroleum ether/ethyl acetate gradient, 60:40 to 30:70) to give **52** (10.90 g, 61% *over two steps*, white solid) as a 6:4 mixture of α,β-anomers.

The spectroscopic data are in agreement with those reported in literature.[22]

***Allyl* 2,3-di-*O*-benzyl-4,6-*O*-benzylidene-D-glucopyranoside (53)**

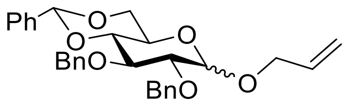

Chemical Formula: $C_{30}H_{32}O_6$
Exact Mass: 488,22
Molecular Weight: 488,58

To a solution of **52** (10.40 g, 33.70 mmol) in dimethyl sulfoxide (60 mL), a 50% aq solution of sodium hydroxide (18 mL, 0.23 mol) and benzyl bromide (20 mL, 0.17 mol) were added at 0°C. The reaction was gradually warmed to room temperature and stirred for 24 h (TLC analysis: petroleum ether/ethyl acetate 3:7 for **52**, and petroleum ether/ethyl acetate 9:1 for the product).

Then, the mixture was diluted with water, quenched with satd $NaHCO_3$ (100 mL) and extracted with dichloromethane (5 x 150 mL). The combined organic phases were washed with brine (2 x 250 mL), then dried over sodium sulfate, filtered and evaporated. The crude (25 g) was purified by flash chromatography (petroleum ether/ethyl acetate, 90:10) to give **53** (10.50 g, 64%) as a colourless glassy solid. During the purification it was possible to obtain pure fractions of both the α (major) and β-anomers.

The spectroscopic data are in agreement with those reported in literature.[22]

Allyl 2,3,6-tri-*O*-benzyl-α-D-glucopyranoside (43)

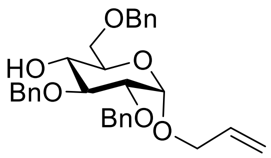

Chemical Formula: $C_{30}H_{34}O_6$
Exact Mass: 490,24
Molecular Weight: 490,60

Pure α anomer of compound **53** (1.00 g, 2.05 mmol) was dissolved in dichloromethane (10 mL) and Et_3SiH (1.65 mL, 10.25 mmol) was added dropwise. After 30 min, the mixture was cooled to 0 °C and trifluoracetic acid (0.79 mL, 10.25 mmol) was slowly added. The reaction was gradually warmed to room temperature and stirred for 2 h. Then, the reaction was diluted with dichloromethane, and quenched by the addiction of a saturated aqueous solution of $NaHCO_3$. The aqueous phase was extracted with dichloromethane (2 x 50 mL), and the combined organic phases were dried over Na_2SO_4. The solvent was removed under reduced pressure. The crude product was purified by automated flash chromatography (Biotage Isolera Prime, SNAP 50g column, hexane/ethyl acetate gradient, 97:3 to 50:50) to give **43** (0.81 g, 80%) as a colourless oil.

The spectroscopic data are in agreement with those reported in literature.[20]

4.3.2 Synthesis of trichloroacetimidate 13

Known trichloroacetimidate **13** was synthetized in six steps from D-glucose diacetonide **33** in 36% overall yield, following a literature procedure[19] modified according to our lab protocols. In particular, 3-*O*-benzyl-D-glucopyranose (**35**), described above, was subjected to 4,6-*O*-benzylidene protection to give compound **54**, which was acetylated to **55**. Subsequently, the anomeric acetate was regioselectively removed with ethylenediamine acetate to give hemiacetal **56**, and reaction with trichloroacetonitrile under standard conditions afforded donor **13**.

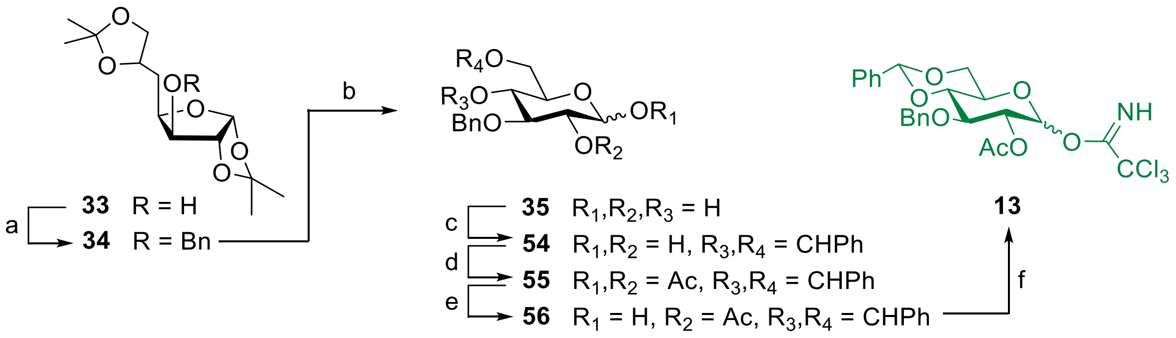

Reagents and conditions: a) BnBr, NaOH aq (50%), DMSO, **95%**; b) Dowex H^+, 1:3 $EtOH/H_2O$, **99%**; c) $PhCH(OMe)_2$, *p*TSA, DMF, 60°C, **60%**; d) Ac_2O, DMAP, Py, **97%**; e) $NH_2(CH_2)_2NH_2$, AcOH, THF, +10°C, **70%**; f) Cl_3CCN, DBU, DCM, **95%**.
*p*TSA = *p*-Toluenesulfonic acid; DMAP = 4-(Dimethylamino)pyridine; DBU = 1,8-Diazabicyclo[5.4.0]undec-7-ene

1,2-di-*O*-Acetyl-3-*O*-benzyl-4,6-*O*-benzylidene-D-glucopyranose (55)

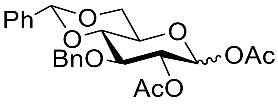

Chemical Formula: $C_{24}H_{26}O_8$
Exact Mass: 442,16
Molecular Weight: 442,46

To a solution of crude **35** (36.53 mmol) in DMF (70 mL, 0.5 M), benzaldehyde dimethyl acetal (16.45 mL, 0.11 mol) and *p*-toluenesulfonic acid (2.10 g, 10.95 mmol) were added. The flask was connected to the water pump through a junction, and the reaction was stirred at reduced pressure to remove methanol for 5 h at 45°C (disappearance of starting material was followed by TLC, dichloromethane/methanol 85:15, and product formation with hexane/ethyl acetate 1:1). Then the reaction was quenched by addition of TEA and concentrated. Flash column purification (hexane/ethyl acetate gradient, 40:60 to 30:70) gave 3-*O*-Benzyl-4,6-*O*-benzylidene-α/β-D-glucopyranose (**54**, 7.85 g, 60%) as an amorphous white solid. Compound **54** (5.56 g, 15.50 mmol) was dissolved in pyridine (30 mL, 0.5 M) and cooled to 0°C. Acetic anhydride (14.60 mL, 0.16 mol) was slowly added to the reaction *via* a dropping funnel. After 24 h (TLC analysis, hexane/ethyl acetate 7:3), the reaction was diluted dichloromethane (150 mL), washed with 1N HCl until pH 5 (2 x 100 mL), then the combined aqueous layers were extracted with dichloromethane (50 mL). The combined organic layers were washed with satd $NaHCO_3$ (3 x 200 mL, until basic pH) and brine (150 mL), then dried over Na_2SO_4 and evaporated. Flash chromatography of the crude (petroleum ether/ethyl acetate gradient, 80:20 to 70:30) gave **55** (6.65 g, 97%, α/β 6:4) as a white solid.

The spectroscopic data are in agreement with those reported in literature.[19]

2-*O*-Acetyl-3-*O*-benzyl-4,6-*O*-benzylidene-D-glucopyranose (56)

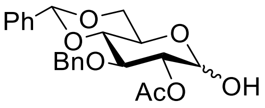

Chemical Formula: $C_{22}H_{24}O_7$
Exact Mass: 400,15
Molecular Weight: 400,43

A solution of glucoside **55** (6.60 g, 15.0 mmol) in tetrahydrofuran (40 mL) was cannulated into a solution of ethylenediamine acetate freshly prepared by adding dropwise acetic acid (1.70 mL, 30.00 mmol) into a cooled (0 °C) solution of ethylenediamine (1.84 mL, 30.00 mmol) in tetrahydrofuran (10 mL). The reaction mixture was stirred for 5 h at 10 °C, then diluted with dichloromethane (200 mL) and washed with satd $NaHCO_3$ (2 x 150 mL) and brine (150 mL), dried over sodium sulfate, filtered and evaporated. The residue was purified by automated flash chromatography (Biotage Isolera Prime, SNAP 100g column, hexane/ethyl acetate gradient, 95:5 to 40:60) to give **56** (4.20 g, 70%, α/β = 6:4) as a colourless syrup.

The spectroscopic data are in agreement with those reported in literature.[19]

2-*O*-Acetyl-3-*O*-benzyl-4,6-*O*-benzylidene-α/β-D-glucopyranosyl Trichloroacetimidate (13)

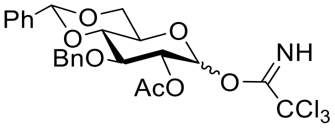

Chemical Formula: $C_{24}H_{24}Cl_3NO_7$
Exact Mass: 543,06
Molecular Weight: 544,81

To a solution of compound **56** (4.20 g, 10.49 mmol) in dichloromethane (70 mL) trichloroacetonitrile (5.30 mL, 52.86 mmol) was added, followed by addition of few drops of DBU (1,8-Diazabicyclo(5.4.0)undec-7-ene). The reaction was stirred at room temperature for 1 h, then concentrated under reduced pressure. The crude was purified by flash chromatography (hexane/ethyl acetate, 85:15 + 1% of TEA) to give donor **13** (5.43 g, 95%, white solid) as a mixture of α,β-anomers.

The spectroscopic data are in agreement with those reported in literature.[19]

Allyl (2-*O*-Acetyl-3-*O*-benzyl-4,6-*O*-benzylidene-β-D-glucopyranosyl)-(1→4)-2,3,6-tri-*O*-benzyl-α-D-glucopyranoside (44)

Allyl glucosyl acceptor **43** (0.80 g, 1.63 mmol), glucosyl trichloroacetimidate **13** (1.33 g, 2.44 mmol) and freshly dried 4Å molecular sieves (1 g) were suspended in dichloromethane (15 mL), stirred under argon for 20 min and then the mixture was cooled to -20 °C. Trimethylsilyl trifluoromethanesulfonate (0.1M solution in dichloromethane, 3.26 mL) was added dropwise and the disappearance of the starting material was followed by TLC (toluene/ethyl acetate 9:1 + 1% triethylamine; hexane/ethyl acetate 8:2 + 1% triethylamine). After 15 min, the reaction was quenched with TEA, diluted with dichloromethane, filtered over Celite, and the solvent evaporated. The crude product was purified by automated flash chromatography using a Biotage Isolera Prime purification system (SNAP 100g column, hexane/ethyl acetate gradient, 95:5 to 60:40) to give **44** (1.39 g, 98%) as an amorphous white solid.

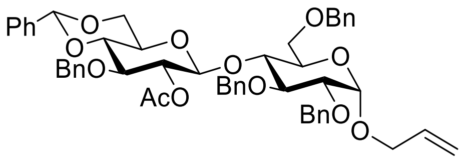

Chemical Formula: $C_{52}H_{56}O_{12}$
Exact Mass: 872,38
Molecular Weight: 873,01

R_f = 0.29 (hexane/ethyl acetate 8:2) and 0.26 (toluene/ethyl acetate 9:1)

$[\alpha]_D^{20}$ = +14.72 (*c* 1 in $CHCl_3$)

^{1}H NMR ($CDCl_3$) δ 7.54 – 7.24 (m, 25H, arom.), 6.01 – 5.90 (m, 1H, $OCH_2CH{=}CH_2$), 5.50 (s, 1H, C*H*Ph), 5.35 (br d, 1H, $OCH_2CH{=}CHH$), 5.25 (br d, 1H, $OCH_2CH{=}CHH$), 4.97 – 4.73 (m, 7H, H-2', 5x OC*H*HPh, H-1), 4.65 (br dd, 2H, 2x OC*H*HPh), 4.46 (d, $J_{1'\text{-}2'}$ = 8.0 Hz, 1H, H-1'), 4.41 (d, *J* = 12.0 Hz, 1H, OCH*H*Ph), 4.21 – 4.11 (m, 2H, H-6'a, OC*H*HCH=CH_2), 4.03 (br dd, 1H, OCH*H*CH=CH_2), 3.92 – 3.84 (m, 2H, H-4, H-3), 3.77 (dd, $J_{6a\text{-}6b}$ = 10.6, $J_{6a\text{-}5}$ = 2.8 Hz, 1H, H-6a), 3.72 – 3.63 (m, 2H, H-5, H-4'), 3.59 (dd, $J_{6b\text{-}6a}$ = 10.6, $J_{6b\text{-}5}$ = 1.6 Hz, 1H, H-6b), 3.56 – 3.41 (m, 3H, H-2, H-3', H-6b'), 3.18 – 3.10 (m, 1H, H-5'), 1.95 (s, 3H, $OCOCH_3$).

^{13}C NMR ($CDCl_3$) δ 169.07 (O*C*OCH_3), 139.44 – 137.30 (5C, quat.), 133.74 ($OCH_2CH{=}CH_2$), 129.03 – 126.05 (25 C, arom.), 118.22 ($OCH_2CH{=}CH_2$), 101.17 (*C*HPh), 100.82 (C-1'), 95.85 (C-1), 81.63 (C-4'), 79.81 (C-4), 79.09 (C-2), 78.59 (C-3'), 77.12 (C-3), 75.27 (O*C*H_2Ph), 73.95 (O*C*H_2Ph), 73.66 (O*C*H_2Ph), 73.37 (C-2'), 73.35 (O*C*H_2Ph), 70.10 (C-5), 68.62 (C-6'), 68.43 ($OCH_2CH{=}CH_2$), 67.56 (C-6), 65.93 (C-5'), 20.91 ($OCOCH_3$).

HRMS (ESI+): *m/z* for $C_{52}H_{56}O_{12}Na$ calcd 895.3669 $[M+Na]^+$, found 895.3654.

Allyl 3-*O*-benzyl-4,6-*O*-benzylidene-β-D-glucopyranosyl-(1→4)-2,3,6-tri-*O*-benzyl-α-D-glucopyranoside (45)

According to general Procedure A compound **44** (1.38 g, 1.58 mmol) was de-*O*-acetylated affording **45**. Automated flash chromatography (Biotage Isolera Prime, SNAP 100g column, hexane/ethyl acetate gradient, 95:5 to 60:40) gave **45** (1.05 g, 80%) as colourless oil.

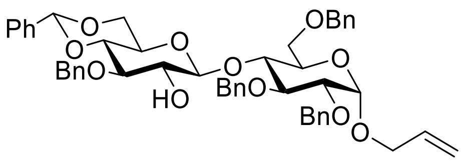

Chemical Formula: $C_{50}H_{54}O_{11}$
Exact Mass: 830,37
Molecular Weight: 830,97

R_f = 0.27 (hexane/ethyl acetate 8:2)

$[\alpha]_D^{20}$ = +19.08 (*c* 1 in $CHCl_3$)

^{1}H NMR ($CDCl_3$) δ 7.54 – 7.22 (m, 25H, arom.), 6.02 – 5.92 (m, 1H, $OCH_2C\textit{H}=CH_2$), 5.50 (s, 1H, C*H*Ph), 5.35 (br d, 1H, $OCH_2CH=C\textit{H}H$), 5.26 (br d, 1H, $OCH_2CH=CH\textit{H}$), 4.99 – 4.88 (m, 3H, $OC\textit{H}_2Ph$, 1x OC*H*HPh), 4.83 (d, $J_{1\text{-}2}$ = 3.5 Hz, 1H, H-1), 4.82 – 4.48 (m, 6H, 5x OC*H*HPh, H-1'), 4.18 (br dd, 1H, $OC\textit{H}HCH=CH_2$), 4.08 – 3.93 (m, 5H, $OCH\textit{H}CH=CH_2$, H-6a, H-6a', H-3, H-4), 3.87 (m, 1H, H-5), 3.70 (br d, 1H, H-6b), 3.64 – 3.43 (m, 5H, H-4', H-2, H-6b', H-2', H-3'), 3.17 – 3.09 (m, 1H, H-5'), 3.02 (br s, 1H, O*H*).

^{13}C NMR ($CDCl_3$) δ 139.37 – 137.44 (5C, quat.), 133.77 ($OCH_2\textit{C}H=CH_2$), 128.94 – 126.09 (25C, arom.), 118.11 ($OCH_2CH=\textit{C}H_2$), 103.55 (C-1'), 101.21 (*C*HPh), 95.88 (C-1), 81.32 (C-4'), 80.61 (C-4), 80.38 (C-3'), 79.60 (C-2), 77.63 (C-3), 75.19 ($O\textit{C}H_2Ph$), 75.16 (C-2'), 74.45 ($O\textit{C}H_2Ph$), 73.64 ($O\textit{C}H_2Ph$), 73.33 ($O\textit{C}H_2Ph$), 69.89 (C-5), 68.70 (C-6'), 68.51 (C-6), 68.40 ($O\textit{C}H_2CH=CH_2$), 66.35 (C-5').

HRMS (ESI+): *m/z* for $C_{50}H_{54}O_{11}Na$ calcd 853.3564 $[M+Na]^+$, found 853.3553.

Allyl 3-*O*-benzyl-4,6-*O*-benzylidene-2-*O*-(*N*-imidazole-1-sulfonyl)-β-D-glucopyranosyl-(1→4)-2,3,6-tri-*O*-benzyl-α-D-glucopyranoside (46)

As described above in general Procedure B, compound **45** (1.04 g, 1.25 mmol) was activated as 2-O-sulfonylimidazole obtaining **46**. Automated flash chromatography by Biotage Isolera Prime purification system (SNAP 50g column, hexane/ethyl acetate gradient, 95:5 to 50:50) gave **46** (0.96 g, 80%) as a colourless sticky gel.

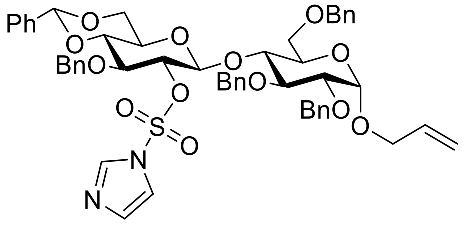

Chemical Formula: $C_{53}H_{56}N_2O_{13}S$
Exact Mass: 960,35
Molecular Weight: 961,09

R_f = 0.25 (hexane/ethyl acetate 7:3)

$[\alpha]_D^{20}$ = +4.47 (*c* 1 in $CHCl_3$)

^{1}H NMR ($CDCl_3$) δ 7.90 (s, 1H, Imid.), 7.52 – 7.23 (m, 25H, arom.), 7.19 (s, 1H, Imid.), 7.06 (s, 1H, Imid.), 6.09 – 5.96 (m, 1H, $OCH_2C\textit{H}=CH_2$), 5.48 (s, 1H, C*H*Ph), 5.42 (br d, 1H, $OCH_2CH=C\textit{H}H$), 5.31 (br d, 1H, $OCH_2CH=CH\textit{H}$), 4.88 – 4.73 (m, 6H, OC*H*HPh, H-1), 4.70 – 4.60 (m, 2H, OCH*H*Ph), 4.47 (t, $J_{2'\text{-}1'} = J_{2'\text{-}3'}$ = 8.6 Hz, 1H, H-2'), 4.31 – 4.19 (m, 4H, OCH*H*Ph, H-1', $OC\textit{H}HCH=CH_2$, H-6a'), 4.03 (br dd, 1H, $OCH\textit{H}CH=CH_2$), 3.90 (t, $J_{4\text{-}3} = J_{4\text{-}5}$ = 9.5 Hz, 1H, H-4), 3.78 (t, $J_{3\text{-}2} = J_{3\text{-}4}$ = 9.5 Hz, 1H, H-3), 3.60 (t, $J_{4'\text{-}3'} = J_{4'\text{-}5'}$ = 8.8 Hz, 1H, H-4'), 3.55 – 3.32 (m, 6H, H-2, H-3', H-6a, H-6b, H-6b', H-5), 3.06 – 2.96 (m, 1H, H-5').

^{13}C NMR ($CDCl_3$) δ 139.21 – 136.92 (5C, quat.), 136.86 (*C*H, Imid.), 133.71 ($OCH_2\textit{C}H=CH_2$), 130.39 (*C*H, Imid.), 129.22 – 126.03 (25C, arom.), 118.51 (*C*H, Imid.), 118.37 ($OCH_2CH=\textit{C}H_2$), 101.41 (*C*HPh), 98.47 (C-1'), 95.73 (C-1), 85.44 (C-2'), 81.83 (C-4'), 79.35 (C-3), 79.17 (C-2),

77.14 (C-3'), 75.82 (C-4), 75.36 (OCH_2Ph), 74.40 (OCH_2Ph), 73.66 (OCH_2Ph), 73.33 (OCH_2Ph), 69.11 (C-5), 68.41 (C-6', OCH_2CH=CH_2), 67.26 (C-6), 65.79 (C-5').

HRMS (ESI+): *m/z* for $C_{53}H_{56}N_2O_{13}NaS$ calcd 983.3401 $[M+Na]^+$, found 983.3394.

Allyl (2-azido-3-*O*-benzyl-4,6-*O*-benzylidene-2-deoxy-β-D-mannopyranosyl)-(1→4)-2,3,6-tri-*O*-benzyl-α-D-glucopyranoside (47)

Compound **47** was prepared from compound **46** (0.95 g, 0.99 mmol) as described in general Procedure D. Automated flash chromatography of the crude by Biotage Isolera Prime purification system (SNAP 50g column, hexane/ethyl acetate gradient, 93:7 to 50:50) afforded **47** (0.75 g, 89%) as an amorphous white solid.

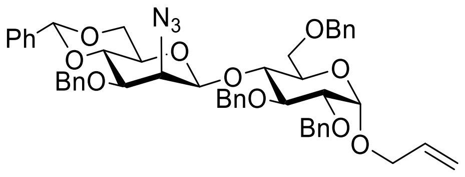

Chemical Formula: $C_{50}H_{53}N_3O_{10}$
Exact Mass: 855,37
Molecular Weight: 855,99

R_f = 0.35 (hexane/ethyl acetate 7:3)

$[\alpha]_D^{20}$ = -1.45 (*c* 1 in $CHCl_3$)

^{1}H NMR ($CDCl_3$) δ 7.53 – 7.19 (m, 25H, arom.), 6.01 – 5.91 (m, 1H, OCH_2CH=CH_2), 5.52 (s, 1H, C*H*Ph), 5.36 (br dd, 1H, OCH_2CH=C*H*H), 5.26 (br dd, 1H, OCH_2CH=CH*H*), 4.97 (d, *J* = 10.8 Hz, 1H, OC*H*HPh), 4.92 (d, *J* = 10.8 Hz, 1H, OCH*H*Ph), 4.84 (d, $J_{1\text{-}2}$ = 3.5 Hz, 1H, H-1), 4.81 – 4.60 (m, 5H, OC*H*HPh), 4.58 (d, $J_{1'\text{-}2'}$ = 1.1 Hz, 1H, H-1'), 4.40 (d, *J* = 12.0 Hz, 1H, OC*H*HPh), 4.19 (br ddt, 1H, OC*H*HCH=CH_2), 4.08 – 3.88 (m, 5H, OCH*H*CH=CH_2, H-6a', H-3, H-4, H-4'), 3.85 (br dt, 1H, H-5), 3.78 – 3.70 (m, 2H, H-6a, H-2'), 3.65 (dd, $J_{6b\text{-}6a}$ = 10.9, $J_{6b\text{-}5}$ = 1.9 Hz, 1H, H-6b), 3.61 – 3.52 (m, 2H, H-6b', H-2), 3.44 (dd, $J_{3'\text{-}4'}$ = 9.6, $J_{3'\text{-}2'}$ = 3.7 Hz, 1H, H-3'), 3.04 – 2.95 (m, 1H, H-5').

^{13}C NMR ($CDCl_3$) δ 139.27 – 137.37 (5C, quat.), 133.65 (OCH_2CH=CH_2), 128.98 – 126.06 (25C, arom.), 118.30 (OCH_2CH=CH_2), 101.49 (*C*HPh), 100.16 (C-1'), 95.80 (C-1), 80.17 (C-3), 79.42 (C-2), 78.44 (C-4'), 77.38 (C-4), 76.65 (C-3'), 75.21 (OCH_2Ph), 73.63 (OCH_2Ph), 73.34 (OCH_2Ph), 72.73 (OCH_2Ph), 69.53 (C-5), 68.42 (C-6), 68.38 (C-6', OCH_2CH=CH_2), 67.18 (C-5'), 63.64 (C-2').

HRMS (ESI+): *m/z* for $C_{31}H_{36}N_4O_7Na$ calcd 878.3629 $[M+Na]^+$, found 878.3639; *m/z* for $C_{31}H_{36}N_2O_7Na$ calcd 850.3567 $[M+Na\text{-}N_2]^+$, found 850.3578.

2-Azido-3-*O*-benzyl-4,6-*O*-benzylidene-2-deoxy-β-D-mannopyranosyl-(1→4)-2,3,6-tri-*O*-benzyl-D-glucopyranose (48)

Compound **48** was obtained from allyl glycoside **47** (0.84 g, 0.98 mmol) as described in general Procedure F. Flash chromatography of the crude (hexane/ethyl acetate gradient, 70:30 to 50:50) afforded **48** (0.69 g, 86%) as an amorphous white solid in a 4:1 α/β mixture.

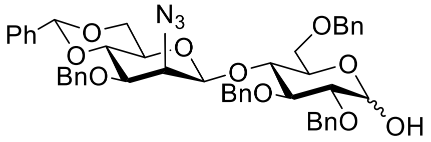

Chemical Formula: $C_{47}H_{49}N_3O_{10}$
Exact Mass: 815,34
Molecular Weight: 815,92

R_f α = R_f β = 0.20 (hexane/ethyl acetate 7:3)

α/β = 0.80:0.20

^{1}H NMR ($CDCl_3$) δ 7.68 – 7.07 (m, 25H, arom.), 5.53 (s, 0.80H, C*H*Ph α), 5.52 (s, 0.20H, C*H*Ph β), 5.23 (d, $J_{1\text{-}2}$ = 3.6 Hz, 0.80H, H-1 α), 5.07 – 4.32 (m, 9.20H, H-1 β, H-1’, 4 x OCH_2Ph), 4.13 – 3.85 (m, 4.60H, H-5 α, H-6a’, H-4, H-4’, H-3 α), 3.83 – 3.62 (m, 3.20H, H-2’ α, H-2’ β, H-6a, H-6b, H-3 β), 3.62 – 3.50 (m, 2H, H-6b’, H-2 α, H-5 β), 3.50 – 3.32 (m, 1.20H, H-3’, H-2 β), 3.09 – 2.92 (m, 2H, H-5’, O*H*).

^{13}C NMR ($CDCl_3$) δ 139.06 – 137.37 (5C, quat.), 128.99 – 126.06 (25C, arom.), 101.51 (*C*HPh), 100.15 (C-1’), 97.50 (0.20C, C-1 β), 91.34 (0.80C, C-1 α), 82.80 (0.20C, C-3 β), 82.76 (0.20C, C-2 β), 79.97 (0.80C, C-3 α), 79.44 (0.80C, C-2 α), 78.44 (0.80C, C-4’ α), 78.41 (0.20C, C-4’ β), 77.40 (0.20C, C-4 β), 77.27 (0.80C, C-4 α), 76.60 (C-3’), 75.26 (0.80C, O*C*H_2Ph α), 75.22 (0.20C, O*C*H_2Ph β), 74.85 (0.20C, O*C*H_2Ph β), 74.26 (0.20C, C-5 β), 73.73 (0.20C, O*C*H_2Ph β), 73.68 (0.80C, O*C*H_2Ph α), 73.55 (0.80C, O*C*H_2Ph α), 72.78 (0.20C, O*C*H_2Ph β), 72.74 (0.80C, O*C*H_2Ph α), 69.70 (0.80C, C-5 α), 68.60 (0.20C, C-6 β), 68.45 (0.80C, C-6 α), 68.38 (0.80C, C-6’ α), 68.33 (0.20C, C-6’ β), 67.22 (0.20C, C-5’ β), 67.19 (0.80C, C-5’ α), 63.61 (0.80C, C-2’ α), 60.85 (0.20C, C-2’ β).

HRMS (ESI+): *m/z* for $C_{47}H_{49}N_3O_{10}Na$ calcd 838.3316 $[M+Na]^+$, found 838.3307; *m/z* for $C_{47}H_{49}NO_{10}Na$ calcd 810.3254 $[M+Na-N_2]^+$, found 810.3243.

2-Azido-3-*O*-benzyl-4,6-*O*-benzylidene-2-deoxy-β-D-mannopyranosyl-(1→4)-2,3,6-tri-*O*-benzyl-D-glucopyranosyl trichloroacetimidate (10)

To a solution of compound **48** (0.65 g, 0.78 mmol) in dichloromethane (8 mL), trichloroacetonitrile (0.79 mL, 7.80 mmol) was added, followed by few drops of DBU (1,8-Diazabicyclo(5.4.0)undec-7-ene). The reaction was stirred at room temperature for 1 h, then concentrated under reduced pressure. The crude was purified by flash chromatography (hexane/ethyl acetate, 85:25 + 0.1% of TEA) to give donor **10** (0.71 g, 94%) as a white solid in a ~ 4:1 α/β ratio of anomers.

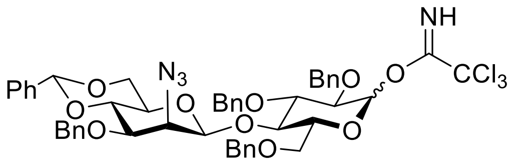

Chemical Formula: $C_{49}H_{49}Cl_3N_4O_{10}$
Exact Mass: 958,25
Molecular Weight: 960,30

R_f α = R_f β = 0.24 (hexane/ethyl acetate 65:35)

α/β = 0.85:0.15

^{1}H NMR ($CDCl_3$) δ 8.73 (s, 0.15H, *H*N=C-CCl_3 β), 8.63 (s, 0.85H, *H*N=C-CCl_3 α), 7.52 – 7.19 (m, 25H, arom.), 6.50 (d, $J_{1\text{-}2}$ = 3.3 Hz, 0.85H, H-1 α), 5.83 (d, $J_{1\text{-}2}$ = 7.2 Hz, 0.15H, H-1 β), 5.53 (s, 1H, C*H*Ph), 5.05 – 4.59 (m, 8H, H-1' α, H-1' β, 3 x OCH_2Ph), 4.48 (d, *J* = 11.9 Hz, 0.15H, OCH*H*Ph β), 4.43 (d, *J* = 11.9 Hz, 0.85H, OCH*H*Ph α), 4.13 – 3.97 (m, 3.70H, H-6a' α, H-6a' β, H-5 α, H-3 α, H-4 α, H-4 β), 3.97 – 3.89 (m, 1H, H-4' α, H-4' β), 3.87 – 3.65 (m, 4H, H-2' α, H-2 α, H-2 β, H-5 β, H-6a α, H-6a β, H-6b α, H-6b β), 3.65 – 3.45 (m, 2H, H-6b' α, H-6b' β, H-3' α, H-3' β), 3.09 – 2.96 (m, 1H, H-5' α, H-5' β).

^{13}C NMR (126 MHz, $CDCl_3$) δ 161.31 (HN=*C*-CCl_3 α), 161.22 (HN=*C*-CCl_3 β), 138.94 – 137.37 (5C, quat.), 129.06 – 125.33 (25C, arom.), 101.54 (*C*HPh), 100.24 (C-1' α), 100.19 (C-1' β), 98.23 (C-1 β), 94.22 (C-1 α), 91.25 (HN=C-*C*Cl_3 α), 90.96 (HN=C-*C*Cl_3 β), 82.86 (C-4 β), 80.41 (C-2 β), 79.60 (C-4 α), 78.92 (C-2 α), 78.44 (C-4'), 76.77 (C-3' β), 76.73 (C-3' α), 76.65 (C-3 β), 76.51 (C-3 α), 75.18 (O*C*H_2Ph α), 75.07 (C-5 β), 74.98 (O*C*H_2Ph β), 73.73 (O*C*H_2Ph α), 73.71 (O*C*H_2Ph β), 73.68 (O*C*H_2Ph), 73.06 (O*C*H_2Ph β), 72.79 (O*C*H_2Ph α), 72.36 (C-5 α), 68.39 (C-6' α), 68.36 (C-6' β), 68.19 (C-6 β), 68.13 (C-6 α), 67.30 (C-5'), 63.59 (C-2' β), 63.57 (C-2' α).

HRMS (ESI+): *m/z* for $C_{49}H_{49}N_4O_{10}NaCl_3$ calcd 981.2412 $[M+Na]^+$, found 981.2409; *m/z* for $C_{49}H_{49}N_2O_{10}NaCl_3$ calcd 953.2350 $[M+Na-N_2]^+$, found 953.2342.

Allyl 2-acetamido-3-*O*-benzyl-4,6-*O*-benzylidene-2-deoxy-β-D-mannopyranosyl-(1→4)-2,3,6-tri-*O*-benzyl-α-D-glucopyranoside (49)

Compound **47** (0.58 g, 0.68 mmol) was converted into acetamide **49** as described in general Procedure D. Automated flash chromatography (Biotage Isolera Prime, SNAP 50g column, hexane/ethyl acetate gradient, 90:10 to 20:80) gave **49** (0.33 g, 56%) as an amorphous glassy solid.

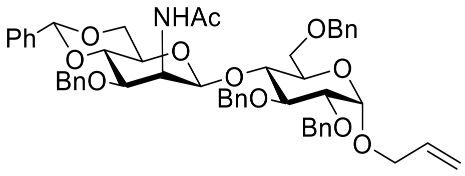

Chemical Formula: $C_{52}H_{57}NO_{11}$
Exact Mass: 871,39
Molecular Weight: 872,02

R_f = 0.38 (hexane/ethyl acetate 1:1)

$[\alpha]_{\mathrm{D}}^{20}$ = -14.49 (*c* 1 in $CHCl_3$)

^{1}H NMR ($CDCl_3$) δ 7.60 – 7.07 (m, 25H, arom), 6.03 – 5.91 (m, 1H, $OCH_2CH{=}CH_2$), 5.58 (d, *J* = 9.0 Hz, 1H, N*H*Ac), 5.50 (s, 1H, C*H*Ph), 5.36 (br dd, 1H, $OCH_2CH{=}CHH$), 5.27 (br dd, 1H, $OCH_2CH{=}CHH$), 5.01 (d, *J* = 11.5 Hz, 1H, OC*H*HPh), 4.87 – 4.79 (m, 2H, OCH*H*Ph, H-1), 4.78 – 4.69 (m, 3H, OC*H*HPh), 4.68 – 4.60 (m, 2H, OCH*H*Ph, H-2'), 4.58 (br s, 1H, H-1'), 4.52 (d, *J* = 12.3 Hz, 1H, OCH*H*Ph), 4.47 (d, *J* = 12.1 Hz, 1H, OCH*H*Ph), 4.24 – 4.08 (m, 2H, $OCHHCH{=}CH_2$, H-6a'), 4.07 – 3.95 (m, 2H, $OCHHCH{=}CH_2$, H-4), 3.88 (t, $J_{3\text{-}2}$ = $J_{3\text{-}4}$ = 9.3 Hz, 1H, H-3), 3.81 – 3.73 (m, 2H, H-6a, H-5), 3.68 – 3.52 (m, 4H, H-6b', H-6b, H-4', H-2), 3.41 (dd, $J_{3'\text{-}4'}$ = 9.6, $J_{3'\text{-}2'}$ = 4.3 Hz, 1H, H-3'), 3.18 – 3.06 (m, 1H, H-5'), 1.89 (s, 3H, $NHCOCH_3$).

^{13}C NMR ($CDCl_3$) δ 170.58 ($NHCOCH_3$), 139.44 – 137.33 (5C, quat.), 133.64 ($OCH_2CH{=}CH_2$), 129.01 – 126.09 (25C, arom.), 118.40 ($OCH_2CH{=}CH_2$), 101.61 (*C*HPh), 100.01 (C-1'), 95.77 (C-1), 80.55 (C-3), 79.51 (C-2), 78.61 (C-4'), 76.53 (C-4), 75.70 (C-3'), 75.08 (OCH_2Ph), 73.52 (OCH_2Ph), 73.23 (OCH_2Ph), 71.26 (OCH_2Ph), 69.67 (C-5), 68.66 ($OCH_2CH{=}CH_2$), 68.44 (C-6'), 68.27 (C-6), 67.02 (C-5'), 50.49 (C-2'), 23.28 ($NHCOCH_3$).

HRMS (ESI+): *m/z* for $C_{52}H_{57}NO_{11}Na$ calcd 894.3829 $[M+Na]^+$, found 894.3817.

2-Acetamido-3-*O*-benzyl-4,6-*O*-benzylidene-2-deoxy-β-D-mannopyranosyl-(1→4)-2,3,6-tri-*O*-benzyl-D-glucopyranose (11)

Compound **49** (0.32 g, 0.36 mmol) was converted in compound **11** as described in general Procedure F. Automated flash chromatography by Biotage Isolera Prime purification system (SNAP 50g column, hexane/ethyl acetate gradient, 25:75 to 100% ethyl acetate) afforded **11** (0.29 g, 94%, amorphous white solid) in a 3:2 α/β mixture.

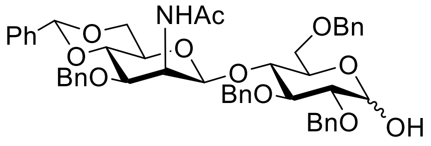

Chemical Formula: $C_{49}H_{53}NO_{11}$
Exact Mass: 831,36
Molecular Weight: 831,96

R_f β = 0.30 and R_f α = 0.22 (hexane/ethyl acetate 25:75)

α/β = 0.60:0.40

^{1}H NMR ($CDCl_3$) δ 7.61 – 7.06 (m, 25H, arom.), 5.58 (br d, *J* = 9.1 Hz, 1H, N*H*Ac), 5.50 (s, 1H, C*H*Ph), 5.24 (br d, *J* = 3.0 Hz, 0.60H, H-1 α), 5.00 – 4.41 (m, 10.40H, H-1 β, H-1', H-2', 4 x OCH_2Ph), 4.18 – 4.08 (m, 1H, H-6a'), 4.07 – 3.96 (m, 1.60H, H-4 α, H-5 α, H-4 β), 3.89 – 3.81 (m, 0.60H, H-3 α), 3.81 – 3.52 (m, 5H, H-2 α, H-3 β, H-4', H-6a, H-6b, H-6b'), 3.44 (m, 1.80H, H-3', H-2 β, H-5 β), 3.23 (s, 0.60H, OH α), 3.19 – 3.08 (m, 1H, H-5'), 1.88 (s, 3H, $NHCOCH_3$).

^{13}C NMR ($CDCl_3$) δ 170.64, 170.60 ($NHCOCH_3$), 139.22 – 137.26 (5C, quat.), 129.04 – 126.10 (25C, arom.), 101.63 (*C*HPh), 99.96 (C-1' α, C-1' β), 97.60 (C-1 β), 91.29 (C-1 α), 83.10 (C-3 β), 82.93 (C-2 β), 80.30 (C-3 α), 79.61 (C-2 α), 78.59 (C-4' α, C-4' β), 76.47 (C-4 β), 76.39 (C-4 α),

75.68 (C-3’ α), 75.65 (C-3’ β), 75.10 (O*C*H_2Ph α), 75.05 (O*C*H_2Ph β), 74.75 (O*C*H_2Ph β), 74.36 (C-5 β), 73.59 (O*C*H_2Ph β), 73.56 (O*C*H_2Ph α), 73.36 (O*C*H_2Ph α, O*C*H_2Ph β), 71.34 (O*C*H_2Ph β), 71.28 (O*C*H_2Ph α), 69.78 (C-5 α), 68.66 (C-6’ α), 68.62 (C-6’ β), 68.49 (C-6 β), 68.34 (C-6 α), 67.08 (C-5’ β), 67.04 (C-5’ α), 50.43 (C-2’ α), 50.40 (C-2’ β), 23.27 (NHCO*C*H_3).

HRMS (ESI+): *m/z* for $C_{49}H_{53}NO_{11}Na$ calcd 854.3516 $[M+Na]^+$, found 854.3510.

2-Acetamido-3-*O*-benzyl-4,6-*O*-benzylidene-2-deoxy-β-D-mannopyranosyl-(1→4)-2,3,6-tri-*O*-benzyl-α-D-glucopyranosyl trichloroacetimidate (12)

To a solution of compound **11** (0.12 g, 0.14 mmol) in dichloromethane (1.5 mL, 0.1 M) trichloroacetonitrile (0.15 mL, 1.44 mmol) was added followed by few drops of DBU (1,8-Diazabicyclo(5.4.0)undec-7-ene). The reaction was stirred at room temperature for 1 h, then concentrated under reduced pressure. The crude was purified by flash chromatography (hexane/ethyl acetate, 60:40 + 1% of TEA) to give donor **12** (0.12 g, 88%, white solid) as a pure α product.

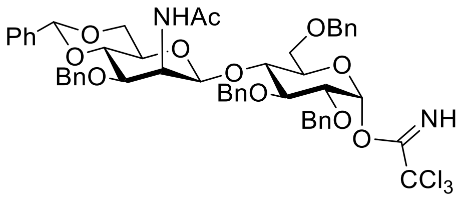

Chemical Formula: $C_{51}H_{53}Cl_3N_2O_{11}$
Exact Mass: 974,27
Molecular Weight: 976,34

R_f = 0.49 (hexane/ethyl acetate 1:1)

$[\alpha]_D^{20}$ = + 6.81 (*c* 1 in $CHCl_3$)

^{1}H NMR ($CDCl_3$) δ 8.64 (s, 1H, N*H*CCl_3), 7.59 – 7.00 (m, 25H, arom.), 6.51 (d, *J* = 3.5 Hz, 1H, H-1), 5.58 (d, *J* = 9.2 Hz, 1H, N*H*Ac), 5.51 (s, 1H, C*H*Ph), 5.00 (d, *J* = 11.6 Hz, 1H, OC*H*HPh), 4.82 (d, *J* = 11.6 Hz, 1H, OCH*H*Ph), 4.76 – 4.62 (m, 6H, H-2’, H-1’, OC*H₂*Ph), 4.55 – 4.45 (m, 2H, 2x OC*H*HPh), 4.18 – 4.06 (m, 2H, H-4, H-6a’), 3.97 – 3.89 (m, 2H, H-5, H-3), 3.82 – 3.72 (m, 2H, H-6a, H-2), 3.71 – 3.63 (m, 2H, H-6b, H-6b’), 3.59 (t, $J_{4',3'}$ = 9.6 Hz, 1H, H-4’), 3.43 (dd, $J_{3',4'}$ = 9.6, $J_{3',2'}$ = 4.3 Hz, 1H, H-3’), 3.18 – 3.08 (m, 1H, H-5’), 1.86 (s, 3H, NHCOC*H_3*).

^{13}C NMR ($CDCl_3$) δ 170.55 (NH*C*OCH_3), 161.26 (NH*C*Cl_3), 139.17 – 137.28 (5C, quat.), 129.04 – 126.10 (25C, arom.), 101.65 (*C*HPh), 100.05 (C-1’), 94.20 (C-1), 80.08 (C-3), 78.95 (C-2), 78.54 (C-4’), 75.74 (C-3’), 75.46 (C-4), 74.98 (O*C*H_2Ph), 73.54 (O*C*H_2Ph), 72.97 (2C, O*C*H_2Ph), 72.51 (C-5), 71.26 (O*C*H_2Ph), 68.63 (C-6’), 67.88 (C-6), 67.17 (C-5’), 50.42 (C-2’), 23.29 (NHCO*C*H_3).

HRMS (ESI+): *m/z* for $C_{51}H_{53}N_2O_{11}NaCl_3$ calcd 997.2613 $[M+Na]^+$, found 997.2610.

5 Synthesis and characterization of disaccharides

N-Benzyl-*N*-(carbobenzyloxy)-3-aminopropyl 2-*O*-Acetyl-3-*O*-benzyl-4,6-*O*-benzylidene-β-D-glucopyranosyl-(1→4)-2,3,6-tri-*O*-benzyl-β-D-glucopyranoside (14)

Allyl 4-OH glucopyranoside acceptor **7** (1.00 g, 1.36 mmol), glucosyl trichloroacetimidate **13** (1.12 g, 2.00 mmol) and freshly dried 4Å molecular sieves (1 g) were suspended in dichloromethane (50 mL), stirred under argon for 20 min and then the mixture was cooled to -20 °C. Triethylsilyl trifluoromethanesulfonate (0.1M solution in dichloromethane, 2.7 mL) was added dropwise and the disappearance of the starting material was followed by TLC (hexane/ethyl acetate 65:35). After 2 h, the reaction was quenched with TEA (0.30 mL), diluted with dichloromethane, filtered over Celite, and the solvent evaporated. The crude product was purified by flash chromatography (hexane/ethyl acetate, 70:30) to give **14** (1.12 g, 74%) as an amorphous white solid.

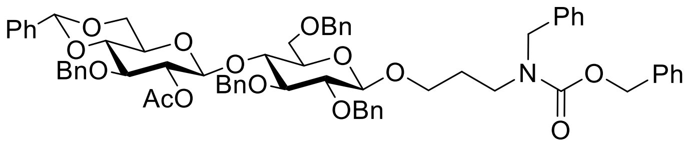

Chemical Formula: $C_{67}H_{71}NO_{14}$
Exact Mass: 1113,49
Molecular Weight: 1114,30

R_f = 0.25 (hexane/ethyl acetate 7:3)

$[\alpha]_D^{20}$ = +1.44 (*c* 1 in $CHCl_3$)

^{1}H NMR ($CDCl_3$): δ (ppm) = 7.56 – 7.10 (m, 35H, arom.), 5.50 (s, 1H, C*H*Ph), 5.19 (br s, 2H, NCOOC*H*$_2$Ph), 4.99 – 4.92 (br t, 1H, H-2’), 4.92 – 4.84 (m, 2H, OC*H*$_2$Ph), 4.84 – 4.39 (m, 9H, NC*H*$_2$Ph, H-1’, OC*H*$_2$Ph), 4.29 (br d, 1H, H-1), 4.14 (dt, $J_{5'\text{-}6a'}$ = 9.7 Hz, $J_{6a'\text{-}6b'}$ = 18.6, 1H, H-6a’), 3.92 (m, 2H, H-4, OC*H*HCH$_2$CH$_2$N), 3.70 (m, 3H, H-4’, H-6a, H-6b), 3.46 (m, 8H, H-2, H-3, OCH*H*CH$_2$CH$_2$N, H-5, H-3’, H-6b’, OCH$_2$CH$_2$C*H*$_2$N), 3.16 (td, $J_{5'\text{-}4'}$ = 4.9 Hz, $J_{5'\text{-}6a'}$ = 9.7, 1H, H-5’), 1.96 (s, 3H, OCOC*H*$_3$), 1.95 – 1.79 (m, 2H, OCH$_2$C*H*$_2$CH$_2$N).

^{13}C NMR ($CDCl_3$): δ(ppm) = 169.11 (NH*C*OCH$_3$), 156.73 and 156.15 (N*C*OOCH$_2$Ph), 139.08 – 136.82 (7C, quat.), 129.04 – 126.05 (35C, arom.), 103.48 (C-1), 101.20 (*C*HPh), 100.80 (C-1’), 82.63 (C-3), 81.66 (2C, C-2 and C-4’), 78.59 (C-3’), 76.94 (C-4), 75.35 (O*C*H$_2$Ph), 74.82 (O*C*H$_2$Ph), 74.76 (C-5), 74.04 (O*C*H$_2$Ph), 73.62 (O*C*H$_2$Ph), 73.40 (C-2’), 68.59 (C-6’), 67.78 (C-6), 67.38 (NCOO*C*H$_2$Ph), 67.22 (O*C*H$_2$CH$_2$CH$_2$N), 65.99 (C-5’), 50.86 and 50.76 (N*C*H$_2$Ph), 44.80 and 43.72 (OCH$_2$CH$_2$*C*H$_2$N), 28.66 and 28.22 (OCH$_2$*C*H$_2$CH$_2$N), 20.88 (OCO*C*H$_3$).

HRMS (ESI+): *m/z* for $C_{67}H_{71}NO_{14}Na$ calcd 1136.4772 $[M+Na]^+$, found 1136.4771.

N-Benzyl-*N*-(carbobenzyloxy)-3-aminopropyl 3-*O*-benzyl-4,6-*O*-benzylidene-β-D-glucopyranosyl-(1→4)-2,3,6-tri-*O*-benzyl-β-D-glucopyranoside (15)

According to general Procedure A compound **14** (1.10 g, 0.99 mmol) was deacetylated affording **15**. Automated flash chromatography by Biotage Isolera Prime purification system (SNAP 50g column, hexane/ethyl acetate gradient, 95:5 to 30:70) gave **15** (0.98 g, 93%) as a white solid.

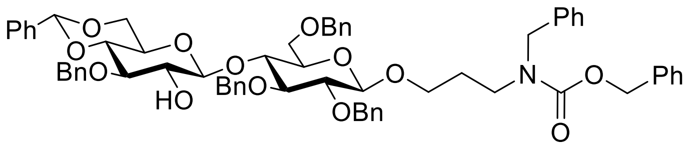

R_f = 0.33 (hexane/ethyl acetate 65:35)

$[\alpha]_D^{20}$ = -2.14 (*c* 1 in $CHCl_3$)

^{1}H NMR ($CDCl_3$) δ 7.65 – 7.06 (m, 35H, arom.), 5.50 (s, 1H, C*H*Ph), 5.21 (br s, 2H, NCOOC*H*$_2$Ph), 5.01 – 4.43 (m, 11H, OC*H*$_2$Ph, NC*H*$_2$Ph, H-1'), 4.34 (br dd, 1H, H-1), 4.07 – 3.88 (m, 4H, H-4, H-6a, H-6a', OC*H*HCH$_2$CH$_2$N), 3.83 – 3.71 (m, 1H, H-6b), 3.67 – 3.20 (m, 11H, H-4', H-3, H-3', H-6b', OCH*H*CH$_2$CH$_2$N, H-2', H-5, OCH$_2$CH$_2$C*H*$_2$N, H-2, O*H*), 3.16 – 3.09 (m, 1H, H-5'), 2.02 – 1.78 (m, 2H, OCH$_2$C*H*$_2$CH$_2$N).

^{13}C NMR ($CDCl_3$) δ 156.69 and 156.15 (N*C*OOCH$_2$Ph), 139.00 – 137.36 (7C, quat.), 128.97 – 126.07 (35C, arom.), 103.69 (C-1', C-1), 101.18 (*C*HPh), 83.39 (C-3), 82.00 (C-2), 81.23 (C-4'), 80.32 (C-3'), 77.57 (C-4), 75.33 (C-2'), 75.15 (O*C*H$_2$Ph), 74.87 (O*C*H$_2$Ph), 74.53 (O*C*H$_2$Ph), 74.36 (C-5), 73.65 (O*C*H$_2$Ph), 68.62 (C-6'), 68.51 (C-6), 67.44 (O*C*H$_2$CH$_2$CH$_2$N), 67.25 (NCOO*C*H$_2$Ph), 66.35 (C-5'), 50.87 and 50.76 (N*C*H$_2$Ph), 44.77 and 43.73 (OCH$_2$CH$_2$*C*H$_2$N), 28.72 and 28.26 (OCH$_2$*C*H$_2$CH$_2$N).

HRMS (ESI+): *m/z* for $C_{65}H_{69}NO_{13}Na$ calcd 1094.4667 $[M+Na]^+$, found 1094.4655.

***N*-Benzyl-*N*-(carbobenzyloxy)-3-aminopropyl 3-*O*-benzyl-4,6-*O*-benzylidene-2-*O*-(*N*-imidazole-1-sulfonyl)-β-D-glucopyranosyl-(1→4)-2,3,6-tri-*O*-benzyl-β-D-glucopyranoside (16)**

As described in general Procedure B, compound **15** (0.95 g, 0.88 mmol) was activated with sulfonyldiimidazole obtaining **16**. Automated flash chromatography by Biotage Isolera Prime purification system (SNAP 50g column, hexane/ethyl acetate gradient, 95:5 to 30:70) gave **16** (0.79 g, 75%) as a white solid.

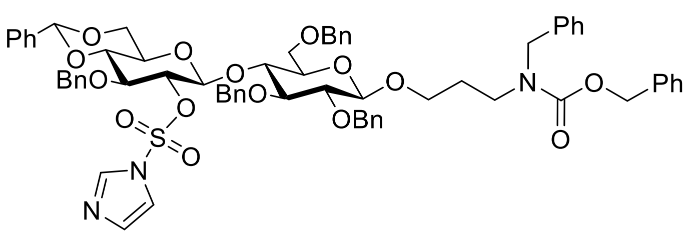

R_f = 0.33 (hexane/ethyl acetate 6:4)

$[\alpha]_D^{20}$ = -14.19 (*c* 1 in $CHCl_3$)

^{1}H NMR ($CDCl_3$) δ 7.87 (s, 1H, Imid.), 7.52 – 7.14 (m, 36H, 35x arom., 1x Imid.), 7.04 (s, 1H, Imid.), 5.49 (s, 1H, C*H*Ph), 5.21 (br s, 2H, NCOOC*H₂*Ph), 4.86 – 4.42 (m, 11H, 7x OC*H*HPh, NC*H₂*Ph, H-2', H-1'), 4.36 – 4.18 (m, 3H, OC*H*HPh, H-6a', H-1), 4.03 – 3.85 (m, 2H, H-4, OC*H*HCH₂CH₂N), 3.66 – 3.28 (m, 10H, H-4', OCH*H*CH₂CH₂N, H-3', H-6b', H-6a, H-6b, OCH₂CH₂C*H₂*N, H-3, H-2), 3.08 – 2.97 (m, 2H, H-5, H-5'), 2.03 – 1.76 (m, 2H, OCH₂C*H₂*CH₂N).

^{13}C NMR ($CDCl_3$) δ 156.71 and 156.16 (N*C*OOCH₂Ph), 138.86 – 137.17 (6C, quat.), 137.00 (*C*H, Imid.), 136.87 (quat.), 130.37 (*C*H, Imid.), 129.24 – 125.31 (35C, arom.), 118.49 (*C*H, Imid.), 103.56 (C-1), 101.43 (*C*HPh), 98.33 (C-1'), 85.43 (C-2'), 81.92 (C-4', C-3), 81.78 (C-2), 77.03 (C-3'), 75.48 (C-4), 75.40 (O*C*H₂Ph), 74.85 (O*C*H₂Ph), 74.42 (O*C*H₂Ph), 73.74 (C-5), 73.57 (O*C*H₂Ph), 68.42 (C-6'), 67.46 (C-6, O*C*H₂CH₂CH₂N), 67.23 (NCOO*C*H₂Ph), 65.83 (C-5'), 50.89 and 50.80 (N*C*H₂Ph), 44.83 and 43.75 (OCH₂CH₂*C*H₂N), 28.68 and 28.22 (OCH₂*C*H₂CH₂N).

HRMS (ESI+): *m/z* for $C_{68}H_{71}N_3O_{15}NaS$ calcd 1224.4504 $[M+Na]^+$, found 1224.4479.

N-Benzyl-N-(carbobenzyloxy)-3-aminopropyl 2-Azido-3-O-benzyl-4,6-O-benzylidene-2-deoxy-β-D-mannopyranosyl-(1→4)-2,3,6-tri-O-benzyl-β-D-glucopyranoside (17)

Compound **16** (0.77 g, 0.64 mmol) was transformed into **17** as described in general Procedure C. Flash chromatography purification (hexane/ethyl acetate gradient, 80:20 to 70:30) afforded **17** (0.63 g, 90%) as a white solid.

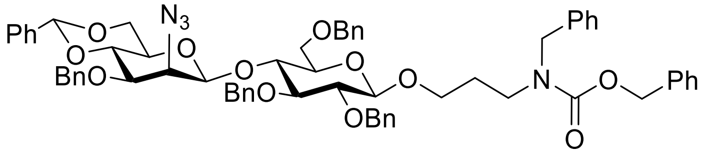

Chemical Formula: $C_{65}H_{68}N_4O_{12}$
Exact Mass: 1096,48
Molecular Weight: 1097,28

R_f = 0.33 (hexane/ethyl acetate 7:3)

$[\alpha]_D^{20}$ = -22.30 (*c* 1 in $CHCl_3$)

^{1}H NMR ($CDCl_3$) δ 7.62 – 7.01 (m, 35H, arom.), 5.52 (s, 1H, C*H*Ph), 5.19 (br s, 2H, NCOOC*H₂*Ph), 5.01 – 4.39 (m, 11H, 7x OC*H*HPh, 2x NC*H₂*Ph, H-1'), 4.39 – 4.26 (br dd, 1H, H-1), 4.06 – 3.87 (m, 4H, H-6a', OC*H*HCH₂CH₂N, H-4, H-4'), 3.82 (br s, 1H, H-2'), 3.80 – 3.61 (m, 2H, H-6a, H-6b), 3.66 – 3.31 (m, 8H, H-6b', OCH*H*CH₂CH₂N, H-3, H-3', H-5, H-2, OCH₂CH₂C*H₂*N), 3.10 – 2.95 (m, 1H, H-5'), 2.02 – 1.78 (m, 2H, OCH₂C*H₂*CH₂N).

^{13}C NMR ($CDCl_3$) δ 156.75 and 156.18 (N*C*OOCH₂Ph), 138.95 – 137.36 (7C, quat.), 128.97 – 126.06 (35C, arom.), 103.62 (C-1), 101.51 (*C*HPh), 100.28 (C-1'), 82.93 (C-3), 81.83 (C-2), 78.42 (C-4'), 77.54 (C-4), 76.63 (C-3'), 75.33 (O*C*H₂Ph), 74.86 (O*C*H₂Ph), 74.10 (C-5), 73.64 (O*C*H₂Ph), 72.75 (O*C*H₂Ph), 68.66 (C-6), 68.36 (C-6'), 67.23 (O*C*H₂CH₂CH₂N, C-5'), 63.61 (C-2'), 50.86 and 50.78 (N*C*H₂Ph), 44.78 and 43.68 (OCH₂CH₂*C*H₂N), 28.68 and 28.23 (OCH₂*C*H₂CH₂N).

HRMS (ESI+): *m/z* for $C_{65}H_{68}N_4O_{12}Na$ calcd 1119.4731 $[M+Na]^+$, found 1119.4716.

N-Benzyl-N-(carbobenzyloxy)-3-aminopropyl 2-Acetamido-3-O-benzyl-4,6-O-benzylidene-2-deoxy-β-D-mannopyranosyl-(1→4)-2,3,6-tri-O-benzyl-β-D-glucopyranoside (18)

Compound **18** was prepared from compound **17** (0.61 g, 0.55 mmol) as described in general Procedure D. Flash chromatography purification (hexane/ethyl acetate, 40:60) gave **18** (0.40 g, 66%) as a white solid.

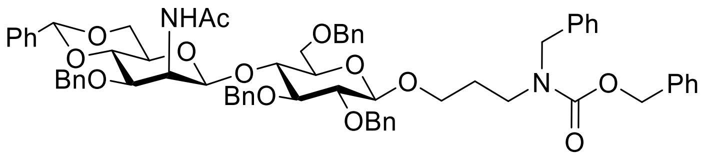

Chemical Formula: $C_{67}H_{72}N_2O_{13}$
Exact Mass: 1112,50
Molecular Weight: 1113,31

R_f = 0.22 (hexane/ethyl acetate 4:6)

$[\alpha]_D^{20}$ = -33.51 (*c* 1 in $CHCl_3$)

^{1}H NMR ($CDCl_3$) δ 7.55 – 7.12 (m, 35H), 5.60 (br d, 1H, N*H*Ac), 5.51 (s, 1H, C*H*Ph), 5.20 (br s, 2H, NCOOCH_2Ph), 4.92 (br d, 1H, OC*H*HPh), 4.87 – 4.41 (m, 12H, 4x OCH_2Ph, NCH_2Ph, H-1', H-2'), 4.39 – 4.24 (m, 1H, H-1), 4.13 (br dd, 1H, H-6a), 4.08 – 3.86 (m, 2H, H-4, OC*H*HCH_2CH_2N), 3.78 (br d, 1H, H-6a'), 3.74 – 3.27 (m, 10H, H-6b', H-6b, H-4', OCH*H*CH_2CH_2N, H-3, H-3', OCH_2CH_2CH_2N, H-2, H-5), 3.19 – 3.09 (m, 1H, H-5'), 2.02 – 1.78 (m, 5H, OCH_2CH_2CH_2N, NHCOCH_3).

^{13}C NMR ($CDCl_3$) δ 170.51 (NH*C*OCH$_3$), 156.71 and 156.18 (N*C*OOCH$_2$Ph), 139.13 – 136.72 (7C, quat.), 129.01 – 126.10 (35C, arom.), 103.67 (C-1), 101.63 (*C*HPh), 99.99 (C-1'), 83.20 (C-3), 81.95 (C-2), 78.69 (C-4'), 76.52 (C-4), 75.77 (C-3'), 75.06 (O*C*H$_2$Ph), 74.76 (O*C*H$_2$Ph), 74.30 (C-5), 73.49 (O*C*H$_2$Ph), 71.41 (O*C*H$_2$Ph), 68.68 (C-6'), 68.46 (C-6), 67.42 (O*C*H$_2$CH$_2$CH$_2$N), 67.24 (NCOO*C*H$_2$Ph), 67.04 (C-5'), 50.87 and 50.80 (N*C*H$_2$Ph), 50.45 (C-2'), 44.80 and 43.75 (OCH$_2$CH$_2$*C*H$_2$N), 28.69 and 28.26 (OCH$_2$*C*H$_2$CH$_2$N), 23.25 (NHCO*C*H$_3$).

HRMS (ESI+): *m/z* for $C_{67}H_{72}N_2O_{13}Na$ calcd 1135.4932 $[M+Na]^+$, found 1135.4937.

3-aminopropyl 2-Acetamido-2-deoxy-β-D-mannopyranosyl-(1→4)-β-D-glucopyranoside (1)

Compound **18** (60 mg, 0.054 mmol) was hydrogenolyzed over $Pd(OH)_2/C$ according to general Procedure G, affording lyophilized hydrochloride **1** as a white foam (25 mg, 99% yield).

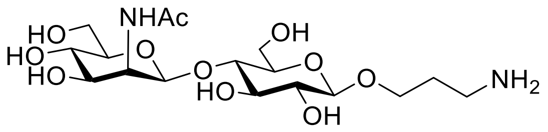

Chemical Formula: $C_{17}H_{32}N_2O_{11}$
Exact Mass: 440,20
Molecular Weight: 440,45

R_f = 0.20 (ethyl acetate/isopropanol/water 3:3:2)

$[\alpha]_D^{20}$ = -27.43 (*c* 1 in H_2O)

^{1}H NMR (D_2O) δ 4.80 (d, $J_{1'-2'}$ = 1.2 Hz, 1H, H-1'), 4.46 (br d, 1H, H-2'), 4.40 (d, J_{1-2} = 8.0 Hz, 1H, H-1), 4.00 – 3.91 (m, 1H, OC*H*HCH2CH2N), 3.87 – 3.68 (m, 5H, H-6a', H-6a, H-3', H-6b', OC*H*HCH2CH2N), 3.63 (dd, J_{6b-6a} = 12.3, J_{6b-5} = 5.1 Hz, 1H, H-6b), 3.60 – 3.53 (m, 2H, H-5, H-3), 3.49 – 3.40 (m, 2H, H-4, H-4'), 3.37 (m, 1H, H-5'), 3.27 – 3.19 (m, 1H, H-2), 3.07 (m, 2H, OCH2CH2C*H2*N), 1.99 (s, *J* = 9.9 Hz, 3H, NHCOC*H3*), 1.97 – 1.86 (m, 2H, OCH2C*H2*CH2N).

^{13}C NMR (D_2O) δ 175.43 (NH*C*OCH3), 102.10 (C-1), 99.31 (C-1'), 78.66 (C-5), 76.50 (C-5'), 74.48 (C-4), 74.18 (C-3), 72.85 (C-2), 71.93 (C-3'), 67.86 (O*C*H2CH2CH2N), 66.61 (C-4'), 60.38 (C-6'), 60.09 (C-6), 53.22 (C-2'), 37.57 (OCH2CH2*C*H2N), 26.71 (OCH2*C*H2CH2N), 22.01 (NHCO*C*H3).

HRMS (ESI+): *m/z* for $C_{17}H_{32}N_2O_{11}Na$ calcd 463.1904 [M+Na]$^+$, found 463.1903; *m/z* for $C_{17}H_{33}N_2O_{11}$ calcd 441.2084 [M+H]$^+$, found 441.2082.

N-Benzyl-N-(carbobenzyloxy)-3-aminopropyl 2-Acetamido-3,6-di-O-benzyl-2-deoxy-β-D-mannopyranosyl-(1→4)-2,3,6-tri-O-benzyl-β-D-glucopyranoside (19)

Compound **18** (0.12 g, 0.11 mmol) and 4Å MS (120 mg) in dichloromethane (3.5 mL, 0.03M) were stirred for 10 min at room temperature, then triethylsilane (0.17 mL, 1.07 mmol) was added and the mixture was stirred for additional 30 min. Subsequently, $BF_3·Et_2O$ (150 µL, 0.54 mmol) was added dropwise to the reaction mixture. After 3 h, the reaction was quenched with 0.1 mL of TEA, diluted with dichloromethane, filtered over a Celite pad and concentrated *in vacuo*. The residue was purified by flash chromatography (hexane/ethyl acetate, 40:60) to afford **19** (0.095 g, 80%) as a colourless oil.

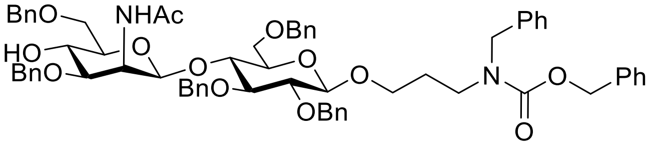

Chemical Formula: $C_{67}H_{74}N_2O_{13}$
Exact Mass: 1114,52
Molecular Weight: 1115,33

R_f = 0.27 (hexane/ethyl acetate 4:6)

$[\alpha]_D^{20}$ = -39.08 (*c* 1 in $CHCl_3$)

^{1}H NMR ($CDCl_3$) δ 7.47 – 7.04 (m, 35H, arom.), 5.65 (d, *J* = 9.7 Hz, 1H, N*H*Ac), 5.20 (br s, 2H, NCOOC*H2*Ph), 4.94 – 4.85 (m, 2H, OC*H2*Ph), 4.85 – 4.72 (m, 2H, 2x OC*H*HPh), 4.72 – 4.41 (m, 9H, OC*H2*Ph, H-1', H-2', NC*H2*Ph), 4.38 – 4.22 (m, 2H), 4.07 – 3.86 (m, 2H, H-4, OC*H*HCH2CH2N), 3.80 – 3.68 (m, 2H, H-6a, H-6b), 3.65 (t, *J* = 9.5 Hz, 1H, H-4'), 3.62 – 3.29 (m, 8H, H-3, H-6a', H-6b', OCH*H*CH2CH2N, H-2, H-5, OCH2CH2C*H2*N), 3.22 – 3.12 (m, 2H, H-3', H-5'), 2.50 (br s, 1H, OH), 2.01 – 1.82 (m, 2H, OCH2C*H2*CH2N), 1.81 (s, 3H, NHCOC*H3*).

^{13}C NMR ($CDCl_3$) δ 170.51 (NH*C*OCH3), 156.70 and 156.16 (N*C*OOCH2Ph), 139.30 – 137.65 (7C, quat.), 128.56 – 126.72 (35C, arom.), 103.61 (C-1), 99.73 (C-1'), 83.23 (C-3), 81.96 (C-2), 80.00

(C-3'), 76.41 (C-4), 74.92 (2C, C-5', O*C*H_2Ph), 74.74 (O*C*H_2Ph), 74.22 (C-5), 73.53 (O*C*H_2Ph), 73.29 (O*C*H_2Ph), 71.03 (O*C*H_2Ph), 69.27 (C-6'), 68.74 (C-6), 67.42 (2C, C-4', NCOO*C*H_2Ph), 67.23 (O*C*$H_2CH_2CH_2$N), 50.86 and 50.74 (N*C*H_2Ph), 49.20 (C-2'), 44.80 and 43.76 (OCH_2CH_2*C*H_2N), 28.69 and 28.24 (OCH_2*C*H_2CH_2N), 23.23 (NHCO*C*H_3).

HRMS (ESI+): *m/z* for $C_{67}H_{74}N_2O_{13}Na$ calcd 1137.5089 $[M+Na]^+$, found 1137.5084.

***N*-Benzyl-*N*-(carbobenzyloxy)-3-aminopropyl-{2'-Acetamido-3',6'-di-*O*-benzyl-4'-*O*-[bis(benzyloxy)phosphoryl]-2'-deoxy-β-D-mannopyranosyl}-(1→4)-2,3,6-tri-*O*-benzyl-β-D-glucopyranoside (20)**

To a solution of compound **19** (60 mg, 0.054 mmol) in dichloromethane (1.5 mL) 1H-tetrazole (0.45M in acetonitrile, 0.27 mL, 0.12 mmol) and dibenzyl-*N*,*N*-diisopropylphosphoramidite (30 µL, 0.080 mmol) were added at room temperature.[25] The reaction mixture was stirred at room temperature for 1 h, then cooled to -20°C, and a 0.66 M solution of *m*-chloroperbenzoic acid (0.14 mL, 0.10 mmol) in dichloromethane was added dropwise. After 2 h, the reaction was gradually warmed to room temperature and then diluted with 5mL of dichloromethane, and washed with satd $NaHCO_3$ (2 x 10 mL) and brine (10 mL). The aqueous phases were extracted with dichloromethane (20 mL) and the resulting organic layer was washed with brine (20 mL). Then, the combined organic phases were dried over sodium sulfate, filtered and evaporated. The residue was purified by flash chromatography (hexane/ethyl acetate, 35:65) to furnish **20** (59 mg, 80%) as white solid.

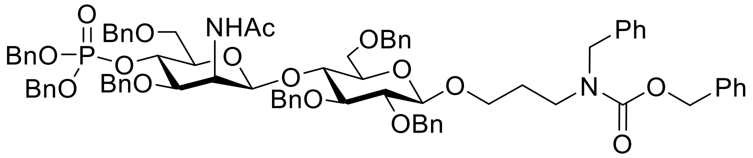

Chemical Formula: $C_{81}H_{87}N_2O_{16}P$
Exact Mass: 1374,58
Molecular Weight: 1375,56

R_f = 0.22 (hexane/ethyl acetate 4:6)

$[\alpha]_D^{20}$ = -18.44 (*c* 1 in $CHCl_3$)

^{1}H NMR ($CDCl_3$) δ 7.54 – 6.88 (m, 45H, arom.), 5.77 (d, *J* = 9.8 Hz, 1H, N*H*Ac), 5.19 (br s, 2H, NCOOC*H*$_2$Ph), 4.98 – 4.40 (m, 17H, OC*H*$_2$Ph, H-1', H-2', H-4', NC*H*$_2$Ph), 4.38 – 4.21 (m, 3H, 2x OCH*H*Ph, H-1), 4.03 – 3.85 (m, 2H, H-4, OC*H*HCH_2CH_2N), 3.73 (t, *J* = 14.8 Hz, 2H, H-6a, H-6b), 3.61 – 3.32 (m, 9H, H-6a', H-6b', H-3, OCH*H*CH_2CH_2N, H-3', H-2, OCH_2CH_2C*H*$_2$N, H-5), 3.32 – 3.24 (m, 1H, H-5'), 2.00 – 1.80 (m, 2H, OCH_2C*H*$_2CH_2$N), 1.77 (s, 3H, NHCOC*H*$_3$).

^{31}P NMR (202 MHz, $CDCl_3$) δ -2.01.

^{13}C NMR ($CDCl_3$) δ 170.51 (NH*C*OCH_3), 139.19 – 137.47 (9C, quat.), 136.98 (d, $J_{C\text{-}P}$ = 7.3 Hz, 1C, quat., $PO(OBn)_2$), 135.89 (d, $J_{C\text{-}P}$ = 7.3 Hz, 1C, quat., $PO(OBn)_2$), 128.54 – 126.89 (45C, arom.), 103.61 (C-1), 99.40 (C-1'), 83.12 (C-3), 81.99 (C-2), 78.08 (d, $J_{3'\text{-}P}$ = 2.8 Hz, 1C, C-3'), 76.63 (C-4), 74.89 (O*C*H_2Ph), 74.77 (d, $J_{5'\text{-}P}$ = 5.6 Hz, 1C, C-5'), 74.72 (O*C*H_2Ph), 74.21 (C-5), 73.34 (O*C*H_2Ph), 73.30 (O*C*H_2Ph), 73.12 (d, $J_{4'\text{-}P}$ = 6.4 Hz, 1C, C-4'), 70.88 (O*C*H_2Ph), 69.32(d, $J_{C\text{-}P}$ =

5.5 Hz, 1C, PO(O*C*H_2Ph)$_2$), 69.20 (d, $J_{C\text{-}P}$ = 5.0 Hz, 1C, PO(O*C*H_2Ph)$_2$), 68.69 (C-6), 68.28 (C-6'), 67.38 (NCOO*C*H_2Ph), 67.22 (O*C*H_2CH$_2$CH$_2$N), 50.86 and 50.80 (N*C*H_2Ph), 49.19 (C-2'), 44.80 and 43.79 (OCH$_2$CH$_2$*C*H$_2$N), 28.71 and 28.25 (OCH$_2$*C*H$_2$CH$_2$N), 23.15 (NHCO*C*H$_3$).

HRMS (ESI+): *m/z* for $C_{81}H_{87}N_2O_{16}NaP$ calcd 1397.5691 [M+Na]$^+$, found 1397.5704.

3-aminopropyl (2'-Acetamido-2'-deoxy-β-D-mannopyranosyl)-(1→4)-β-D-glucopyranoside 4'-phosphate, bis-sodium salt (2)

Compound **20** (20 mg, 0.0145 mmol) was hydrogenolyzed over Pd/C according to general Procedure G. After 4 days (NMR analysis indicated complete conversion), the reaction mixture was filtered over a Celite pad, and the filtrate was concentrated to 1 mL. The water solution of the deprotected compound was eluted over a column filled with Dowex® 50WX8 resin (H$^+$ form), followed by a second ion exchange column on the same resin in Na$^+$ form. Lyophilization of the eluates provided disaccharide phosphate **2** as the sodium salt (6 mg, 80% yield, white solid).

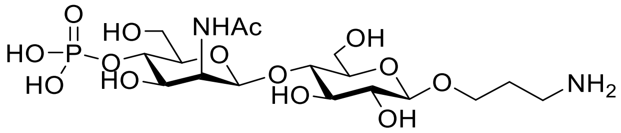

Chemical Formula: $C_{17}H_{33}N_2O_{14}P$
Exact Mass: 520,17
Molecular Weight: 520,42

R_f = 0.10 (ethyl acetate/isopropanol/water 1:1:1 + 1% TEA)

$[\alpha]_D^{20}$ = -8.65 (*c* 1 in CH_3OH)

^{1}H NMR (D_2O) δ 4.79 (br s, 1H, H-1'), 4.48 (br d, 1H, H-2'), 4.41 (d, $J_{1\text{-}2}$ = 8.0 Hz, 1H, H-1), 4.06 – 3.35 (m, 12H, OC*H*HCH$_2$CH$_2$N, H-5', H-3', H-6a, H-6a', H-6b', OCH*H*CH$_2$CH$_2$N, H-6b, H-3, H-4, H-5, H-4'), 3.29 – 3.17 (m, 1H, H-2), 3.17 – 3.00 (m, 2H, OCH$_2$CH$_2$C*H*$_2$N), 2.06 – 1.87 (m, 5H, NHCOC*H*$_3$, OCH$_2$C*H*$_2$CH$_2$N).

^{31}P NMR (D_2O) δ +4.73.

^{13}C NMR (D_2O) δ 175.40 (NH*C*OCH$_3$), 102.21 (C-1), 99.70 (C-1'), 78.95 (C-4), 76.28 (d, $J_{4'\text{-}P}$ = 7.0 Hz, 1C, C-4'), 74.65 (C-5), 74.28 (C-3), 72.95 (C-2), 72.16 (C-3'), 69.61(d, $J_{5\text{-}P}$ = 4.6 Hz, 1C, C-5'), 67.97 (O*C*H$_2$CH$_2$CH$_2$N), 60.62 (C-6'), 60.26 (C-6), 52.47 (C-2'), 37.70 (OCH$_2$CH$_2$*C*H$_2$N), 26.78 (OCH$_2$*C*H$_2$CH$_2$N), 22.12 (NHCO*C*H$_3$).

HRMS (ESI-): *m/z* for $C_{17}H_{32}N_2O_{14}P$ calcd 519.1591 [M-H]$^-$, found 519.1594.

6 Synthesis and characterization of tetrasaccharides

***N*-Benzyl-*N*-(carbobenzyloxy)-3-aminopropyl 2-Acetamido-3-*O*-benzyl-4,6-*O*-benzylidene-2-deoxy-β-D-mannopyranosyl-(1→4)-2,3,6-tri-*O*-benzyl-α-D-glucopyranosyl-(1→4)-2-**

acetamido-3-*O*-benzyl-4,6-*O*-benzylidene-2-deoxy-β-D-mannopyranosyl-(1→4)-2,3,6-tri-*O*-benzyl-β-D-glucopyranoside (21)

Disaccharide acceptor **19** (30 mg, 0.028 mmol), disaccharide donor **12** (75 mg, 0.076 mmol) and freshly dried 4Å molecular sieves (50 mg) were suspended in dichloromethane (2 mL), stirred under argon for 20 min and then the mixture was cooled to 0 °C. A 0.1M solution of trimethylsilyl trifluoromethanesulfonate in dichloromethane (60 μL, 0.0056 mmol) was added dropwise and the disappearance of the starting material was followed by TLC analysis (hexane/ethyl acetate, 65:35). After 24 h, the reaction was quenched with TEA (60 μL), diluted with dichloromethane, filtered over Celite, and the solvent evaporated. The crude product was purified by flash chromatography (hexane/ethyl acetate, 45:55) to give **21** (29 mg, 55%) as a colourless oil. Acceptor **19** (10 mg) was recovered as unreacted substrate (83% conversion yield).

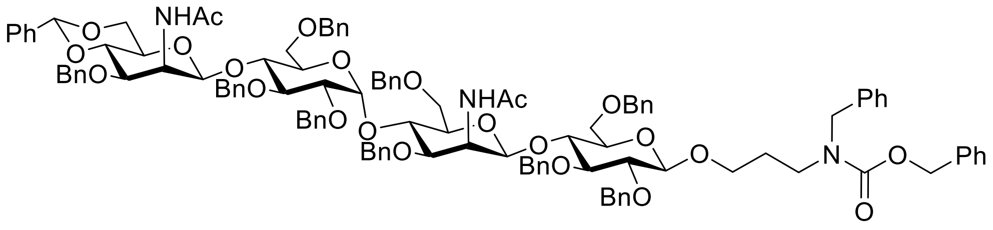

Chemical Formula: $C_{116}H_{125}N_3O_{23}$
Exact Mass: 1927,87
Molecular Weight: 1929,27

R_f = 0.33 (hexane/ethyl acetate 4:6)

$[\alpha]_D^{20}$ = -4.94 (*c* 0.5 in $CHCl_3$)

^{1}H NMR ($CDCl_3$) δ 7.56 – 7.03 (m, 60H, arom.), 5.82 (d, *J* = 9.9 Hz, 1H, N*H*Ac), 5.75 (d, *J* = 3.7 Hz, 1H, H-1’’), 5.52 – 5.45 (m, 2H, C*H*Ph, N*H*Ac), 5.20 (br s, 2H, NCOOCH_2Ph), 5.00 – 4.39 (m, 26H, H-1’, H-2’, H-2’’’, NCH_2Ph, H-1’’’, OCH_2Ph), 4.32 (br dd, *J* = 29.5, 7.0 Hz, 1H, H-1), 4.18 (d, *J* = 12.1 Hz, 1H, OCH*H*Ph), 4.12 – 3.99 (m, 4H, H-4’, H-6a’’’, OCH*H*Ph, H-4), 3.99 – 3.89 (m, 2H, OC*H*HCH_2CH_2N, H-4’’), 3.84 – 3.77 (br dd, 1H, H6a), 3.76 – 3.66 (m, 2H, H-3’’, H-6b), 3.66 – 3.56 (m, 5H, H-6a’, H-3’, H-3, H-5’’, H-6b’’’), 3.56 – 3.44 (m, 5H, OC*H*HCH_2CH_2N, H-4’’’, H-6b’, H-6a’’, H-2’’), 3.44 – 3.33 (m, 6H, H-2, H-5, OCH_2CH_2C*H*$_2$N, H-6b’’, H-5’), 3.31 (dd, $J_{3'''-4'''}$ = 9.8, $J_{3'''-2'''}$ = 4.3 Hz, 1H, H-3’’’), 3.04 (td, $J_{5'''-4'''}$ = 9.8, $J_{5'''-6a'''}$ = 5.0 Hz, 1H, H-5’’’), 1.90 (s, 3H, NHCOC*H*$_3$), 2.01 – 1.78 (m, 2H, OCH_2C*H*$_2$CH_2N), 1.81 (s, 3H, NHCOC*H*$_3$).

^{13}C NMR ($CDCl_3$) δ 170.38, 170.35 (2C, NH*C*OCH_3), 156.72 (N*C*OOCH_2Ph), 139.39 – 137.33 (12C, quat.), 129.02 – 126.09 (60C, arom.), 103.61 (C-1), 101.65 (*C*HPh), 100.19 (C-1’), 99.73 (C-1’’’), 96.27 (C-1’’), 83.20 (C-3), 81.96 (C-2), 81.34 (C-3’), 80.06 (C-3’’), 78.91 (C-2’’), 78.53 (C-4’’’), 77.21 (C-4), 75.86 (C-4’’), 75.76 (C-3’’’), 75.01 (C-5’), 74.85 (O*C*H_2Ph), 74.78 (O*C*H_2Ph), 74.71 (O*C*H_2Ph), 74.18 (C-5), 73.46 (O*C*H_2Ph), 73.17 (2C, O*C*H_2Ph), 72.55 (O*C*H_2Ph), 71.17 (O*C*H_2Ph), 70.50 (O*C*H_2Ph), 70.28 (C-5’’), 68.97 (C-4’), 68.73 (C-6’), 68.60 (2C: C-6’’’, C-6), 67.89 (C-6’’), 67.39 (O*C*H_2CH_2CH_2N), 67.23 (NCOO*C*H_2Ph), 67.04 (C-5’’’), 50.86 and 50.76 (N*C*H_2Ph), 50.52 (C-2’’’), 49.26 (C-2’), 44.80 and 43.78 (OCH_2CH_2*C*H_2N), 28.70 and 28.26 (OCH_2*C*H_2CH_2N), 23.41 (NHCO*C*H_3), 23.23 (NHCO*C*H_3).

HRMS (ESI+): *m/z* for $C_{116}H_{125}N_3O_{23}Na$ calcd 1950.8602 $[M+Na]^+$, found 1950.8584 $[M+Na]^+$, 1951.8619 $[M+1Na]^+$, 987.4244 $[M+2+2Na]^{++}$.

3-aminopropyl 2-Acetamido-2-deoxy-β-D-mannopyranosyl-(1→4)-α-D-glucopyranosyl-(1→4)-2-acetamido-2-deoxy-β-D-mannopyranosyl-(1→4)-β-D-glucopyranoside (6)

Compound **21** (28 mg, 0.0145 mmol) was hydrogenolyzed over $Pd(OH)_2/C$ according to general Procedure G, affording tetrasaccharide **6**·HCl as a white foam (11 mg, 95% yield).

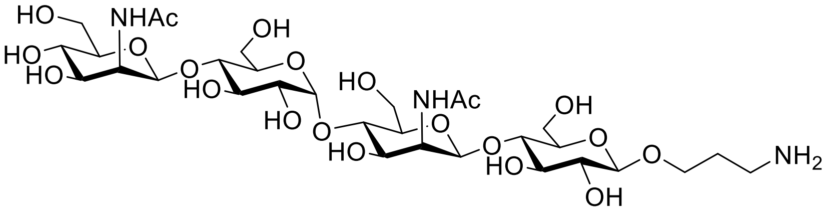

Chemical Formula: $C_{31}H_{55}N_3O_{21}$
Exact Mass: 805,33
Molecular Weight: 805,78

R_f = 0.20 (ethyl acetate/isopropanol/water 1:1:1)

^{1}H NMR (D_2O) δ 5.25 (br d, 1H, H-1’’), 4.82 – 4.76 (m, 2H, H-1’, H-1’’’), 4.48 – 4.41 (m, 2H, H-2’, H-2’’’), 4.38 (br d, 1H, H-1), 4.03 – 3.89 (m, 2H, H-3’ or H-3’’’, OC*H*HCH₂CH₂N), 3.87 – 3.30 (m, 21H, H-3, H-4, H-5, H-6a, H-6b, H-4’, H-5’, H-6a’, H-6b’, H-2’’, H-3’’, H-4’’, H-5’’, H-6a’’, H-6b’’, H-3’’’ or H-3’, H-4’’’, H-5’’’, H-6a’’’, H-6b’’’, OCH*H*CH_2CH_2N), 3.28 – 3.15 (m, 1H, H-2), 3.14 – 3.01 (m, 8H, N(C*H₂*$CH_3)_3$, OCH_2CH_2C*H₂*N), 1.98 (s, 3H, NHCOC*H₃*), 2.07 – 1.80 (m, 2H, OCH_2C*H₂*CH_2N), 1.89 (s, 3H, NHCOC*H₃*), 1.19 (t, 9H, $N(CH_2C$*H₃*$)_3$).

^{13}C NMR (D_2O) δ 175.44 (2C, NH*C*OCH₃), 102.13 (C-1), 99.30 and 99.27 (2C, C-1’, C-1’’’), 99.24 (C-1’’), 78.70 (C-3), 78.48 (C-4’’), 76.52 (C-5), 74.98, 74.49, 74.19 (C-4), 73.83, 72.86 (C-2), 72.52, 71.96 and 71.35 (2C, C-2’’, C-3’’), 71.05, 67.85 (O*C*$H_2CH_2CH_2N$), 66.61 (C-5’’’), 60.41 and 60.37 and 60.09 and 59.96 (4C, C-6, C-6’, C-6’’ and C-6’’’), 53.27 (2C, C-2’and C-2’’’), 46.70 (3C, N(*C*$H_2CH_3)_3$), 37.56 (OCH_2CH_2*C*H_2N), 26.68 (OCH_2*C*H_2CH_2N), 22.00 (2C, NHCO*C*H₃), 8.24 (3C, $N(CH_2$*C*$H_3)_3$).

HRMS (ESI+): *m/z* for $C_{31}H_{56}N_3O_{21}$ calcd 806.3406 $[M+H]^+$, found 806.3382; *m/z* for $C_{31}H_{55}N_3O_{21}Na$ calcd 828.3226 $[M+Na]^+$, found 828.3206.

7 Synthesis and characterization of trisaccharides

***N*-(carbobenzyloxy)-3-aminopropyl (2-Azido-3-*O*-benzyl-4,6-*O*-benzylidene-2-deoxy-β-D-mannopyranosyl)-(1→4)-(2,3,6-tri-*O*-benzyl-α-D-glucopyranosyl)-(1→4)-(2-azido-3,6-di-*O*-benzyl-2-deoxy-β-D-mannopyranoside (22a) and**

***N*-(carbobenzyloxy)-3-aminopropyl (2-Azido-3-*O*-benzyl-4,6-*O*-benzylidene-2-deoxy-β-D-mannopyranosyl)-(1→4)-(2,3,6-tri-*O*-benzyl-β-D-glucopyranosyl)-(1→4)-(2-azido-3,6-di-*O*-benzyl-2-deoxy-β-D-mannopyranoside (22b)**

Acceptor **8** (0.25 g, 0.43 mmol), disaccharide donor **10** (0.62 g, 0.65 mmol) and freshly dried 4Å molecular sieves (300 mg) were suspended in dichloromethane (9 mL), stirred under argon for 20 min and then the mixture was cooled to -20 °C. A 0.1M solution of triethylsilyl trifluoromethanesulfonate in dichloromethane (0.86 mL, 0.086 mmol) was added dropwise and the disappearance of the starting material was followed by TLC (hexane/ethyl acetate 65:35). After 1 h, the reaction was quenched with TEA (0.6 mL), diluted with dichloromethane, filtered over Celite, and the solvent evaporated. The crude product was purified by flash chromatography (hexane/ethyl acetate, 45:55) to give 375 mg of **22a** and 125 mg of **22b** (85%, white solid).

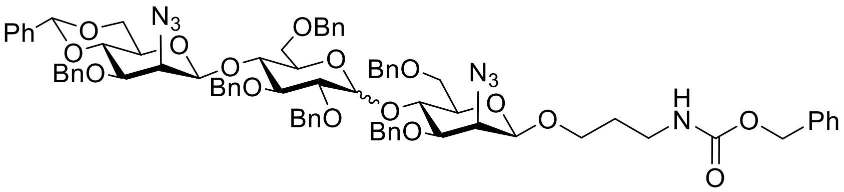

Chemical Formula: $C_{78}H_{83}N_7O_{16}$
Exact Mass: 1373,59
Molecular Weight: 1374,56

α anomer (**22a**):

R_f α = 0.37 (hexane/ethyl acetate 6:4)

$[\alpha]_D^{20}$ = -5.88 (*c* 1 in $CHCl_3$)

^{1}H NMR (500 MHz, $CDCl_3$) δ 7.65 – 6.99 (m, 40H, arom.), 5.68 (d, $J_{1'-2'}$ = 3.7 Hz, 1H, H-1'), 5.56 – 5.40 (m, 2H, C*H*Ph, N*H*Cbz), 5.15 – 5.05 (m, 2H, NCOOC*H*$_2$Ph), 4.99 (d, *J* = 10.7 Hz, 1H, OC*H*HPh), 4.86 – 4.73 (m, 2H, OCH*H*Ph, OC*H*HPh), 4.70 – 4.44 (m, 11H, OC*H*$_2$Ph, H-1'', H-1), 4.29 – 4.21 (br t, 1H, H-4), 4.15 (d, *J* = 12.2 Hz, 1H, OC*H*HPh), 4.01 – 3.75 (m, 9H, OC*H*HCH$_2$CH$_2$N, H-6a'', H-2, H-4'', H-4', H-3', H-6a, H-6b, H-3), 3.75 – 3.64 (m, 3H, H-5', OCH*H*CH$_2$CH$_2$N, H-2''), 3.63 – 3.56 (m, 1H, H-5), 3.56 – 3.25 (m, 7H, H-6b'', H-2', H-6a', OCH2CH2C*H*$_2$N, H-3'', H-6b'), 3.02 – 2.93 (m, 1H, H-5''), 1.94 – 1.75 (m, 2H, OCH$_2$C*H*$_2$CH$_2$N).

^{13}C NMR (126 MHz, $CDCl_3$) δ 156.56 (N*C*OOCH$_2$Ph), 139.22 – 136.78 (8C, quat.), 128.97 – 126.06 (40C, arom.), 101.49 (*C*HPh), 100.35 (C-1), 99.52 (C-1''), 96.88 (C-1'), 81.64 (C-3), 80.04 (C-4), 78.88 (C-2'), 78.48 (C-4'), 77.41 (C-4''), 76.52 (C-3'), 75.12 (C-5), 74.98 (O*C*H$_2$Ph), 73.60 (O*C*H$_2$Ph), 73.49 (O*C*H$_2$Ph), 73.18 (O*C*H$_2$Ph), 72.73 (O*C*H$_2$Ph), 71.42 (O*C*H$_2$Ph), 70.25 (C-5'), 69.78 (C-4), 69.14 (C-6), 68.34 (C-6''), 68.06 (C-6'), 67.36 (O*C*H$_2$CH$_2$CH$_2$N), 67.13 (C-5''), 66.54 (NCOO*C*H$_2$Ph), 63.69 (C-2''), 60.58 (C-2), 38.21 (OCH$_2$CH$_2$*C*H$_2$N), 29.64 (OCH$_2$*C*H$_2$CH$_2$N).

HRMS (ESI+): *m/z* for $C_{78}H_{83}N_7O_{16}Na$ calcd 1396.5794 $[M+Na]^+$, found 1396.5776.

β anomer (**22b**):

R_f β = 0.50 (hexane/ethyl acetate 6:4), 0.25 (hexane/ethyl acetate 7:3)

$[\alpha]_D^{20}$ = -33.06 (*c* 1 in $CHCl_3$)

^{1}H NMR ($CDCl_3$) δ 7.66 – 6.83 (m, 40H, arom.), 5.52 (s, 1H, C*H*Ph), 5.45 – 5.39 (br t, 1H, N*H*Cbz), 5.14 – 5.06 (m, 2H, NCOOC*H*$_2$Ph), 5.03 (d, *J* = 10.5 Hz, 1H, OC*H*HPh), 4.89 – 4.27 (m,

16H, OC*H*$_2$Ph, H-1, H-1'', H-1'), 4.12 – 4.00 (m, 2H, H-4, H-6a''), 4.00 – 3.82 (m, 5H. H-4', OC*H*HCH$_2$CH$_2$N, H-4'', H-2'', H-2'), 3.82 – 3.45 (m, 9H, H-6a', H-6b', H-6a, H-6b, OCH*H*CH$_2$CH$_2$N, H-3, H-6b'', H-3'', H-3'), 3.45 – 3.26 (m, 4H, OCH$_2$CH$_2$C*H*$_2$N, H-5, H-2'), 3.23 (br dt, 1H, H-5'), 3.05 (m, 1H, H-5''), 1.84 (m, 2H, OCH$_2$C*H*$_2$CH$_2$N).

^{13}C NMR (CDCl$_3$) δ 156.56 (N*C*OOCH$_2$Ph), 138.92 – 136.77 (8C, quat.), 128.99 – 126.05 (40C, arom.), 102.86 (C-1'), 101.51 (*C*HPh), 100.22 (C-1), 99.51 (C-1''), 83.12 (C-3'), 82.15 (C-2'), 78.42 (C-4''), 78.06 (C-3), 77.60 (C-4'), 76.53 (C-3''), 75.54 (C-5), 75.37 (O*C*H$_2$Ph), 75.02 (O*C*H$_2$Ph), 74.59 (C-4), 74.14 (C-5'), 73.59 (O*C*H$_2$Ph), 73.25 (O*C*H$_2$Ph), 72.76 (2C, O*C*H$_2$Ph), 68.56 (C-6), 68.38 (C-6''), 68.18 (C-6'), 67.30 (O*C*H$_2$CH$_2$CH$_2$N), 67.24 (C-5''), 66.52 (NCOO*C*H$_2$Ph), 63.60 (C-2), 62.34 (C-2''), 38.18 (OCH$_2$CH$_2$*C*H$_2$N), 29.70 (OCH$_2$*C*H$_2$CH$_2$N).

HRMS (ESI+): *m/z* for $C_{78}H_{83}N_7O_{16}Na$ calcd 1396.5794 [M+Na]$^+$, found 1396.5820.

N-(carbobenzyloxy)-3-aminopropyl 2-Acetamido-3-*O*-benzyl-4,6-*O*-benzylidene-2-deoxy-β-D-mannopyranosyl-(1→4)-2,3,6-tri-*O*-benzyl-α-D-glucopyranosyl-(1→4)-2-acetamido-3,6-di-*O*-benzyl-2-deoxy-β-D-mannopyranoside (23a)

Bis azido **22a** (260 mg, 0.19 mmol) was converted into bis acetamide **23a** as described in general Procedure D. Purification by flash chromatography (hexane/ethyl acetate gradient, 30:70 to 20:80) afforded **23a** (127 mg, 47%) as an amorphous solid.

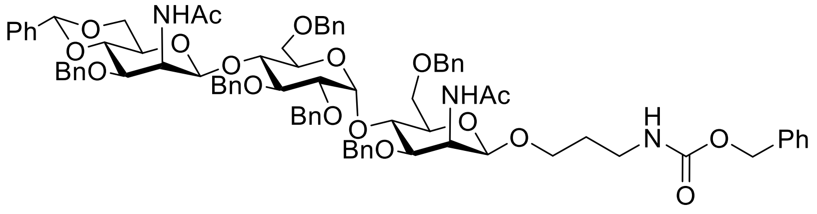

Chemical Formula: $C_{82}H_{91}N_3O_{18}$
Exact Mass: 1405,63
Molecular Weight: 1406,63

R_f = 0.38 (hexane/ethyl acetate 1:9)

$[\alpha]_D^{20}$ = -12.94 (*c* 1 in CHCl$_3$)

^{1}H NMR (500 MHz, CDCl$_3$) δ 7.65 – 6.96 (m, 40H, arom.), 5.88 (d, *J* = 9.3 Hz, 1H, N*H*Ac), 5.56 (br s, 1H, H-1'), 5.49 (m, 3H, C*H*Ph, N*H*Cbz, N*H*Ac), 5.11 (s, 2H, NCOOC*H*$_2$Ph), 4.95 (d, *J* = 11.6 Hz, 1H, OC*H*HPh), 4.88 – 4.39 (m, 13H, H-2, OC*H*$_2$Ph, H-2'', H-1'', H-1), 4.33 (d, *J* = 10.8 Hz, 1H, OCH*H*Ph), 4.26 (d, *J* = 12.0 Hz, 1H, OCH*H*Ph), 4.13 – 4.03 (m, 2H, H-4, H-6a''), 4.01 – 3.20 (m, 17H, H-4', OC*H*$_2$CH$_2$CH$_2$N, H-6a', H-6b', H-3, H-3', H-5, H-5', H-6b'', H-4'', H-2', H-6a, H-6b, H-3'', OCH$_2$CH$_2$C*H*$_2$N), 3.13 – 2.99 (m, 1H, H-5''), 1.96 (s, *J* = 21.6 Hz, 3H, NHCOC*H*$_3$), 1.86 – 1.71 (m, 5H, NHCOC*H*$_3$, OCH$_2$C*H*$_2$CH$_2$N).

^{13}C NMR (126 MHz, CDCl$_3$) δ 170.52 (NH*C*OCH$_3$), 170.41 (NH*C*OCH$_3$), 156.49 (N*C*OOCH$_2$Ph), 139.40 – 136.71 (8C, quat.), 129.01 – 126.10 (40C, arom.), 101.65 (*C*HPh), 99.79 (C-1), 98.97 (C-1''), 96.51 (C-1'), 80.15 (C-3'), 78.97 (C-3, C-2'), 78.58 (C-4''), 75.95 (C-4'), 75.77 (C-3''), 74.82 (O*C*H$_2$Ph), 74.54 (C-5), 73.51 (O*C*H$_2$Ph), 73.35 (O*C*H$_2$Ph), 72.76 (O*C*H$_2$Ph), 71.22 (O*C*H$_2$Ph),

70.95 (O*C*H_2Ph), 70.42 (C-5’), 69.58 (C-4), 69.44 (C-6’), 68.62 (C-6’’), 68.02 (C-6), 67.74 (O*C*$H_2CH_2CH_2$N), 67.06 (C-5’’), 66.63 (NCOO*C*H_2Ph), 50.52 (C-2’’), 48.04 (C-2), 38.92 (OCH_2CH_2*C*H_2N), 29.49 (OCH_2*C*H_2CH_2N), 23.31 (NHCO*C*H_3), 23.27 (NHCO*C*H_3).

HRMS (ESI+): *m/z* for $C_{82}H_{91}N_3O_{18}$Na calcd 1428.6195 $[M+Na]^+$, found 1428.6182.

3-aminopropyl 2-Acetamido-2-deoxy-β-D-mannopyranosyl-(1→4)-α-D-glucopyranosyl-(1→4)-2-acetamido-2-deoxy-β-D-mannopyranoside (3)

According to general Procedure G, compound **23a** (35 mg, 0.025 mmol) was hydrogenolyzed over $Pd(OH)_2$/C affording lyophilized trisaccharide **3** ·HCl as a white foam (16 mg, quantitative yield).

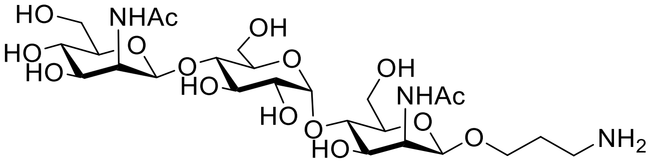

Chemical Formula: $C_{25}H_{45}N_3O_{16}$
Exact Mass: 643,28
Molecular Weight: 643,64

R_f = 0.22 (ethyl acetate/isopropanol/water 3:3:2)

$[\alpha]_D^{20}$ = +7.11 (*c* 1 in H_2O)

^{1}H NMR (500 MHz, D_2O) δ 5.27 (d, $J_{1'-2'}$ = 3.6 Hz, 1H, H-1’), 4.81 (br s, 1H, H-1’’), 4.75 (br s, 1H, H-1), 4.48 (br d, *J* = 4.3 Hz, 1H, H-2), 4.42 (br d, *J* = 4.3 Hz, 1H, H-2’’), 4.03 (dd, J_{3-4} = 9.2, J_{3-2} = 4.3 Hz, 1H, H-3), 3.95 – 3.88 (m, 1H, OC*H*HCH_2CH_2N), 3.88 – 3.80 (m, 2H, H-6a’’, H-6a), 3.80 – 3.62 (m, 9H, H-6b’’, H-3’’, H-6a’, H-6b’, H-5’, OCH*H*CH_2CH_2N, H-4, H-3’, H-6b), 3.59 (br t, $J_{4'-3'}$ = $J_{4'-5'}$ = 9.0 Hz, 1H, H-4’), 3.54 (br dd, $J_{2'-3'}$ = 9.7, $J_{2'-1'}$ = 3.6 Hz, 1H, H-2’), 3.50 – 3.41 (m, 2H, H-5, H-4’’), 3.41 – 3.30 (m, 1H, H-5’’), 3.04 (m, 2H, OCH_2CH_2C*H_2*N), 2.00 (s, 6H, 2x NHCOC*H_3*), 1.92 – 1.83 (m, 2H, OCH_2C*H_2*CH_2N).

^{13}C NMR (D_2O) δ 175.48 (NH*C*OCH_3), 175.45 (NH*C*OCH_3), 99.32 (C-1’, C-1’’), 99.11 (C-1), 78.50 (C-4’), 76.52 (C-5’’), 74.89 (C-5), 74.06 (C-4), 72.26 (C-3), 71.95 (C-3’’), 71.38 (C-5’), 71.35 (C-2’), 71.05 (C-3’), 67.45 (O*C*$H_2CH_2CH_2$N), 66.61 (C-4’’), 60.42 (C-6’’), 60.37 (C-6’), 59.96 (C-6), 53.26 (C-2), 53.10 (C-2’’), 37.68 (OCH_2CH_2*C*H_2N), 26.51 (OCH_2*C*H_2CH_2N), 22.01 (NHCO*C*H_3), 21.97 (NHCO*C*H_3).

HRMS (ESI+): *m/z* for $C_{25}H_{45}N_3O_{16}$Na calcd 666.2698 $[M+Na]^+$, found 666.2708; *m/z* for $C_{25}H_{46}N_3O_{16}$ calcd 644.2878 $[M+H]^+$, found 644.2883.

***N*-(carbobenzyloxy)-3-aminopropyl (2-Acetamido-3-*O*-benzyl-4,6-*O*-benzylidene-2-deoxy-β-D-mannopyranosyl)-(1→4)-(2,3,6-tri-*O*-benzyl-α-D-glucopyranosyl)-(1→4)-(2-acetamido-3,6-di-*O*-benzyl-2-deoxy-β-D-mannopyranoside (23b)**

Compound **22b** (125 mg, 0.09 mmol) was converted into bis acetamide **23b** as described in general Procedure D. Purification by flash chromatography (hexane/ethyl acetate gradient, 20:80 to 10:90) afforded **23b** (38 mg, 30%) as an amorphous solid.

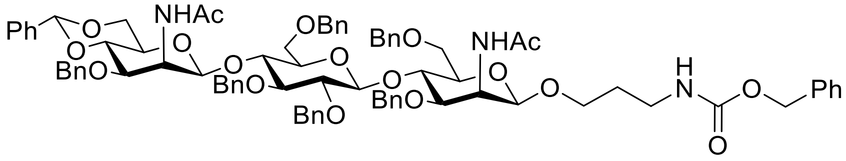

Chemical Formula: $C_{82}H_{91}N_3O_{18}$
Exact Mass: 1405,63
Molecular Weight: 1406,63

R_f = 0.17 (hexane/ethyl acetate 5:95)

$[\alpha]_D^{20}$ = -31.58 (*c* 1 in $CHCl_3$)

^{1}H NMR (500 MHz, $CDCl_3$) δ 7.55 – 7.13 (m, 40H, arom.), 5.82 (br d, 1H, N*H*Ac), 5.64 – 5.51 (m, 2H, N*H*Cbz, N*H*Ac), 5.50 (s, 1H, C*H*Ph), 5.10 (m, 2H, NCOOC*H*$_2$Ph), 4.93 – 4.38 (m, 17H, 6x OC*H*$_2$Ph, H-2'', H-1'', H-2, H-1, H-1'), 4.12 (dd, *J* = 10.4, 4.8 Hz, 1H, H-6a''), 4.04 – 3.97 (m, 2H, H-4, H-4'), 3.93 – 3.86 (m, 1H, OC*H*HCH$_2$CH$_2$N), 3.83 – 3.75 (m, 2H, H-6a, H-6b), 3.75 – 3.67 (m, 3H, H-3, H-5, H-6a'), 3.67 – 3.56 (m, 3H, H-6b', H-6b'', H-4''), 3.56 – 3.47 (m, 2H, OCH*H*CH$_2$CH$_2$N, H-3''), 3.47 – 3.37 (m, 2H, H-3', H-2'), 3.37 – 3.28 (m, 2H, OCH$_2$CH$_2$C*H*$_2$N), 3.24 (m, 1H, H-5'), 3.21 – 3.08 (m, 1H, H-5''), 1.88 (s, 6H, 2x NHCOC*H*$_3$), 1.78 (s, 2H, OCH$_2$C*H*$_2$CH$_2$N).

^{13}C NMR (126 MHz, $CDCl_3$) δ 170.41 (NH*C*OCH$_3$), 170.32 (NH*C*OCH$_3$), 156.48 (N*C*OOCH$_2$Ph), 139.06 – 136.73 (8C, quat.), 129.01 – 126.10 (40C, arom.), 102.73 (C-1'), 101.63 (*C*HPh), 100.04 (C-1''), 98.50 (C-1), 83.30 (C-3'), 81.96 (C-2'), 78.73 (C-4''), 76.52 (C-4'), 75.88 (C-3), 75.82 (C-3''), 75.02 (O*C*H$_2$Ph), 74.88 (O*C*H$_2$Ph), 75.40 (C-5'), 74.36 (C-5), 73.52 (C-4), 73.41 (O*C*H$_2$Ph), 73.22 (O*C*H$_2$Ph), 71.50 (2C, O*C*H$_2$Ph), 69.09 (C-6), 68.69 (C-6''), 68.50 (C-6'), 67.65 (O*C*H$_2$CH$_2$CH$_2$N), 67.06 (C-5''), 66.60 (NCOO*C*H$_2$Ph), 50.41 (C-2''), 47.78 (C-2), 39.02 (OCH$_2$CH$_2$*C*H$_2$N), 29.44 (OCH$_2$*C*H$_2$CH$_2$N), 23.26 (NHCO*C*H$_3$), 23.23 (NHCO*C*H$_3$).

HRMS (ESI+): *m/z* for $C_{82}H_{91}N_3O_{18}Na$ calcd 1428.6195 $[M+Na]^+$, found 1428.6191.

3-aminopropyl 2-Acetamido-2-deoxy-β-D-mannopyranosyl-(1→4)-β-D-glucopyranosyl-(1→4)-2-acetamido-2-deoxy-β-D-mannopyranoside (4)

According to general Procedure G, compound **23b** (33 mg, 0.023 mmol) was hydrogenolyzed over $Pd(OH)_2$/C affording lyophilized trisaccharide **4** ·HCl as a white foam (14 mg, quantitative yield).

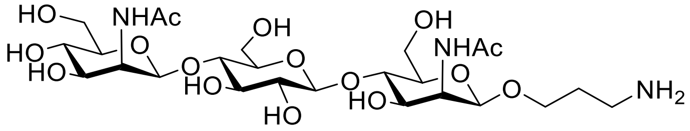

Chemical Formula: $C_{25}H_{45}N_3O_{16}$
Exact Mass: 643,28
Molecular Weight: 643,64

R_f = 0.11 (ethyl acetate/isopropanol/water 3:3:1.5)

$[\alpha]_D^{20}$ = -1.40 (*c* 1 in H_2O)

^{1}H NMR (D_2O) δ 4.81 (s, 1H, H-1''), 4.74 (s, 1H, H-1), 4.48 (m, 2H, H-2, H-2''), 4.43 (d, $J_{1'-2'}$ = 7.9 Hz, 1H, H-1'), 3.97 – 3.54 (m, 13H, OC*H*$_2$CH$_2$CH$_2$N, H-6a'', H-6b'', H-6a, H-6b, H-6a', H-6b', H-3, H-3'', H-4, H-4', H-3'), 3.54 – 3.41 (m, 3H, H-5', H-5, H-4''), 3.41 – 3.33 (m, 1H, H-5''), 3.30 – 3.23 (m, 1H, H-2'), 3.12 – 2.97 (m, 2H, OCH$_2$CH$_2$C*H*$_2$N), 2.00 (s, 6H, 2x NHCOC*H*$_3$), 1.89 (m, 2H, OCH$_2$C*H*$_2$CH$_2$N).

^{13}C NMR (D_2O) δ 175.43 (NH*C*OCH$_3$), 102.46 (C-1'), 99.31 (C-1''), 99.21 (C-1), 78.48 (C-4'), 76.50 (C-5''), 76.10 (C-4), 75.24 (C-5'), 74.57 (C-5), 73.93 (C-3'), 72.96 (C-2'), 71.92 (C-3''), 70.34 (C-3), 67.50 (O*C*H$_2$CH$_2$CH$_2$N), 66.60 (C-4''), 60.36, 60.01, 59.78 (C-6, C-6', C-6''), 53.22 , 52.58 (C-2, C-2''), 37.70 (OCH$_2$CH$_2$*C*H$_2$N), 26.50 (OCH$_2$*C*H$_2$CH$_2$N), 21.99 (2C, NHCO*C*H$_3$).

HRMS (ESI+): *m/z* for $C_{25}H_{45}N_3O_{16}Na$ calcd 666.2698 $[M+Na]^+$, found 666.2691; *m/z* for $C_{25}H_{46}N_3O_{16}$ calcd 644.2878 $[M+H]^+$, found 644.2872.

_N_-(Carbobenzyloxy)-3-aminopropyl 2-Acetamido-3,6-di-_O_-benzyl-4-_O_-[(_N,N_-di-isopropylamino)-2-cyanoethylphosphite]-2-deoxy-β-D-mannopyranoside (24)

To a solution of compound **9** (125 mg, 0.21 mmol) in dichloromethane (4 mL), freshly distilled DIPEA (0.15 mL, 0.84 mmol) and 2-cyanoethoxy-*N,N*-diisopropylamidochloridophosphite (95 µL, 0.42 mmol) were consecutively added. The reaction was stirred at room temperature for one hour, then diluted with ethyl acetate (25 mL), and washed with brine (2 x 30 mL) and water (30 mL). The organic layer was dried with sodium sulphate and concentrated *in vacuo*. The residue was purified by flash column chromatography (hexane/ethyl acetate, 20:80 + 0.5% TEA) to give phosphoramidite **24** (150 mg, 90%, white solid) in a 1:1 mixture of (*R*,*S*)-diastereomers at phosphorus.

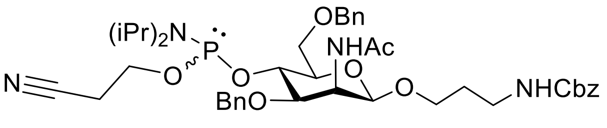

Chemical Formula: $C_{42}H_{57}N_4O_9P$
Exact Mass: 792,39
Molecular Weight: 792,91

R_f = 0.25 (hexane/ethyl acetate 25:75)

(*R*)/(*S*) diastereomers at Phosphorus = 1:1

^{1}H NMR (500 MHz, $CDCl_3$) δ 7.51 – 7.12 (m, 15H, arom.), 6.00 – 5.90 (m, 1H, N*H*Ac), 5.72 (br t, 0.5H, N*H*Cbz), 5.59 (br t, 0.5H, N*H*Cbz), 5.22 – 5.02 (m, 2H, NCOOC*H*$_2$Ph), 4.87 – 4.75 (m, 2H, H-2, OC*H*HPh), 4.69 – 4.39 (m, 4H, H-1, OCH*H*Ph, OC*H*$_2$Ph), 4.07 – 3.82 (m, 3H, H-4, OC*H*HCH$_2$CH$_2$N, H-6a), 3.78 – 3.44 (m, 7H, H-6b, H-5, H-3, OCH*H*CH$_2$CH$_2$N, N[C*H*(CH$_3$)$_2$]$_2$, OC*H*$_2$CH$_2$CN), 3.43 – 3.20 (m, 2H, OCH$_2$CH$_2$C*H*$_2$N), 2.44 – 2.21 (m, 2H, OCH$_2$C*H*$_2$CN), 1.99 (s, 1.5H, NHCOC*H*$_3$), 1.94 (s, 1.5H, NHCOC*H*$_3$), 1.80 (m, 2H, OCH$_2$C*H*$_2$CH$_2$N), 1.20 (m, 12H, N[CH(C*H*$_3$)$_2$]$_2$).

^{31}P NMR ($CDCl_3$) δ 150.77, 149.99.

^{13}C NMR (126 MHz, $CDCl_3$) δ 171.07 and 170.70 (NH*C*OCH_3), 156.61 and 156.54 (N*C*OOCH_2Ph), 138.33 – 136.75 (3C, quat.), 128.75 – 127.60 (15C, arom.), 118.27 and 117.98 (OCH_2CH_2*C*N), 99.52 and 98.95 (C-1), 78.94 and 77.93 (C-3), 75.72 (0.5C, C-5), 75.57 (d, $J_{5\text{-}P}$ = 4.1 Hz, 0.5C, C-5), 73.31 (O*C*H_2Ph), 71.14 and 70.96 (O*C*H_2Ph), 69.75 and 69.61 (C-6), 69.50 and 69.47 (d, $J_{4\text{-}P}$ = 3.6 Hz, 0.5C, C-4), 69.34 (br d, 0.5C, C-4), 67.48 (O*C*$H_2CH_2CH_2$N), 66.54 and 66.45 (NCOO*C*H_2Ph), 58.37 (d, $J_{CH\text{-}P}$ = 19.1 Hz, 0.5C, O*C*H_2CH_2CN), 57.51 (d, $J_{CH\text{-}P}$ = 23.9 Hz, 0.5C, O*C*H_2CH_2CN), 48.60 and 48.06 (C-2), 43.43 (d, $J_{CH\text{-}P}$ = 12.3 Hz, 1C, N[*C*H(CH_3)$_2$]$_2$), 43.09 (d, $J_{CH\text{-}P}$ = 12.8 Hz, 1C, N[*C*H(CH_3)$_2$]$_2$), 38.66 and 38.34 (OCH_2CH_2*C*H_2N), 29.46 (OCH_2*C*H_2CH_2N), 24.74 and 24.68 (1C, N[CH(*C*H_3)$_2$]$_2$), 24.62 and 24.57 (2C, N[CH(*C*H_3)$_2$]$_2$), 24.44 and 24.39 (1C, N[CH(*C*H_3)$_2$]$_2$), 23.31 and 23.25 (NHCO*C*H_3), 20.09 and 20.04 (OCH_2*C*H_2CN).

HRMS (ESI+): *m/z* for $C_{42}H_{57}N_4O_9NaP$ calcd 815.3761 $[M+Na]^+$, found 815.3762.

2-Cyanoethyl {2-acetamido-3,6-di-*O*-benzyl-1-*O*-[*N*-(Carbobenzyloxy)-γ-aminopropyl]-2-deoxy-β-D-mannopyranos-4-yl} [(2-acetamido-3-*O*-benzyl-4,6-*O*-benzylidene-2-deoxy-β-D-mannopyranosyl)-(1→4)-(2,3,6-tri-*O*-benzyl-α-D-glucopyranos-1-yl)] phosphate (26)

Compound **11** (74 mg, 0.088 mmol, 5:1 α/β anomeric mixture) and phosphoramidite **24** (140 mg, 0.18 mmol) were separately pre-dried by 3 co-evaporations with dry acetonitrile. Compound **11**, 4,5-dicyanoimidazole (DCI, 24 mg, 0.20 mmol) and 4Å MS (60 mg) were dissolved in acetonitrile (3 mL) and stirred at room temperature. After 15 min, phosphoramidite **24** dissolved in acetonitrile (3 mL) was added dropwise. After 2 h, TLC analysis confirmed the consumption of compound **8**, then the reaction mixture was diluted with ethyl acetate, filtered over a Celite pad and concentrated under reduced pressure. The residue was purified by flash chromatography to remove a more polar by-product which in TLC analysis would co-spott with the product of the next oxidation step (R_f by-product = 0.16 in toluene/acetone 6:4). Flash chromatography (toluene/acetone gradient, 80:20 to 60:40) gave phosphite **25** (125 mg; R_f α/β = 0.35 in toluene/acetone 6:4 and 0.25 in hexane/ethyl acetate 2:8; LRMS (ESI+): *m/z* for $C_{85}H_{95}N_4O_{21}NaP$ calcd 1561.6124 $[M+Na]^+$, found 1561.6144) as a complex diastereomeric mixture: both ^{31}P-NMR and ^{1}H-^{13}C HSQC analysis showed 4 signals demonstrating the formation of either (*R*) and (*S*) diastereomers at phosphorus and the α/β anomers. Intermediate **25** (115 mg, 0.075 mmol) was dissolved in acetonitrile (4 mL) and subjected to oxidation by addition of a ~5.5 M solution of *tert*-Butyl hydroperoxide in decane (60 µL, 0.30 mmol) at 0 °C. The reaction mixture was allowed to warm to room temperature and stirred for 18 h, then concentrated under reduced pressure. Purification by flash-chromatography (toluene/acetone gradient, 80:20 to 60:40) afforded **26** (64 mg of α anomer and 20 mg of β, 73%, glassy solid,) in an easily separable 4:1 α/β mixture.

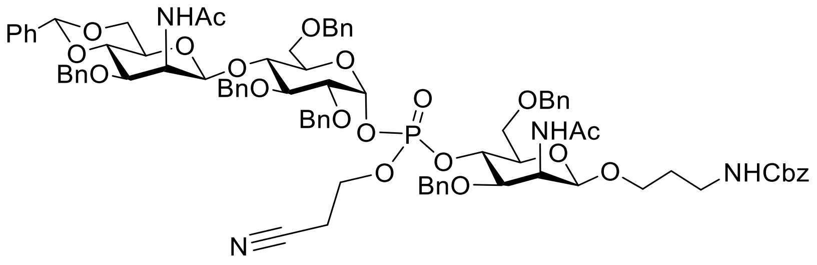

Chemical Formula: $C_{85}H_{95}N_4O_{21}P$
Exact Mass: 1538,62
Molecular Weight: 1539,68

R_f α = 0.27 and R_f β = 0.19 (toluene/acetone 6:4)

α anomer (**26α**):

$[\alpha]_D^{20}$ = + 1.63 (*c* 1 in $CHCl_3$)

^{1}H NMR (500 MHz, $CDCl_3$) δ 7.60 – 7.12 (m, 40H, arom.), 6.07 (br d, 1H, N*H*Ac), 5.83 (br dd, 1H, H-1'), 5.59 (br d, 1H, N*H*Ac), 5.48 (s, 1H, C*H*Ph), 5.46 – 5.35 (br t, 1H, N*H*Cbz), 5.11 (m, 2H, NCOOCH_2Ph), 4.97 – 4.81 (m, 4H, H-2, OC*H*HPh, OCH_2Ph), 4.79 – 4.30 (m, 13H, 9x , OC*H*HPh, H-1, H-1'', H-2'', H-4), 4.10 (br dd, 1H, H-6a'), 4.06 – 3.70 (m, 9H, H-3', H-4', H-5', H-6a, H-6b, H-6a', OCH_2CH$_2$CN, OC*H*HCH$_2$CH$_2$N), 3.70 – 3.50 (m, 7H, H-2', H-3, H-4'', H-5, H-6b', H-6b'', OCH*H*CH$_2$CH$_2$N), 3.45 (br dd, 1H, H-3''), 3.41 – 3.21 (m, 2H, OCH$_2$CH$_2$CH_2N), 3.21 – 3.05 (m, 1H, H-5''), 2.25 – 2.14 (m, 1H, OCH$_2$C*H*HCN), 2.10 – 1.72 (m, 9H, OCH$_2$CH*H*CN, 2x NHCOCH_3, OCH$_2$CH_2CH$_2$N).

^{31}P NMR ($CDCl_3$) δ -2.48.

^{13}C NMR (126 MHz, $CDCl_3$) δ 171.04 and 170.55 (2C, NH*C*OCH$_3$), 156.53 (N*C*OOCH$_2$Ph), 138.88 – 136.73 (8C, quat.), 129.05 – 125.31 (40C, arom.), 116.65 (OCH$_2$CH$_2$*C*N), 101.62 (*C*HPh), 100.20 (C-1''), 99.31 (C-1), 95.83 (d, $J_{1'-P}$ = 5.4 Hz, 1C, C-1'), 79.52 (C-3'), 78.93 (d, $J_{2'-P}$ = 6.9 Hz, 1C, C-2'), 78.58 (C-4''), 77.45 (C-3), 76.13 (C-4'), 75.59 (C-3''), 75.11 (O*C*H$_2$Ph), 74.53 (d, J_{5-P} = 5.7 Hz, 1C, C-5), 73.64 (d, J_{4-P} = 5.7 Hz, 1C, C-4), 73.52 (2C, O*C*H$_2$Ph), 73.47 (O*C*H$_2$Ph), 72.09 (C-5'), 71.39 (O*C*H$_2$Ph), 71.18 (O*C*H$_2$Ph), 68.77 (C-6''), 68.58 (C-6'), 67.98 (C-6), 67.69 (O*C*H$_2$CH$_2$CH$_2$N), 67.09 (C-5''), 66.58 (NCOO*C*H$_2$Ph), 62.01 (d, J_{CH-P} = 4.3 Hz, 1C, O*C*H$_2$CH$_2$CN), 50.35 (C-2''), 48.64 (C-2), 38.54 (OCH$_2$CH$_2$*C*H$_2$N), 29.70 (OCH$_2$*C*H$_2$CH$_2$N), 23.34 and 23.23 (2C, NHCO*C*H$_3$), 18.78 (d, J_{CH-P} = 6.6 Hz, 1C, OCH$_2$*C*H$_2$CN).

HRMS (ESI+): *m/z* for $C_{85}H_{95}N_4O_{21}NaP$ calcd 1561.6124 $[M+Na]^+$, found 1561.6144.

{2-Acetamido-3,6-di-*O*-benzyl-1-*O*-[*N*-(Carbobenzyloxy)-γ-aminopropyl]-2-deoxy-β-D-mannopyranos-4-yl} [(2-acetamido-3-*O*-benzyl-4,6-*O*-benzylidene-2-deoxy-β-D-mannopyranosyl)-(1→4)-(2,3,6-tri-*O*-benzyl-α-D-glucopyranos-1-yl)] phosphate, triethylammonium salt (27)

To a solution of compound **26** (59 mg, 0.038 mmol) in dichloromethane (2 mL), freshly distilled triethylamine (0.20 mL) was added dropwise at 0°C, then the reaction was allowed to warm to room temperature. After 24 h, another portion of TEA (0.10 mL) was added and the reaction was stirred

for additional 48 h. After solvents removal by rotavap evaporation, purification by flash chromatography (dichloromethane/methanol, 95:5) gave **27** (48 mg, 80%, colourless glassy solid) as the triethylammonium salt.

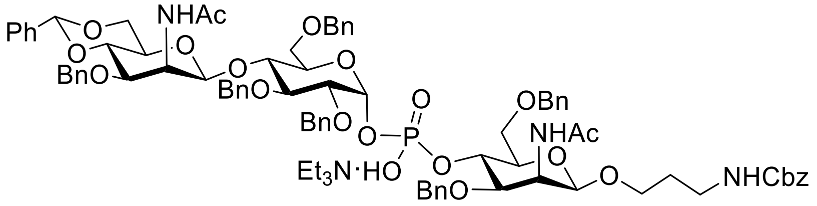

Chemical Formula: $C_{82}H_{92}N_3O_{21}P$
Exact Mass: 1485,60
Molecular Weight: 1486,61

R_f = 0.53 (dichloromethane/methanol 9:1)

$[\alpha]_D^{20}$ = -4.33 (*c* 1 in CH_3OH)

^{1}H NMR (500 MHz, $CDCl_3$) δ 7.60 – 7.09 (m, 40H, arom.), 5.98 (br d, 1H, N*H*Ac), 5.92 (br dd, $J_{1',P}$ = 7.2, $J_{1',2'}$ = 3 Hz, 1H, H-1'), 5.63 (m, 1H, N*H*Ac), 5.58 (br t, 1H, N*H*Cbz), 5.45 (br s, 1H, C*H*Ph), 5.07 (m, 2H, NCOOCH_2Ph), 4.92 (d, *J* = 11.3 Hz, 1H, OC*H*HPh), 4.80 (m, 2H, OC*H*HPh, OCH*H*Ph), 4.74 – 4.40 (m, 14H, H-1'', H-2, H-2'', H-1, H-4, 9x OC*H*HPh), 4.13 (m, 1H, H-5'), 4.09 – 3.74 (m, 8H, H-4', H-6a'', H-6a', H-3', H-6b', OC*H*HCH_2CH_2N, H-6a, H-5), 3.73 – 3.40 (m, 7H, H-3, H-6b, H-6b'', H-4'', H-2', OCH*H*CH_2CH_2N, H-3''), 3.38 – 3.21 (m, 2H, $OCH_2CH_2CH_2N$), 3.21 – 3.07 (m, 1H, H-5''), 2.88 – 2.70 (m, 6H, $N(CH_2CH_3)_3$), 1.83 (s, 3H, $NHCOCH_3$), 1.80 (s, 3H, $NHCOCH_3$), 1.78 – 1.66 (m, 2H, $OCH_2CH_2CH_2N$), 1.13 (t, *J* = 7.3 Hz, 9H, $N(CH_2CH_3)_3$).

^{31}P NMR ($CDCl_3$) δ -2.15.

^{13}C NMR (126 MHz, $CDCl_3$) δ 170.35 and 170.29 ($NHCOCH_3$), 156.48 ($NCOOCH_2Ph$), 139.38 – 136.78 (8C, quat.), 128.95 – 126.09 (40C. arom.), 101.51 (*C*HPh), 100.49 (C-1''), 98.49 (C-1), 93.01 (d, $J_{1',P}$ = 6.0 Hz, 1C, C-1'), 80.21 (C-3'), 79.81 (d, $J_{2',P}$ = 7.6 Hz, 1C, C-2'), 78.73 (C-4''), 77.14 (C-3), 77.06 (C-4'), 75.90 (C-3''), 75.18 (d, $J_{5,P}$ = 4.0 Hz, 1C, C-5), 74.80 (OCH_2Ph), 73.43 (OCH_2Ph), 73.07 (OCH_2Ph), 72.18 (OCH_2Ph), 71.48 (OCH_2Ph), 71.34 (OCH_2Ph), 70.58 (C-5'), 70.29 (br d, C-4), 70.26 (C-6'), 68.70 (C-6''), 68.56 (C-6), 67.53 ($OCH_2CH_2CH_2N$), 66.91 (C-5''), 66.48 ($NCOOCH_2Ph$), 50.47 (C-2''), 47.87 (C-2), 45.25 ($N(CH_2CH_3)_3$), 38.86 ($OCH_2CH_2CH_2N$), 29.45 ($OCH_2CH_2CH_2N$), 23.20 ($NHCOCH_3$), 23.14 ($NHCOCH_3$), 8.38 (3C, $N(CH_2CH_3)_3$).

HRMS (ESI-): *m/z* for $C_{82}H_{92}N_3O_{21}P$ calcd 1484.5883 $[M-H]^-$, found 1484.5897.

[2-Acetamido-1-*O*-(γ-aminopropyl)-2-deoxy-β-D-mannopyranos-4-yl] [(2-acetamido-2-deoxy-β-D-mannopyranosyl)-(1→4)-(α-D-glucopyranos-1-yl)] phosphate, sodium salt (5)

Compound **27** (32 mg, 0.0214 mmol) was hydrogenolyzed over $Pd(OH)_2$/C in a 2:1 mixture of MeOH and H_2O (2mL, 0.01M) with an additional amount of 0.16 mL of HCl 0.1N. After 4 days at room temperature (NMR analysis indicated complete conversion), the reaction mixture was filtered over a Celite pad, and the filtrate was concentrated to 1 mL. The water solution of deprotected

compound was eluted over a column filled with Dowex® 50WX8 resin (H^+ form), followed by a second ion exchange column on the same resin in Na^+ form. Lyophilization of the eluates provided disaccharide phosphodiester **5** as the sodium salt (13 mg, 90% yield).

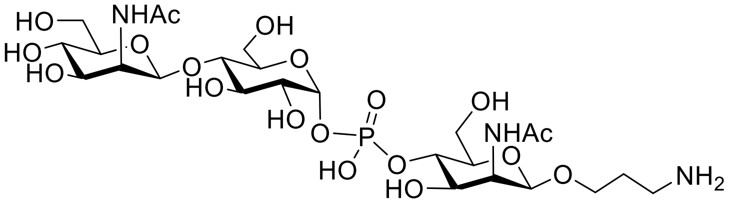

Chemical Formula: $C_{25}H_{46}N_3O_{19}P$
Exact Mass: 723,2463
Molecular Weight: 723,6188

R_f = 0.16 (ethyl acetate/isopropanol/water 1:1:1 + 1% TEA)

$[\alpha]_D^{20}$ = -2.71 (*c* 0.5 in H_2O)

^{1}H NMR (500 MHz, D_2O) δ 5.48 (br dd, $J_{1',P}$ = 6.5, $J_{1',2'}$ = 3.5 Hz, 1H, H-1'), 4.83 (s, 1H, H-1''), 4.73 (s, 1H, H-1), 4.51 – 4.42 (m, 2H, H-2, H-2''), 4.07 – 3.32 (m, 18H, H-4, H-3, H-4', H-6a, H-6b, H-6a', H-6b', H-6a'', H-6b'', H-3', H-3'', H5', H-2', H-5'', H-5, H-4'', OCH_2CH$_2$CH$_2$N), 3.15 – 3.01 (m, 2H, OCH$_2$CH$_2$CH_2N), 2.07 – 1.88 (m, 8H, 2x NHCOCH_3, OCH$_2$CH_2CH$_2$N).

^{31}P NMR (D_2O) δ -1.42.

^{13}C NMR (D_2O) δ 175.48 (2C, NH*C*OCH$_3$), 99.33 (C-1), 99.27 (C-1''), 95.47 (d, $J_{1',P}$ = 5.9 Hz, 1C, C-1'), 78.21 (C-5'), 76.53 (C-4''), 75.60 (d, $J_{5,P}$ = 4.9 Hz, 1C, C-5), 72.03 (C-3''), 71.80 (d, $J_{4,P}$ = 5.4 Hz, 1C, C-4), 71.35 (C-3'), 71.29 (2C, C-4', C-2'), 71.03 (C-3), 67.32 (O*C*H$_2$CH$_2$CH$_2$N), 66.67 (C-5''), 60.42 (2C, C-6, C-6''), 59.80 (C-6'), 55.86 (OCH$_2$CH$_2$*C*H$_2$N), 53.32 and 53.03 (C-2, C-2''), 24.38 (OCH$_2$*C*H$_2$CH$_2$N), 22.05 (2C, NHCO*C*H$_3$).

HRMS (ESI-): *m/z* for $C_{25}H_{26}N_3O_{19}P$ calcd 723.2463 [M-H]$^-$, found 722.2294.

8 References

(1) Dasler, W.; Bauer, C. D. Removal of Peroxides from Organic Solvents. *Ind. Eng. Chem. - Anal. Ed.* **1946**, *18* (1), 52–54. https://doi.org/0.1021/i560149a017.

(2) Lab Protocol for Molybdic Solution: 21 g of $(NH_4)_4Mo_4O_{24}$, 1g of $Ce(SO_4)_2$, 31 mL of H_2SO_4 98%, 970 mL of Water.

(3) Lab Protocol for Ethanolic H_2SO_4 Solution: 50 mL of H_2SO_4 98%, 450 mL of EtOH, 450 mL of Water.

(4) Lab Protocol for Ninhydrin Solution: 2.7g of 2,2-Dihydroxyindane-1,3-Dione, 27 mL of AcOH, 900 mL of EtOH.

(5) Still, W. C.; Kahn, M.; Mitra, A. Rapid Chromatographic Technique for Preparative Separations with Moderate Resolution. *J. Org. Chem.* **1978**, *43* (14), 2923–2925. https://doi.org/10.1021/jo00408a041.

(6) Ren, B.; Wang, M.; Liu, J.; Ge, J.; Zhang, X.; Dong, H. Zemplén Transesterification: A Name Reaction That Has Misled Us for 90 Years. *Green Chem.* **2015**, *17* (3), 1390–1394. https://doi.org/10.1039/c4gc02006e.

(7) Wang, Z. Zemplén Deacetylation. In *Comprehensive Organic Name Reactions and Reagents*; 2010; pp 3123–3128. https://doi.org/10.1002/9780470638859.

(8) Bousquet, E.; Khitri, M.; Lay, L.; Nicotra, F.; Panza, L.; Russo, G. Capsular Polysaccharide of Streptococcus Pneumoniae Type 19F: Synthesis of the Repeating Unit. *Carbohydr. Res.* **1998**, *311* (4), 171–181. https://doi.org/10.1016/S0008-6215(98)00218-3.

(9) Yang, S.; Liu, Q.; Zhang, G.; Zhang, X.; Zhao, Z.; Lei, P. An Approach to Synthesize Chondroitin Sulfate-E (CS-E) Oligosaccharide Precursors. *J. Org. Chem.* **2018**, *83* (11), 5897–5908. https://doi.org/10.1021/acs.joc.8b00157.

(10) Torres-Sánchez, M. I.; Draghetti, V.; Panza, L.; Lay, L.; Russo, G. Synthesis of the Phosphono Analogue of the Dimeric Subunit of Neisseria Meningitidis Type A Capsular Polysaccharide. *Synlett* **2005**, No. 7, 1147–1151. https://doi.org/10.1055/s-2005-865226.

(11) Bayley, H.; Standring, D. N.; Knowles, J. R. Propane-1,3-Dithiol: A Selective Reagent for the Efficient Reduction of Alkyl and Aryl Azides to Amines. *Tetrahedron Lett.* **1978**, *39*, 3633–3634.

(12) Oltvoort, J. J.; van Boeckel, C. A. A.; de Koning, J. H.; van Boom, J. H. Use of the Cationic Iridium Complex 1,5-Cyclooctadiene-Bis[Methyldiphenylphosphine]-Iridium Hexafluorophosphate in Carbohydrate Chemistry: Smooth Isomerization of Allyl Ethers to 1-Propenyl Ethers. *Synthesis (Stuttg).* **1981**, *1981* (4), 305–308. https://doi.org/10.1055/s-1981-29429.

(13) Soliveri, G.; Bertolotti, A.; Panza, L.; Poletti, L.; Jones, C.; Lay, L. Synthesis of Phosphorylated Fragments of Streptococcus Pneumoniae Type 19F Capsular Polysaccharide. *J. Chem. Soc. Perkin 1* **2002**, *19*, 2174–2181. https://doi.org/10.1039/b205684d.

(14) Wang, Y.; Yan, Q.; Wu, J.; Zhang, L. H.; Ye, X. S. A New One-Pot Synthesis of α-Gal Epitope Derivatives Involved in the Hyperacute Rejection Response in Xenotransplantation. *Tetrahedron* **2005**, *61* (17), 4313–4321. https://doi.org/10.1016/j.tet.2005.02.023.

(15) Lenagh-Snow, G. M. J.; Araújo, N.; Jenkinson, S. F.; Martínez, R. F.; Shimada, Y.; Yu, C. Y.; Kato, A.; Fleet, G. W. J. Azetidine Iminosugars from the Cyclization of 3,5-Di-O-Triflates of α-Furanosides and of 2,4-Di-O-Triflates of β-Pyranosides Derived from Glucose. *Org. Lett.* **2012**, *14* (8), 2142–2145. https://doi.org/10.1021/ol300669v.

(16) Fleet, G. W. J.; Witty, D. R. Synthesis of Homochiral β-Hydroxy-α-Aminoacids [(2S,3R,4R)-3,4-Dihydroxyproline and (2S,3R,4R)-3,4-Dihydroxypipecolic Aicd] and of 1,4-Dideoxy-1,4-Imino-D-Arabinitol [DAB1] and Fagomine [1,5-Imino-1,2,5-Trideoxy-D-Arabino-Hexitol]. *Tetrahedron: Asymmetry* **1990**, *1* (2), 119–136. https://doi.org/doi 10.1016/S0957-4166(00)86337-5.

(17) Bichard, C. J. F.; Wheatley, J. R.; Fleet George W. J. Acetonides of Heptonolactones: Kiliani Ascension of 3-O-Benzyl-D-Glucose and 3-O-Benzyl-D-Allose. *Tetrahedron Lett.* **1994**, *5* (3), 431–440. https://doi.org/10.1016/S0957-4166(00)86215-1.

(18) Kosáková, Ľ.; Košíková, B.; Joniak, D. Synthesis of D-Glucose 3-O-Ethers as Models Representing the α-Ether Type Linkage between Lignin and Carbohydrates. *Chem. Zvesti* **1978**, *32* (3), 420–424.

(19) Nitz, M.; Bundle, D. R. Synthesis of Di- to Hexasaccharide 1,2-Linked β-Mannopyranan Oligomers, a Terminal S-Linked Tetrasaccharide Congener and the Corresponding BSA Glycoconjugates. *J. Org. Chem.* **2001**, *66* (25), 8411–8423. https://doi.org/10.1021/jo010570x.

(20) Ernst, A.; Vasella, A. Oligosaccharide Analogues of Polysaccharides. Part 8. Orthogonally Protected Cellobiose-Derived Dialkynes. A Convenient Method for the Regioselective Bromo- and Protodegermylation of Trimethylgermyl- and Trimethylsilyl-protected Dialkynes. *Helv. Chim. Acta* **1996**, *79* (5), 1279–1294. https://doi.org/10.1002/hlca.19960790505.

(21) Csuk, R.; Prell, E.; Korb, C.; Kluge, R.; Ströhl, D. Total Synthesis of 3,3-Difluorinated 1-Deoxynojirimycin Analogues. *Tetrahedron* **2010**, *66* (2), 467–472. https://doi.org/10.1016/j.tet.2009.11.069.

(22) Garneau, S.; Qiao, L.; Chen, L.; Walker, S.; Vederas, J. C. Synthesis of Mono- and Disaccharide Analogs of Moenomycin and Lipid II for Inhibition of Transglycosylase Activity of Penicillin-Binding Protein 1b. *Bioorganic Med. Chem.* **2004**, *12* (24), 6473–6494. https://doi.org/10.1016/j.bmc.2004.09.019.

(23) Mani, N. S.; Kanakamma, P. P. Synthesis of Novel Chiral Macrocycles: Crown Ethers Derived from D-Glucose. *Tetrahedron Lett.* **1994**, *35* (21), 3629–3632. https://doi.org/10.1016/S0040-4039(00)73258-8.

(24) Qiao, L.; Vederad, J. C. Synthesis of a C-Phosphonate Disaccharide as a Potential Inhibitor of Peptidoglycan Polymerization by Transglycosylase. *J. Org. Chem.* **1993**, *58*, 3480–3482. https://doi.org/10.1021/jo00065a004.

(25) Fylaktakidou, K. C.; Duarte, C. D.; Koumbis, A. E.; Nicolau, C.; Lehn, J. M. Polyphosphates and Pyrophosphates of Hexopyranoses as Allosteric Effectors of Human Hemoglobin: Synthesis, Molecular Recognition, and Effect on Oxygen Release. *ChemMedChem* **2011**, *6*, 153–168. https://doi.org/10.1002/cmdc.201000366.

(29): ^{1}H NMR (500 MHz, $CDCl_3$)

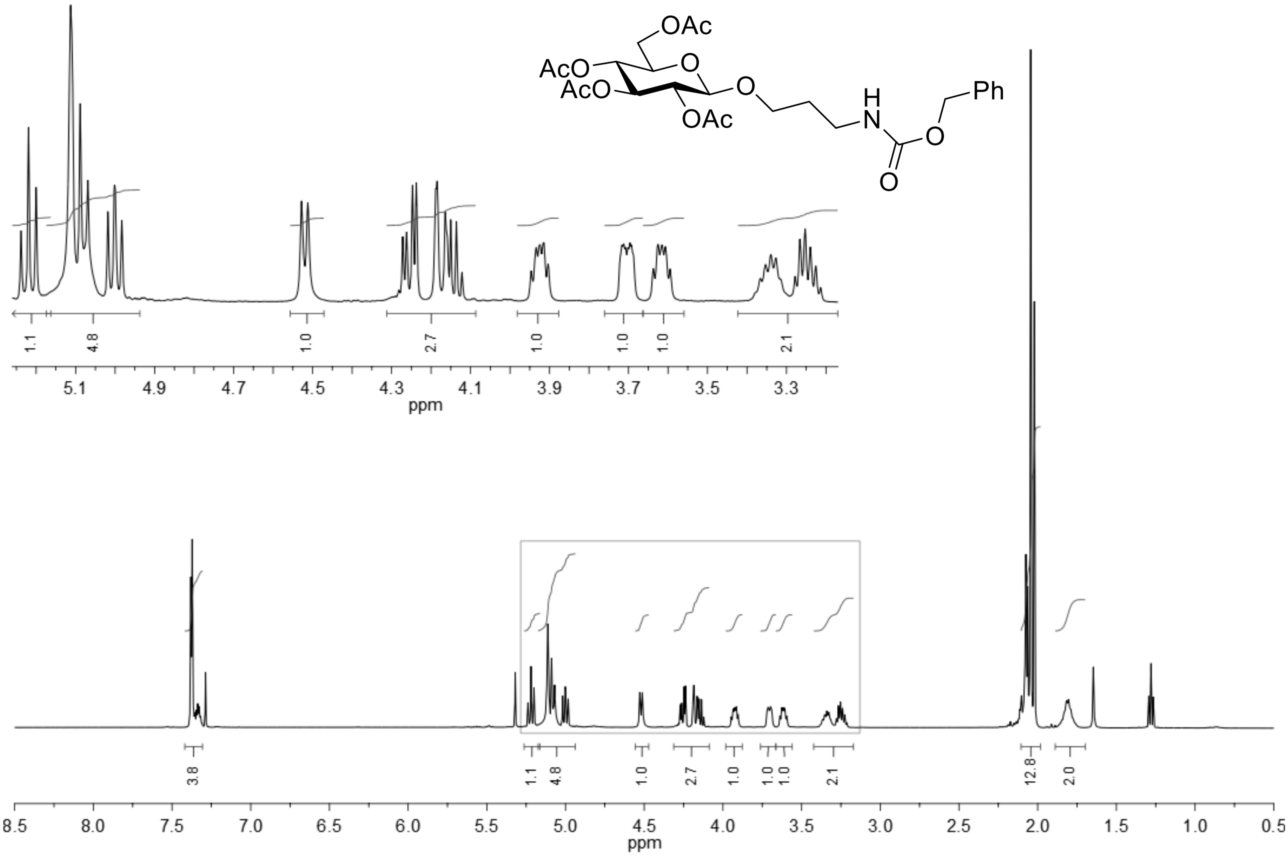

(30): ^{1}H NMR (500 MHz, CD_3OD)

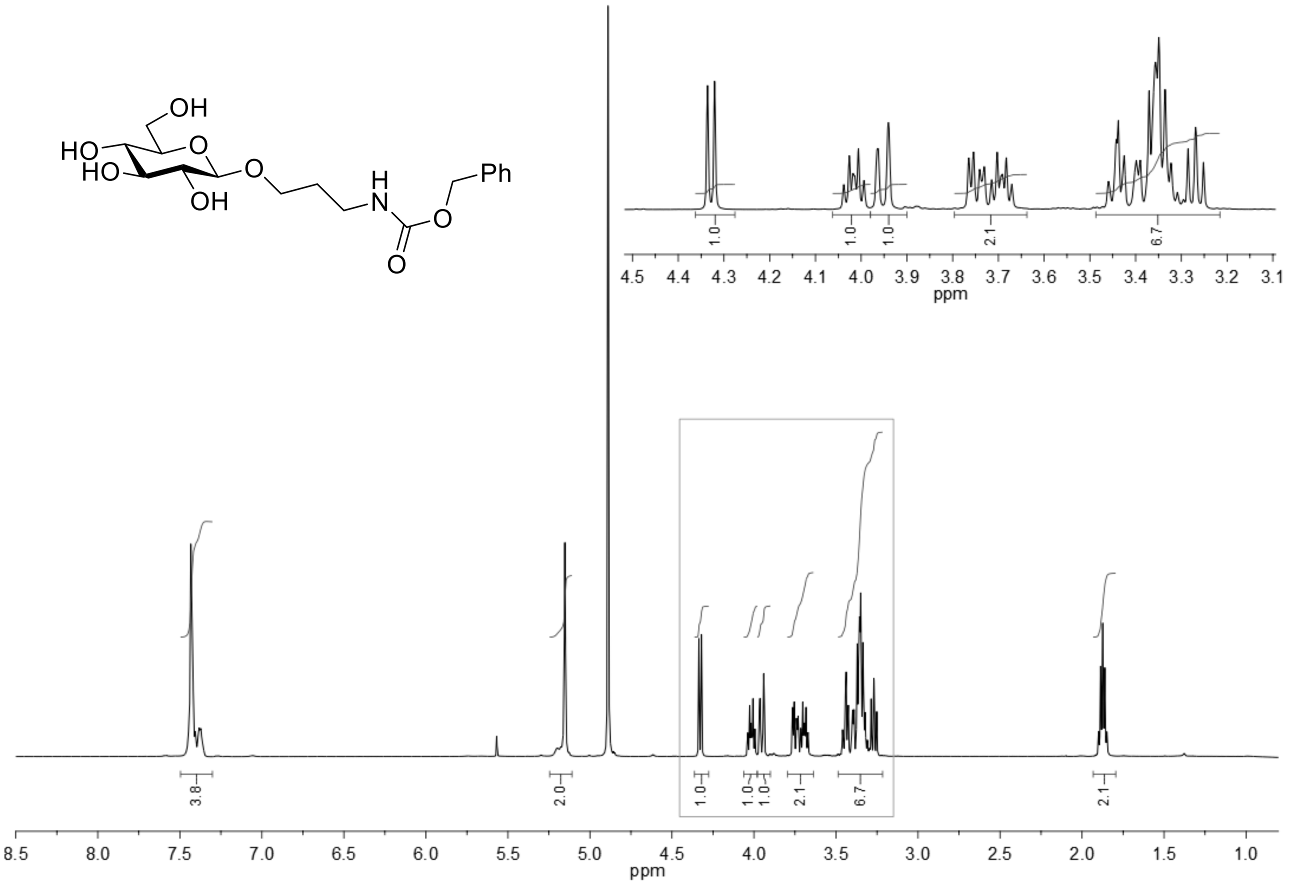

(31): ^{1}H NMR (500 MHz, CD_3OD)

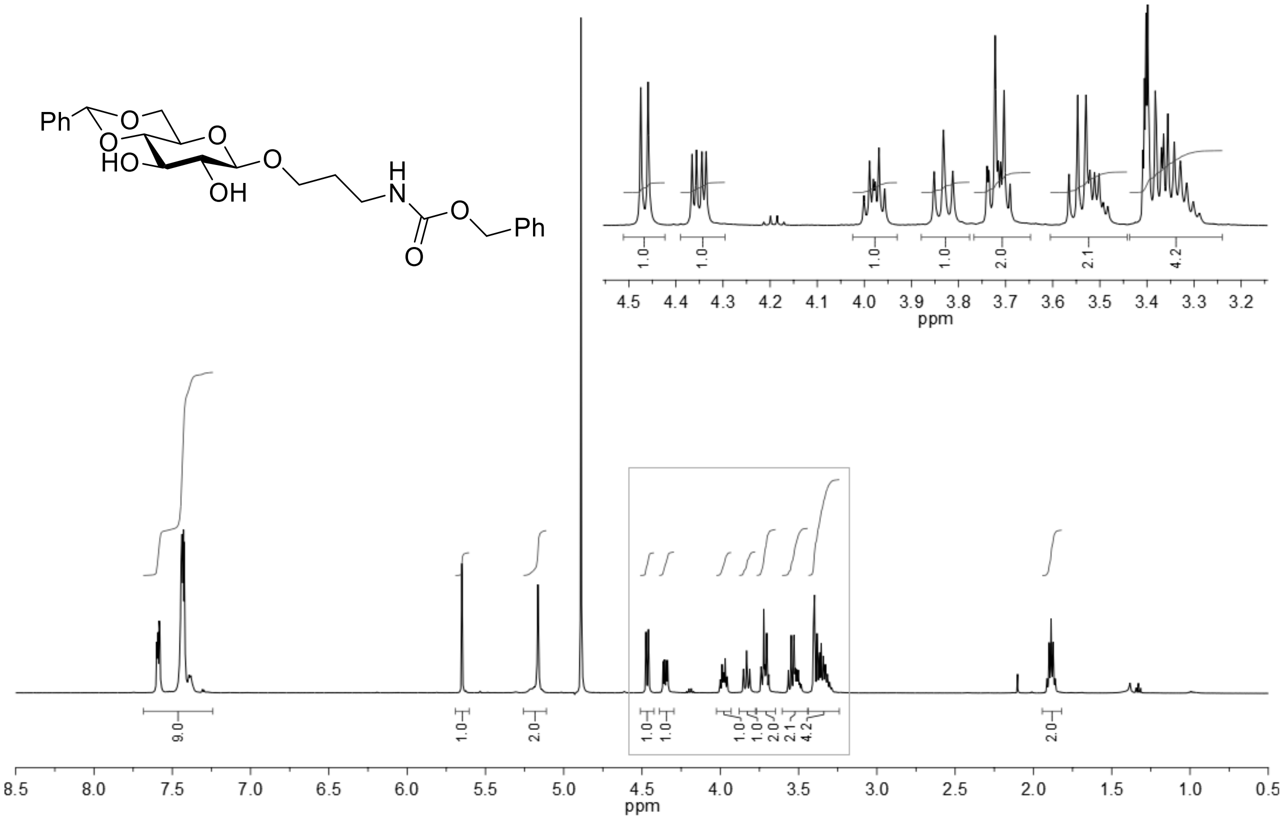

(32): ^{1}H NMR (500 MHz, $CDCl_3$)

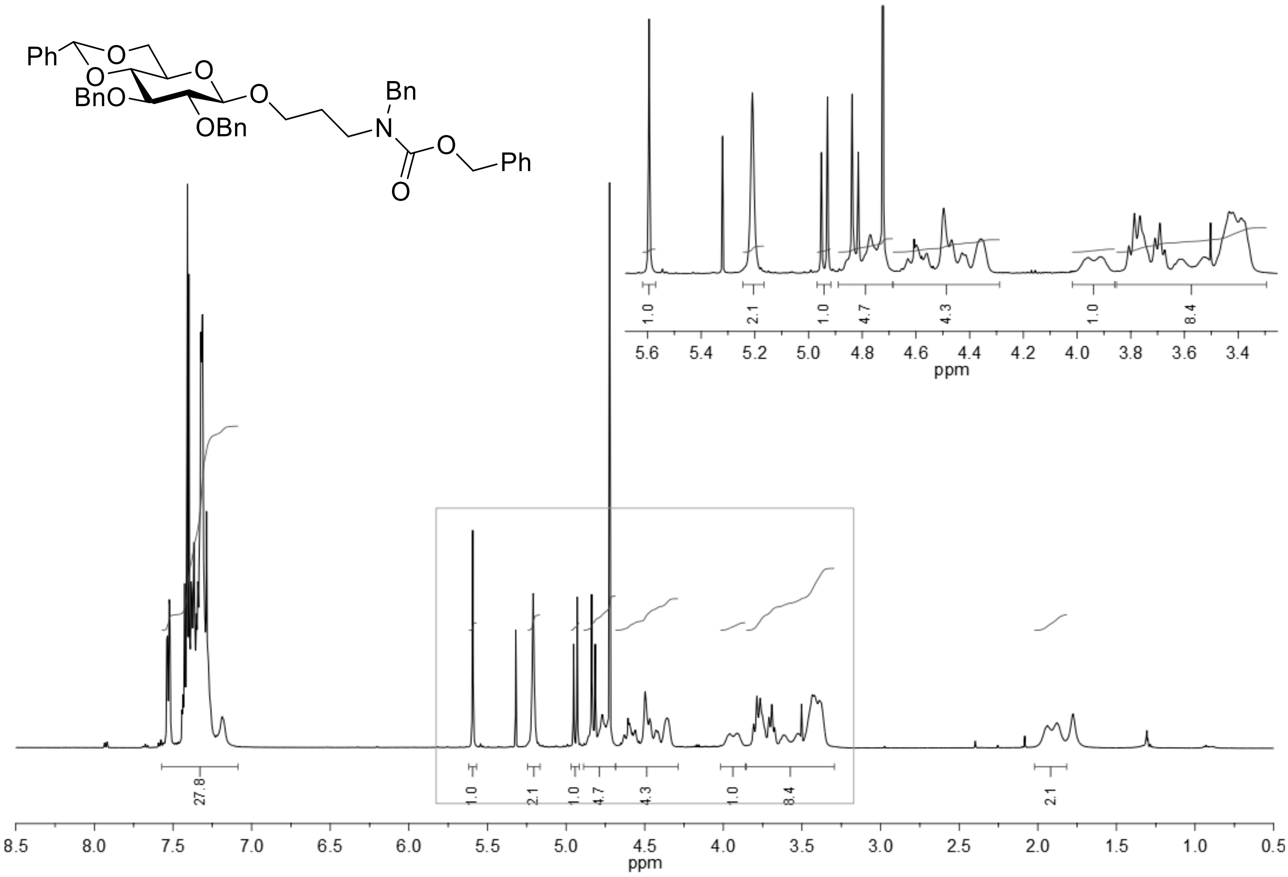

(7): ^{1}H NMR (500 MHz, CDCl$_3$)

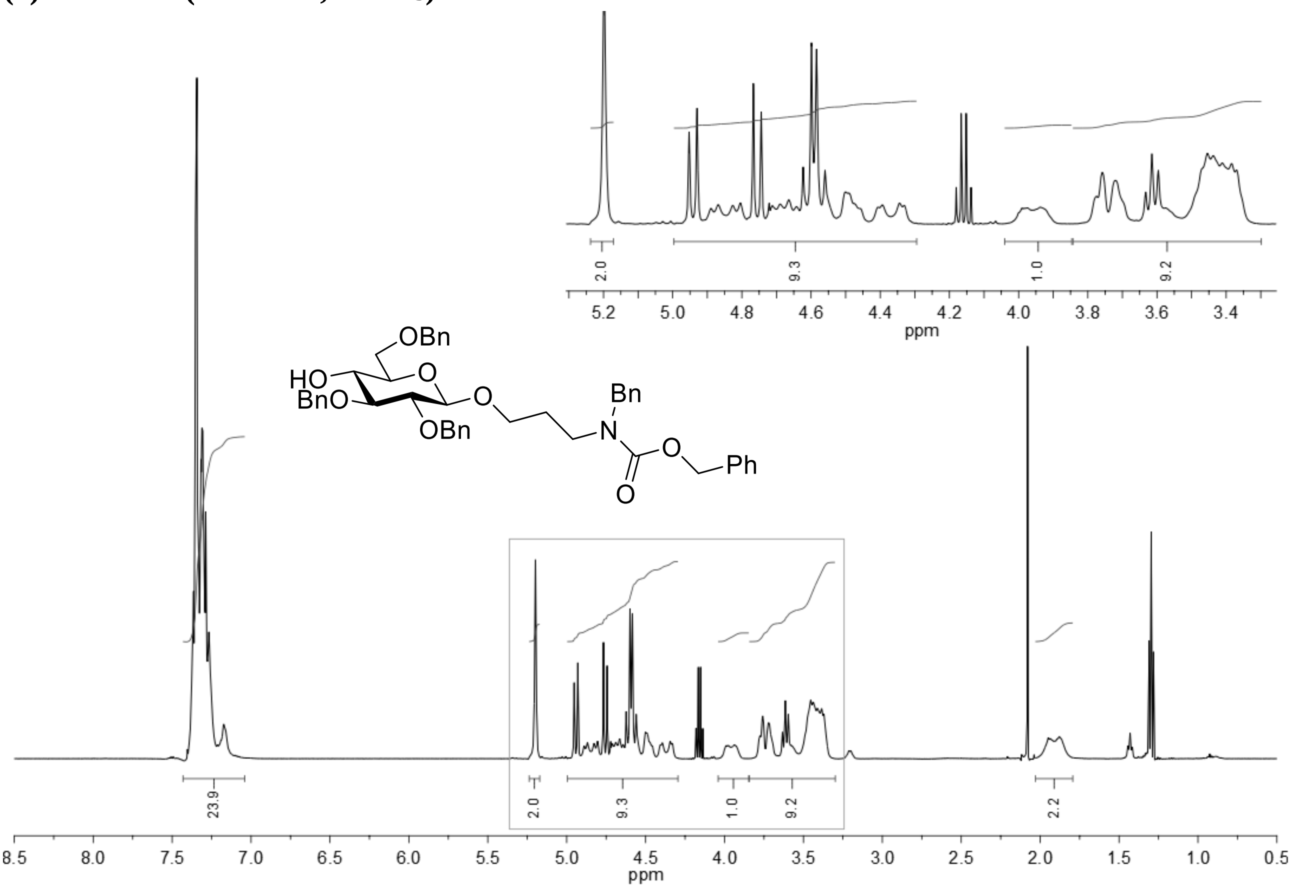

(34): ^{1}H NMR (500 MHz, CDCl$_3$)

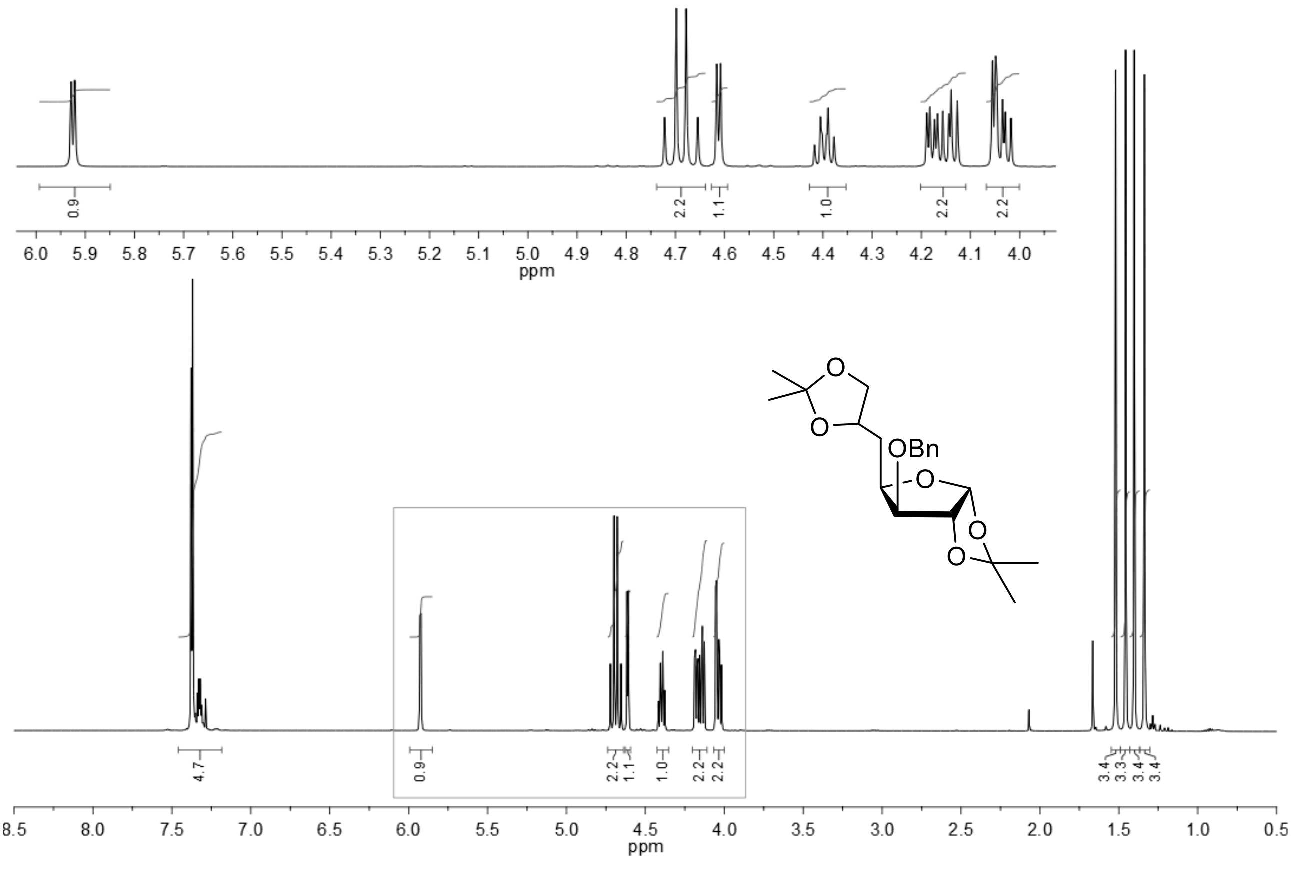

(35): ^{1}H NMR (500 MHz, CD_3OD)

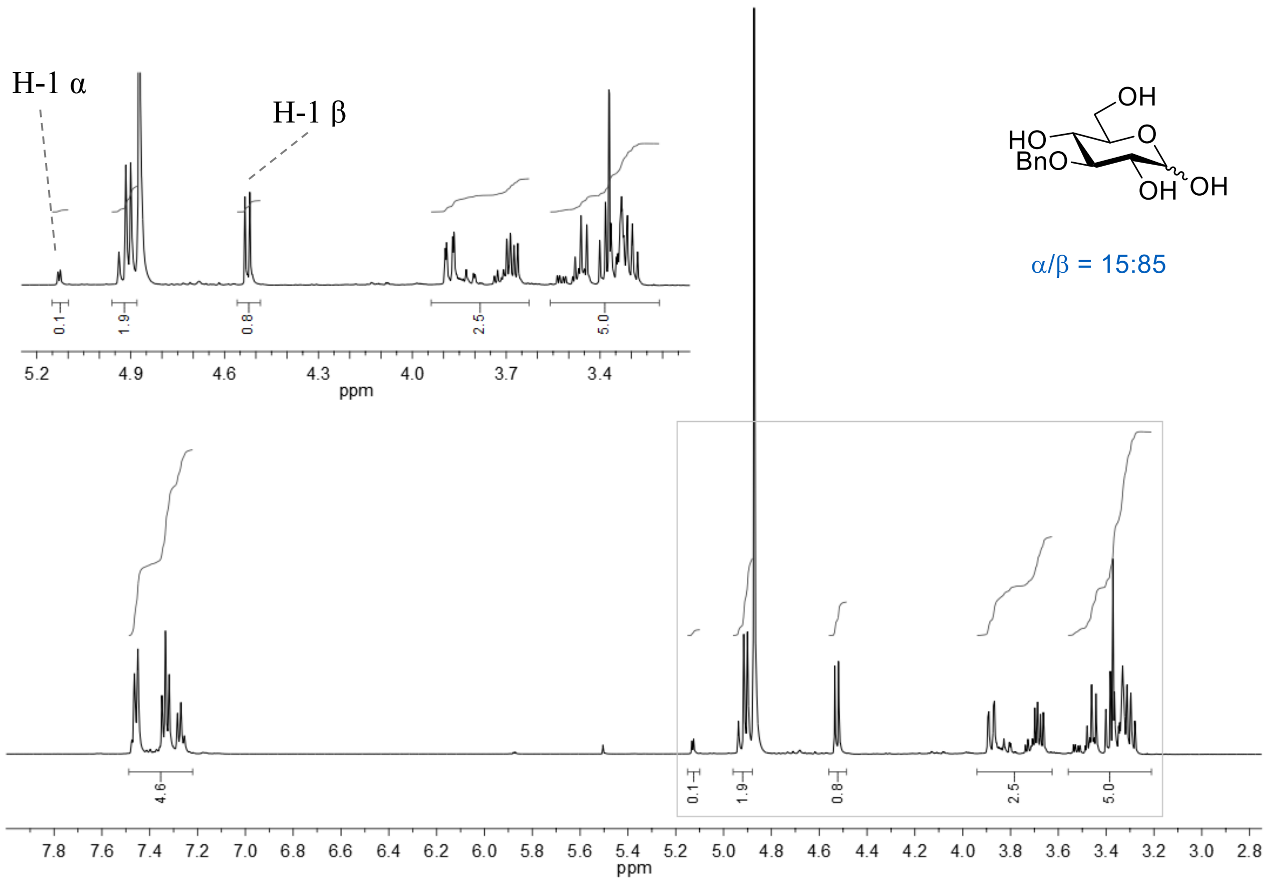

(36): ^{1}H NMR (500 MHz, $CDCl_3$)

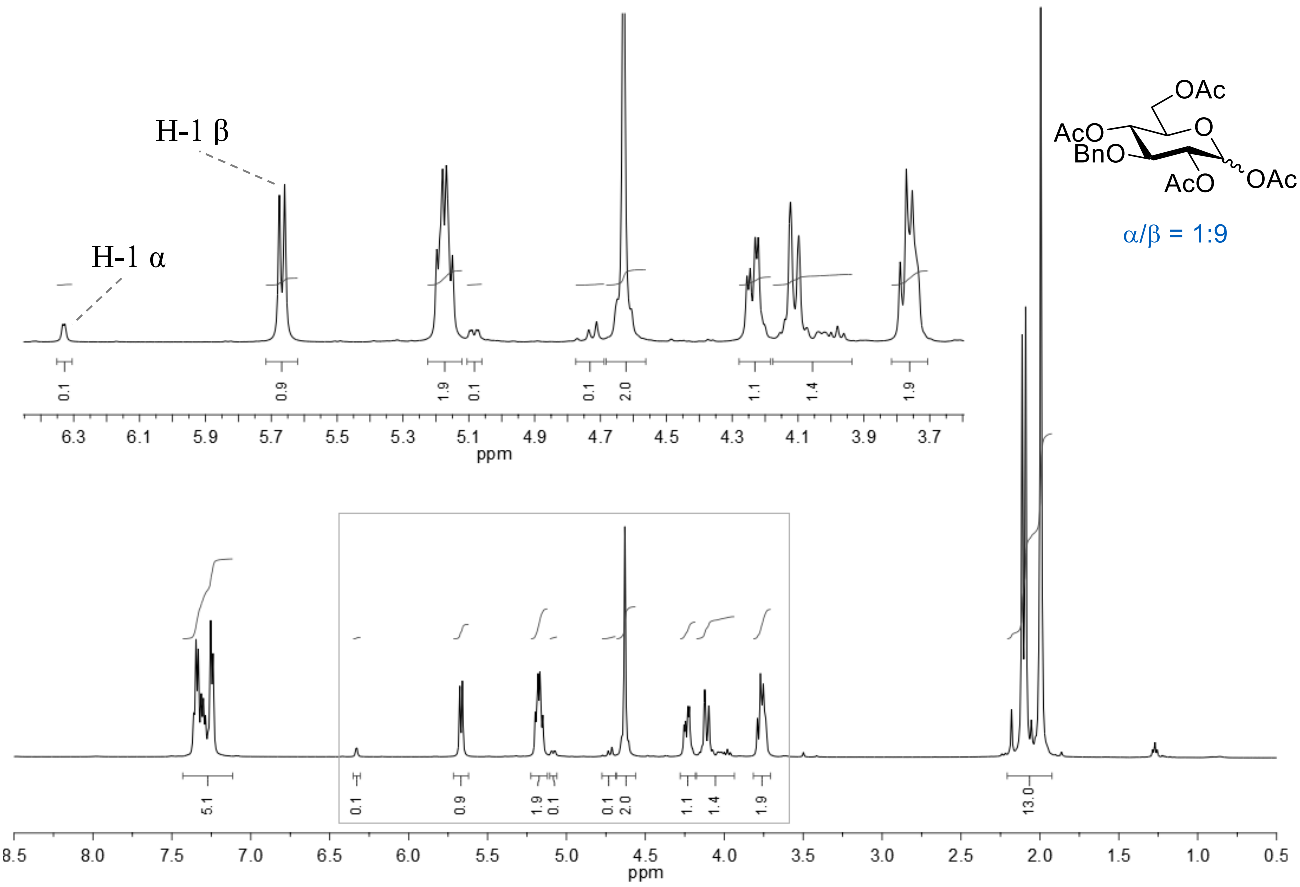

(52): ^{1}H NMR (500 MHz, CDCl$_3$)

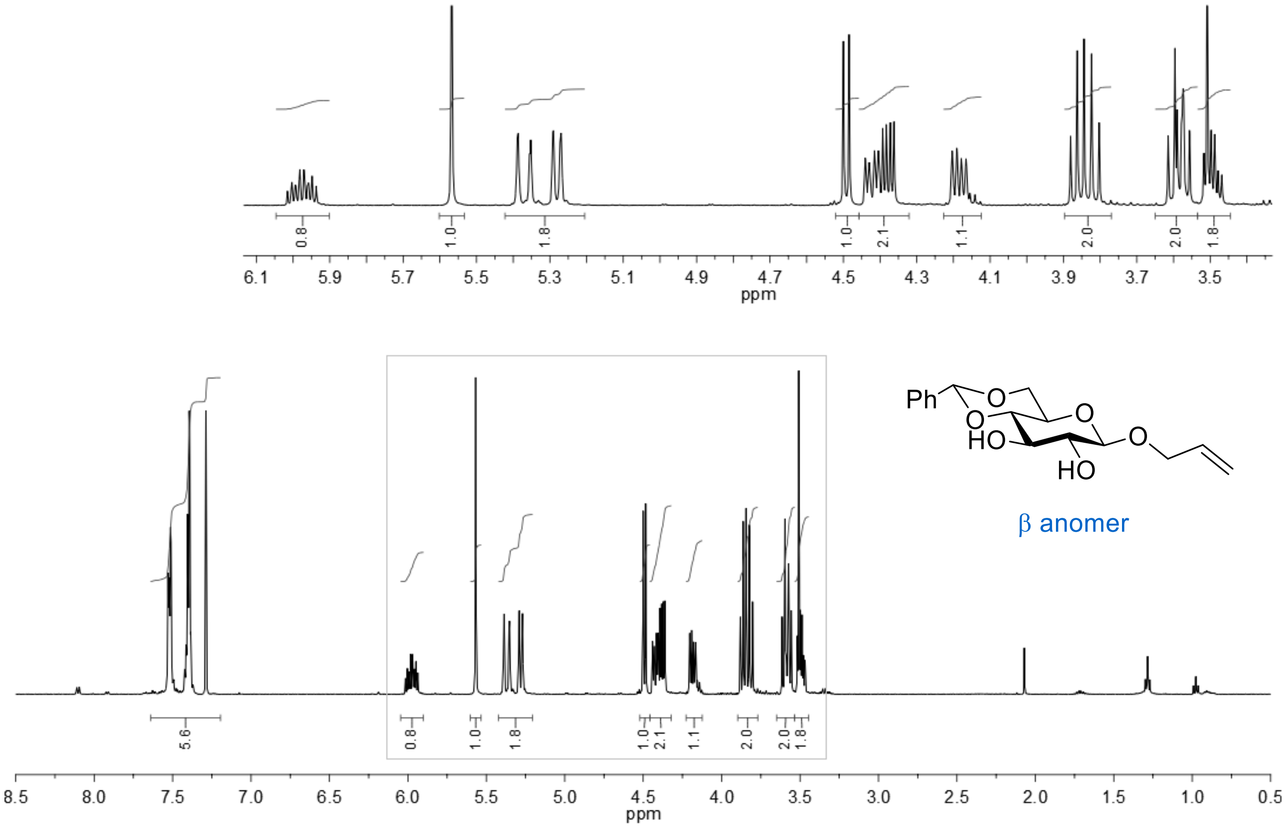

(53): ^{1}H NMR (500 MHz, CDCl$_3$)

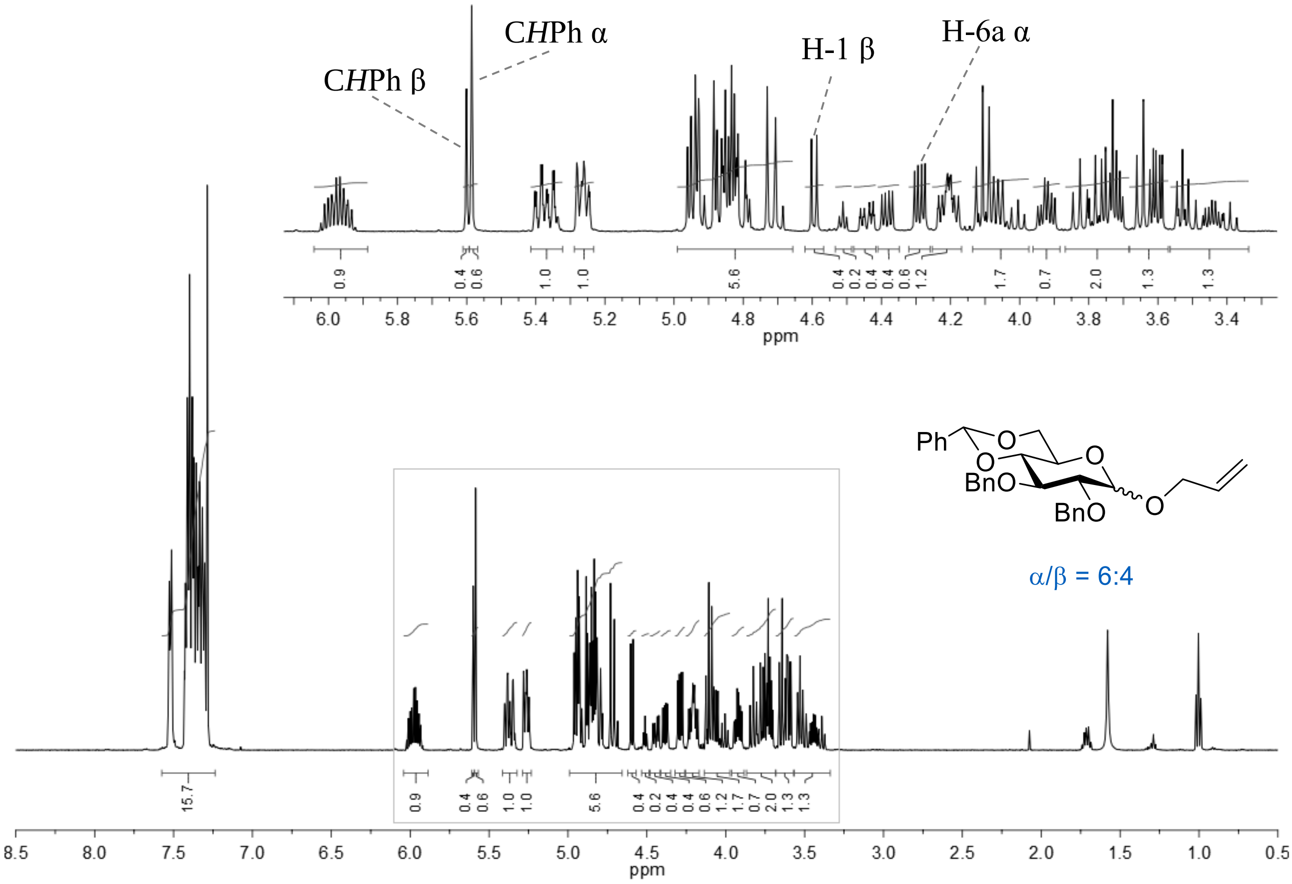

(43): ^{1}H NMR (500 MHz, CDCl₃)

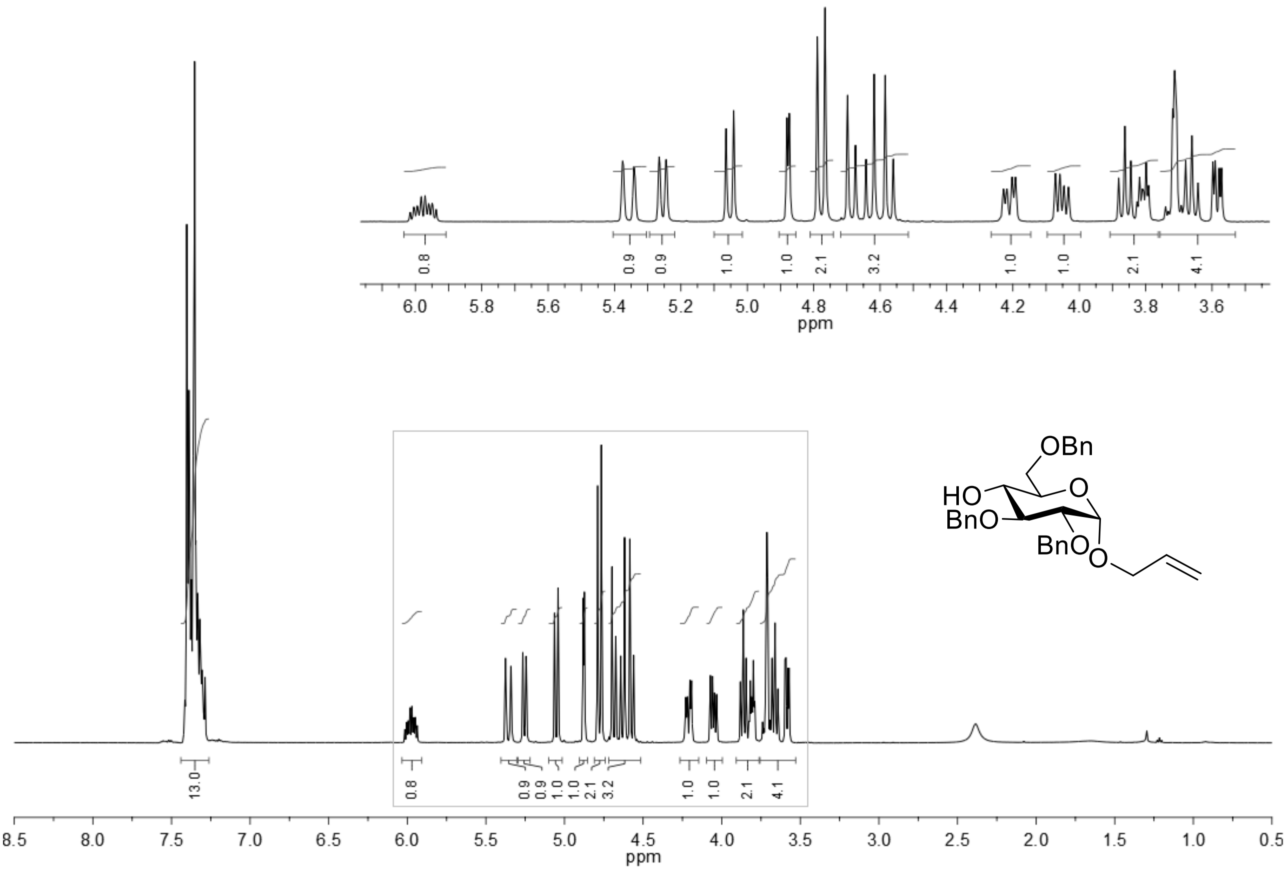

(55): ^{1}H NMR (500 MHz, CDCl₃)

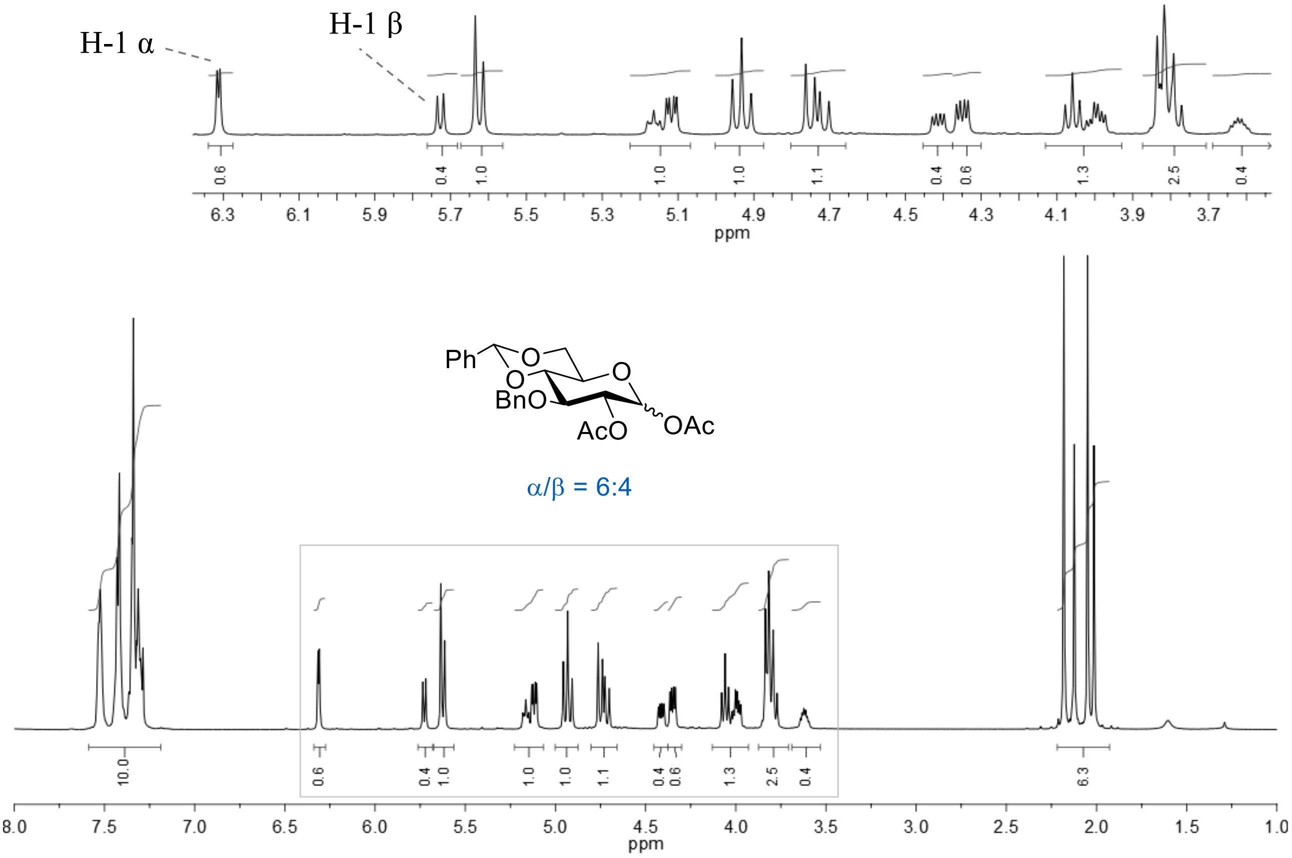

(56): ^{1}H NMR (500 MHz, CDCl$_3$)

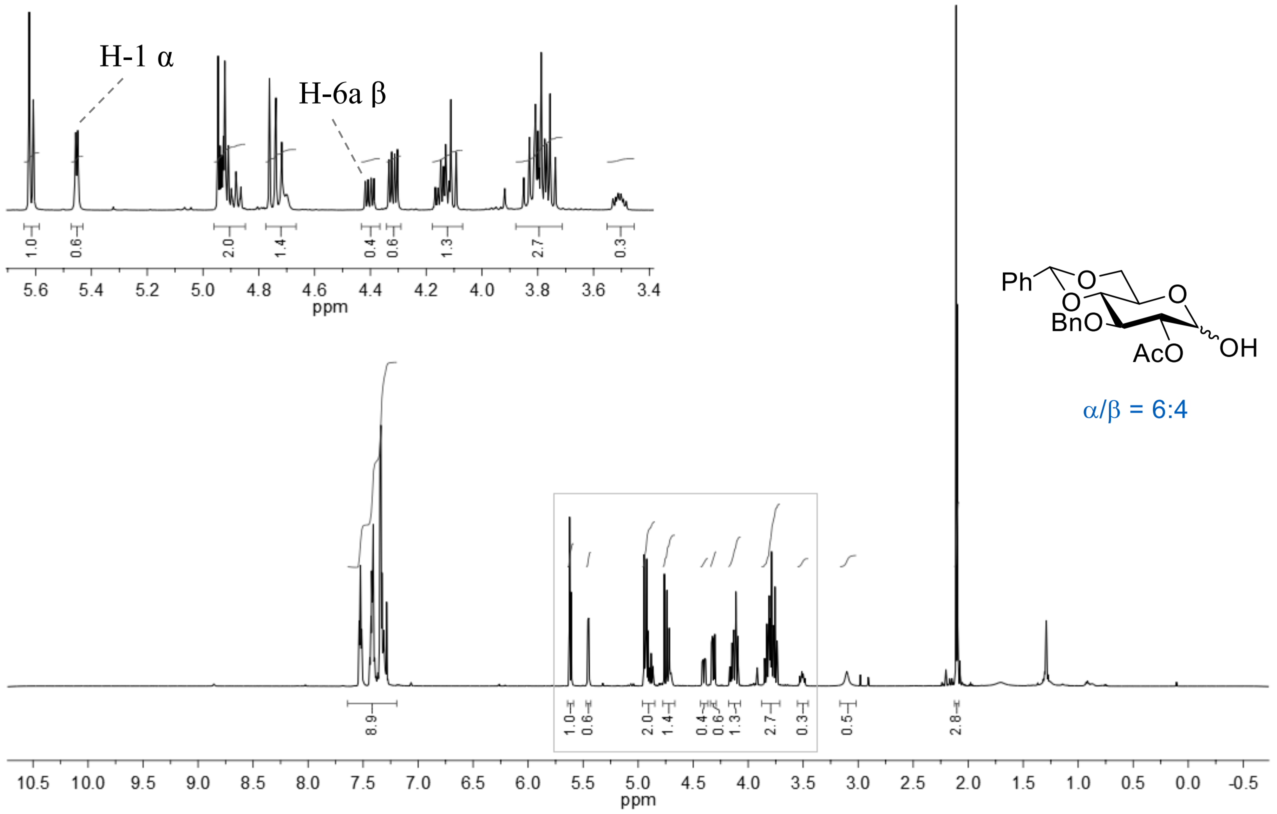

(13): ^{1}H NMR (500 MHz, CDCl$_3$)

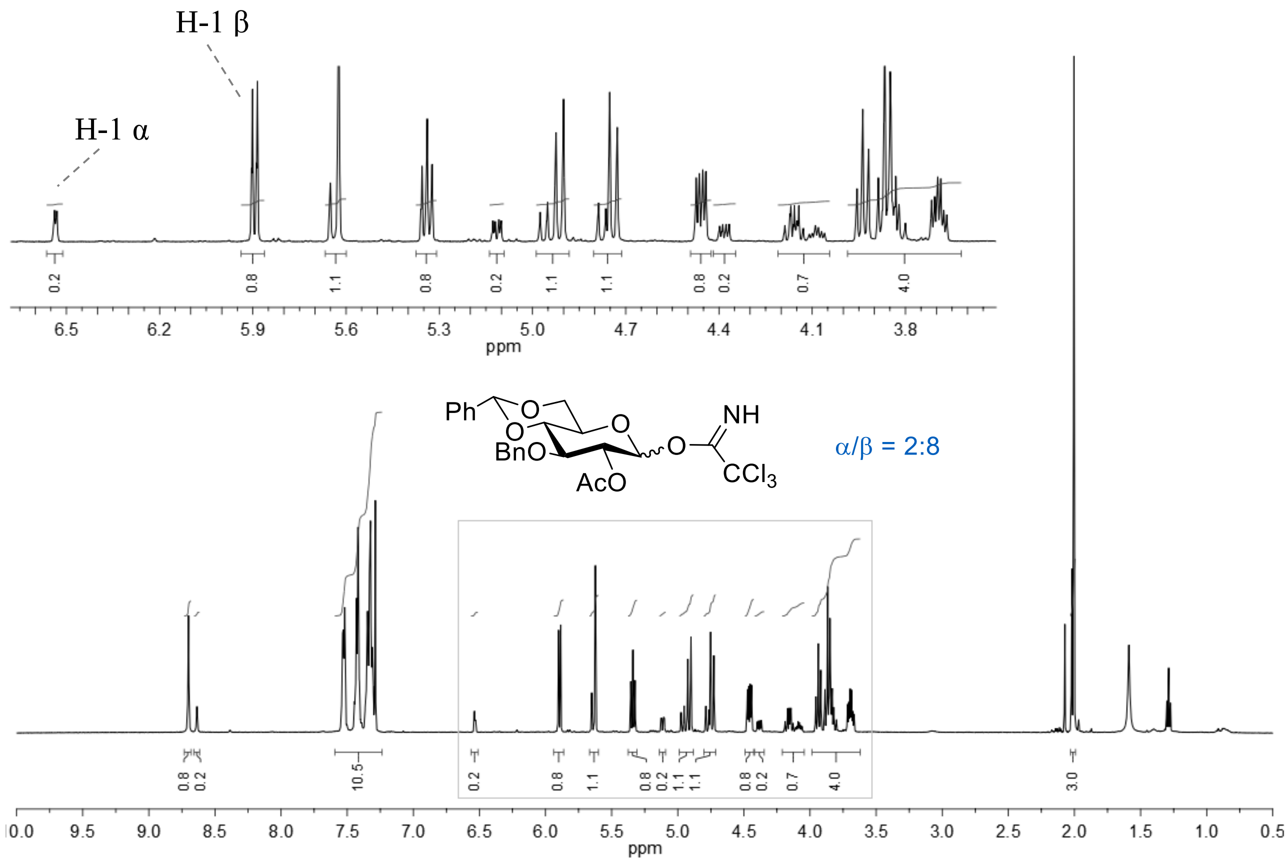

(37): ^{1}H NMR (500 MHz, $CDCl_3$)

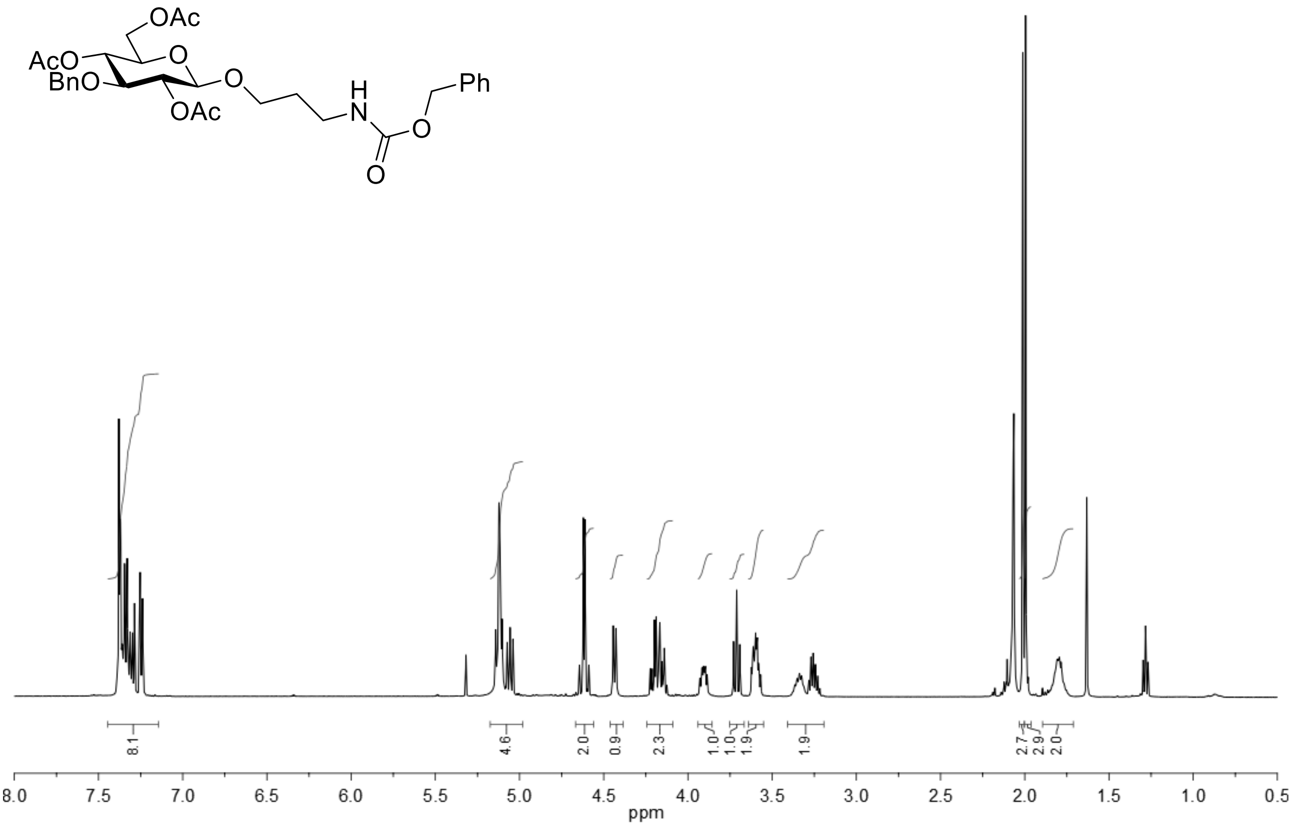

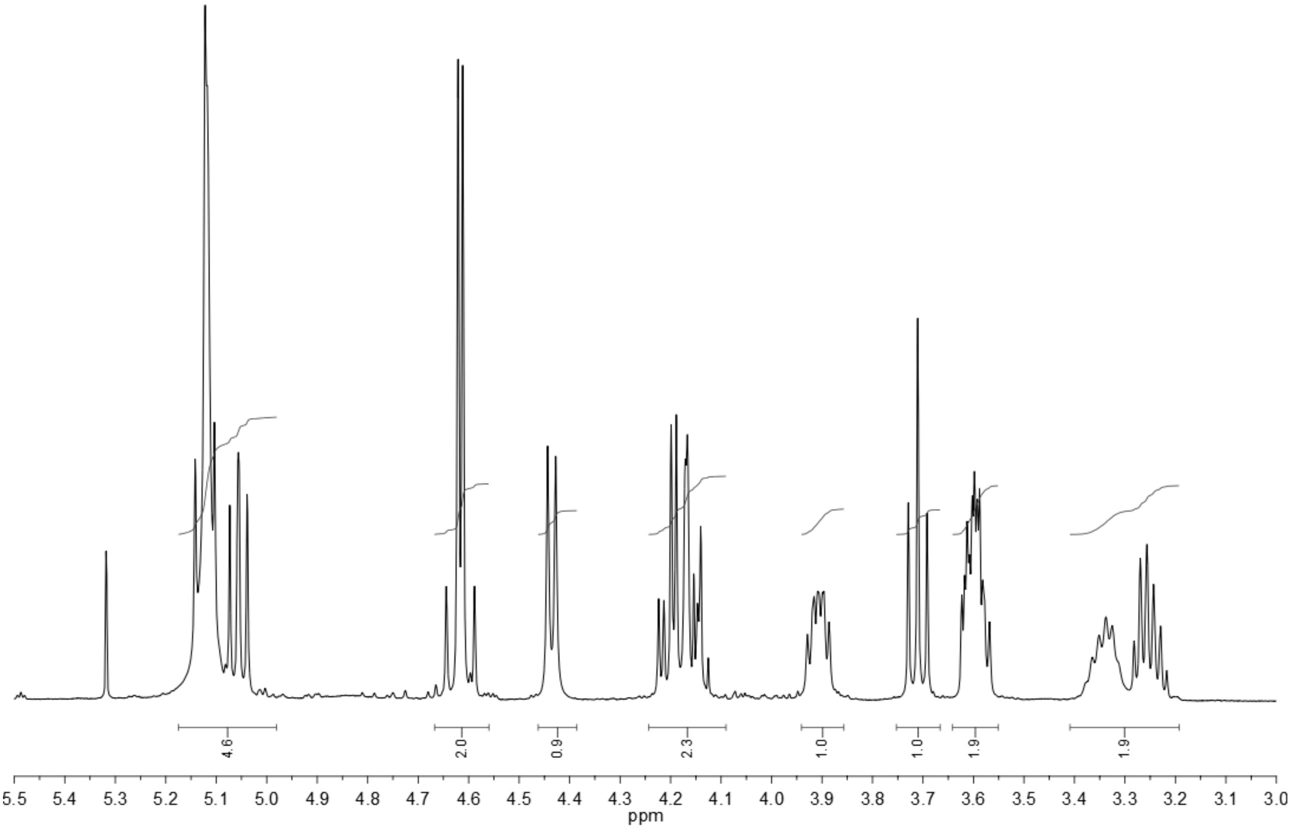

(37): ^{13}C NMR (126 MHz, $CDCl_3$)

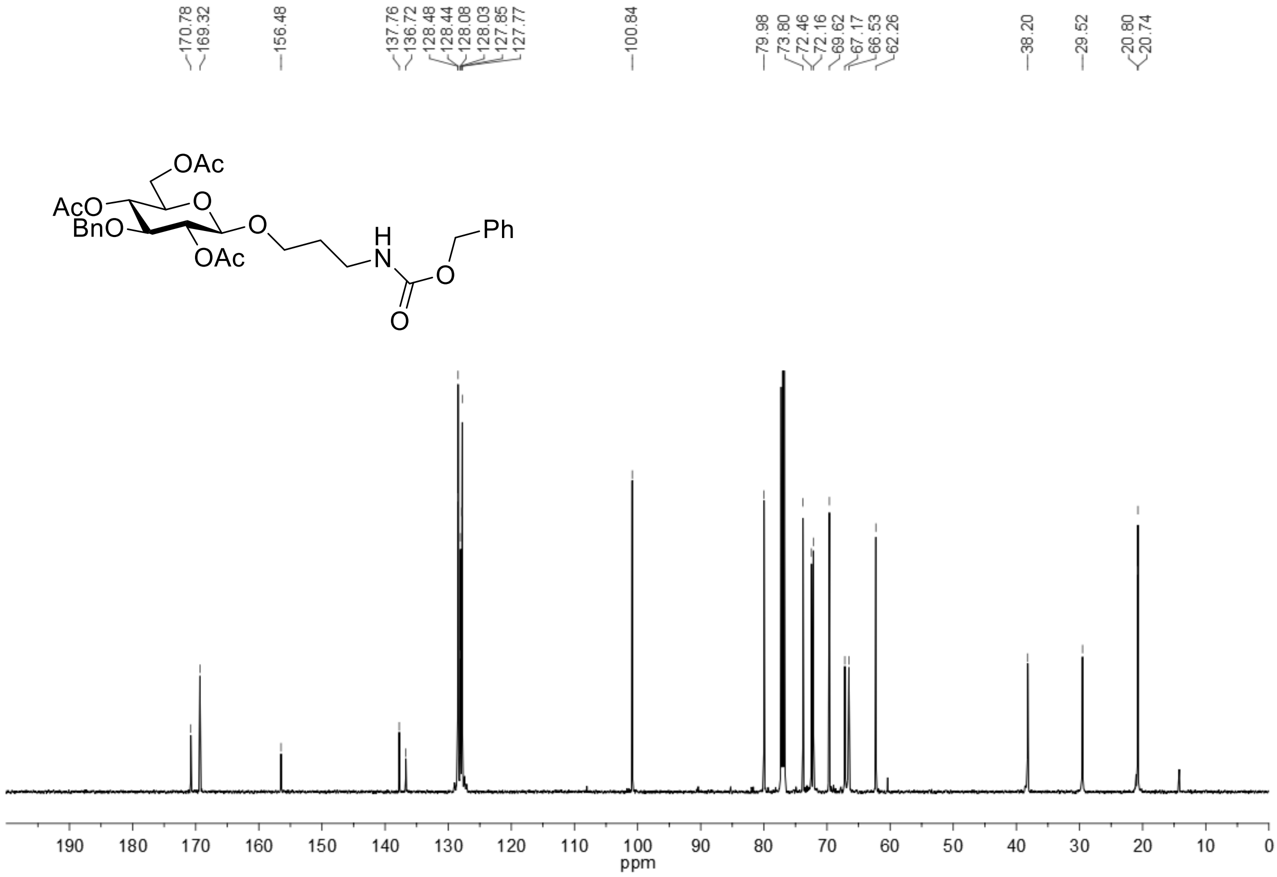

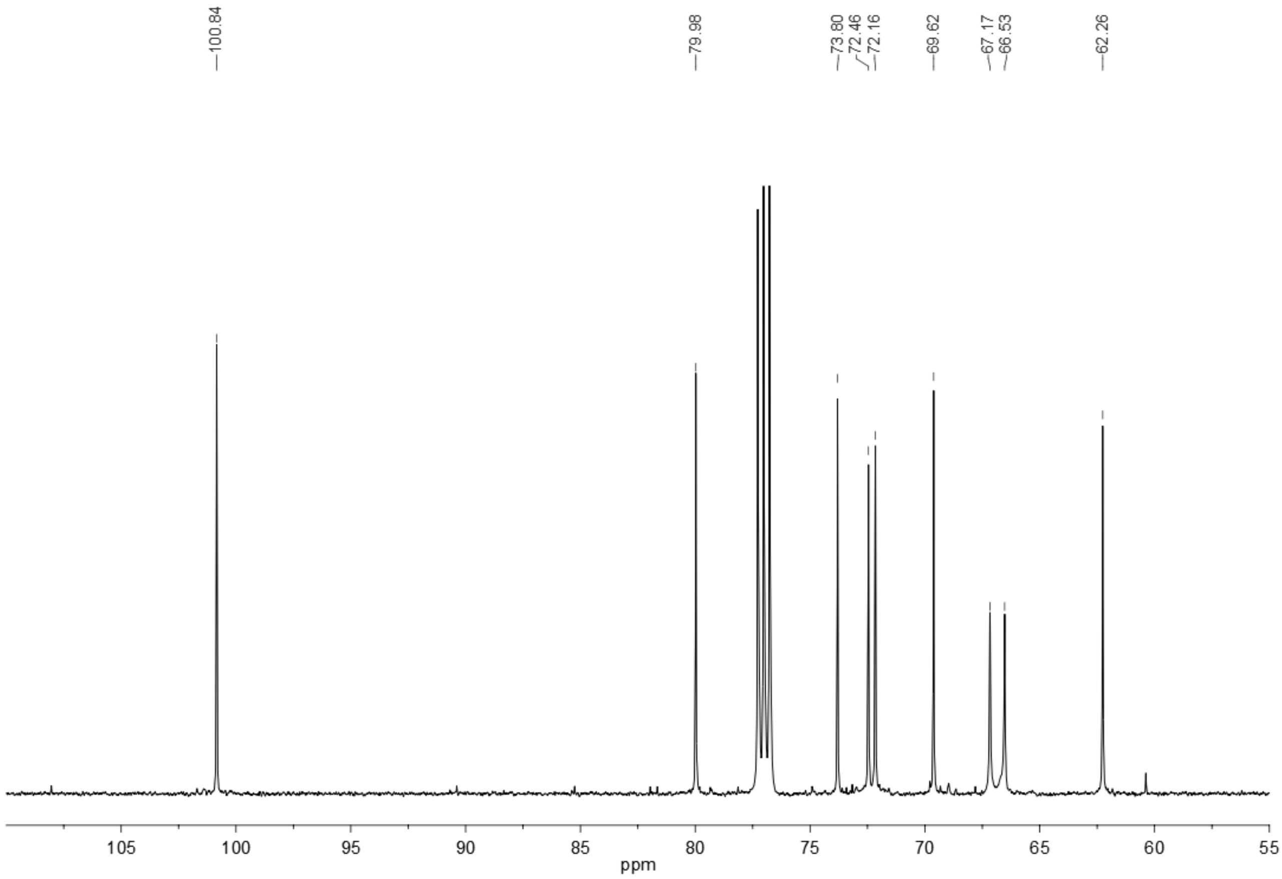

(38): ^{1}H NMR (500 MHz, CD_3OD)

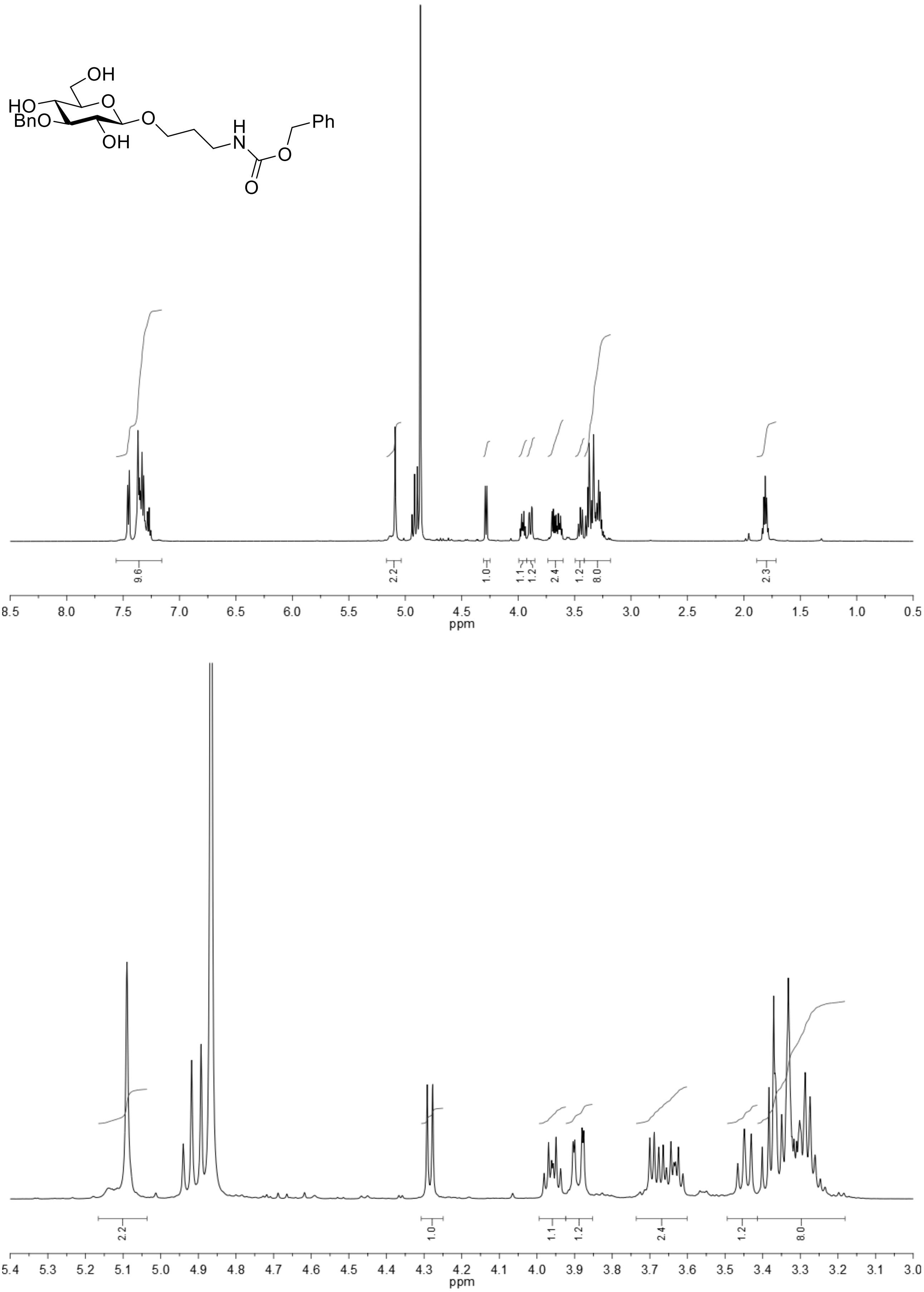

(38): ^{13}C NMR (126 MHz, CD_3OD)

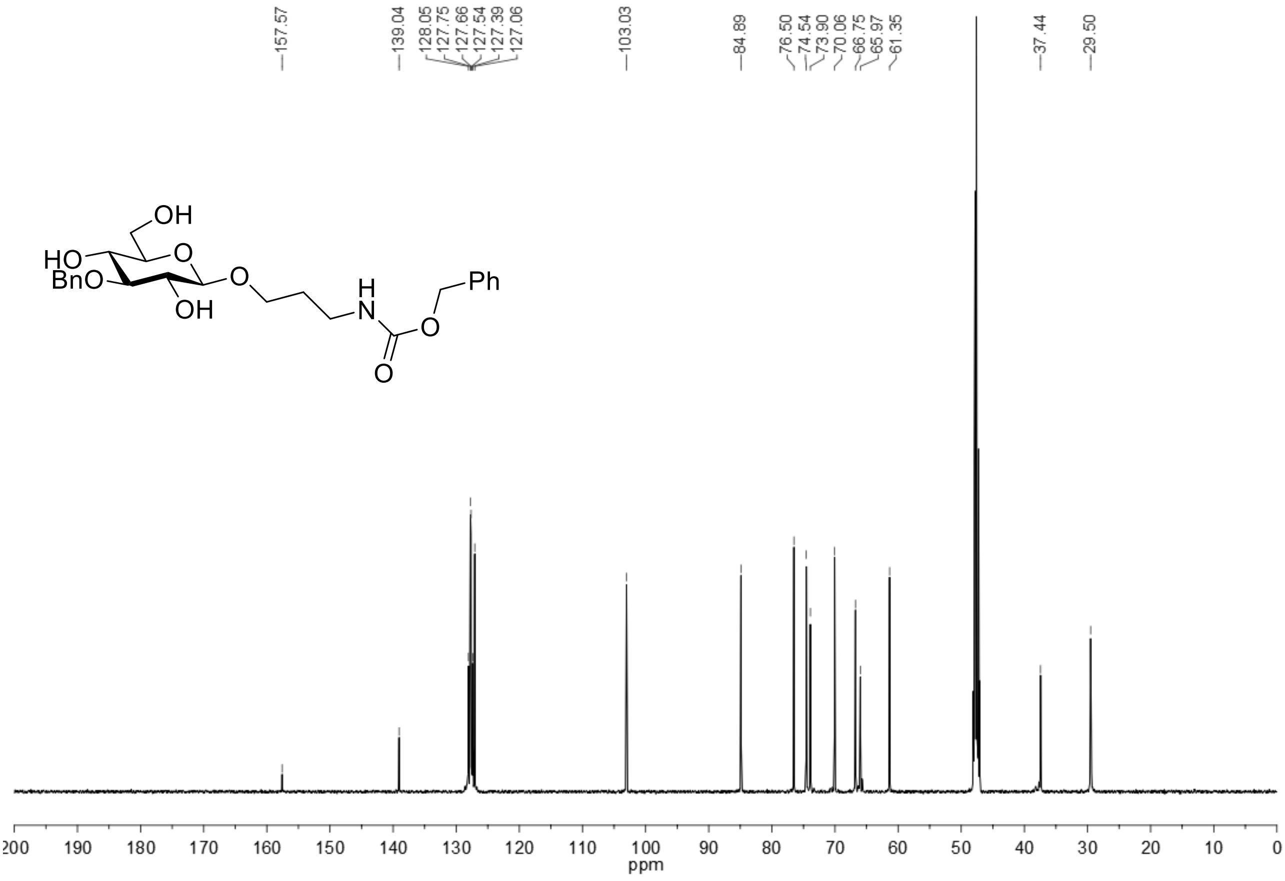

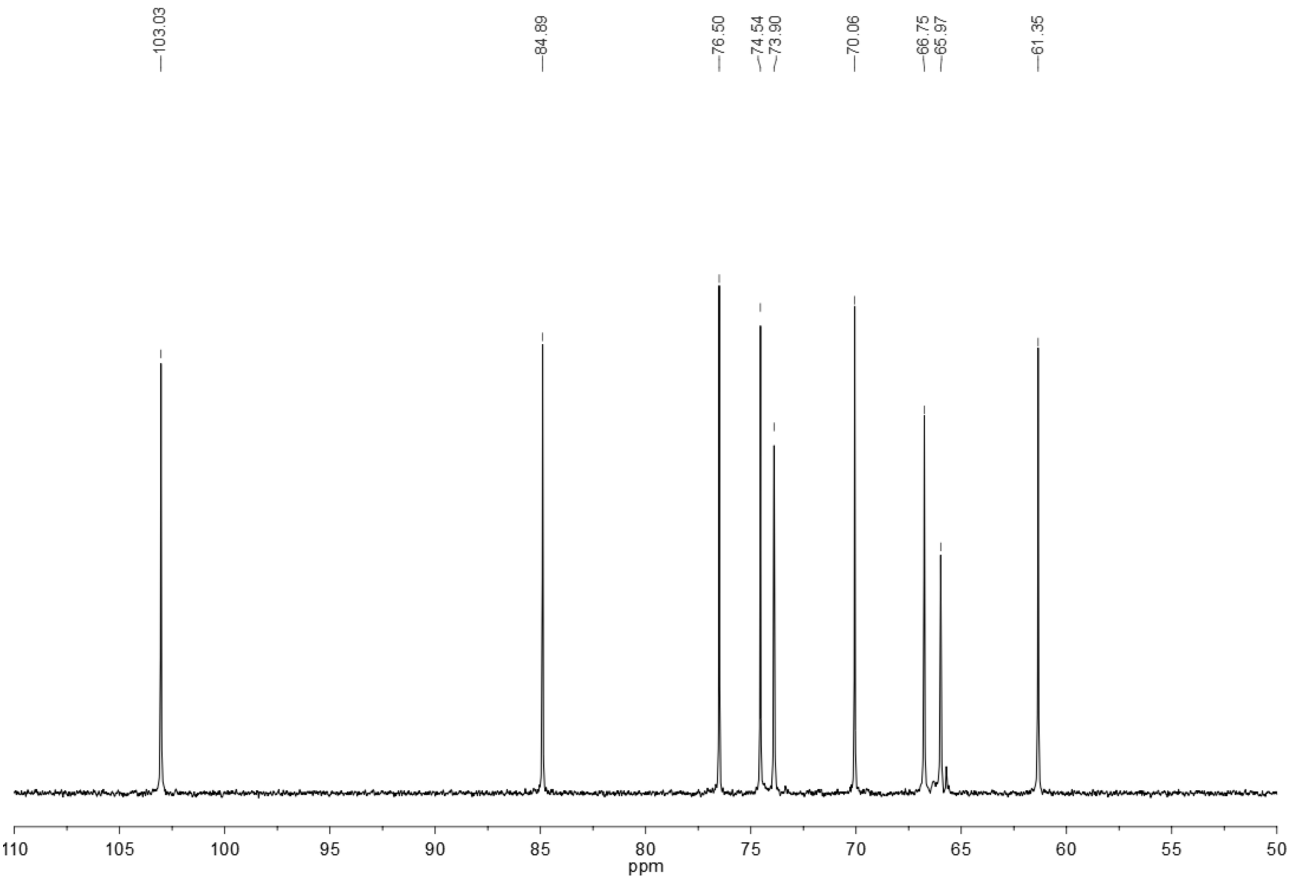

(39): ^{1}H NMR (500 MHz, $CDCl_3$)

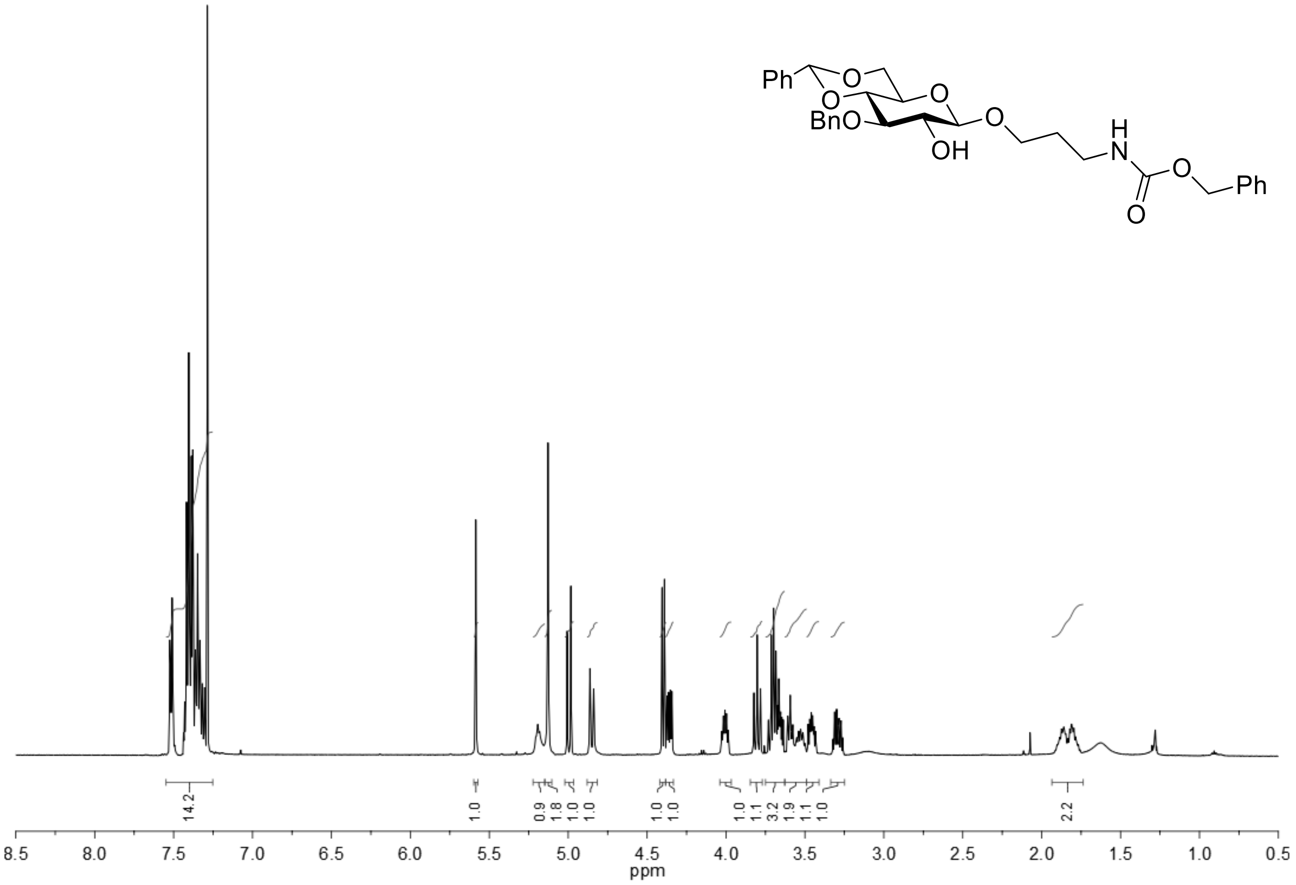

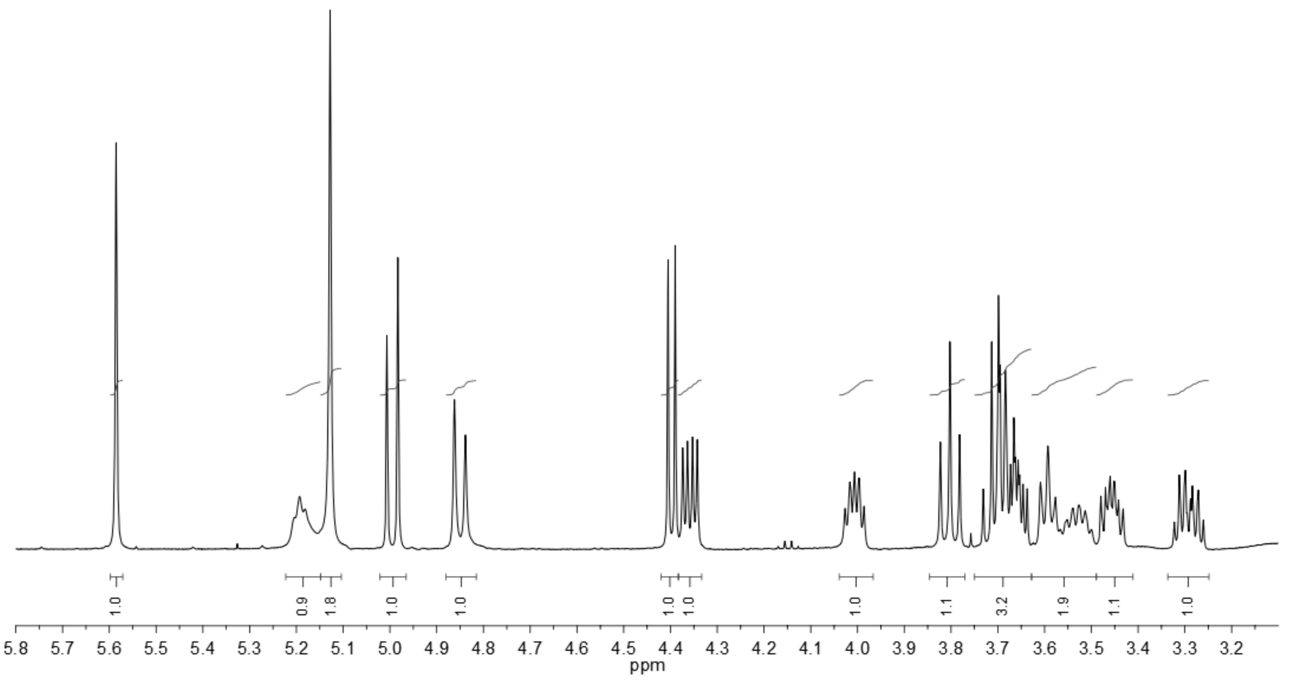

(39): ^{13}C NMR (126 MHz, $CDCl_3$)

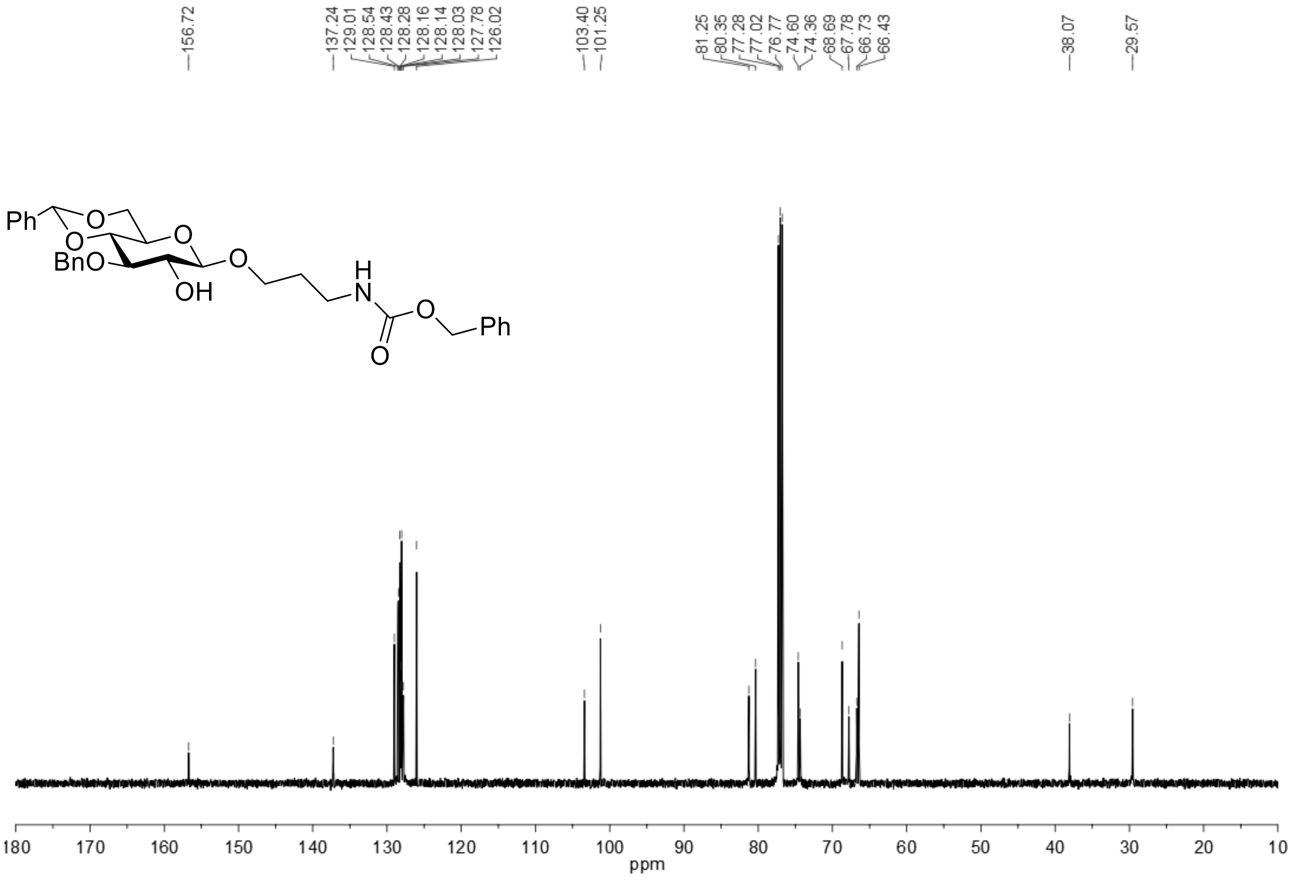

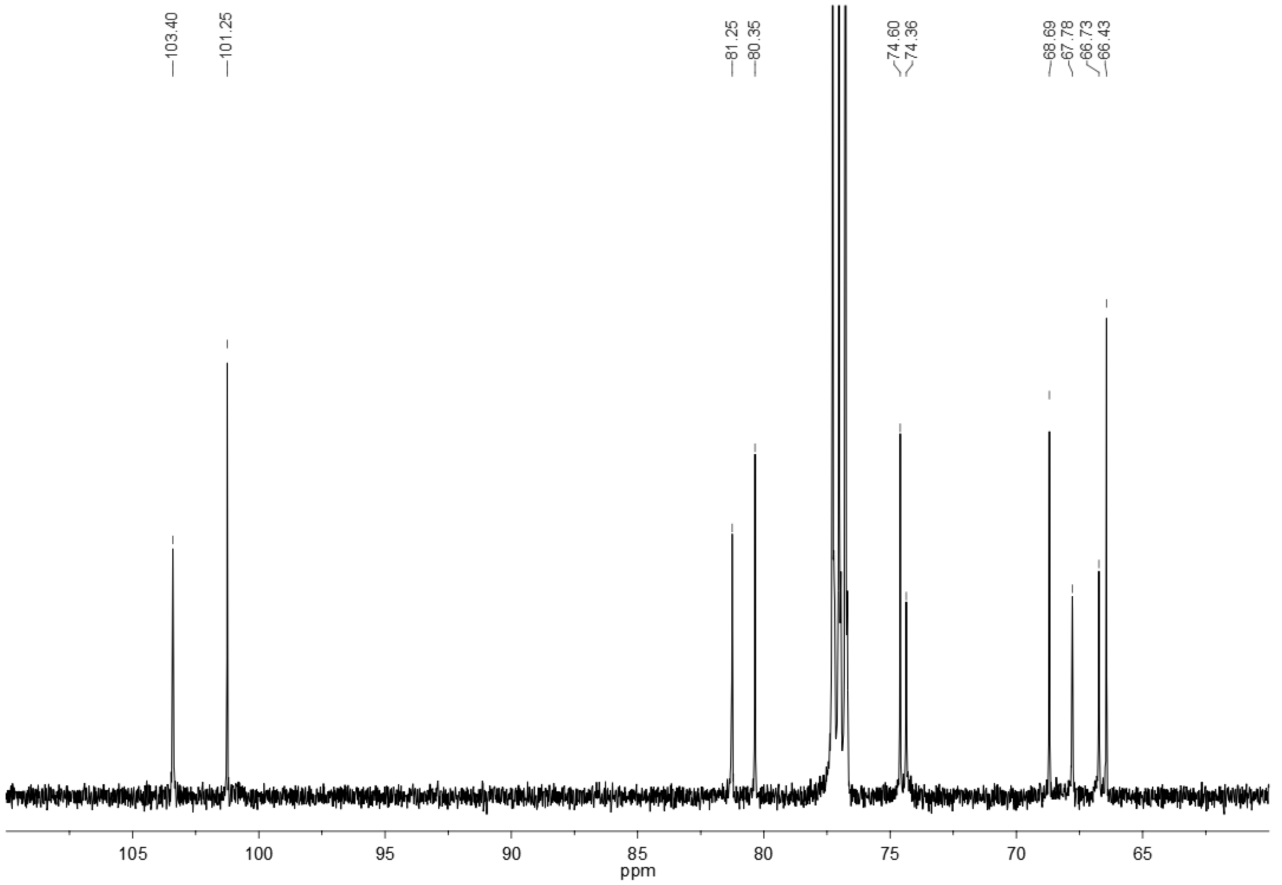

(40): ^{1}H NMR (500 MHz, $CDCl_3$)

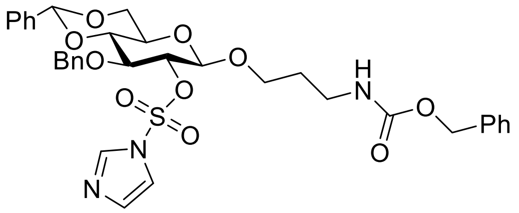

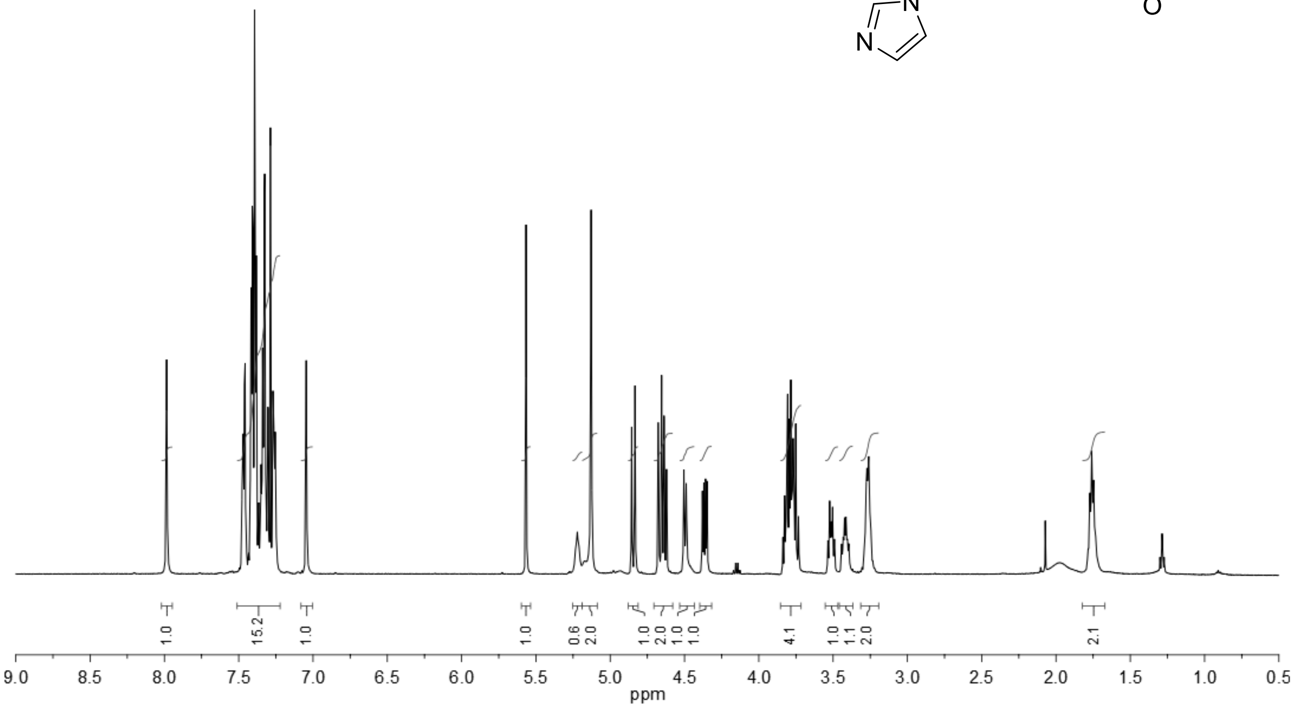

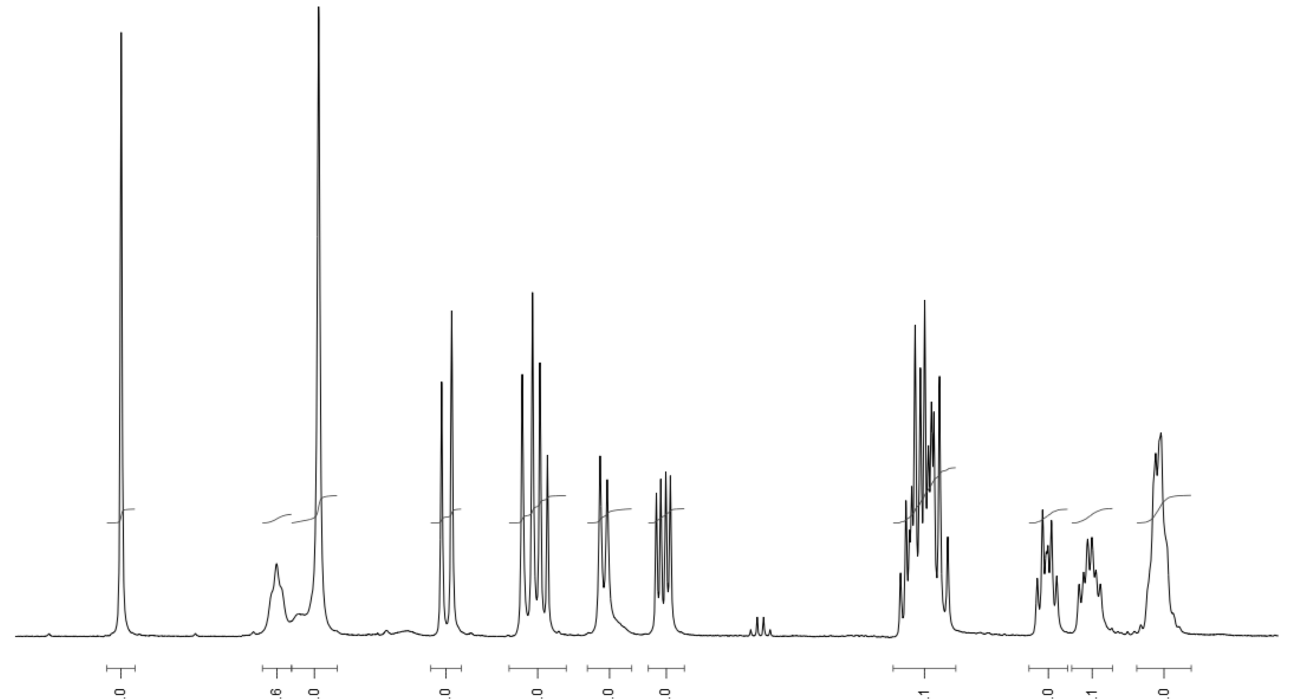

(40): ^{13}C NMR (126 MHz, $CDCl_3$)

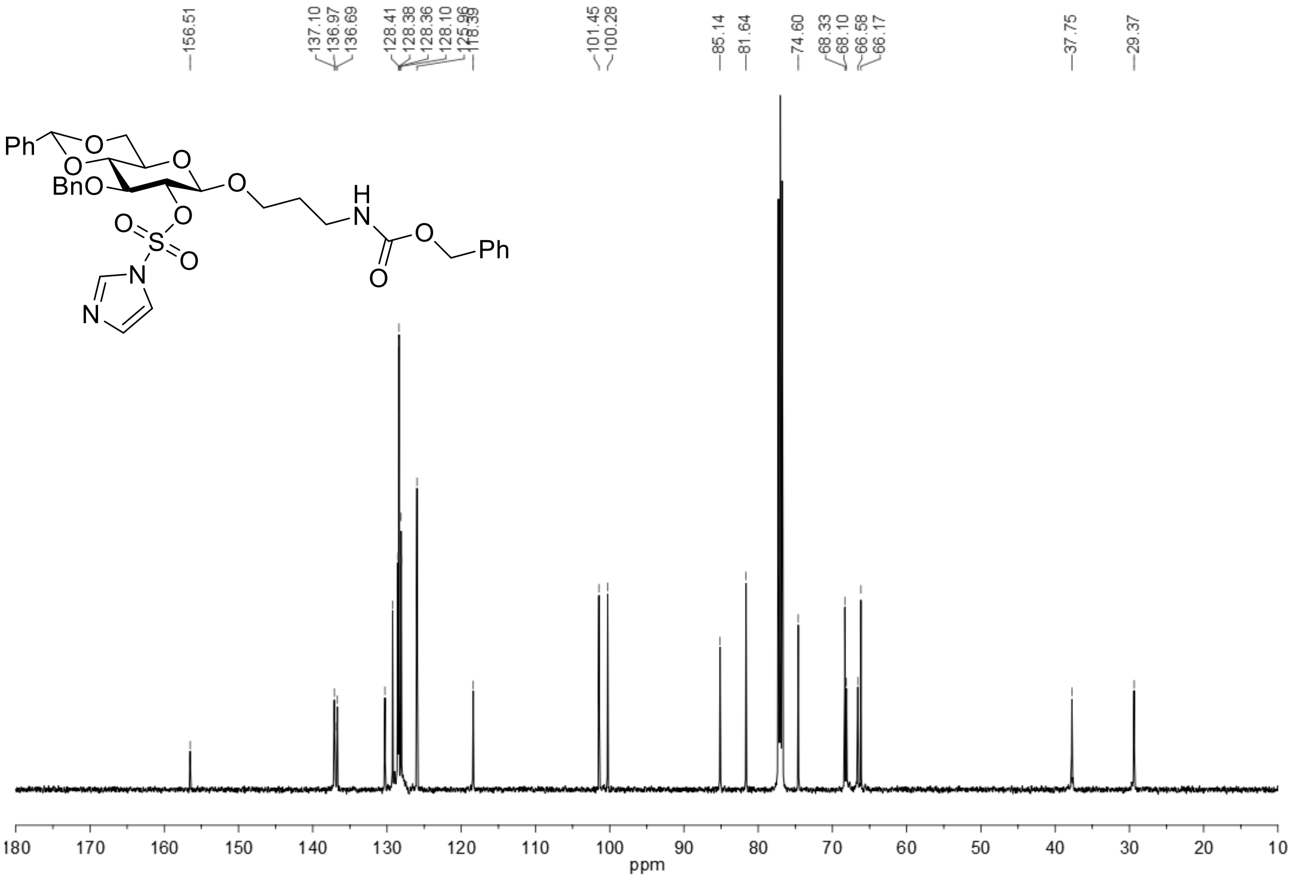

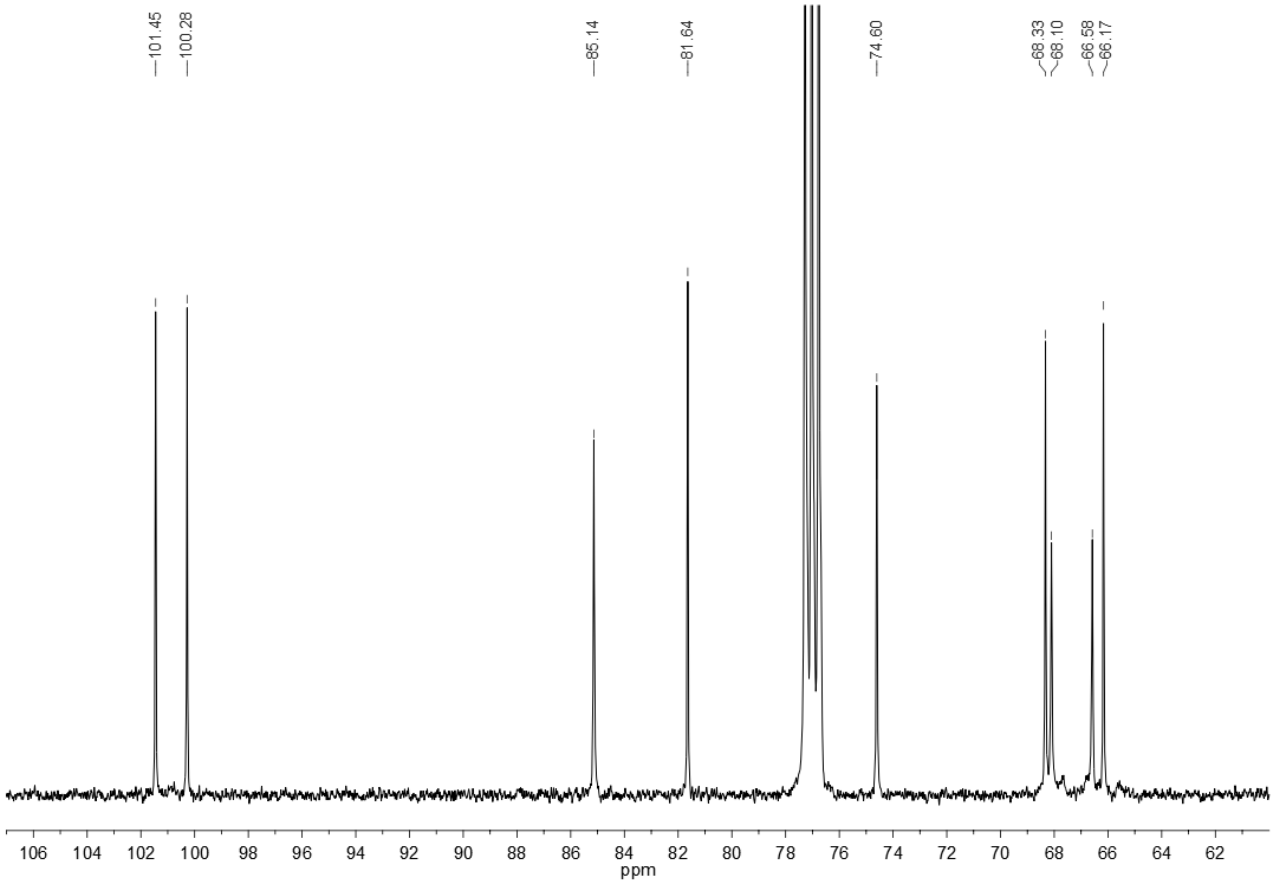

(41): ^{1}H NMR (500 MHz, $CDCl_3$)

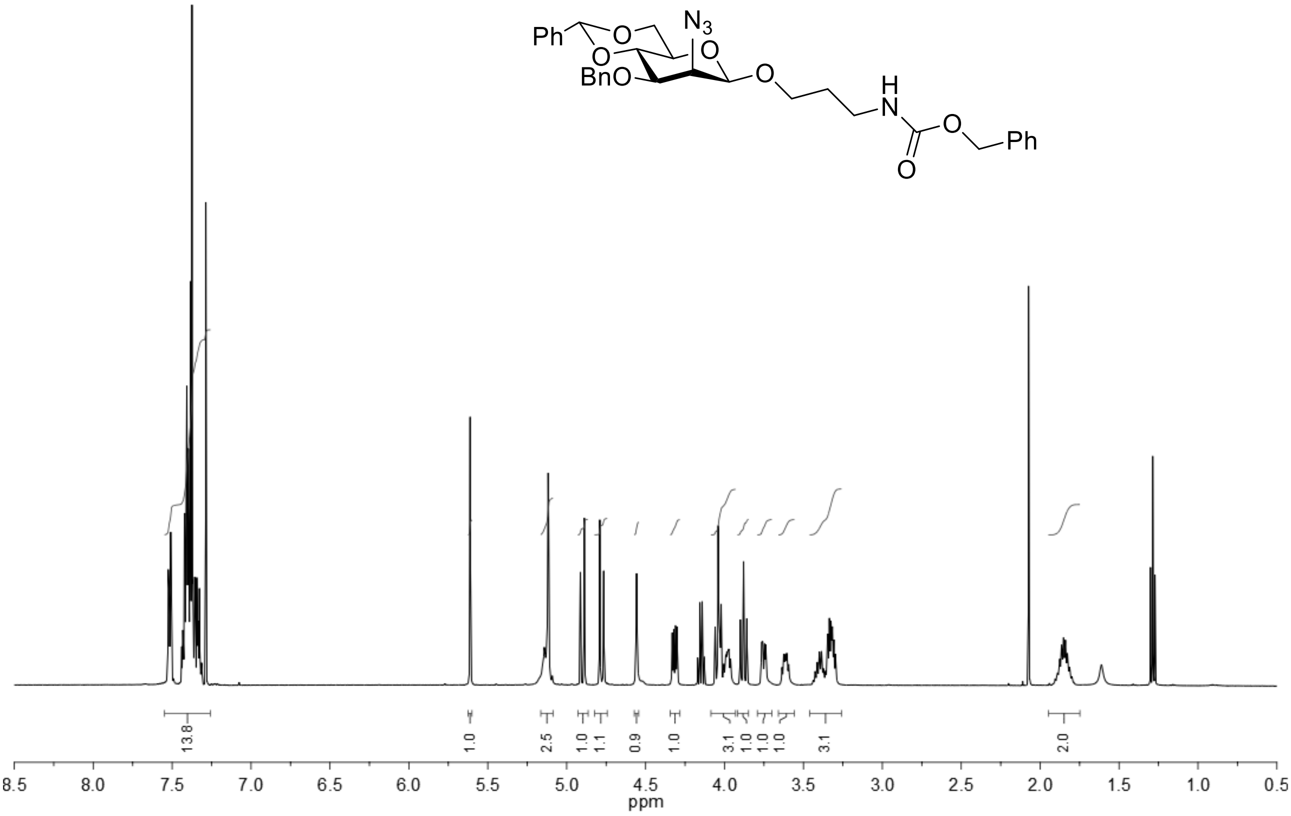

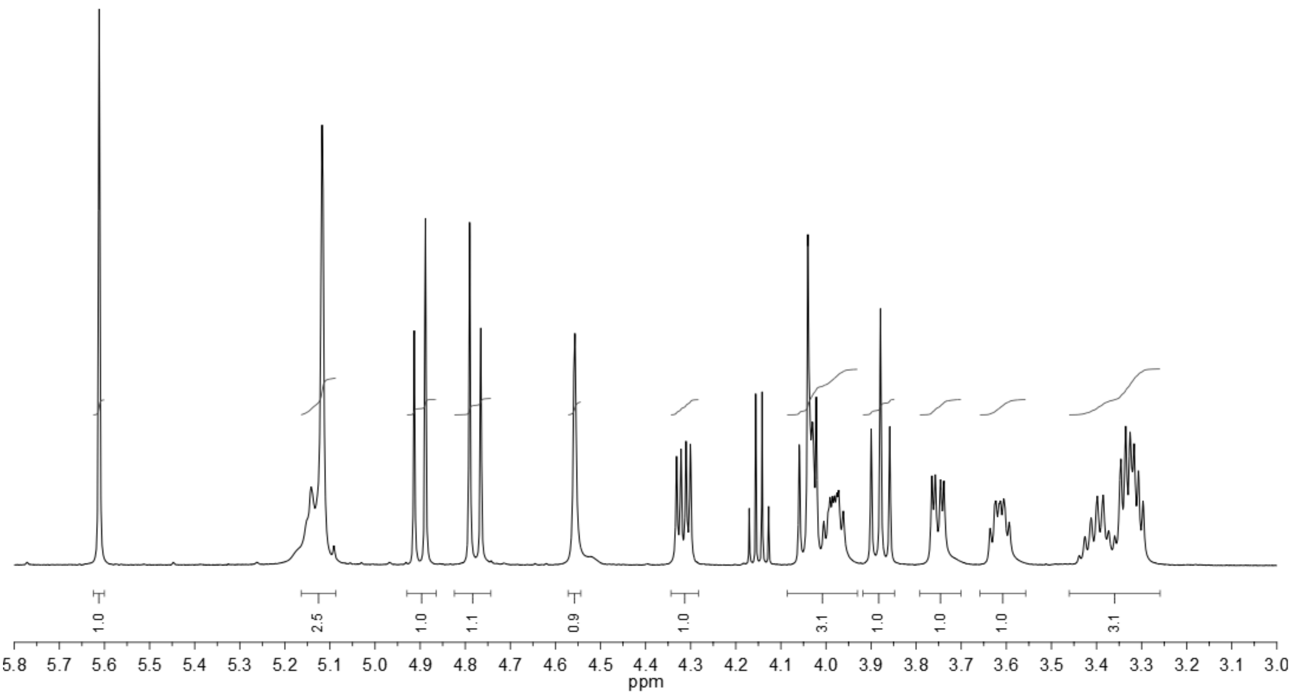

(41): ^{13}C NMR (126 MHz, $CDCl_3$)

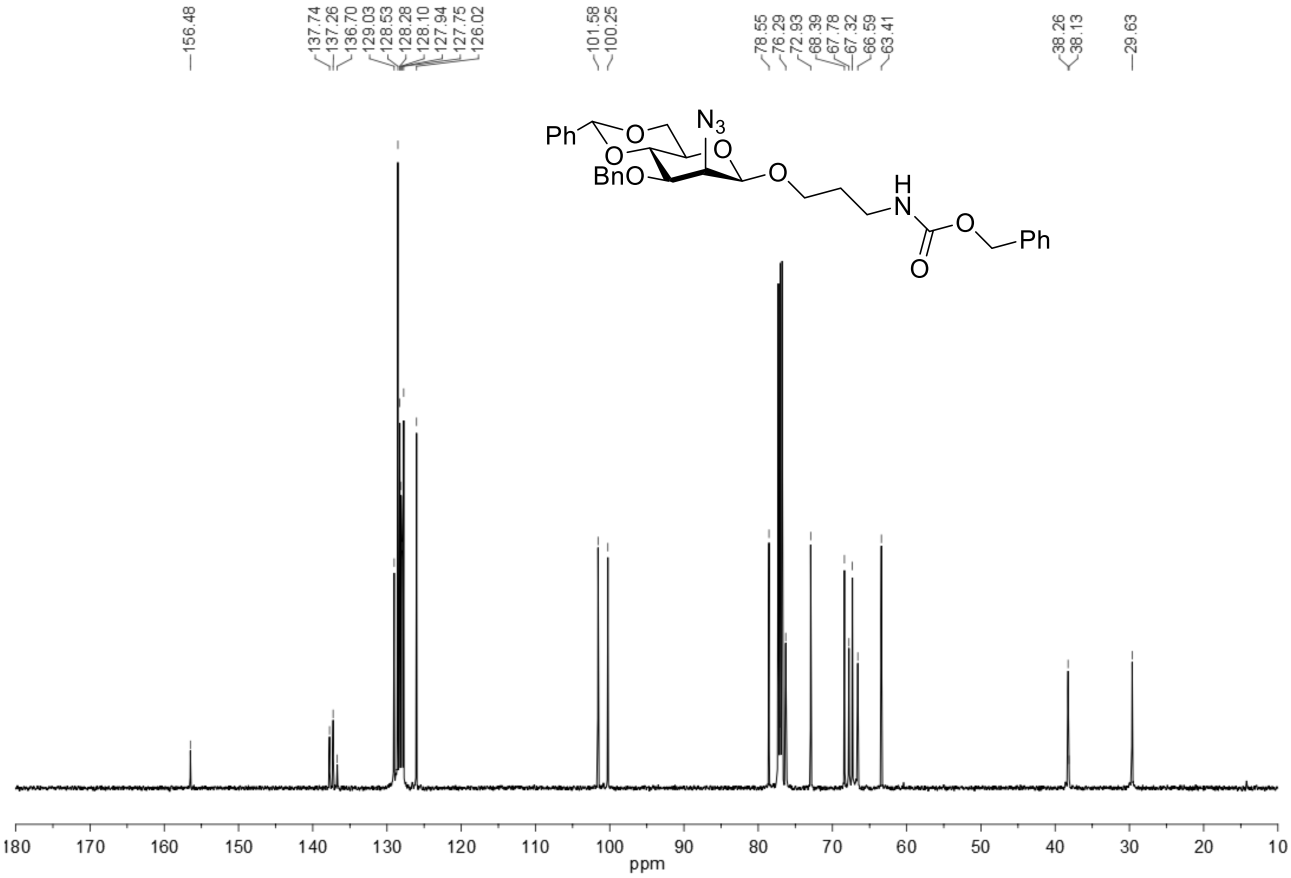

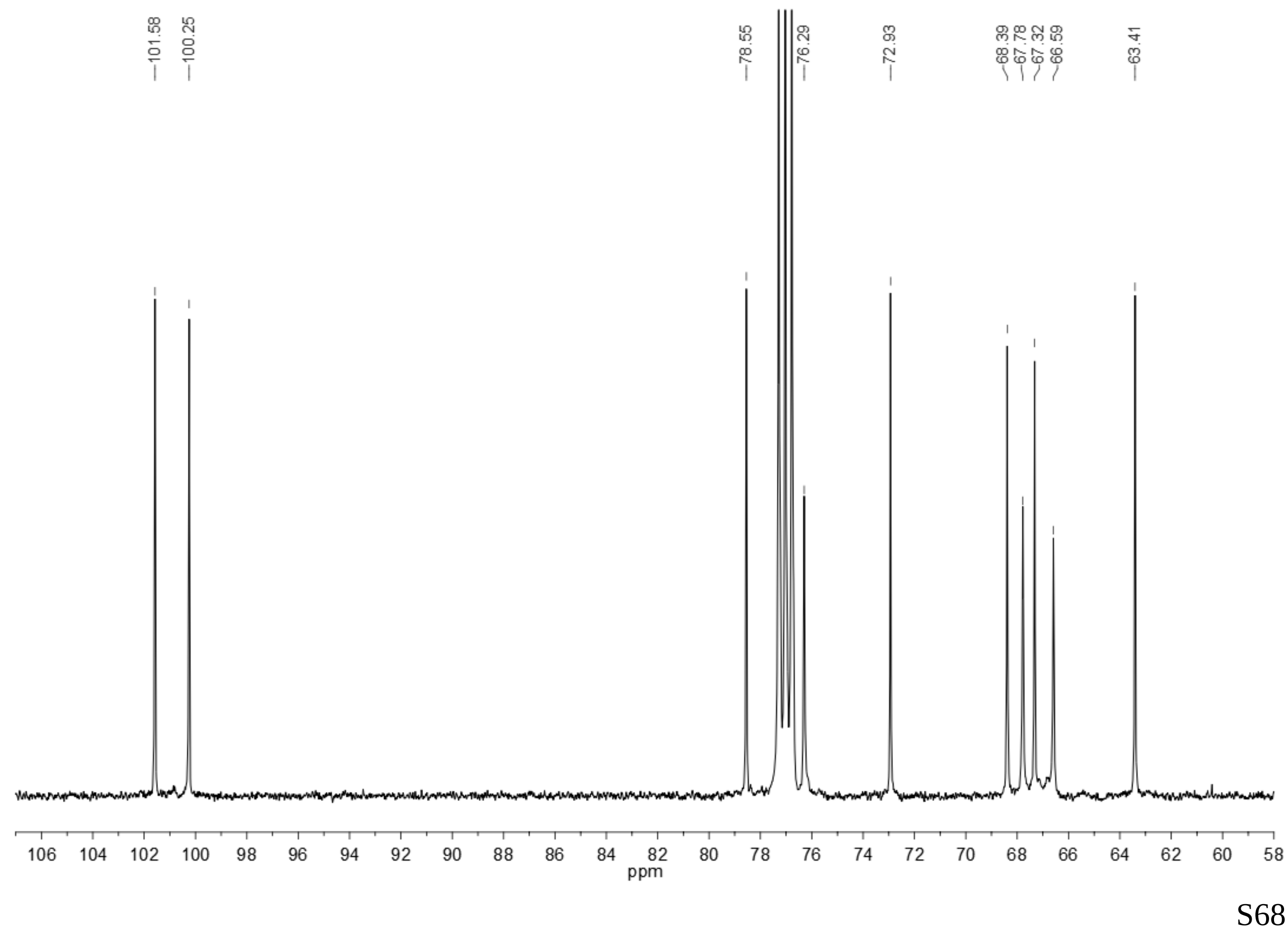

(8): ^{1}H NMR (500 MHz, $CDCl_3$)

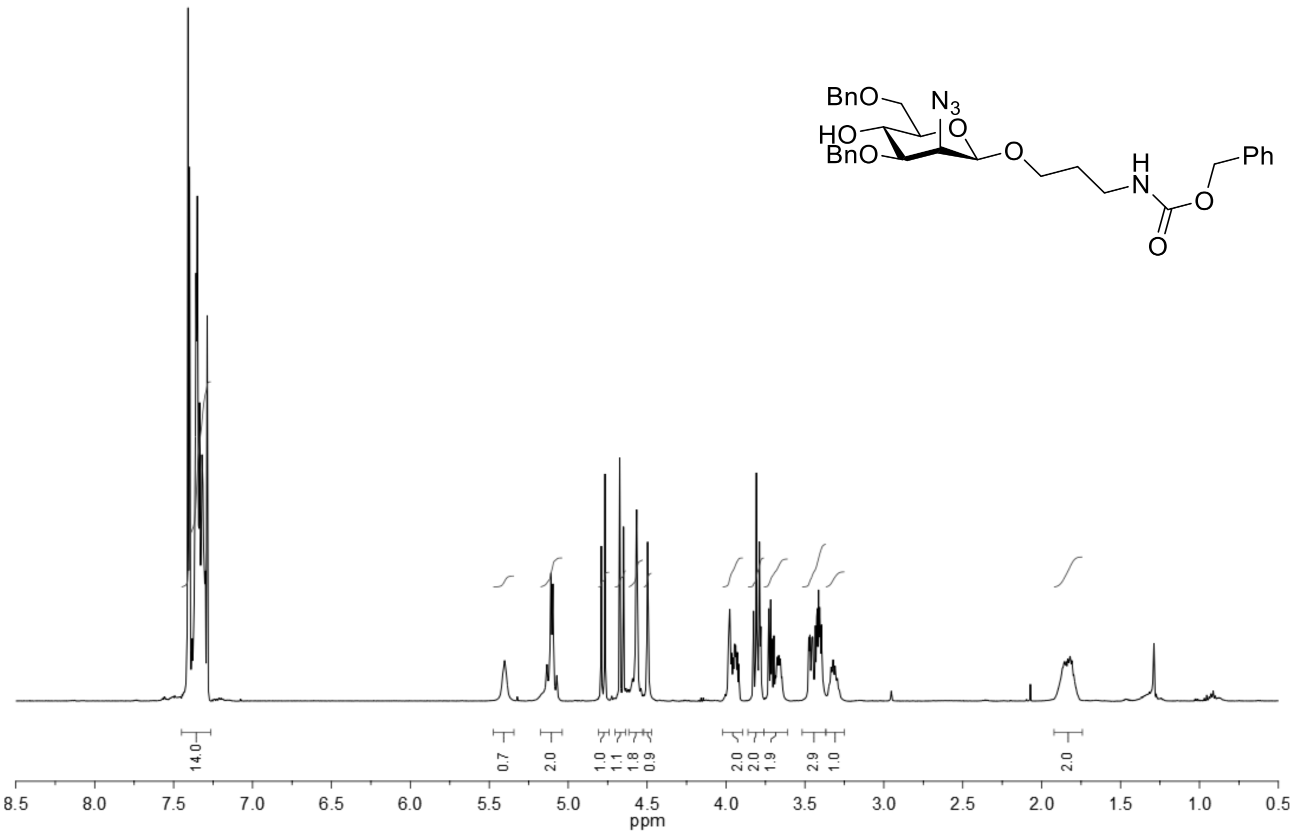

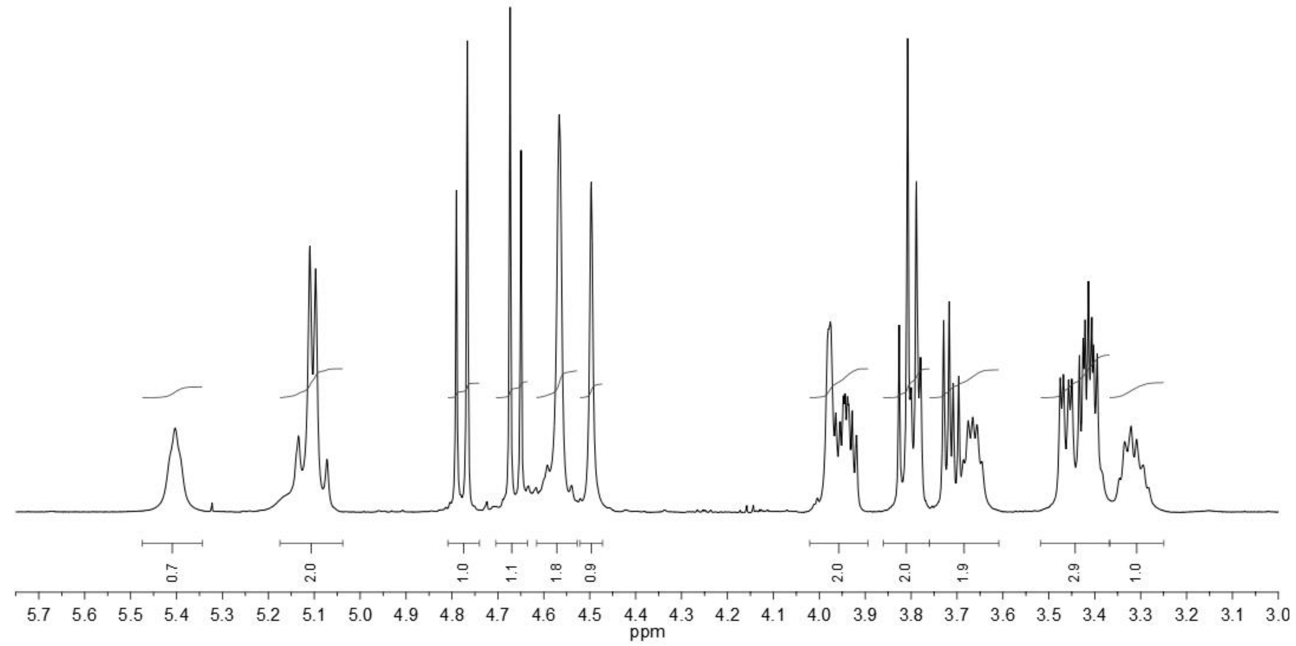

(8): ^{13}C NMR (126 MHz, $CDCl_3$)

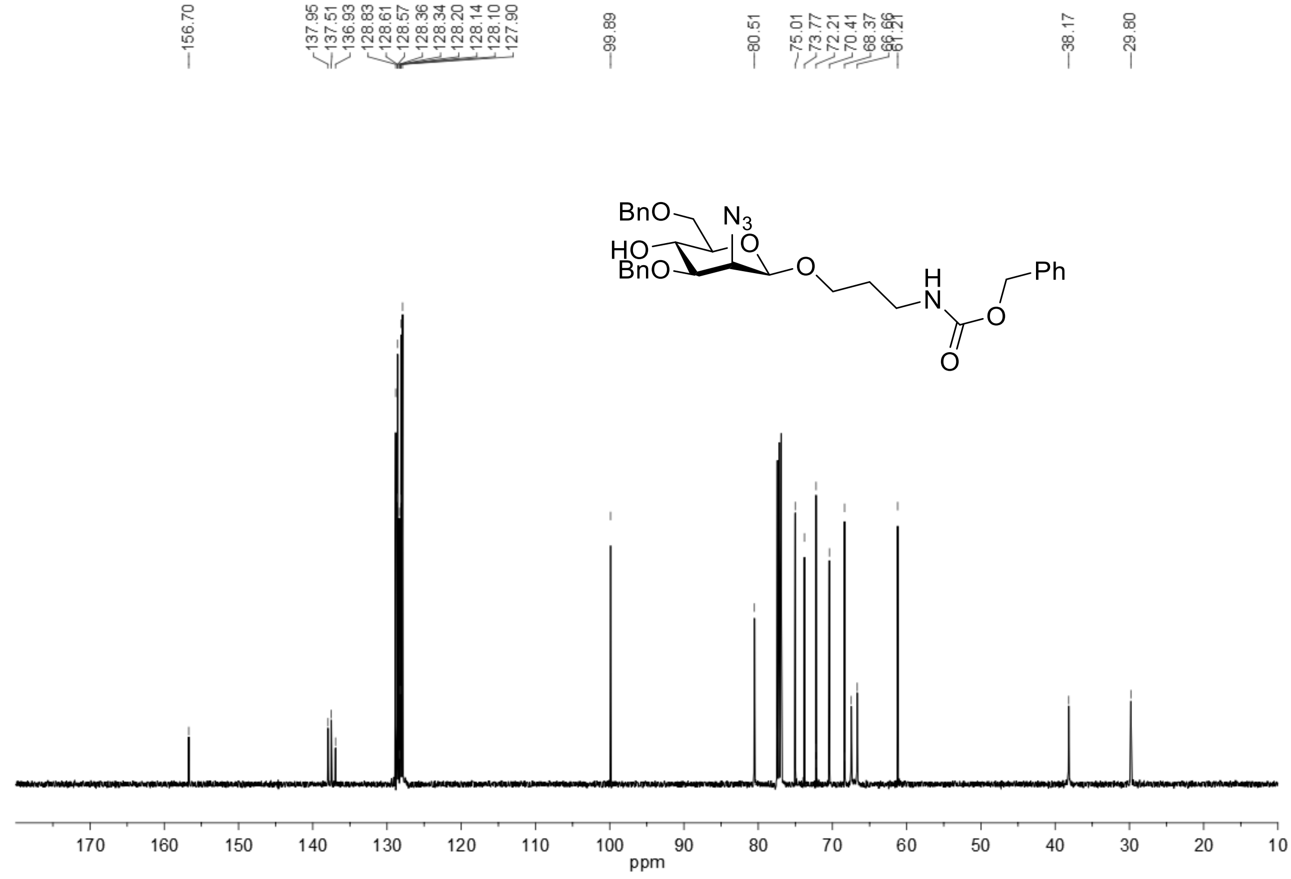

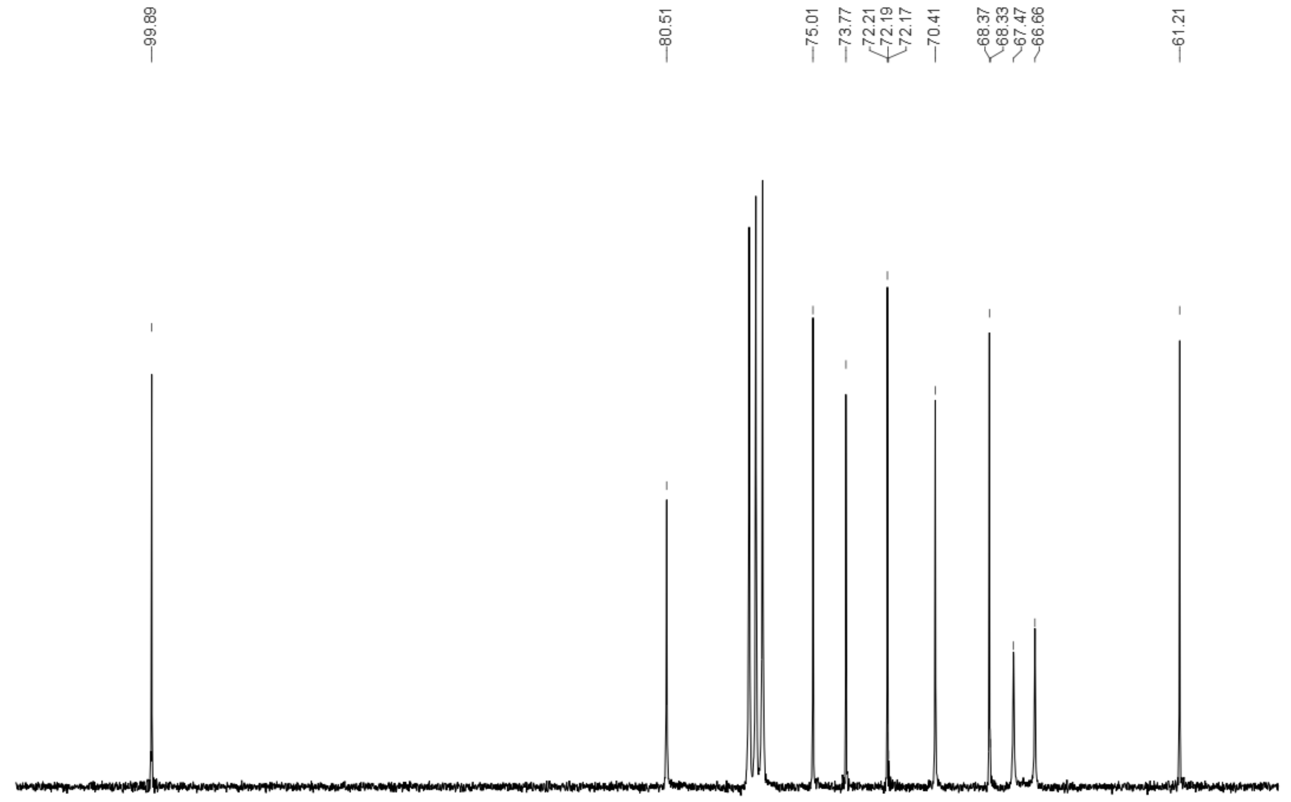

(42): ^{1}H NMR (500 MHz, $CDCl_3$)

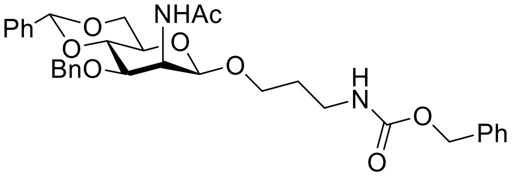

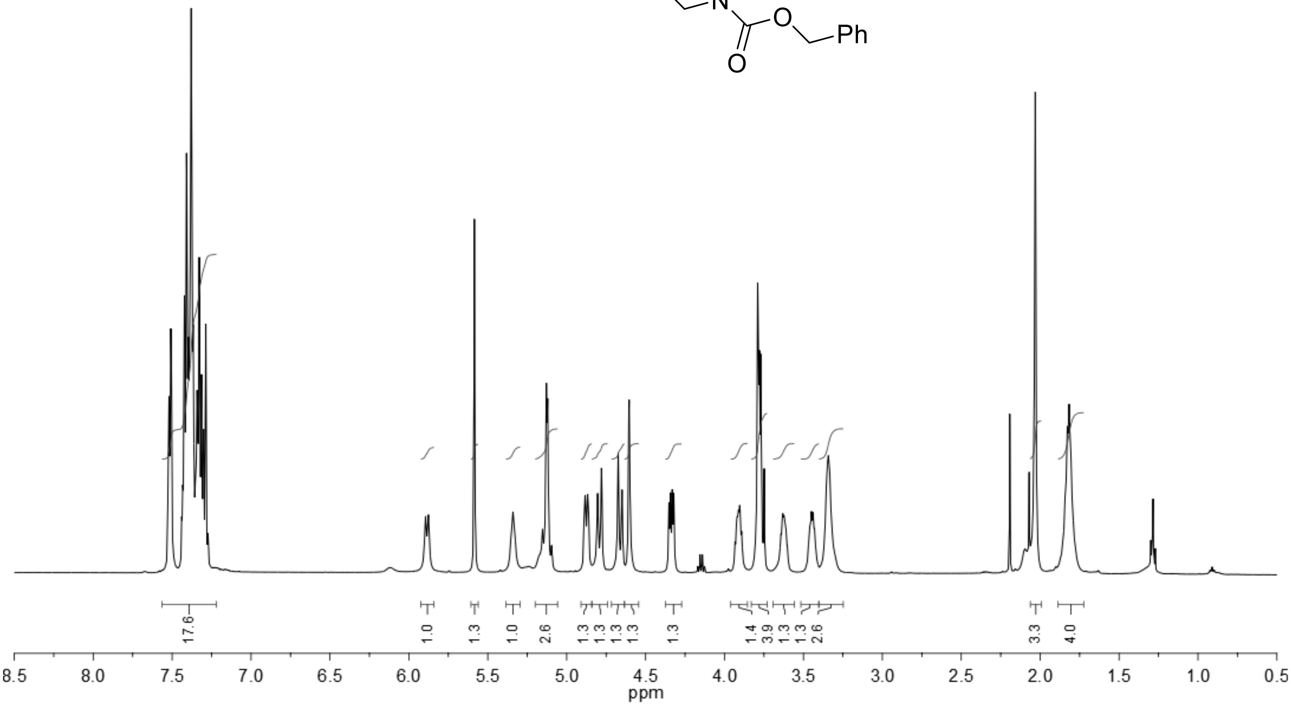

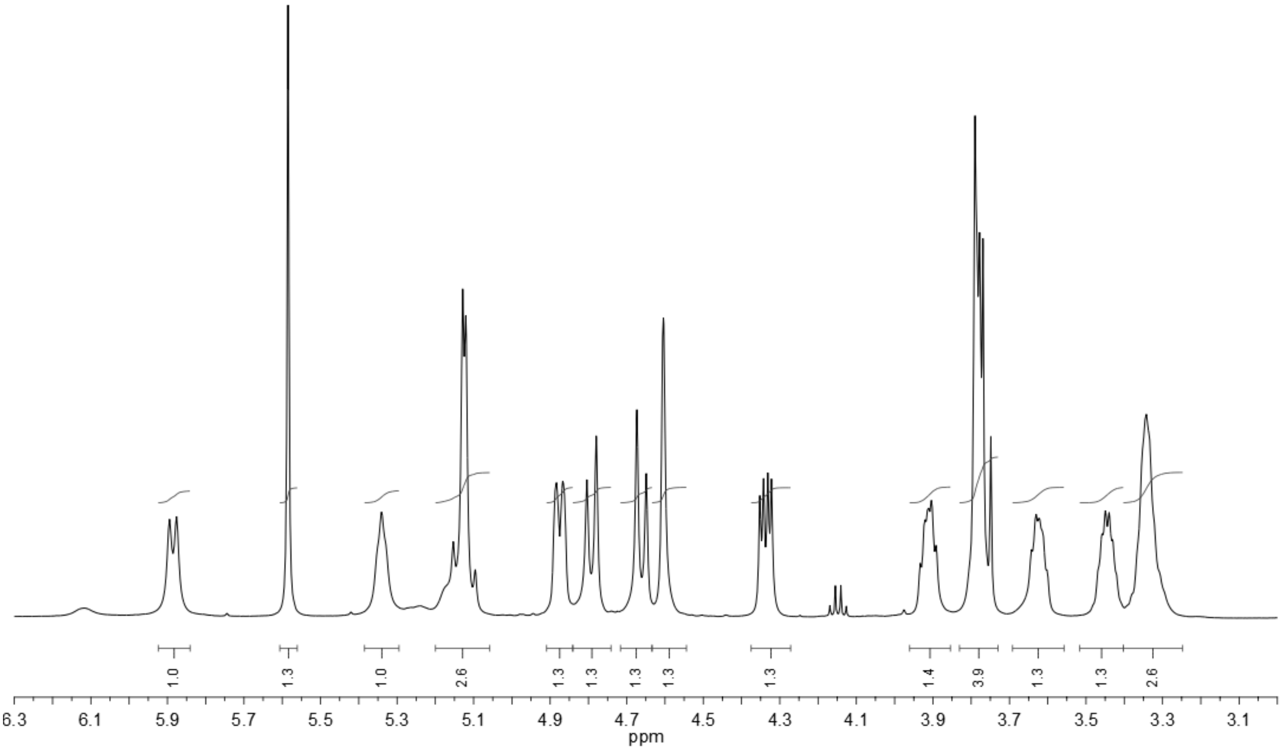

(42): ^{13}C NMR (126 MHz, $CDCl_3$)

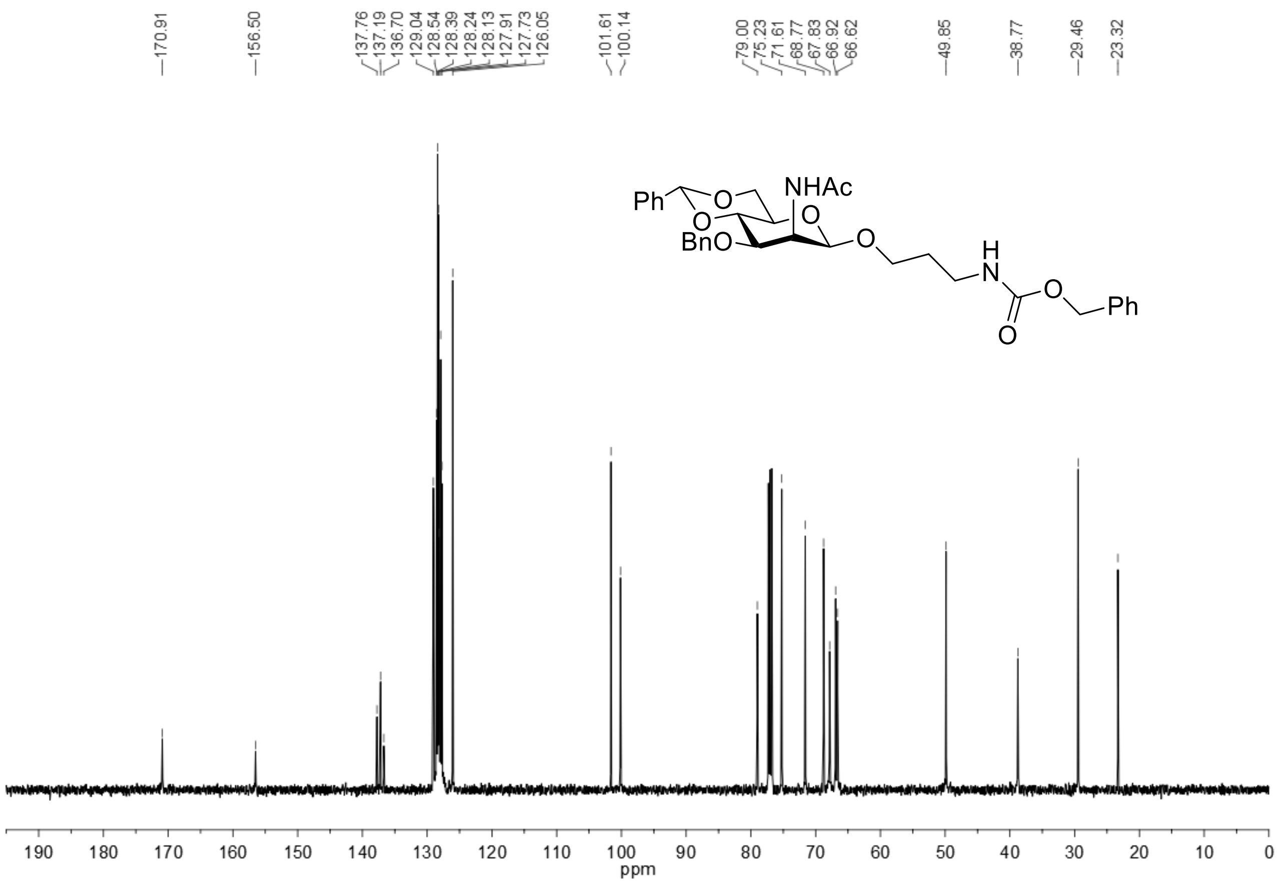

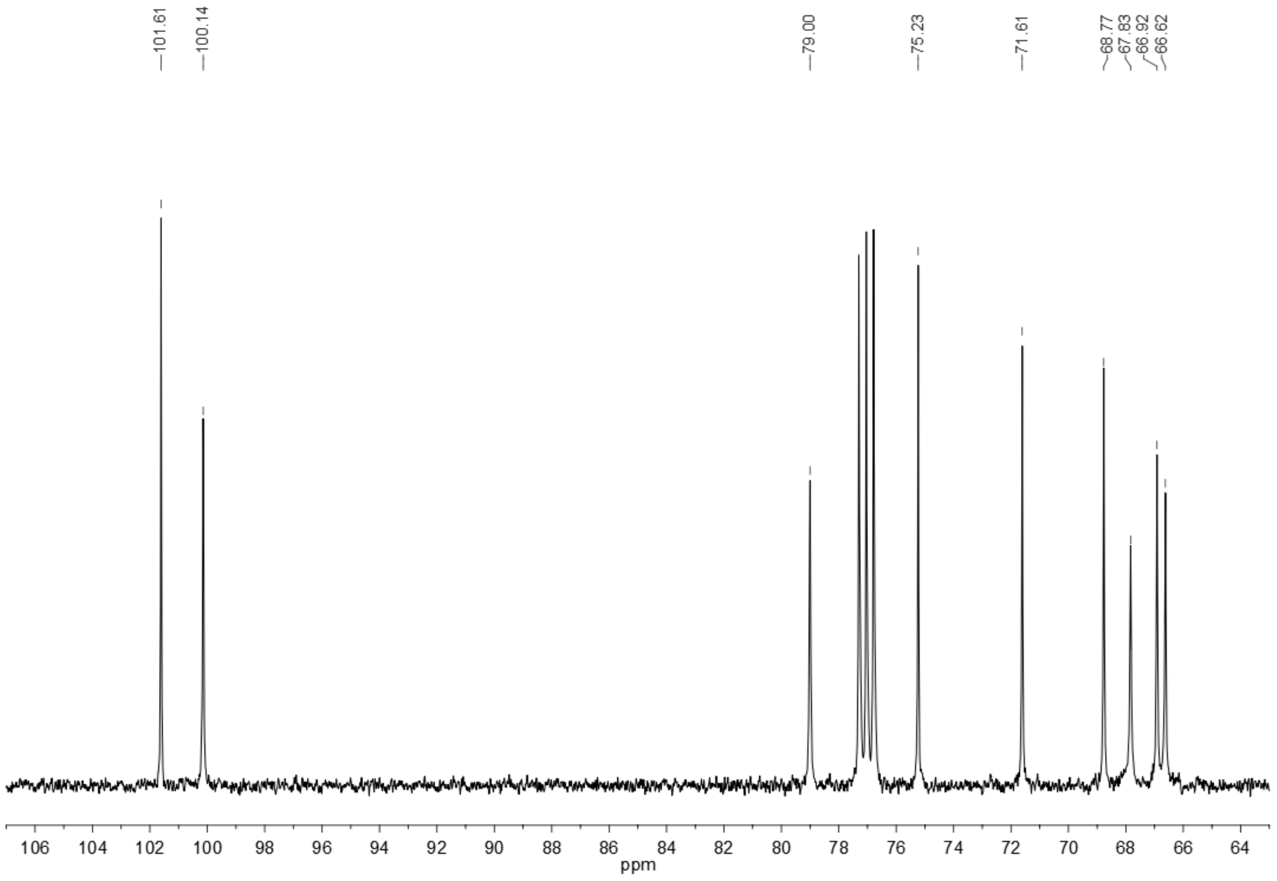

(9): ^{1}H NMR (500 MHz, CD_3OD)

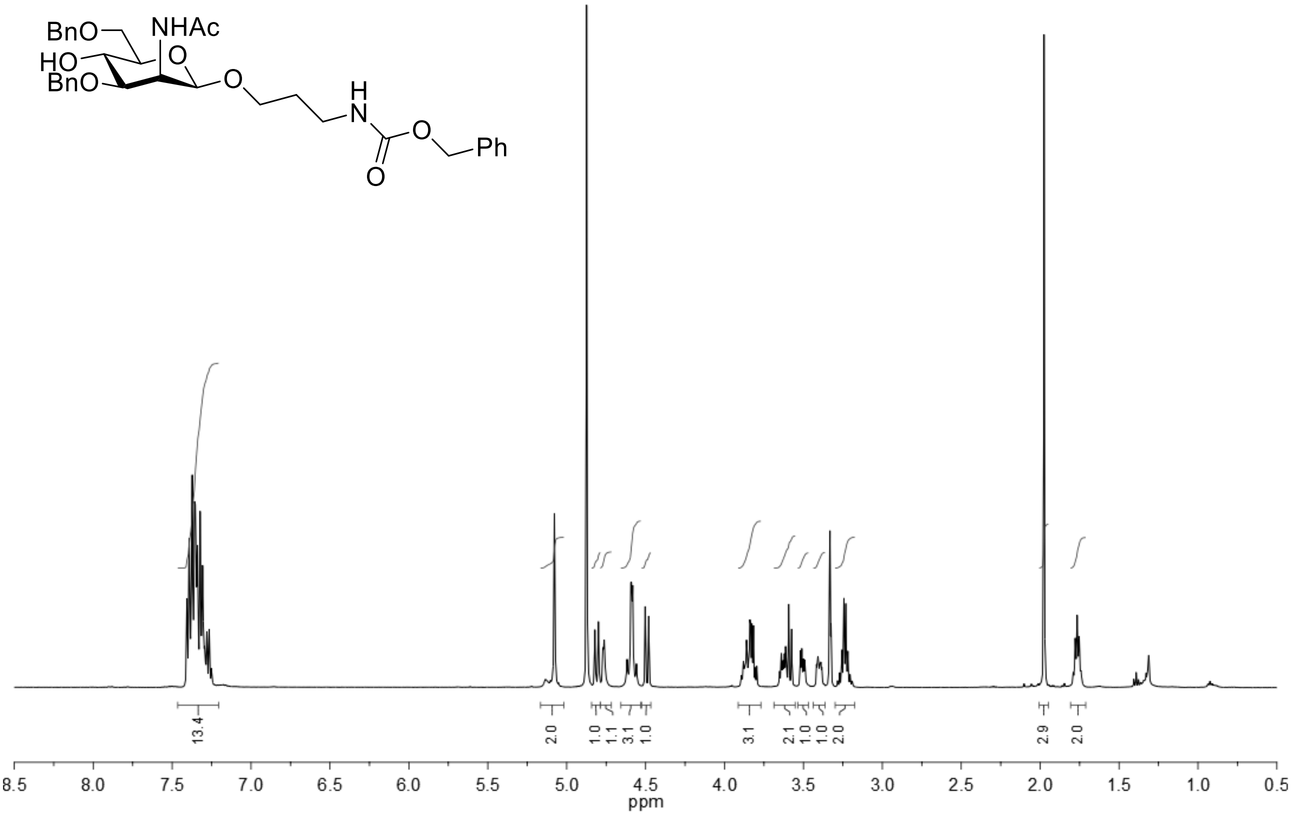

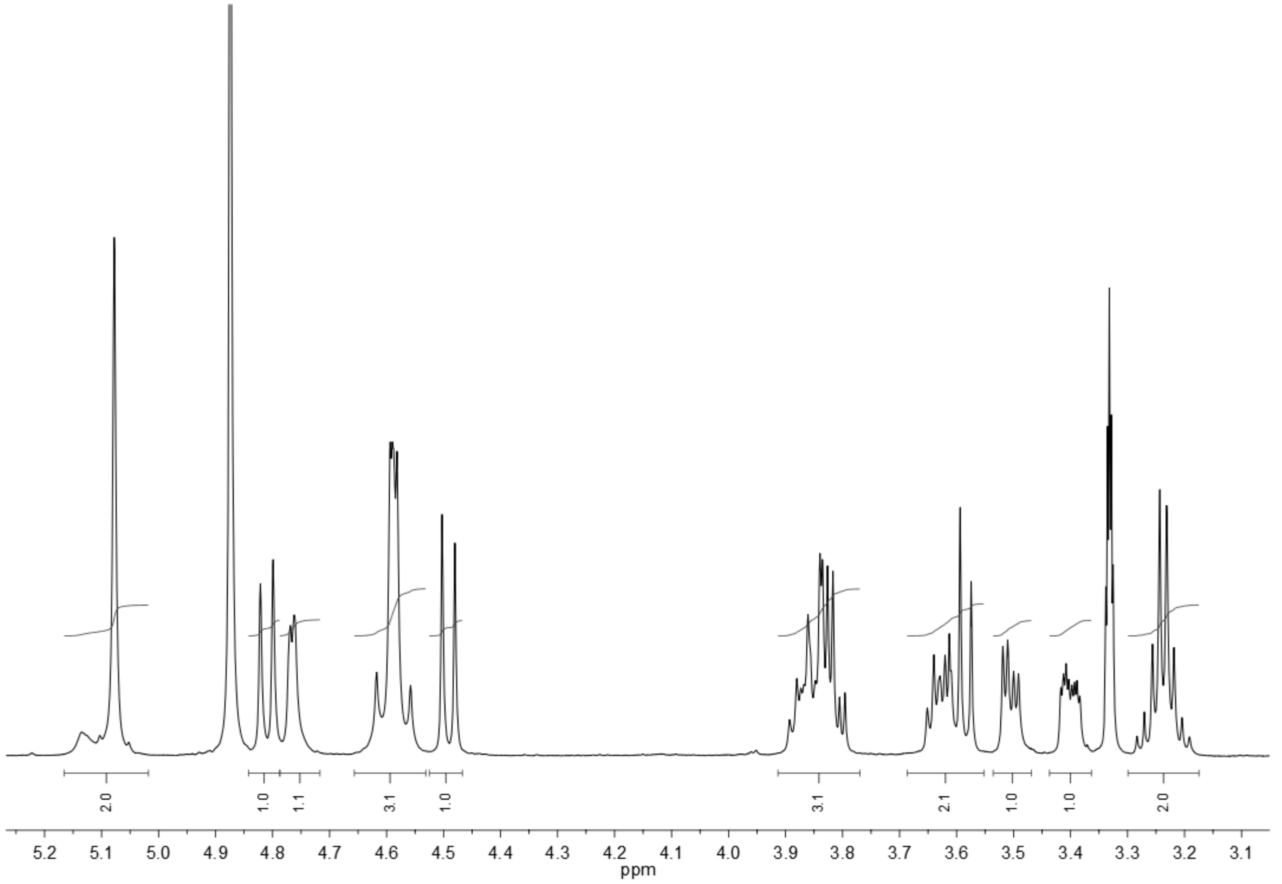

(9): ^{13}C NMR (126 MHz, CD_3OD)

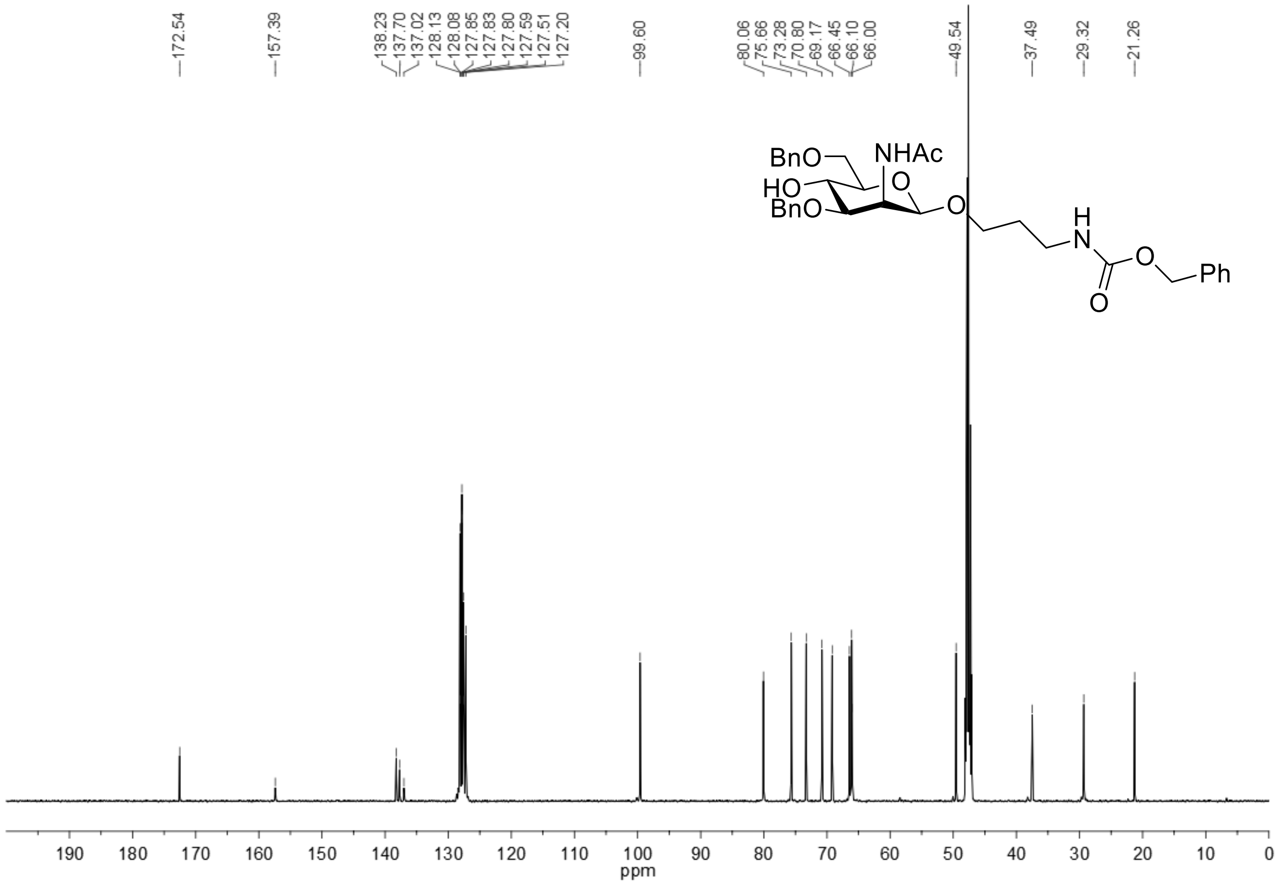

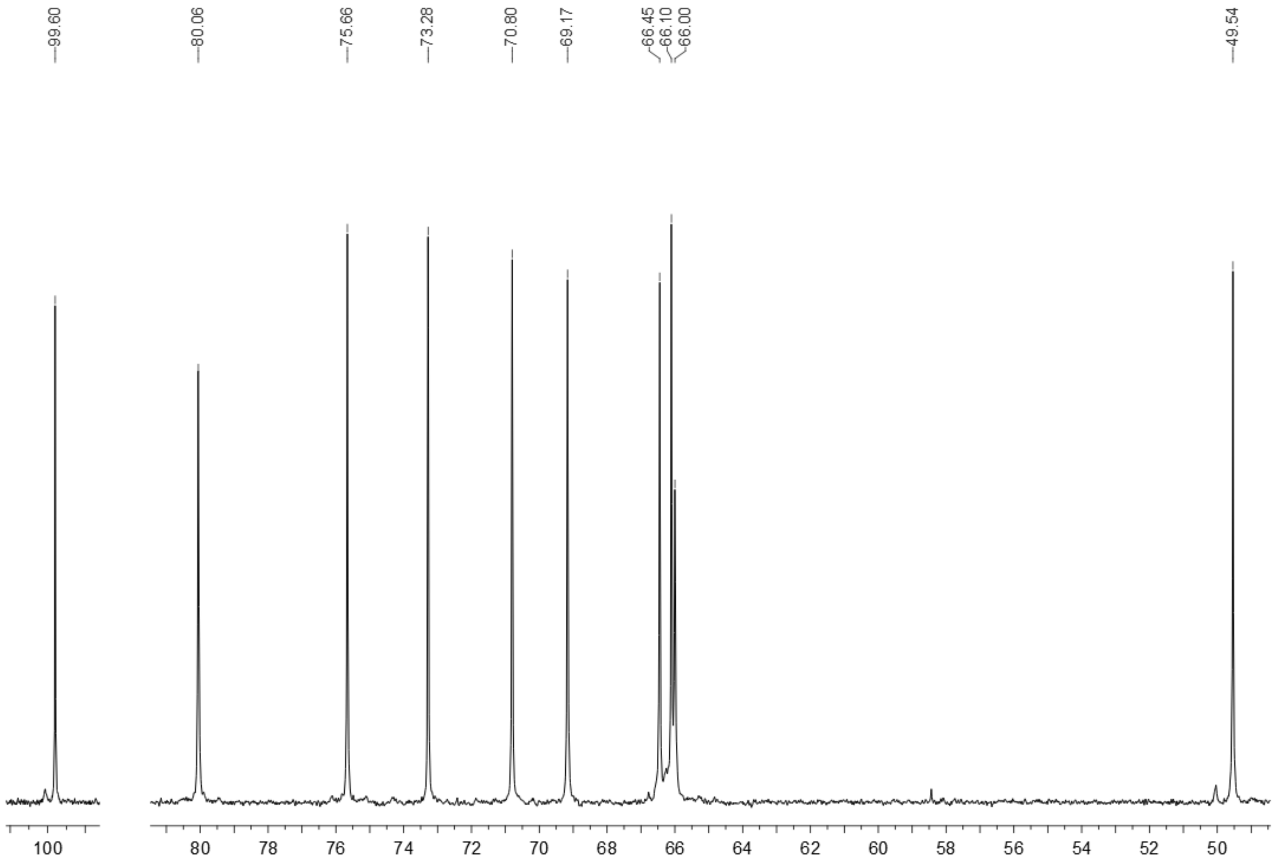

(44): ^{1}H NMR (500 MHz, $CDCl_3$)

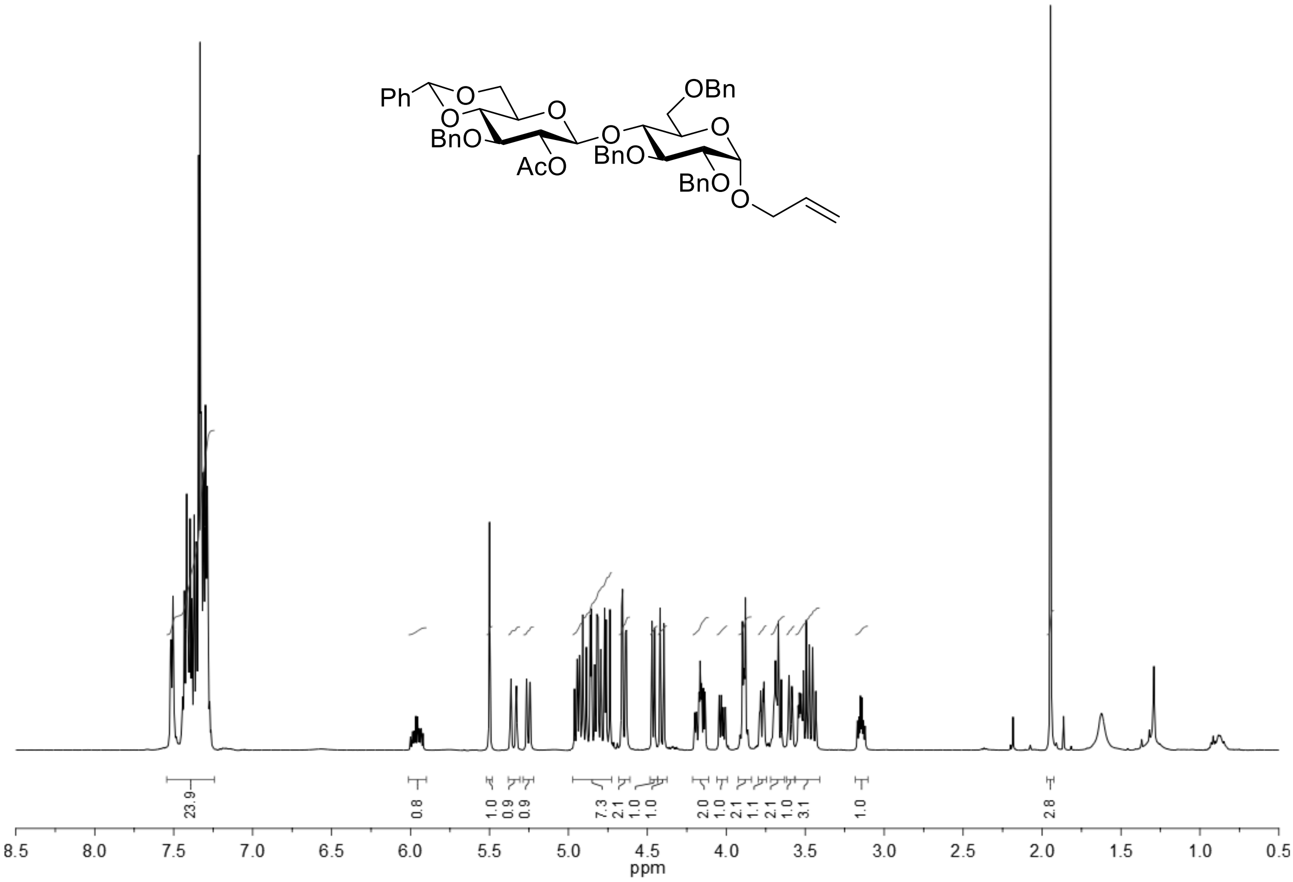

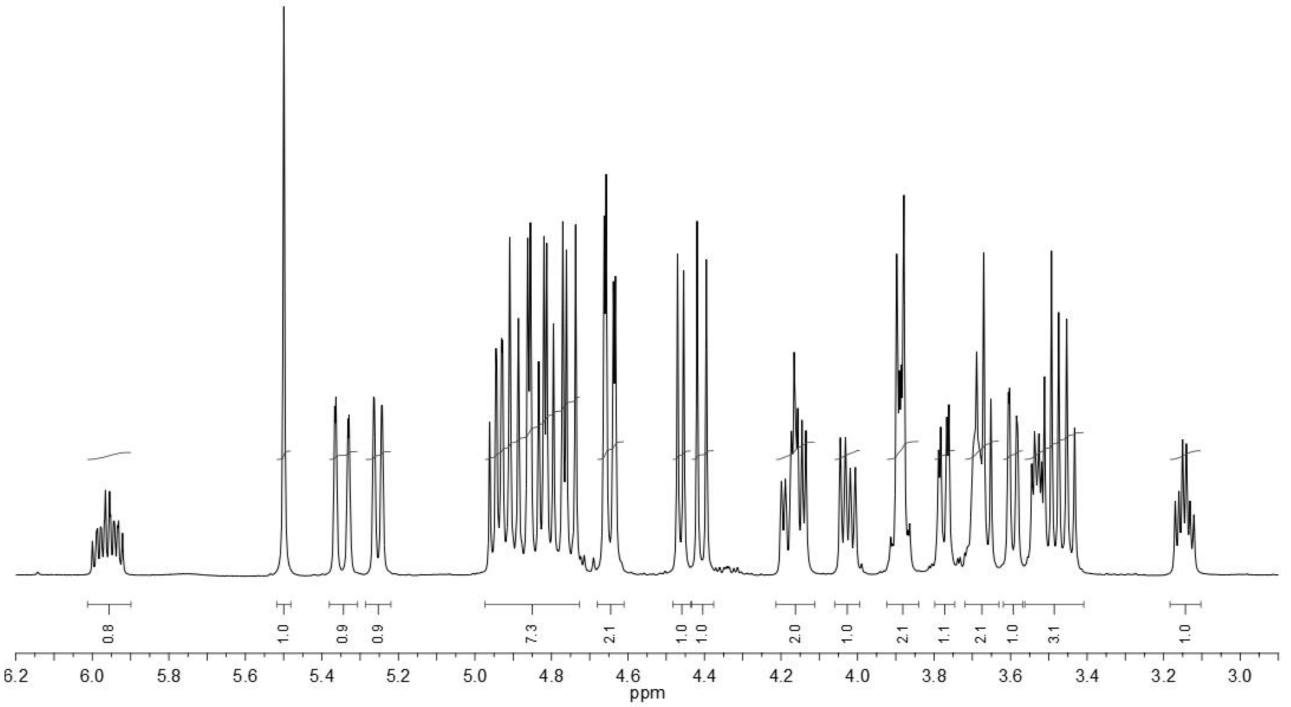

(44): ^{13}C NMR (126 MHz, CDCl$_3$)

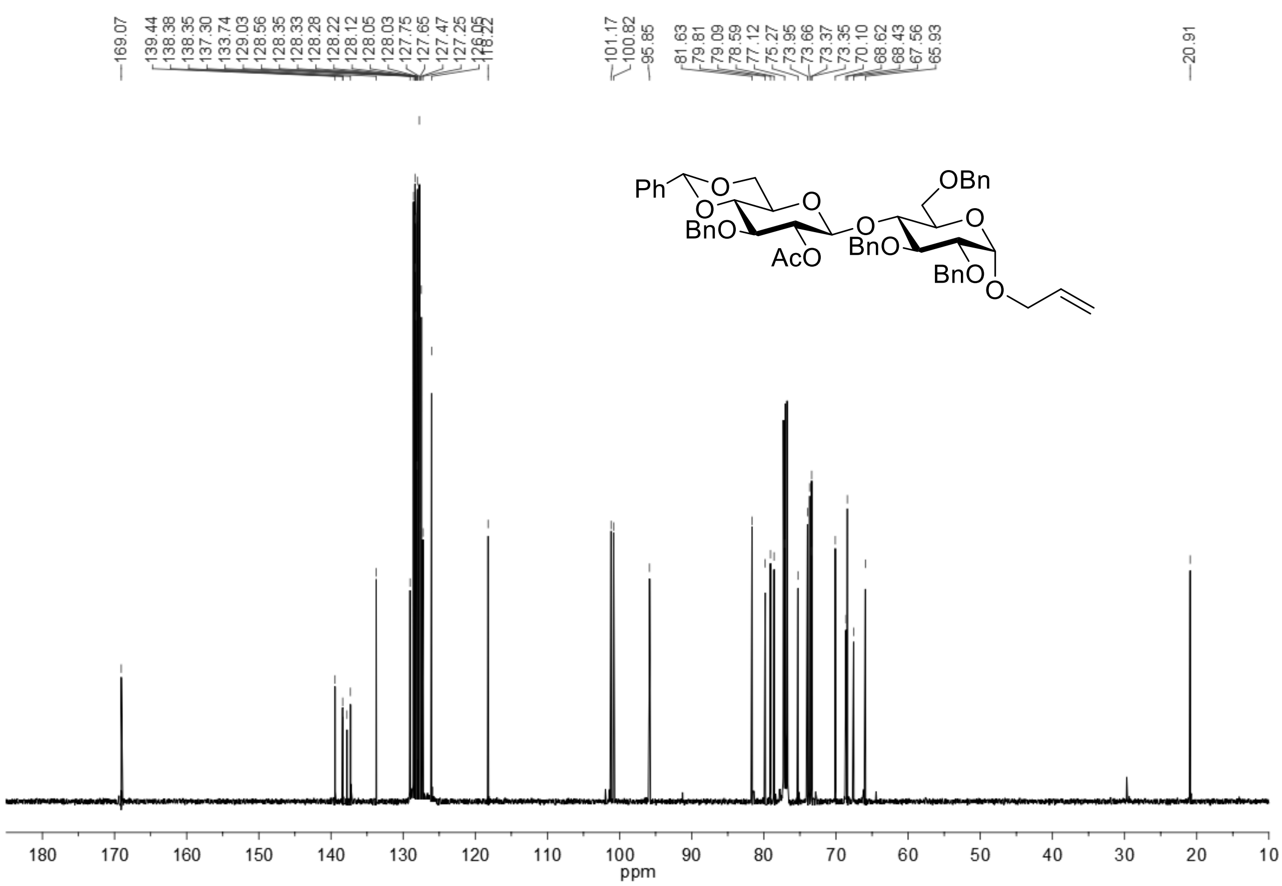

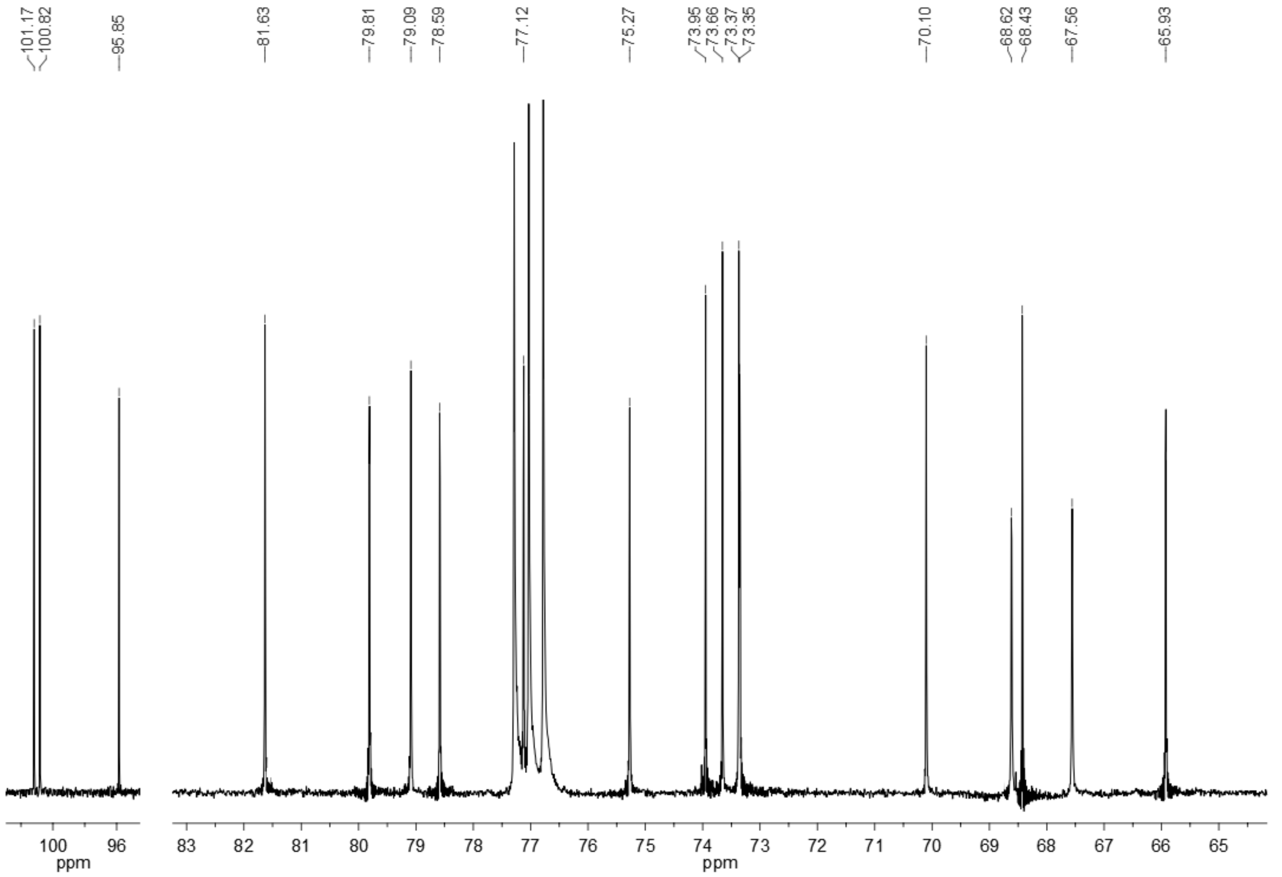

(45): ^{1}H NMR (500 MHz, $CDCl_3$)

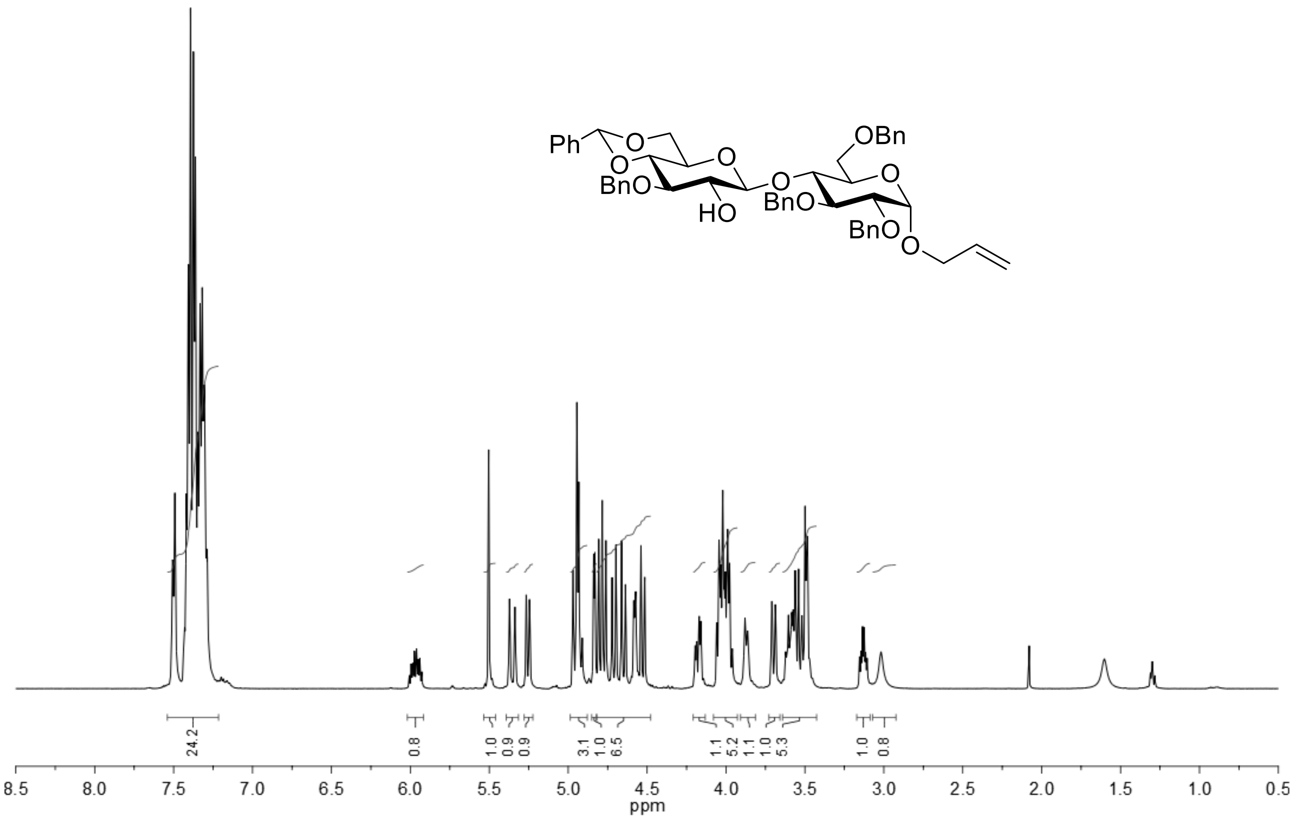

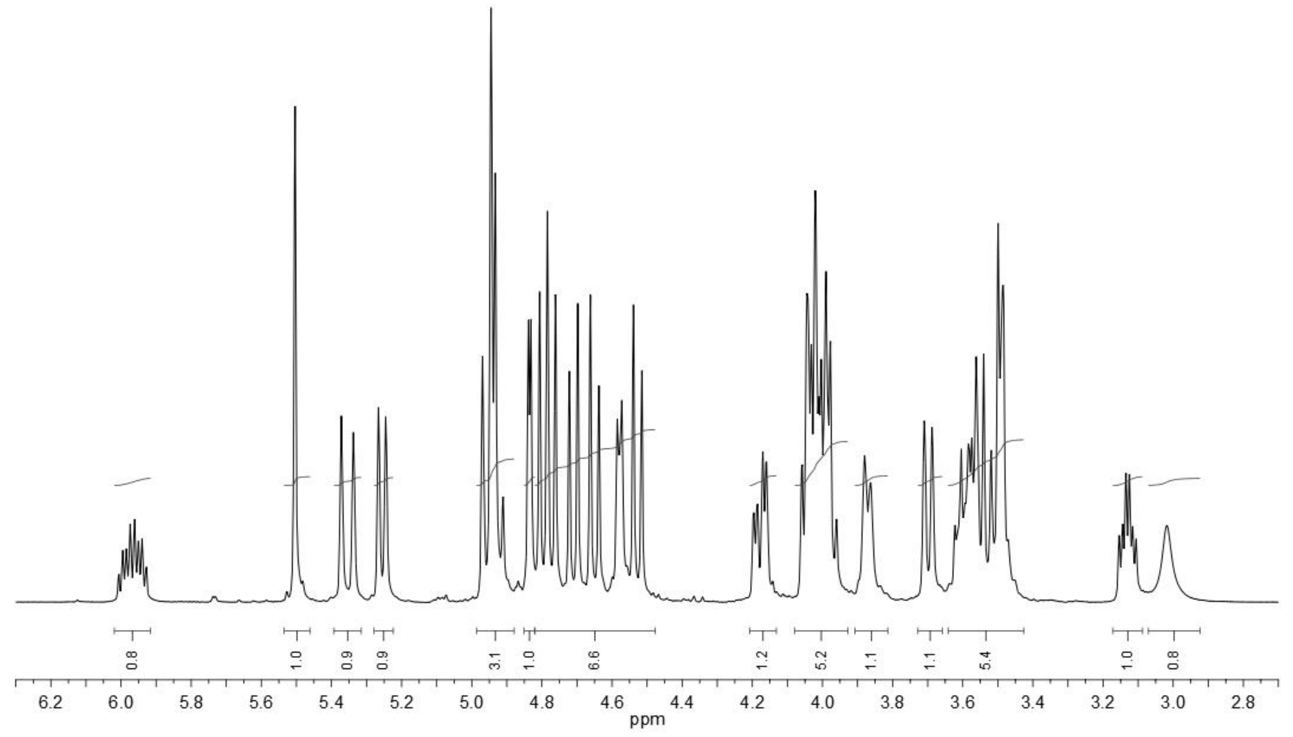

(45): ^{13}C NMR (126 MHz, CDCl$_3$)

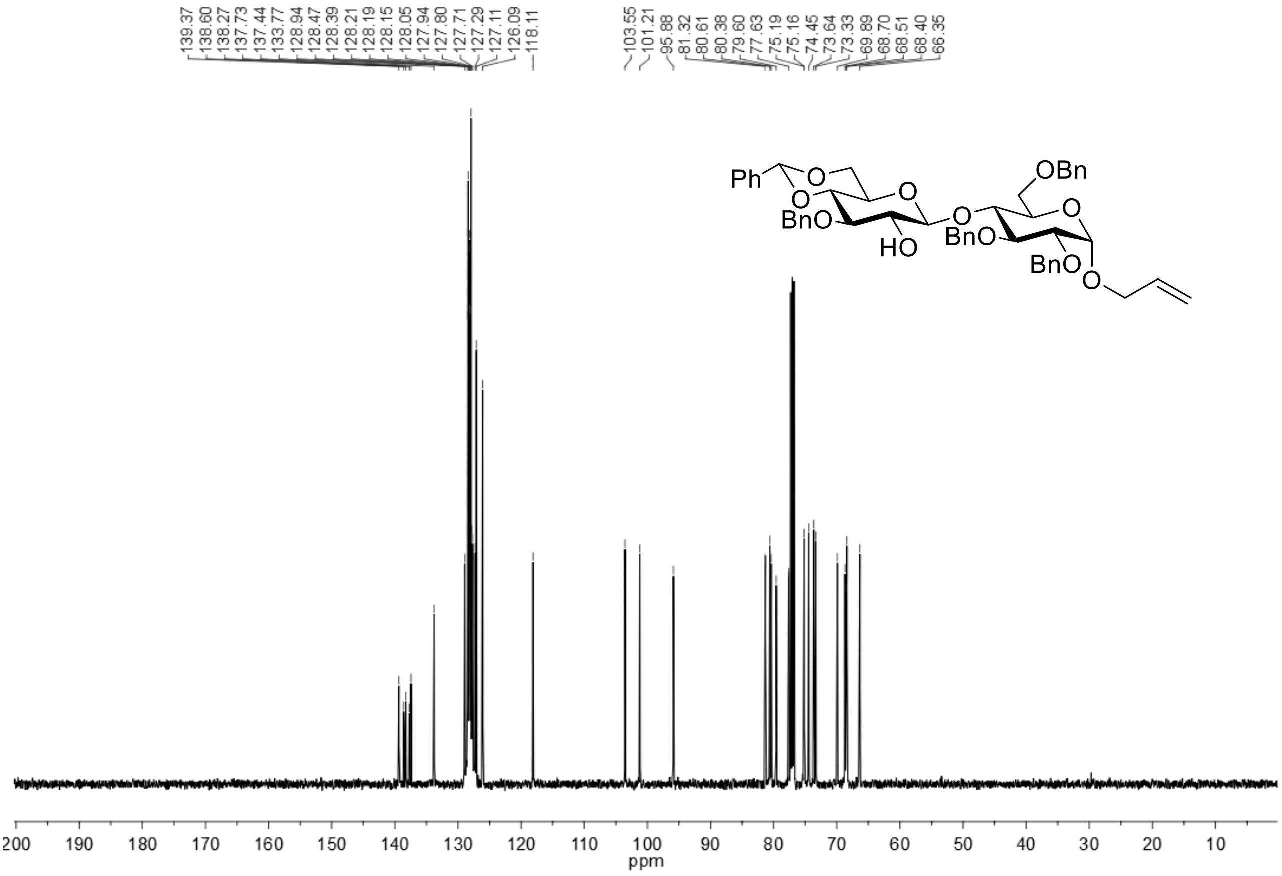

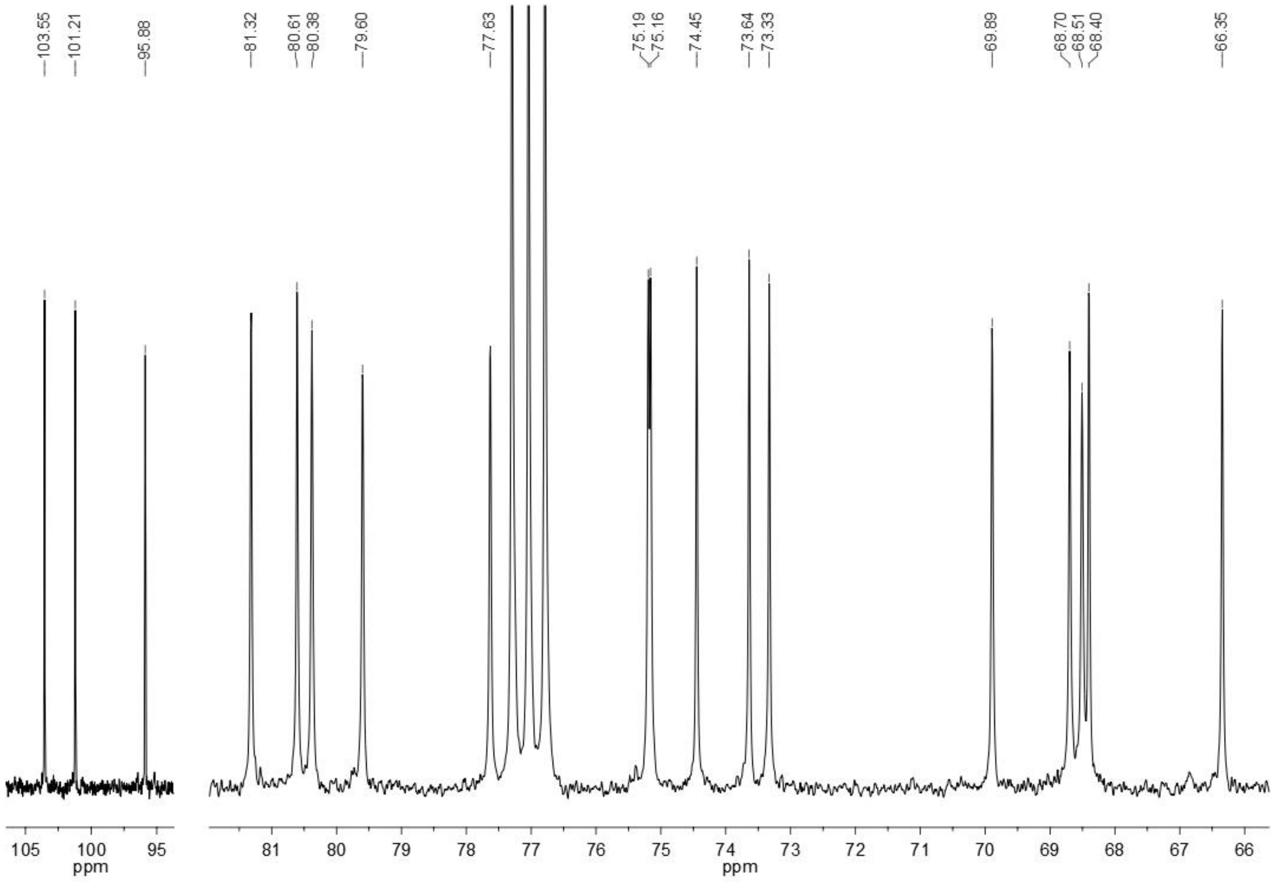

(46): ^{1}H NMR (500 MHz, $CDCl_3$)

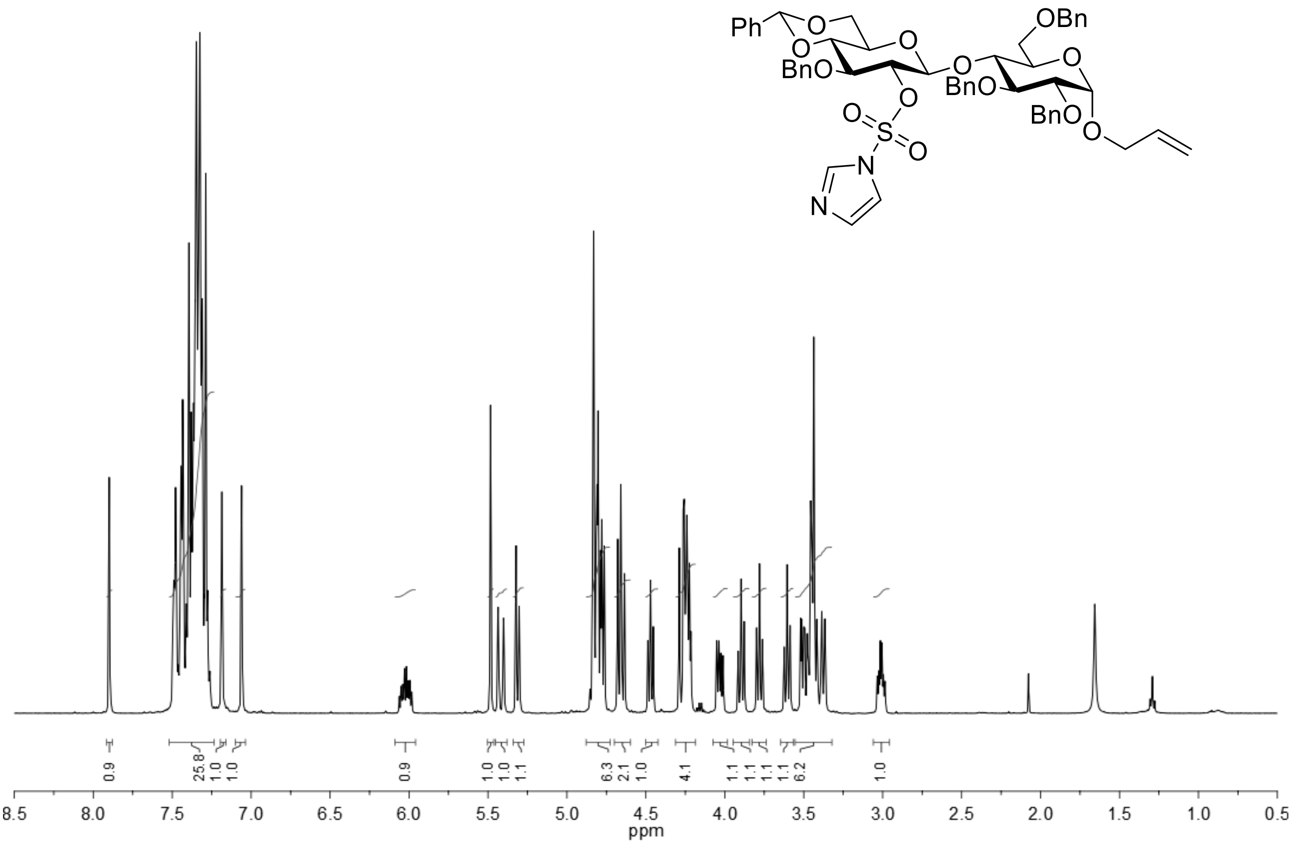

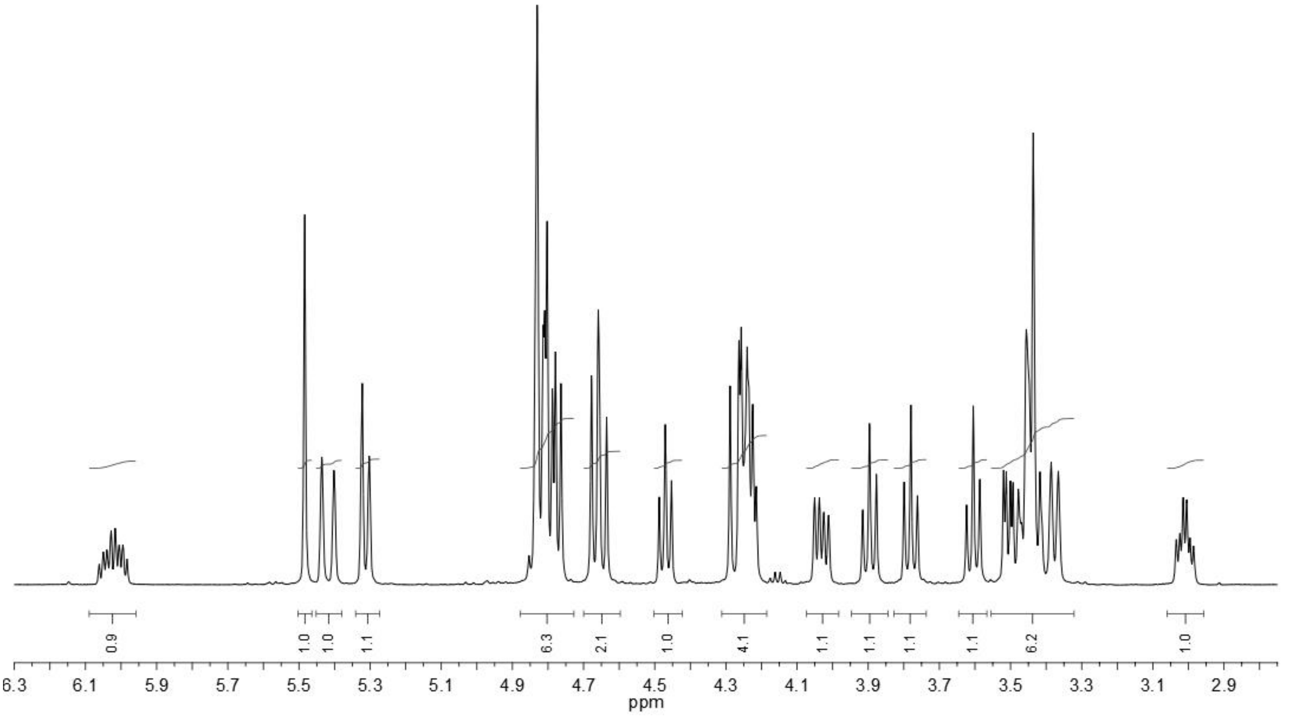

(46): ^{13}C NMR (126 MHz, $CDCl_3$)

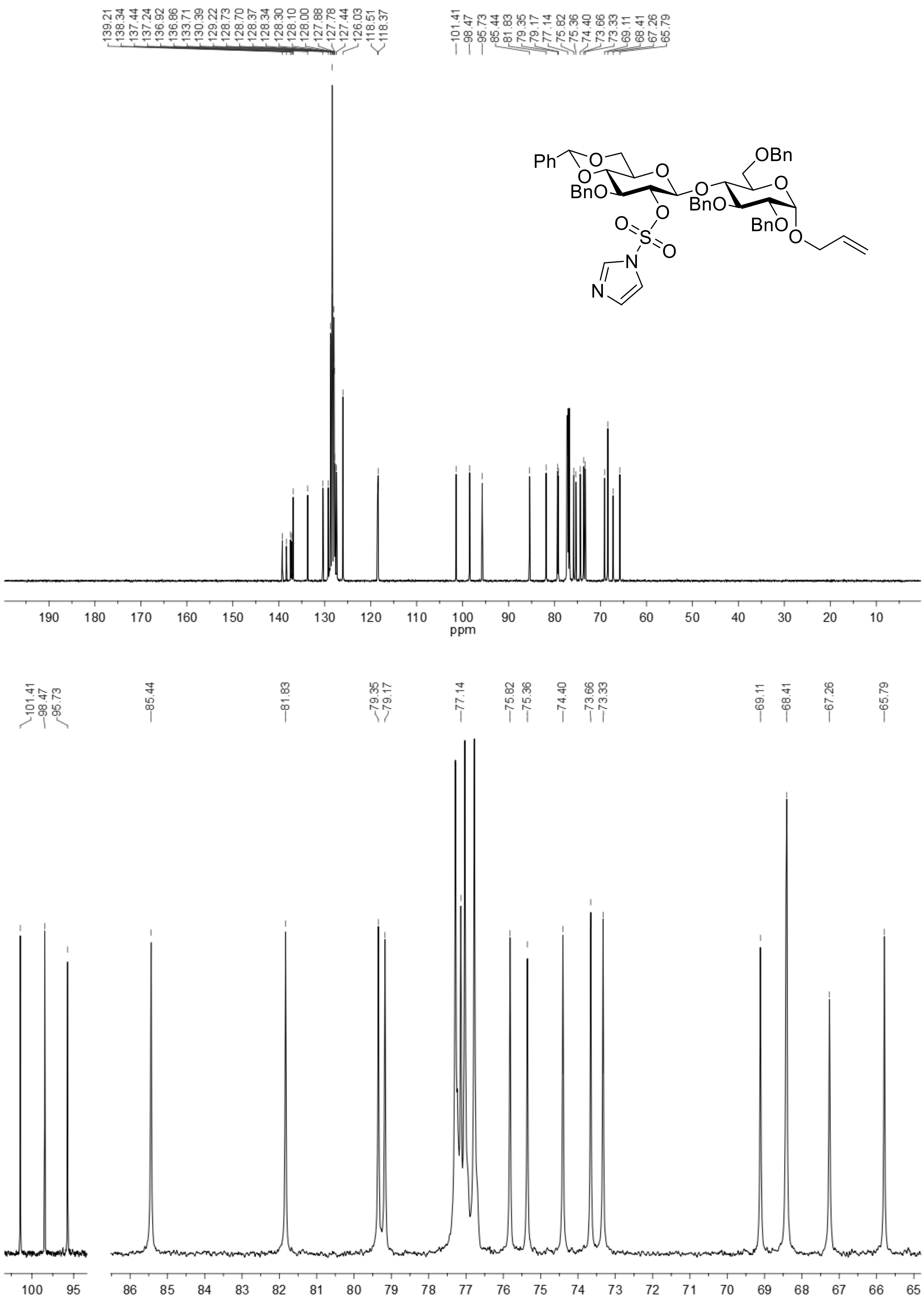

(47): ^{1}H NMR (500 MHz, $CDCl_3$)

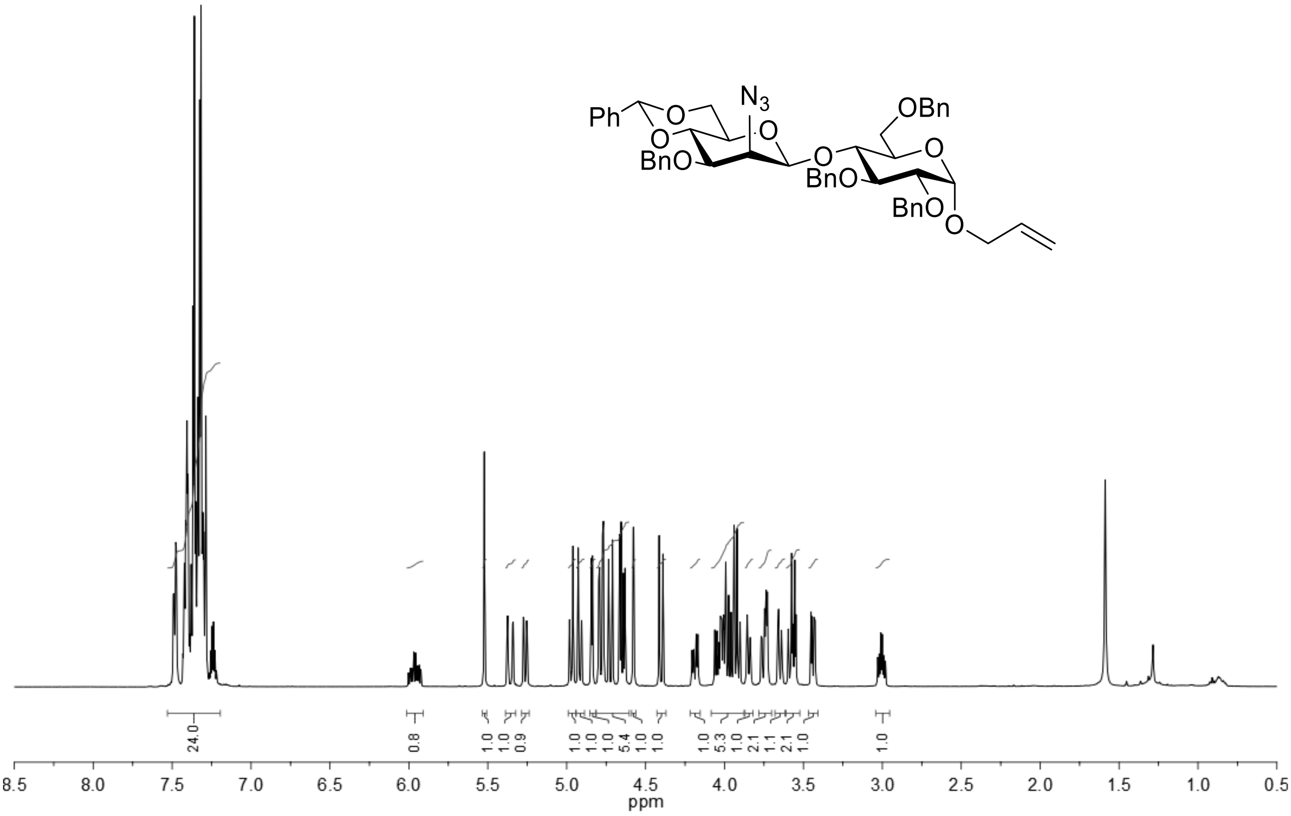

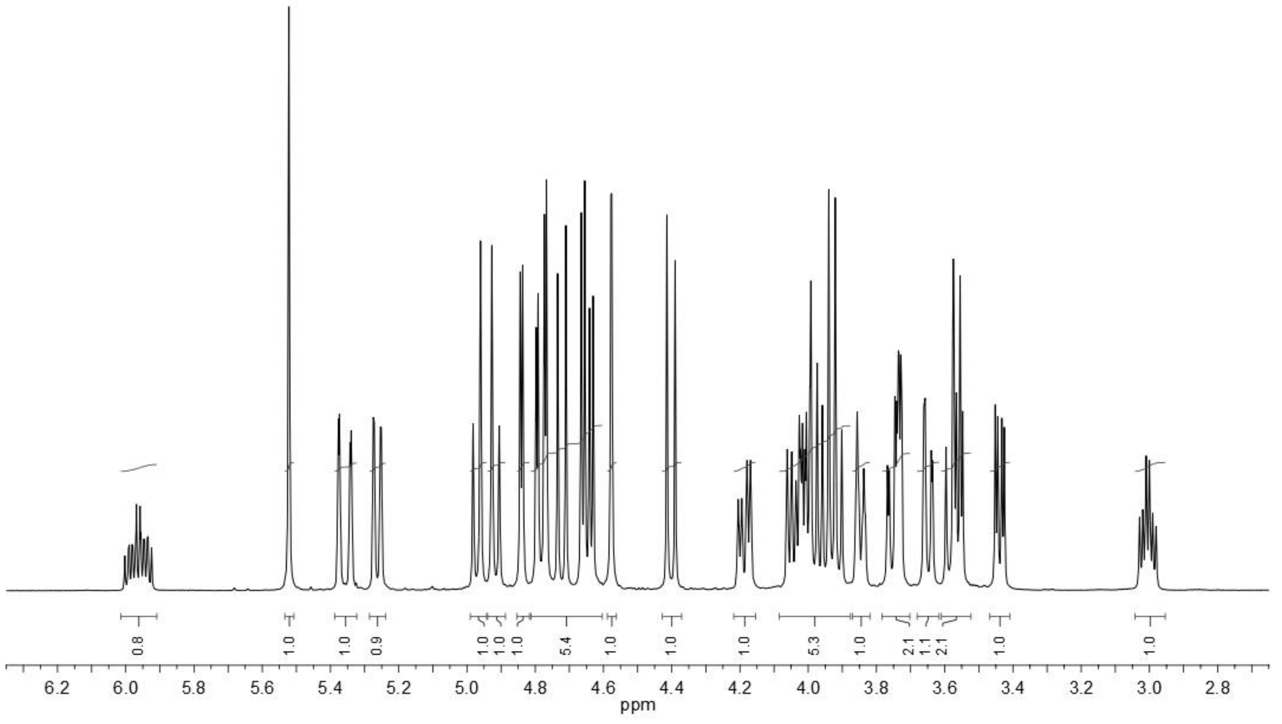

(47): ^{13}C NMR (126 MHz, CDCl$_3$)

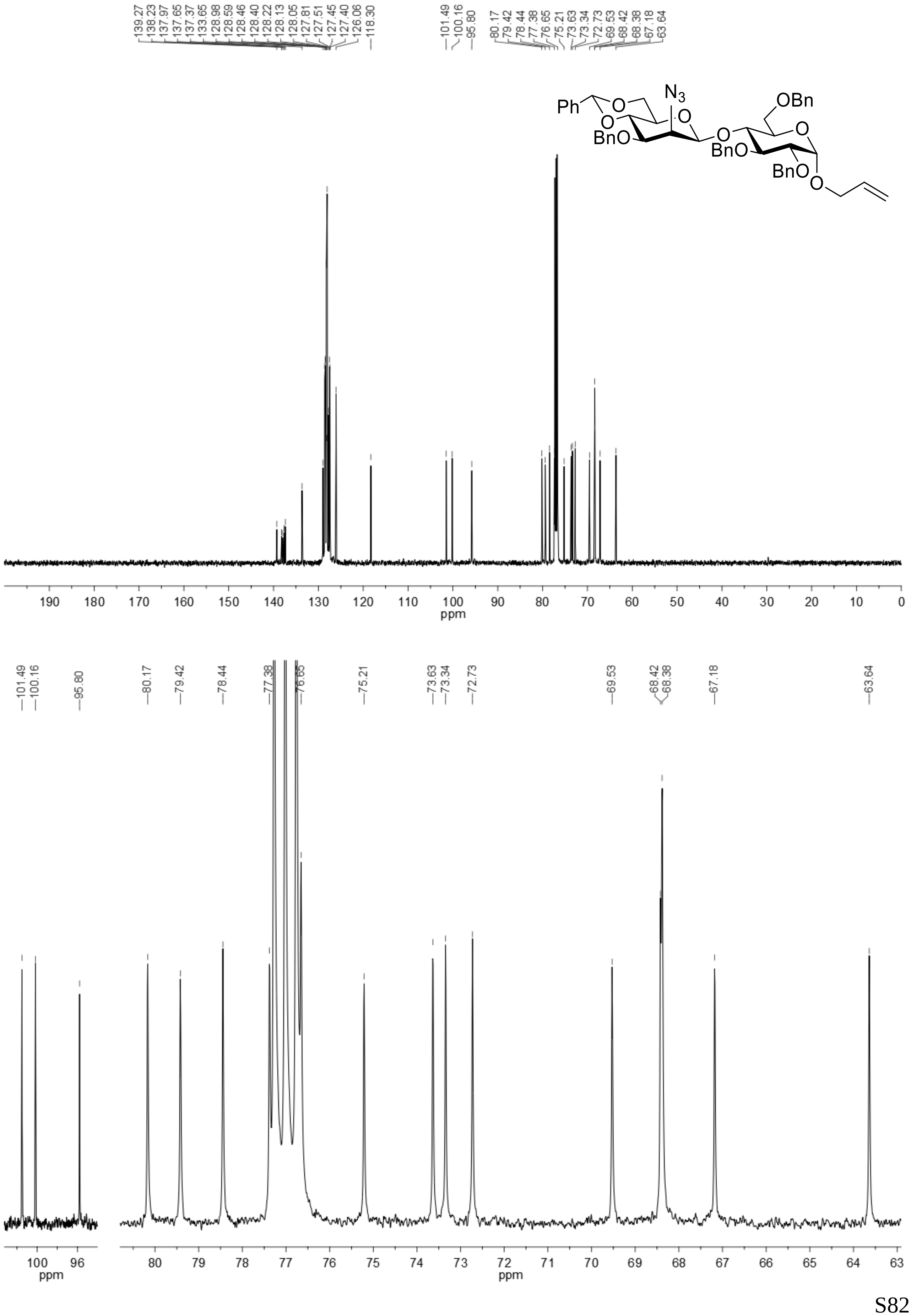

(48): ^{1}H NMR (500 MHz, CDCl$_3$)

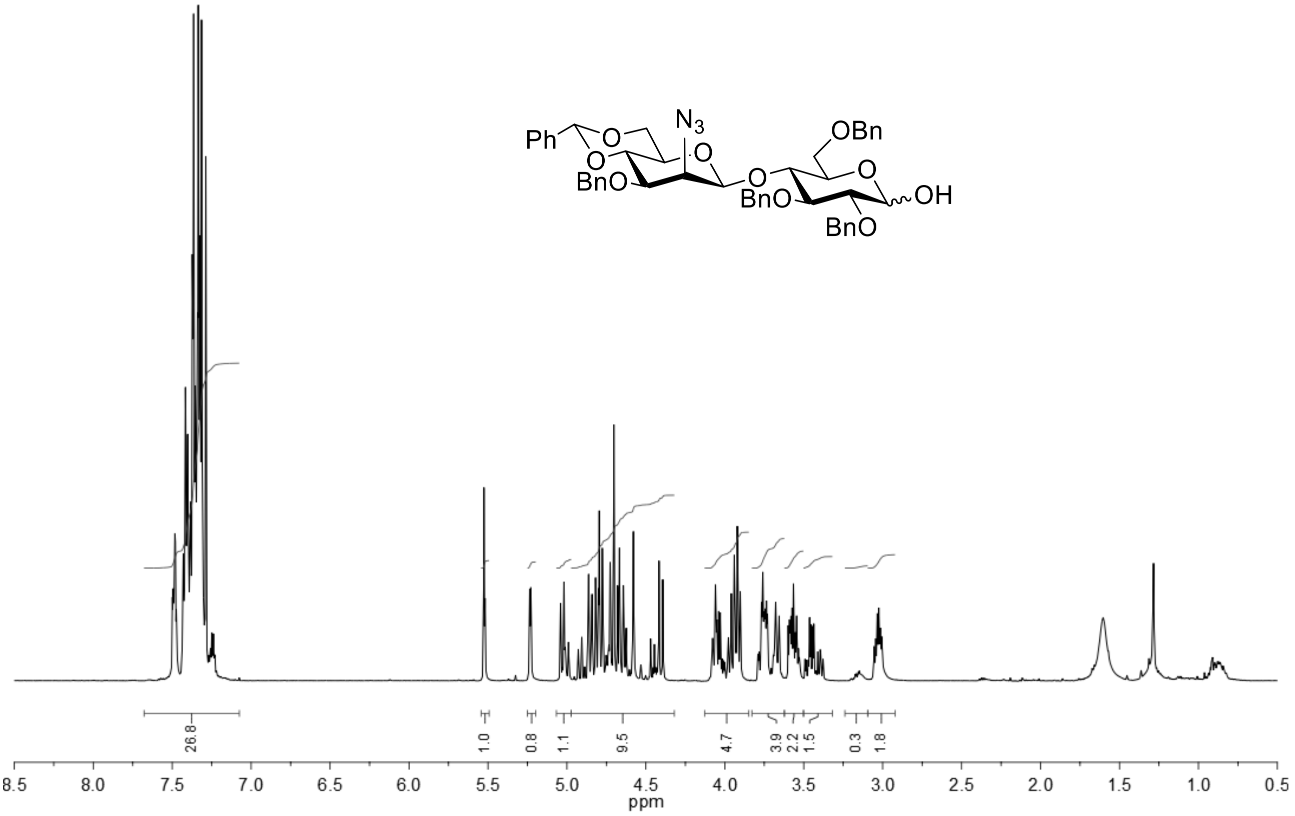

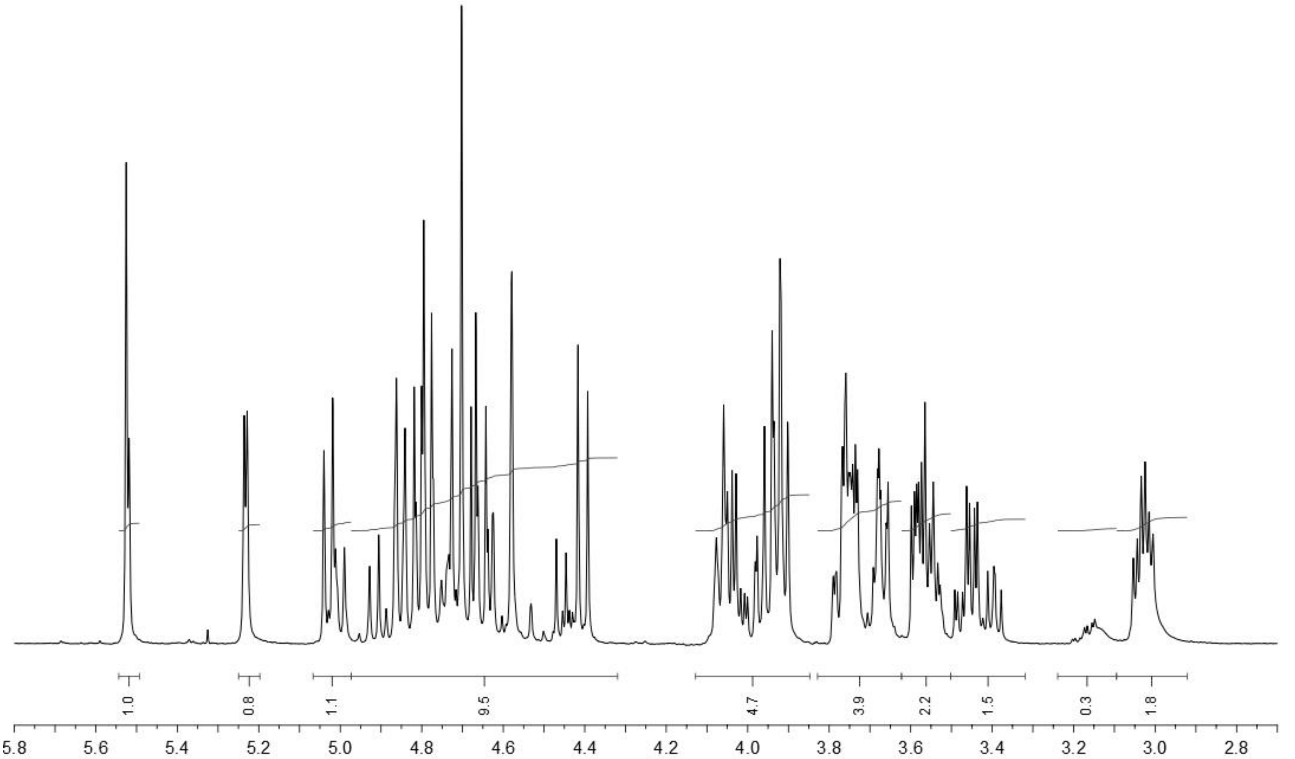

(48): ^{13}C NMR (126 MHz, CDCl$_3$)

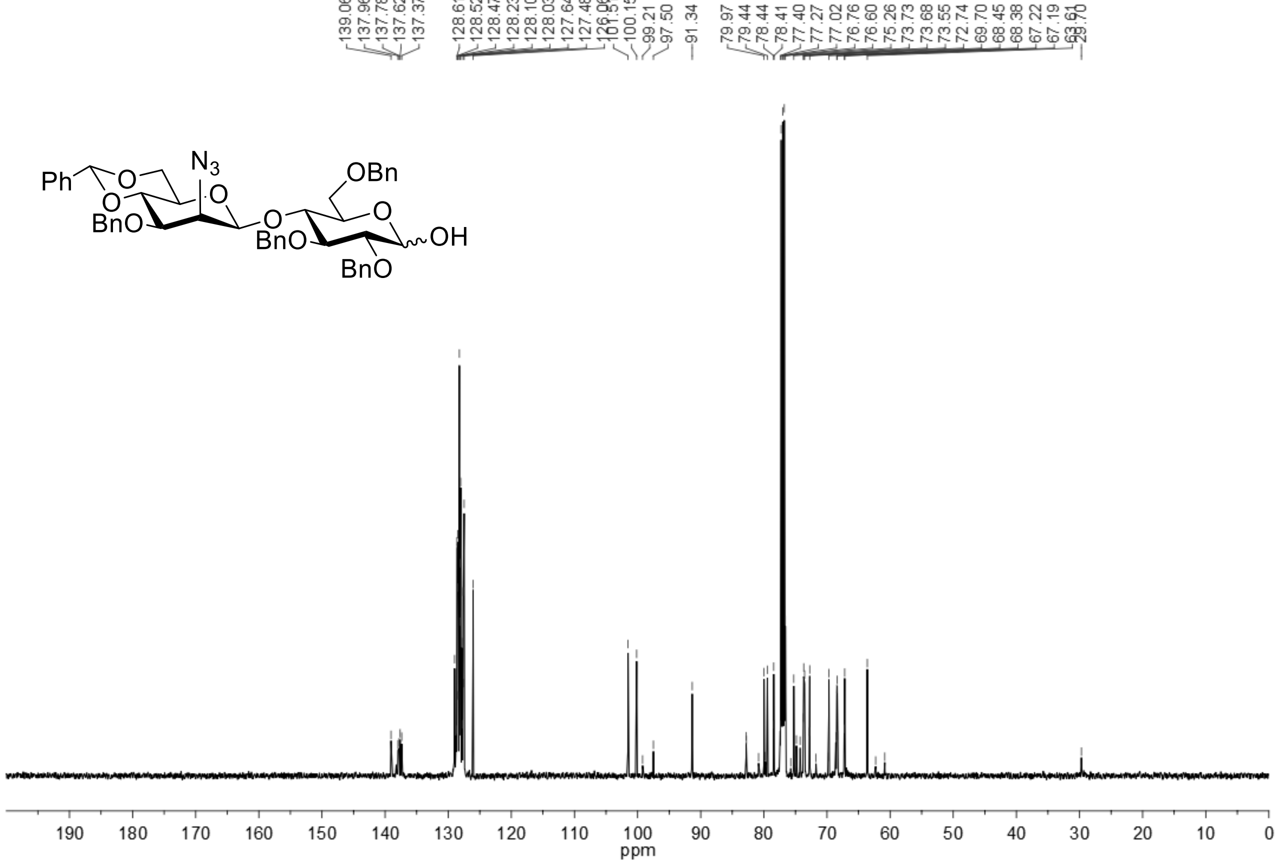

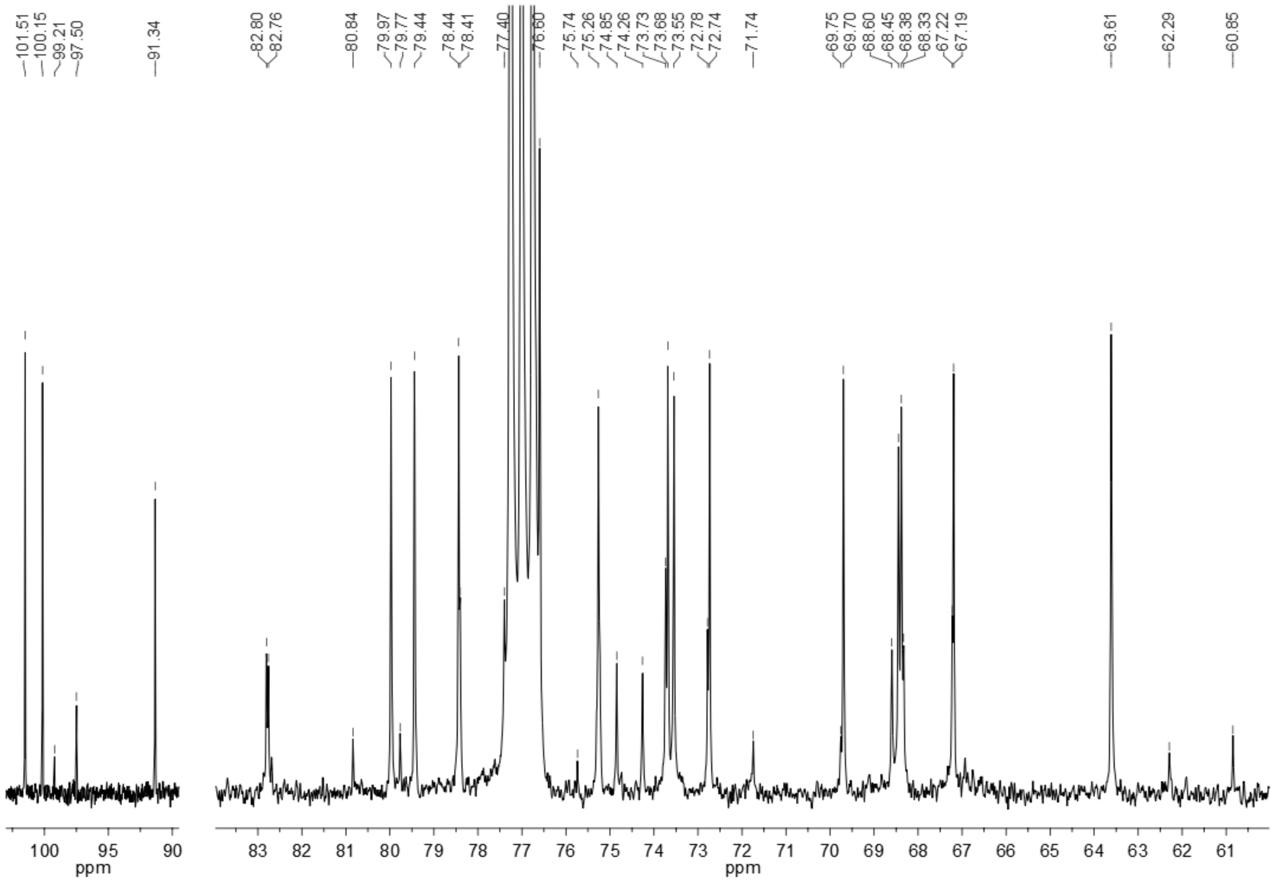

(10): ^{1}H NMR (500 MHz, $CDCl_3$)

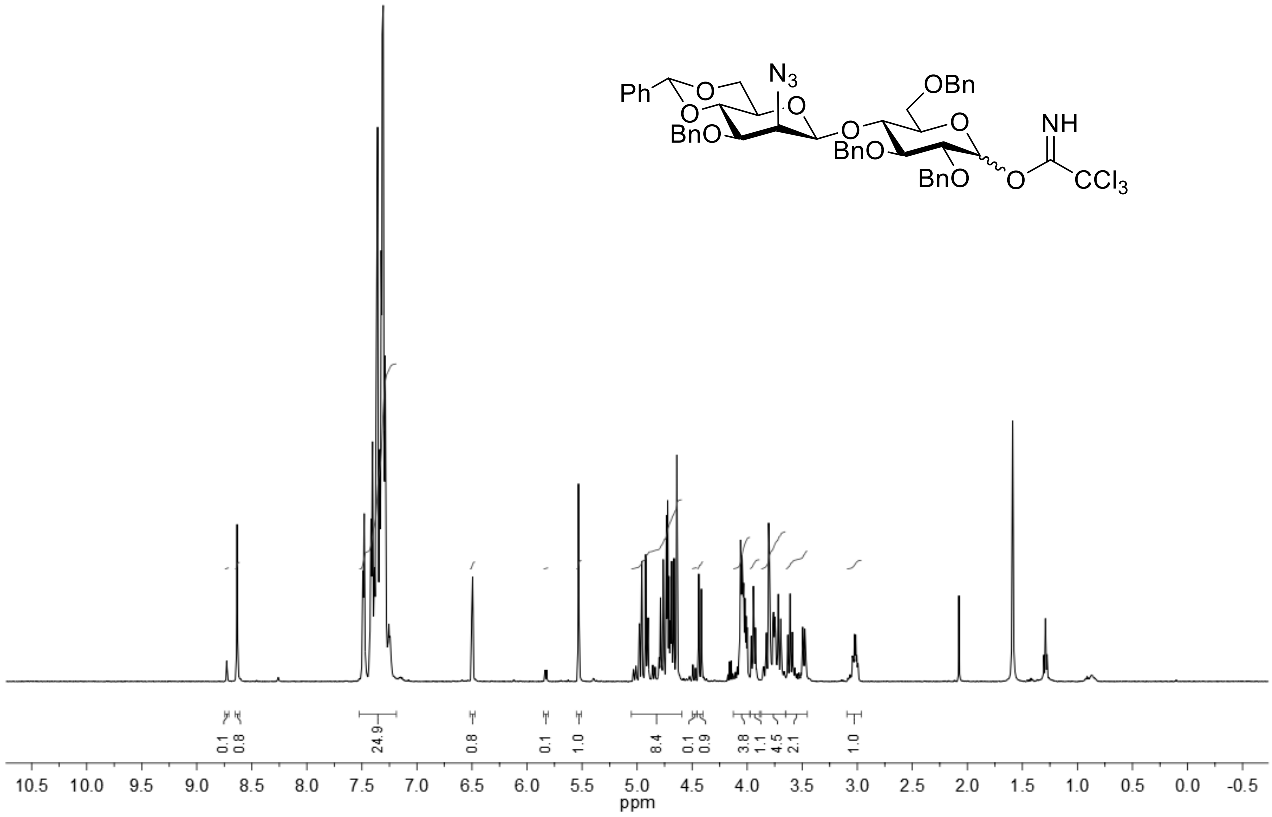

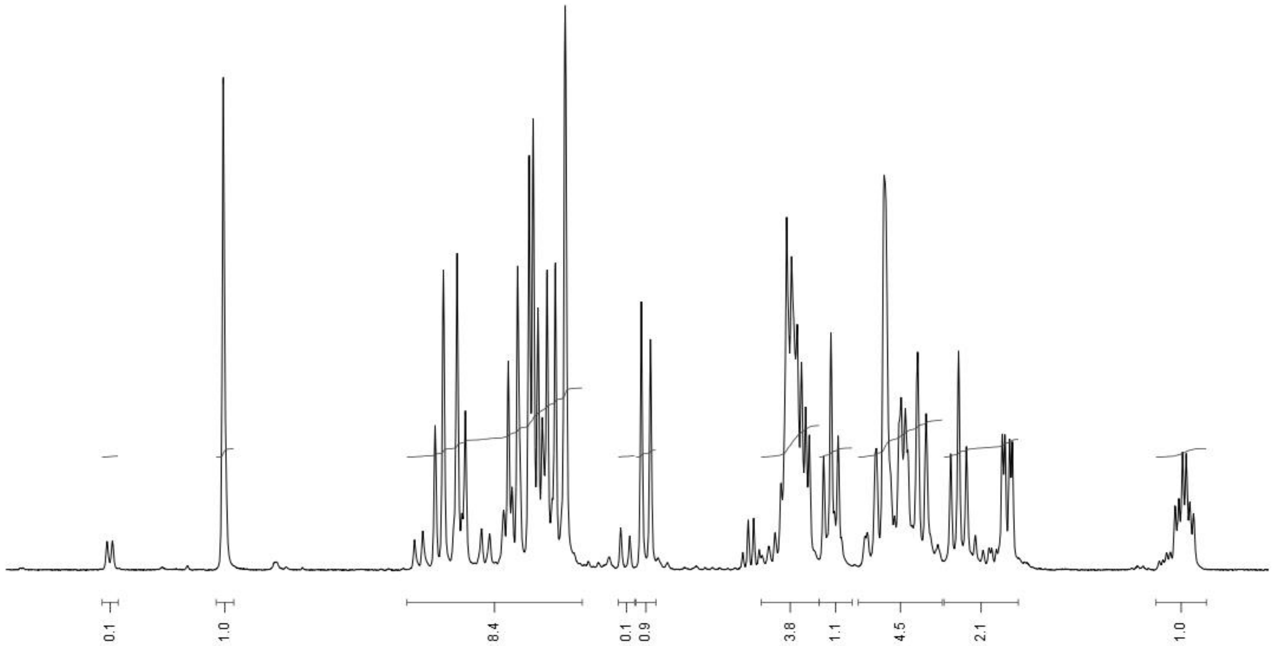

(10): ^{13}C NMR (126 MHz, $CDCl_3$)

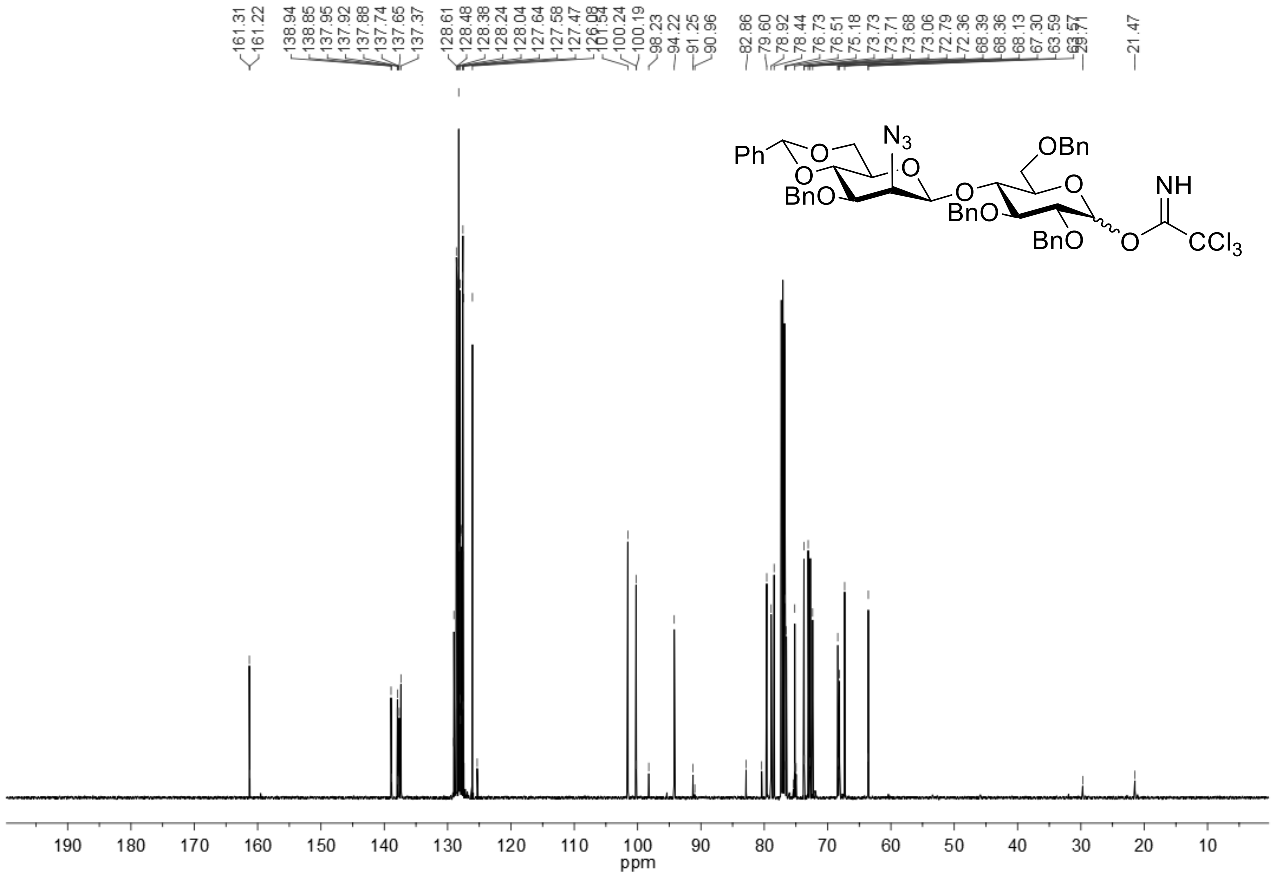

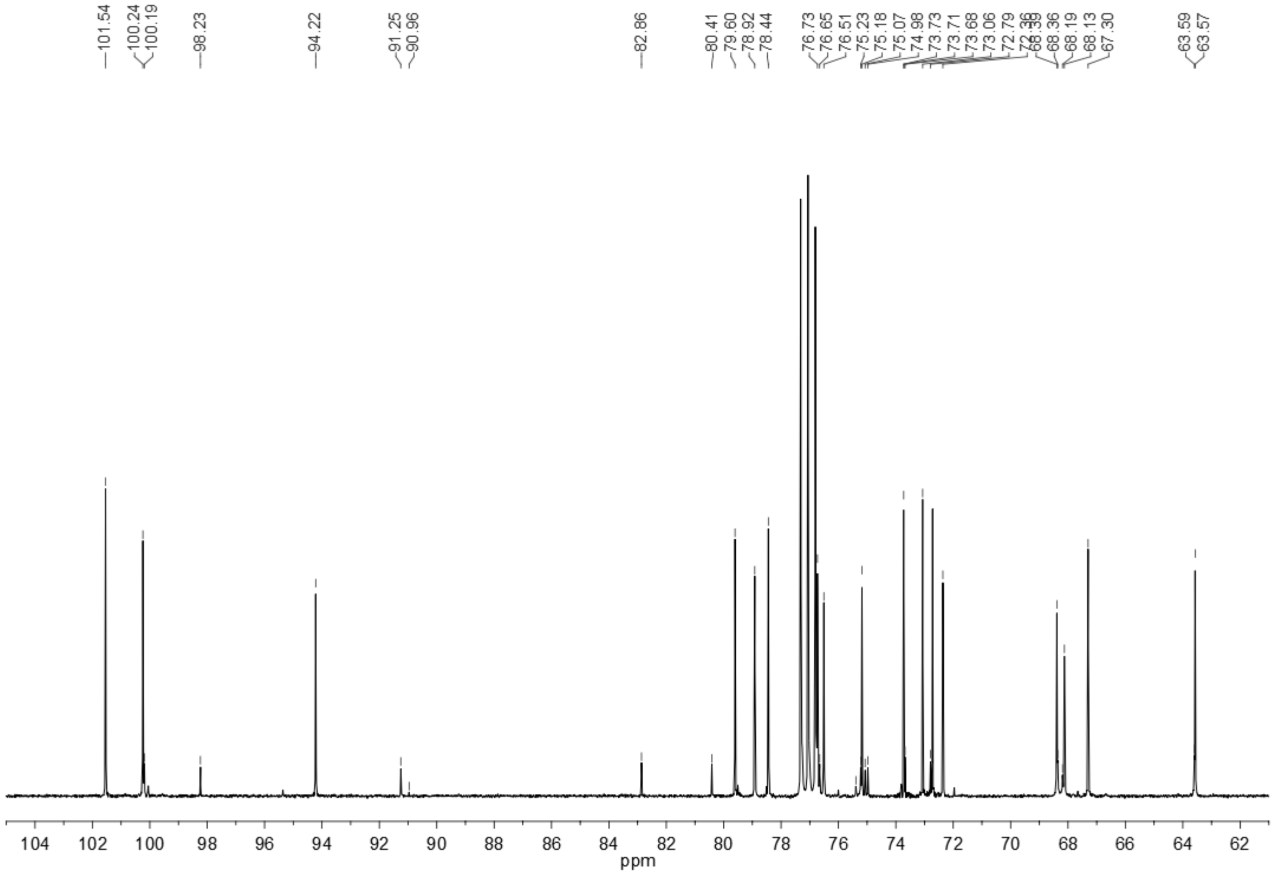

(49): ^{1}H NMR (500 MHz, $CDCl_3$)

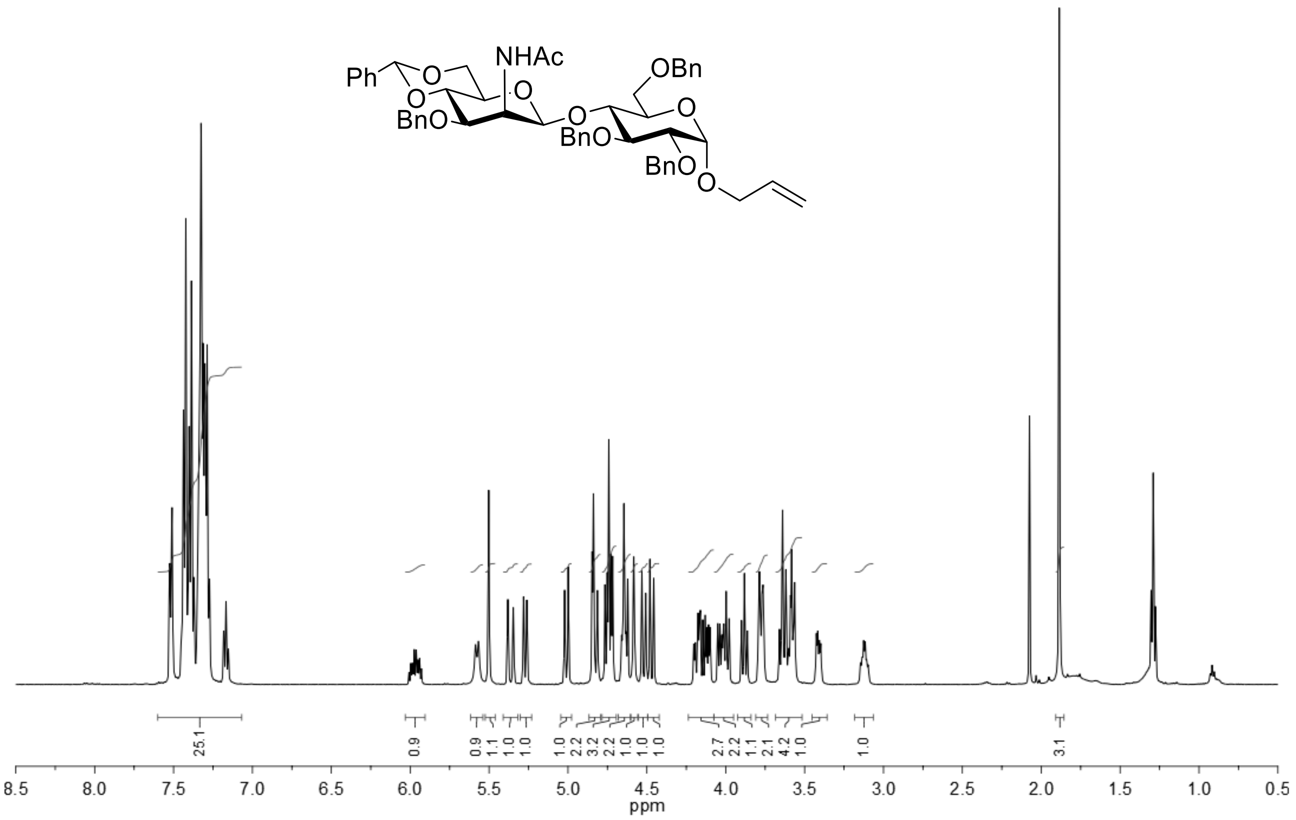

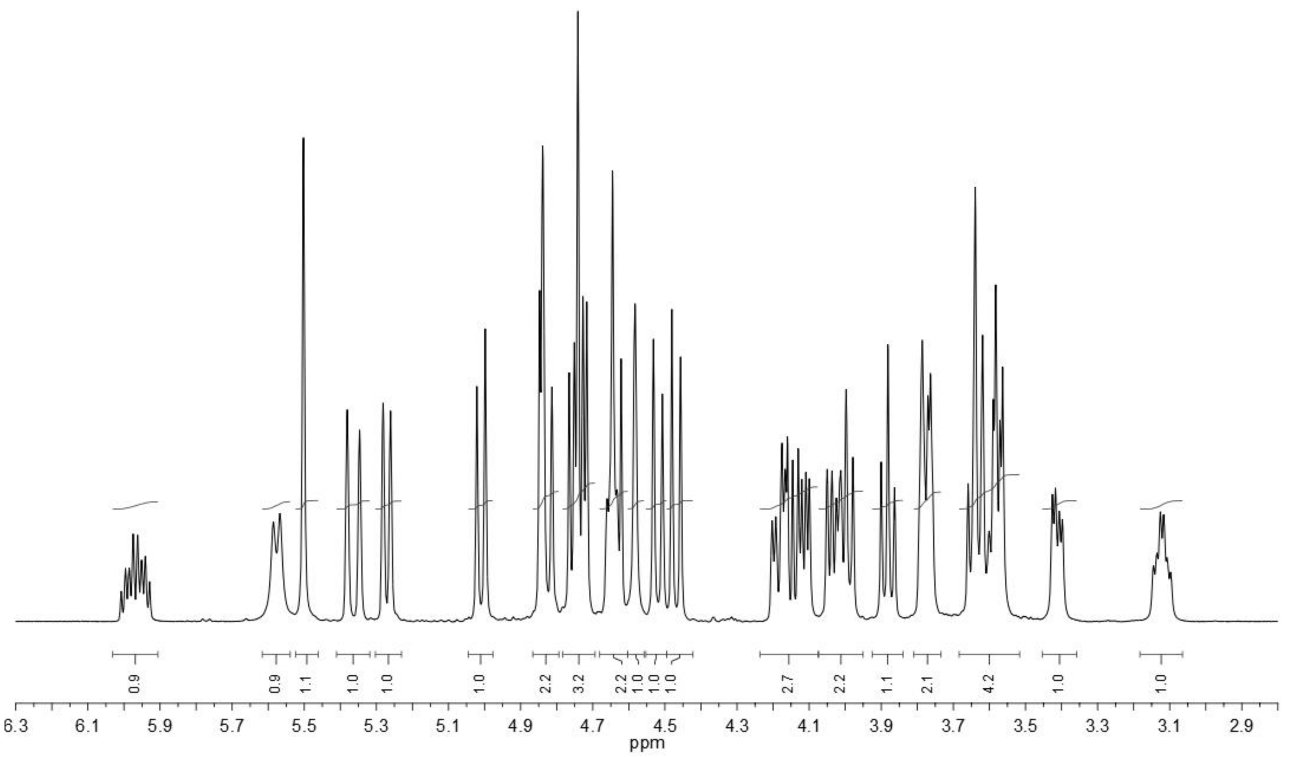

(49): ^{13}C NMR (126 MHz, $CDCl_3$)

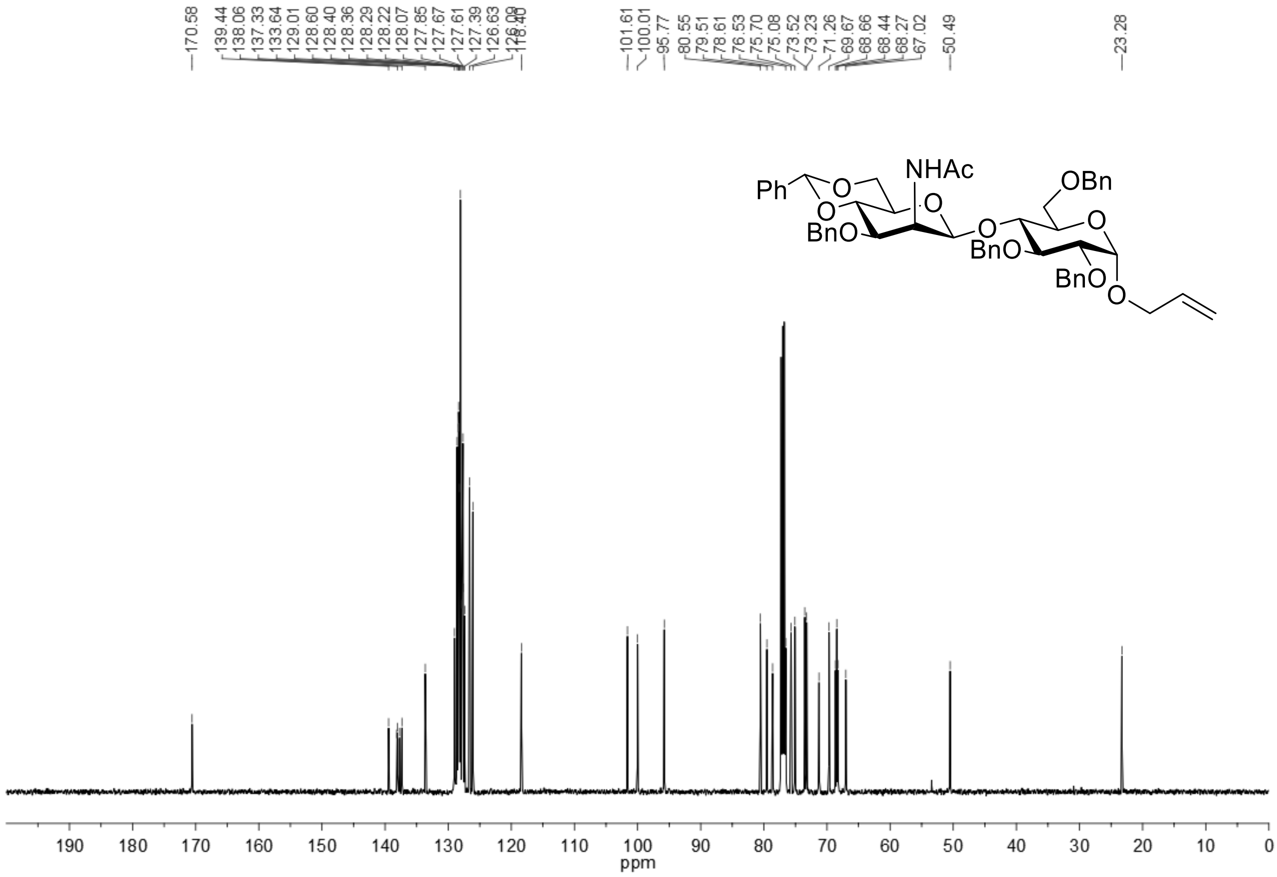

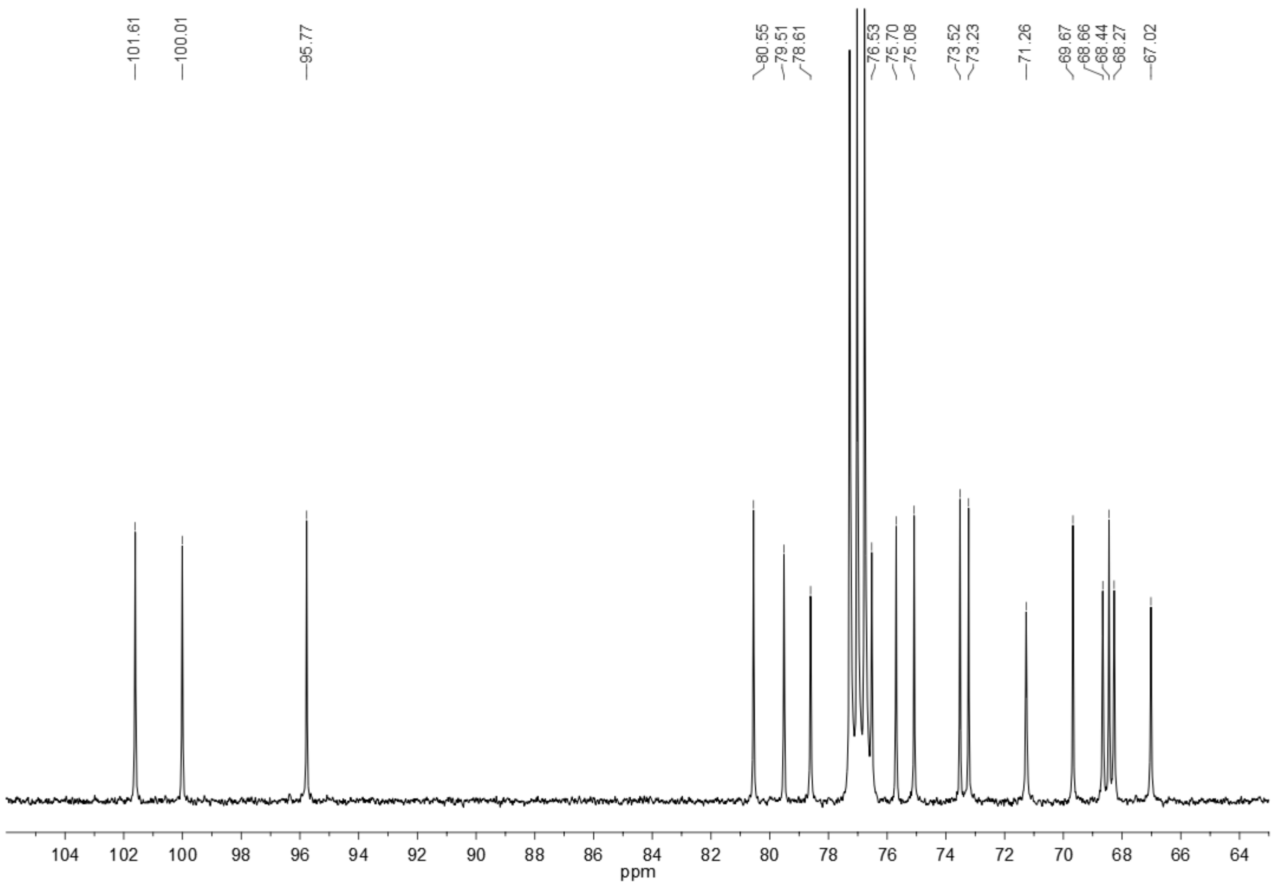

(11): ^{1}H NMR (500 MHz, $CDCl_3$)

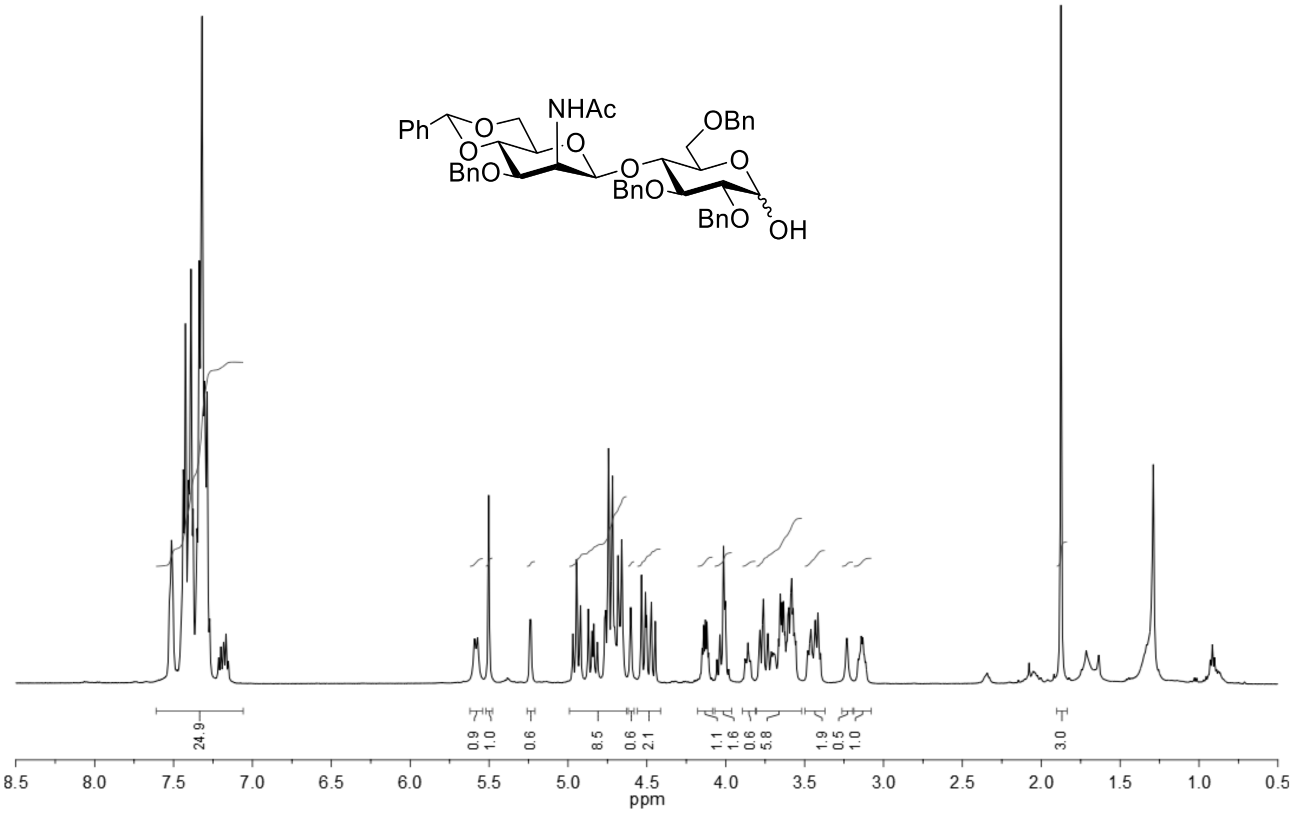

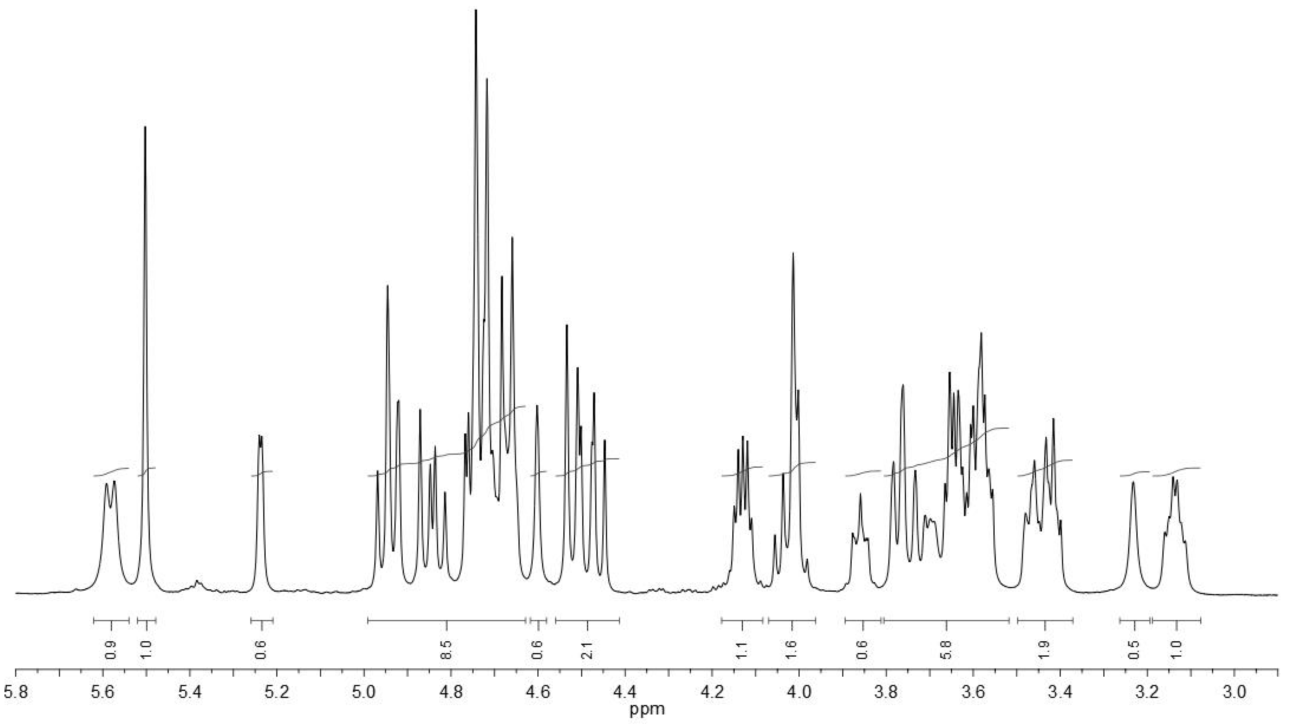

(11): ^{13}C NMR (126 MHz, $CDCl_3$)

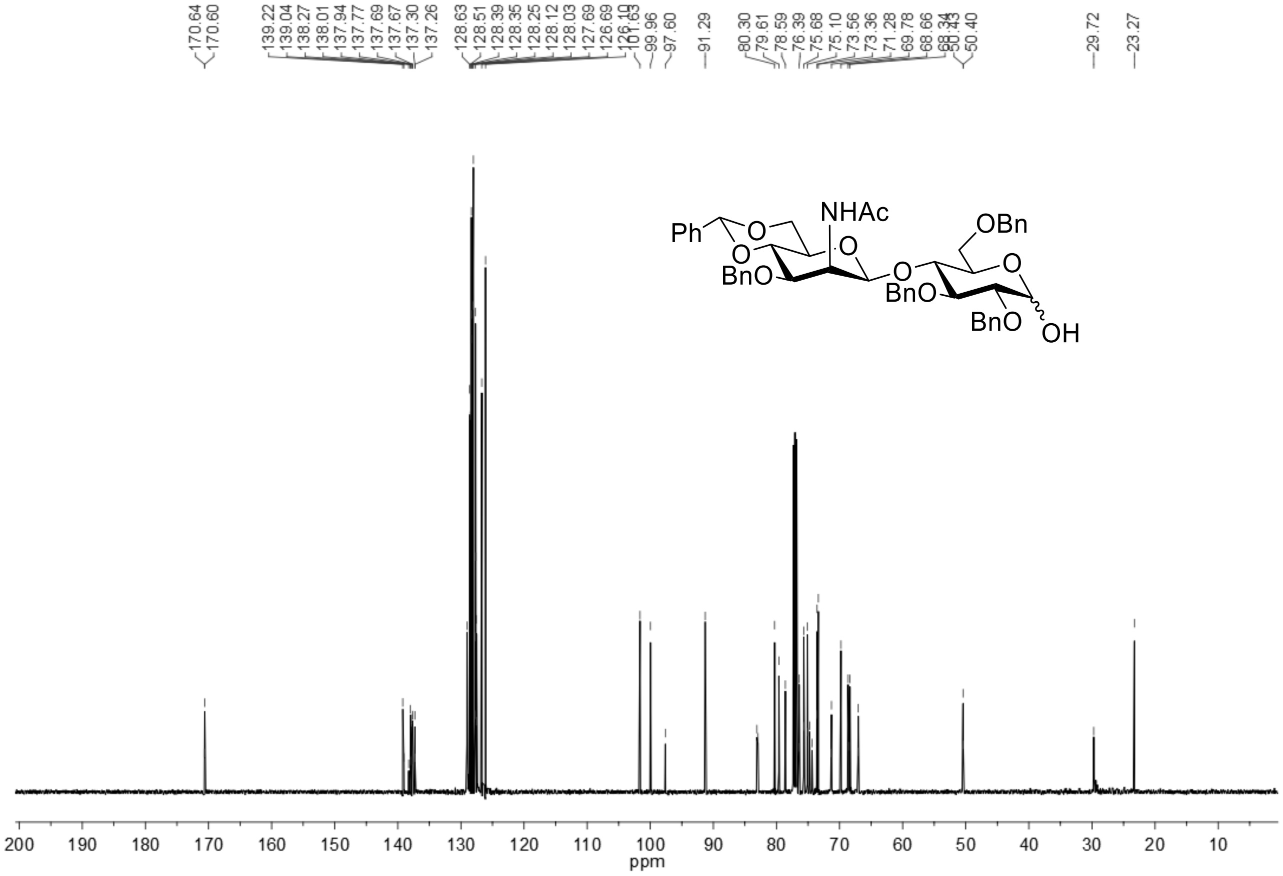

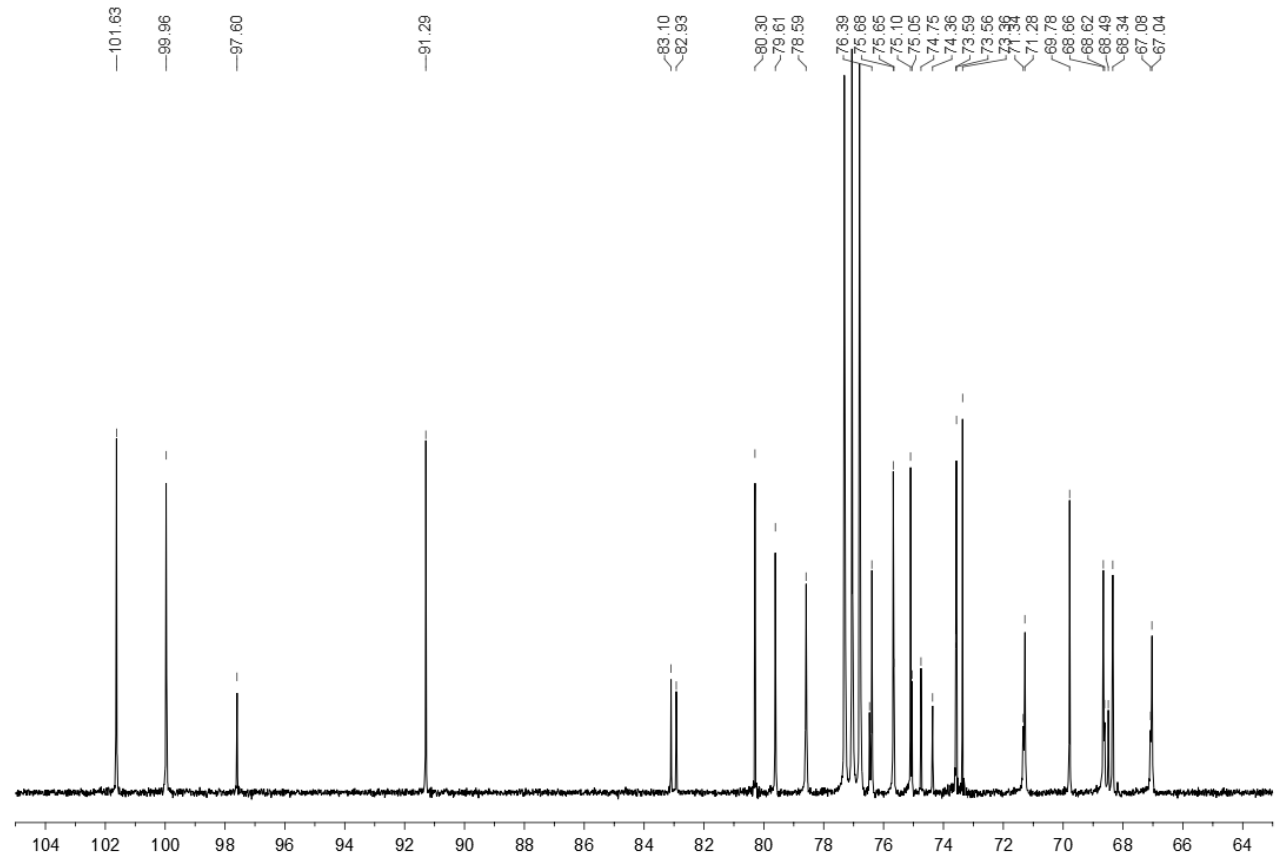

(12): ^{1}H NMR (500 MHz, $CDCl_3$)

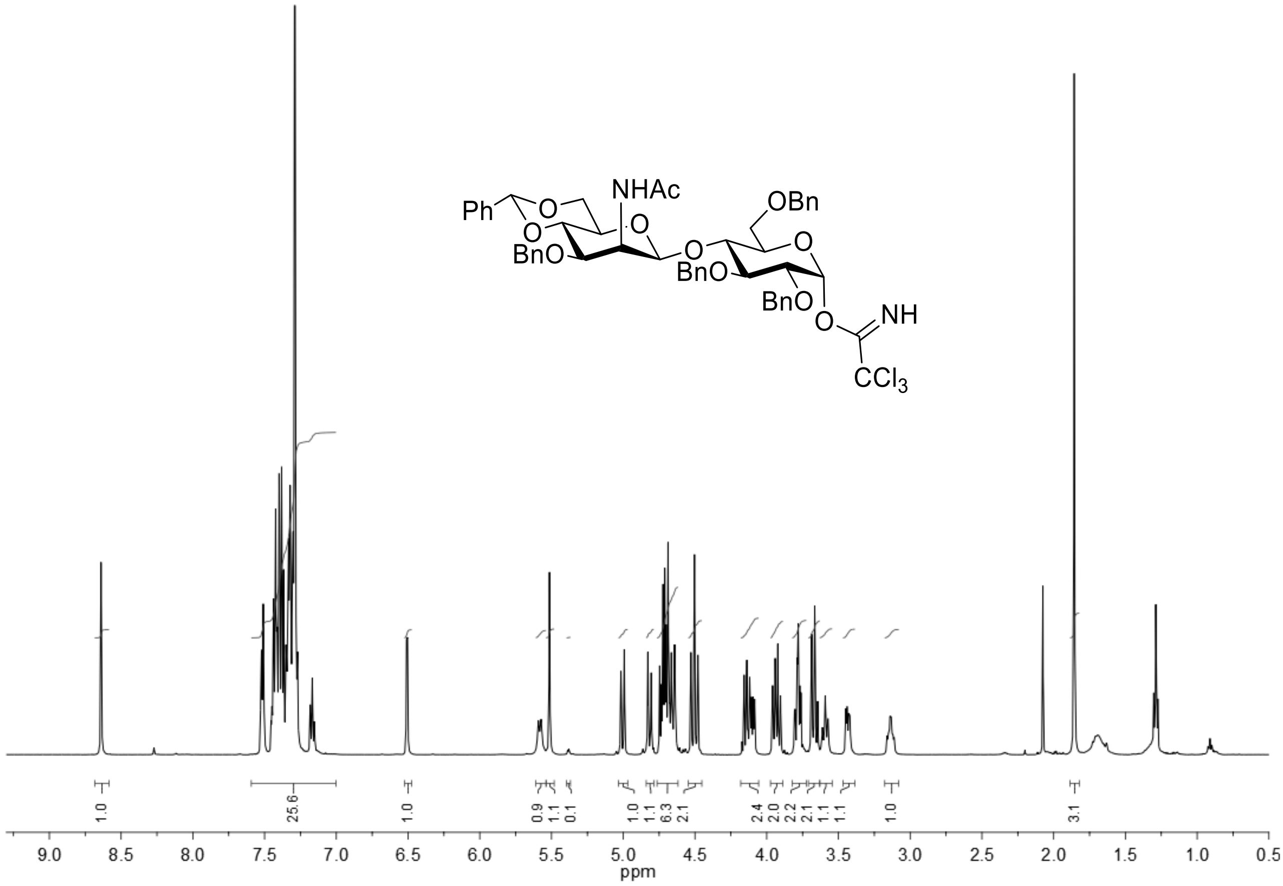

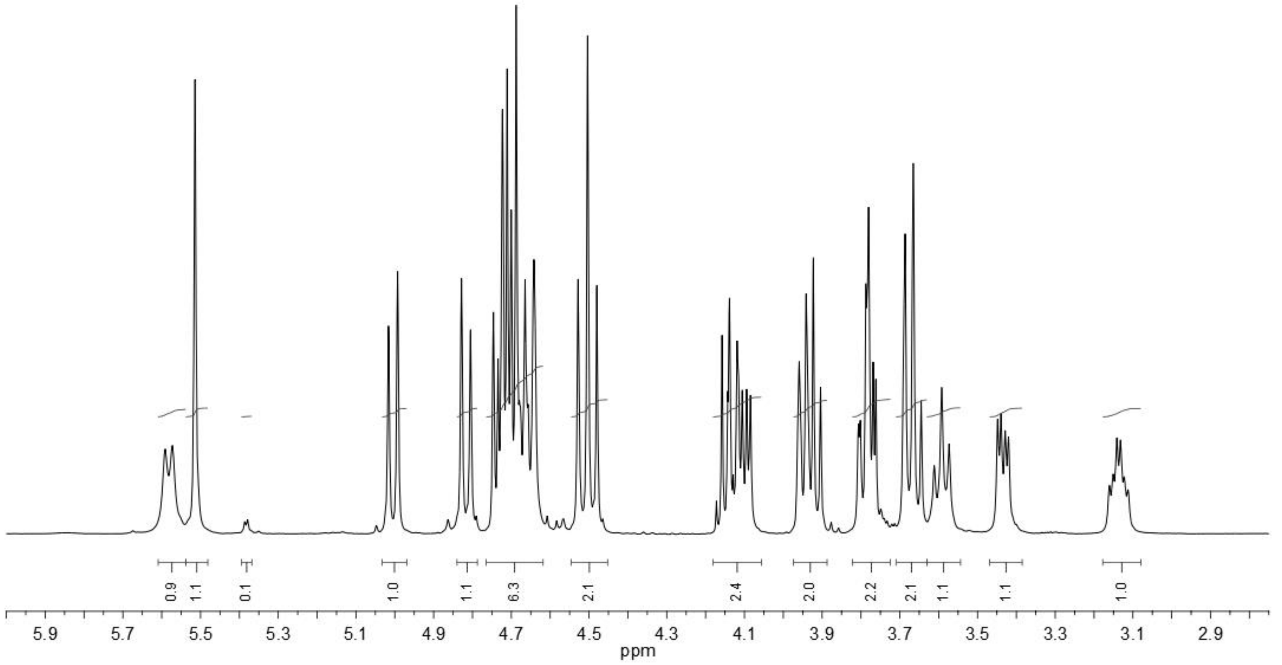

(12): ^{13}C NMR (126 MHz, $CDCl_3$)

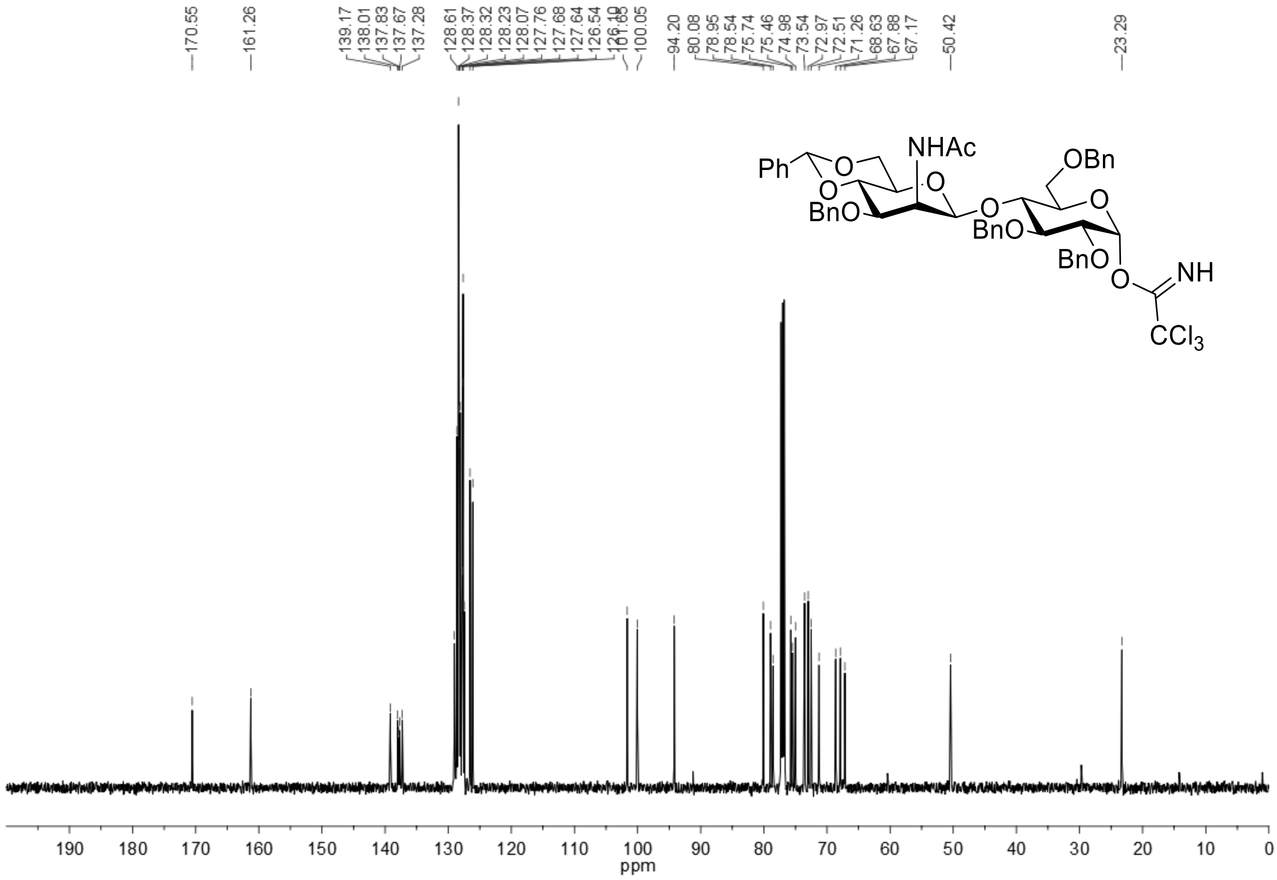

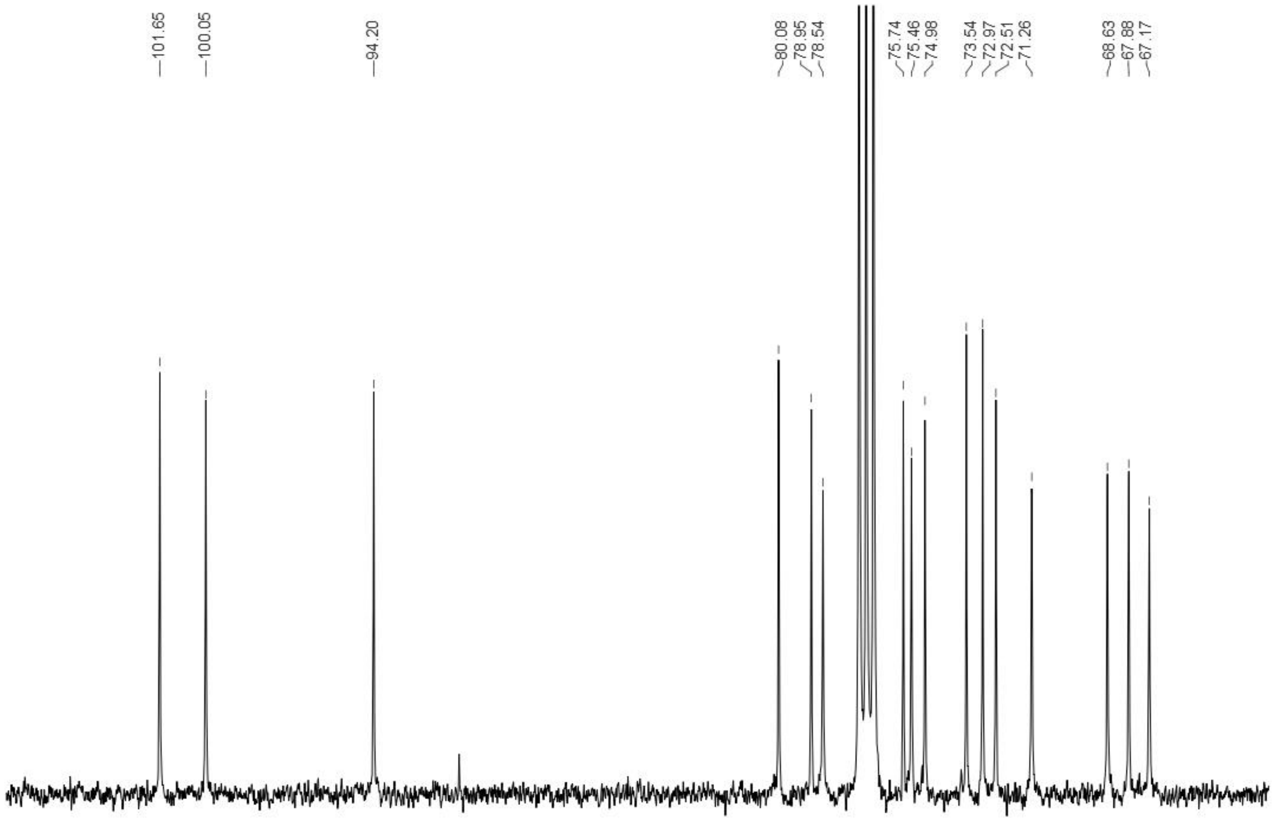

(14): ^{1}H NMR (500 MHz, CDCl$_3$)

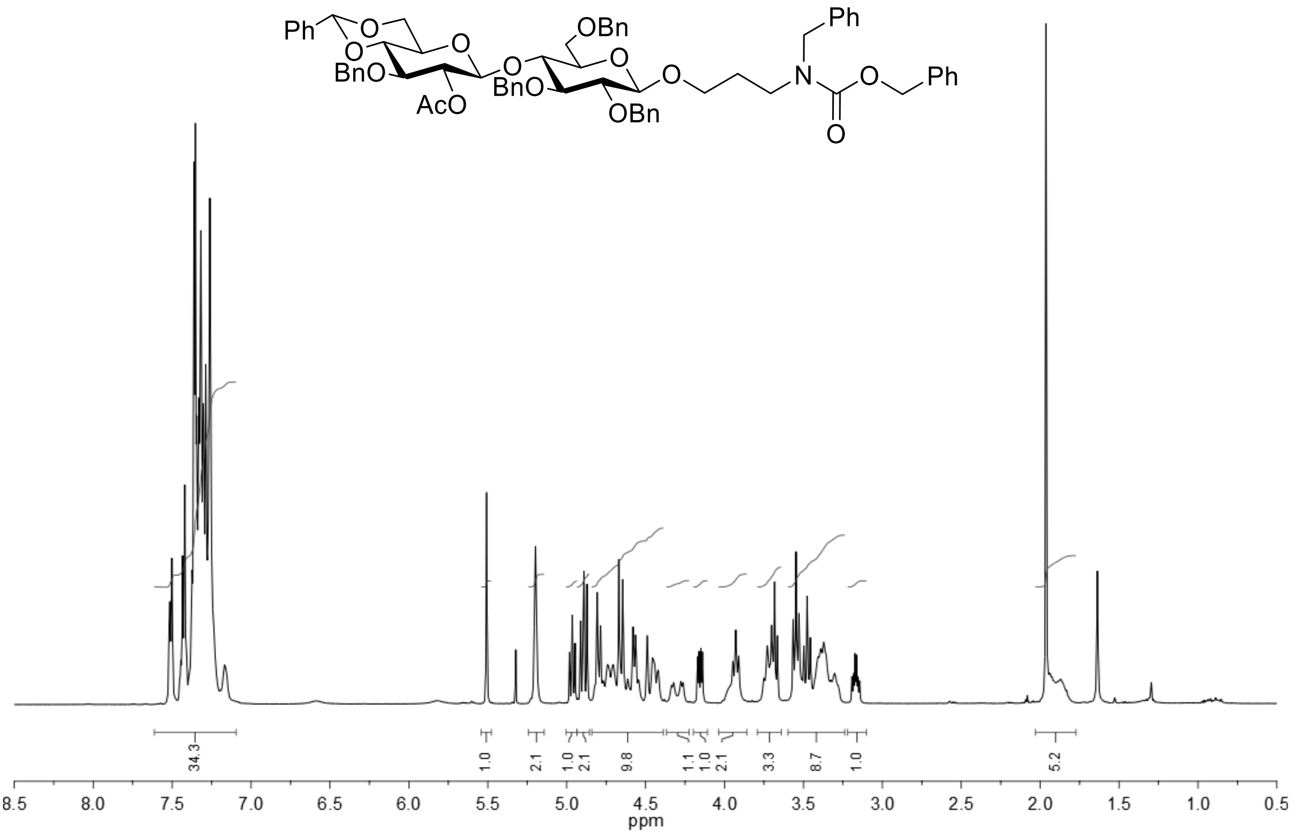

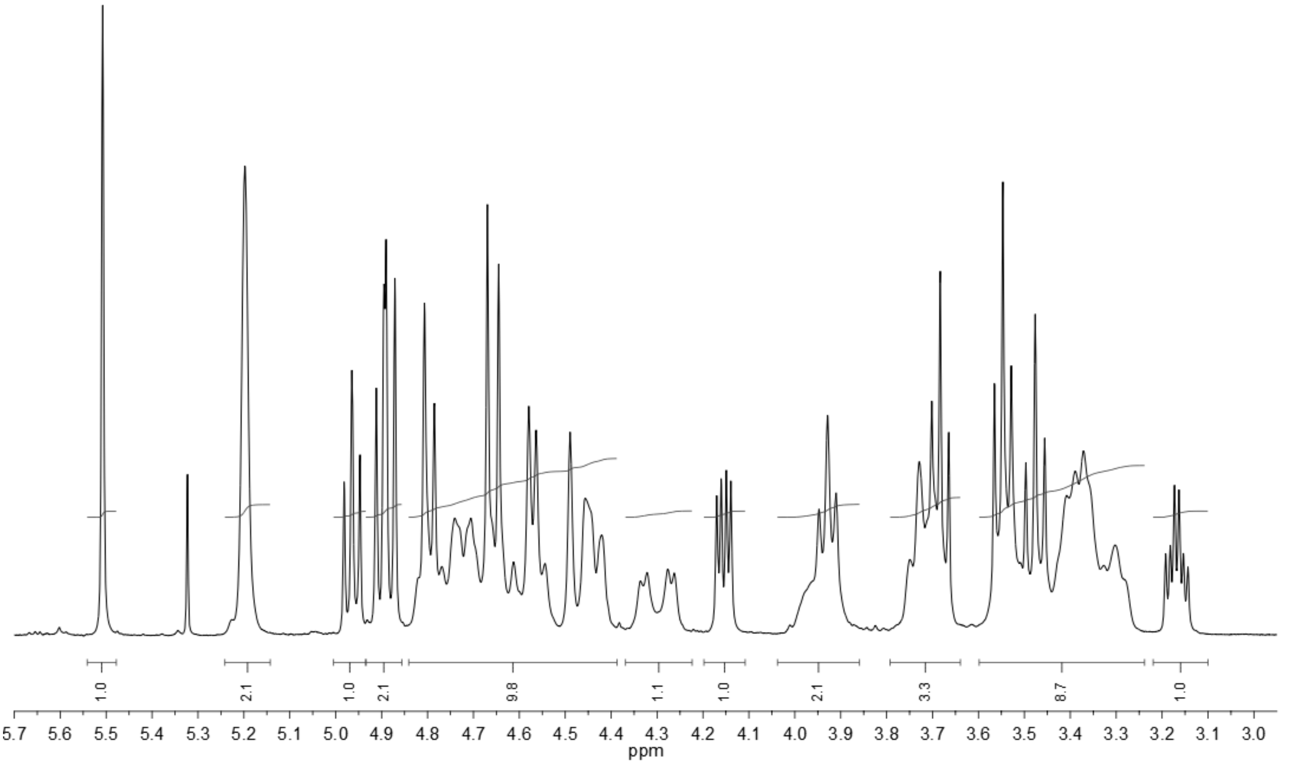

(14): ^{13}C NMR (126 MHz, $CDCl_3$)

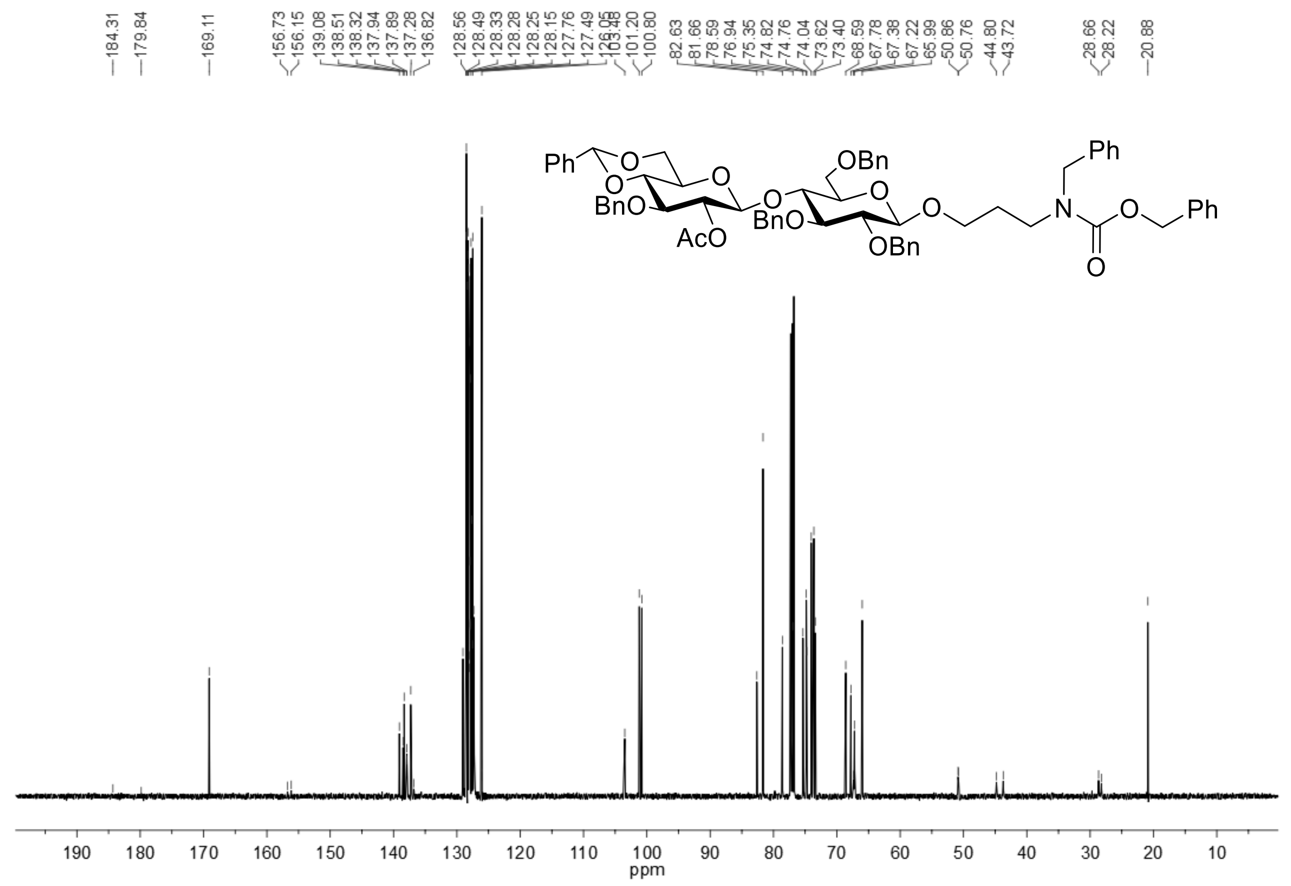

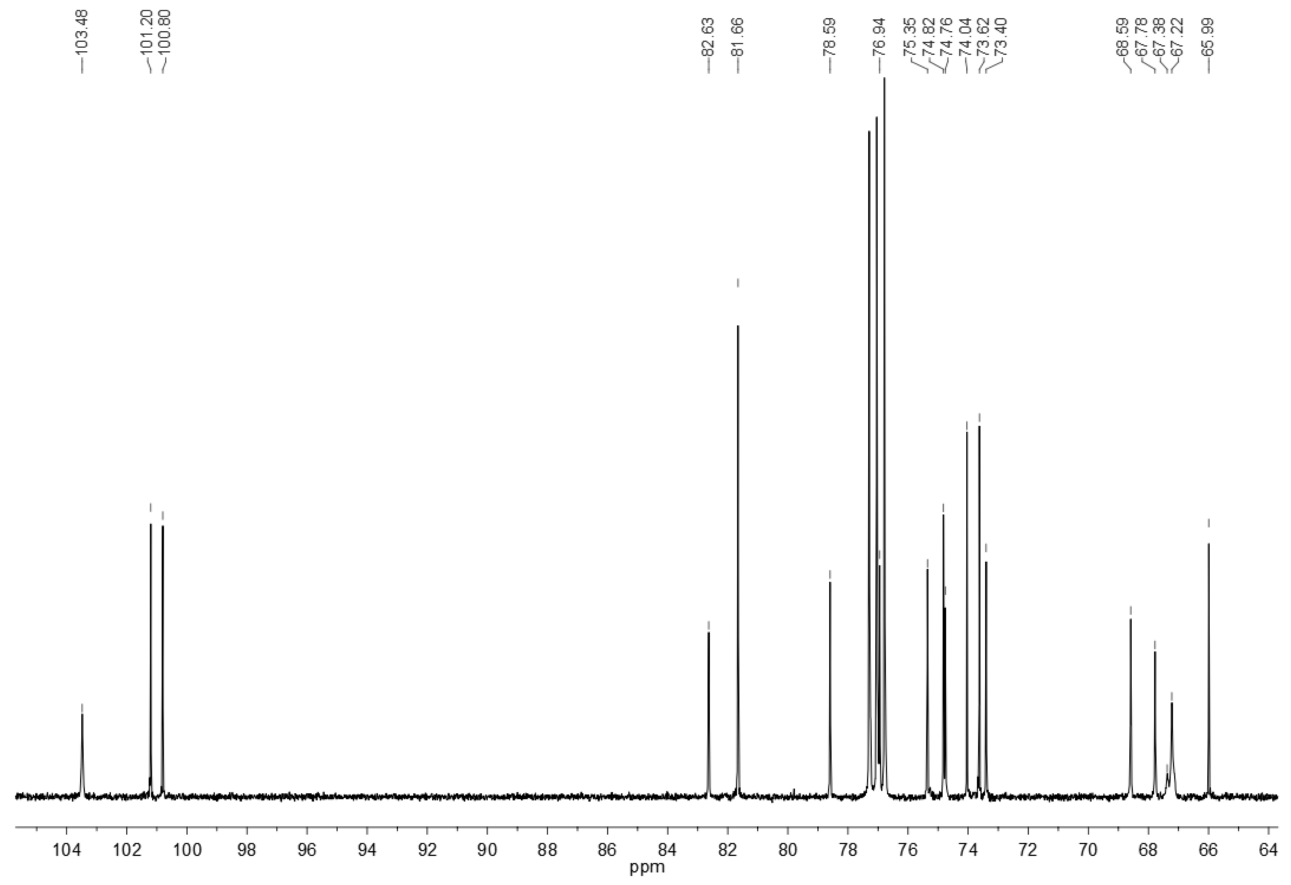

(15): ^{1}H NMR (500 MHz, $CDCl_3$)

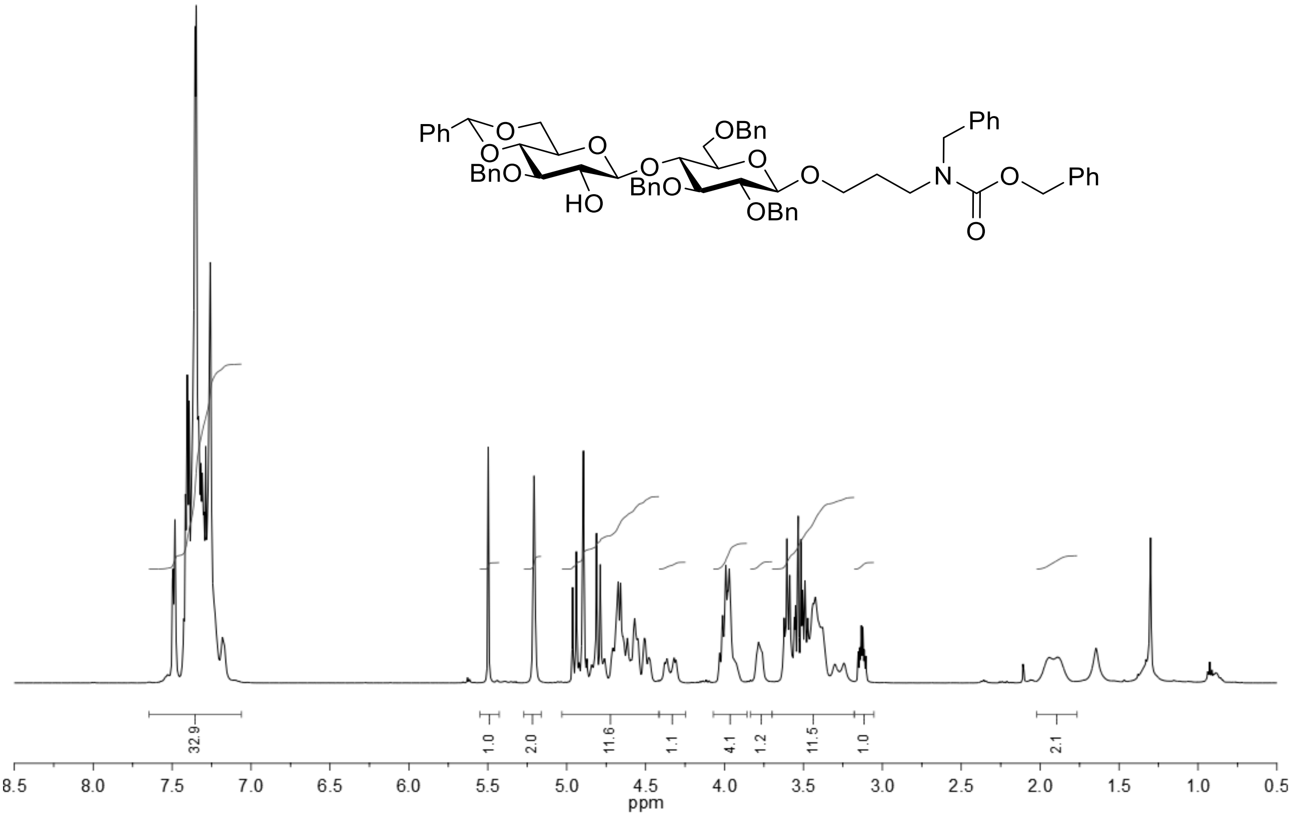

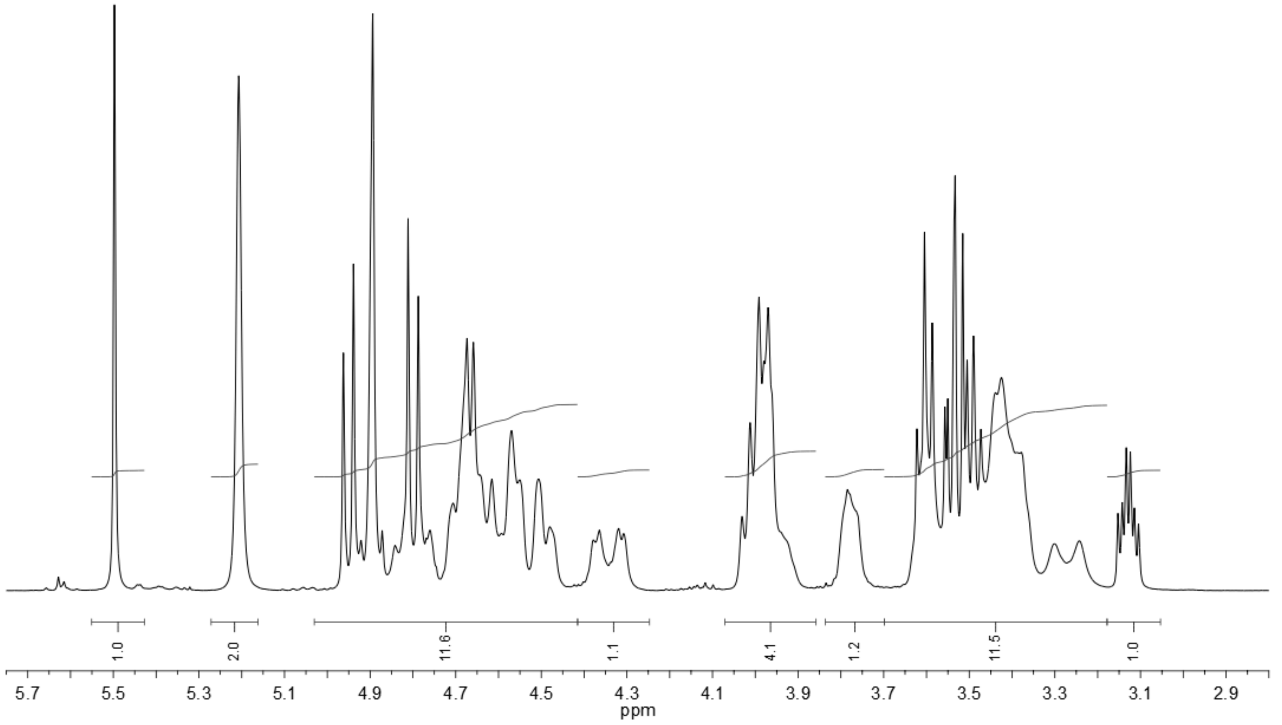

(15): ^{13}C NMR (126 MHz, CDCl$_3$)

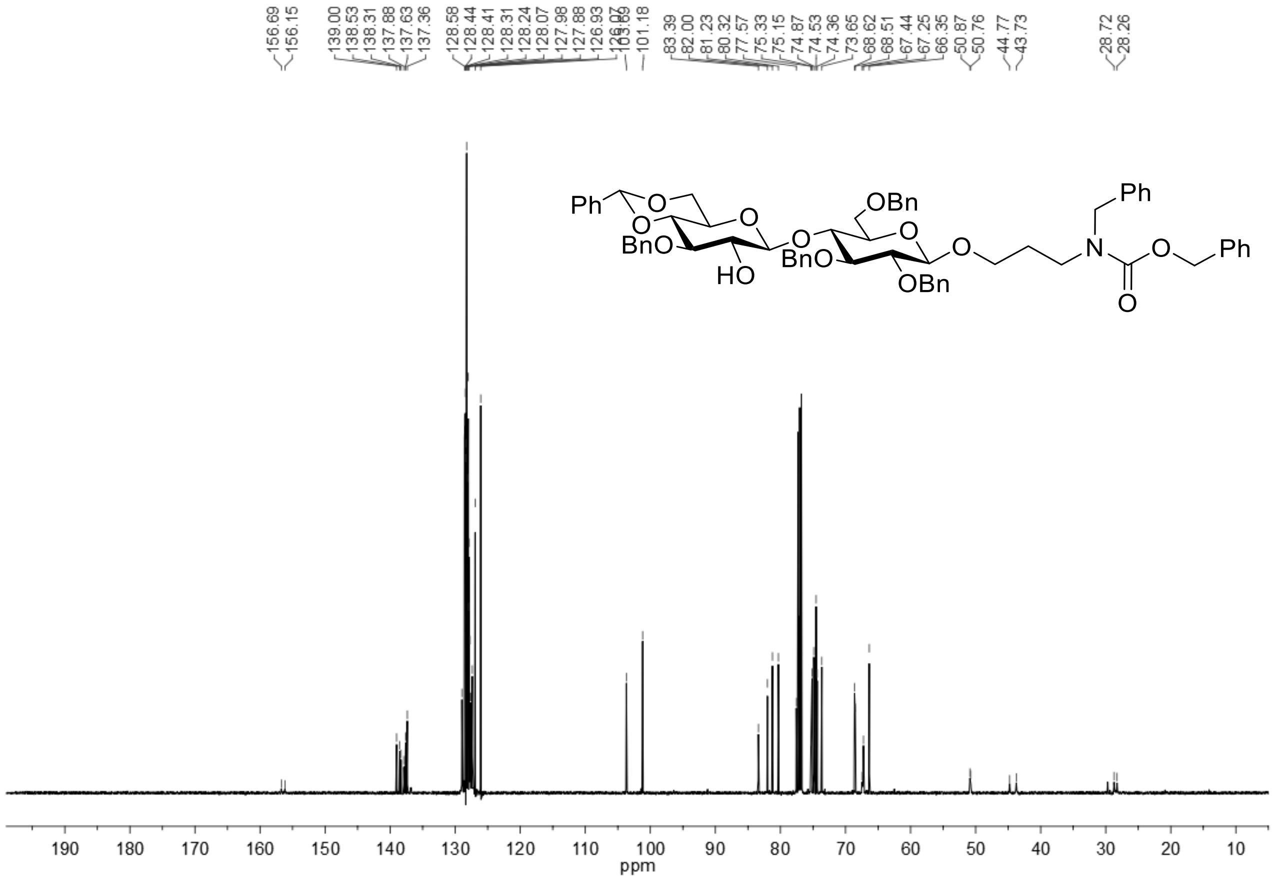

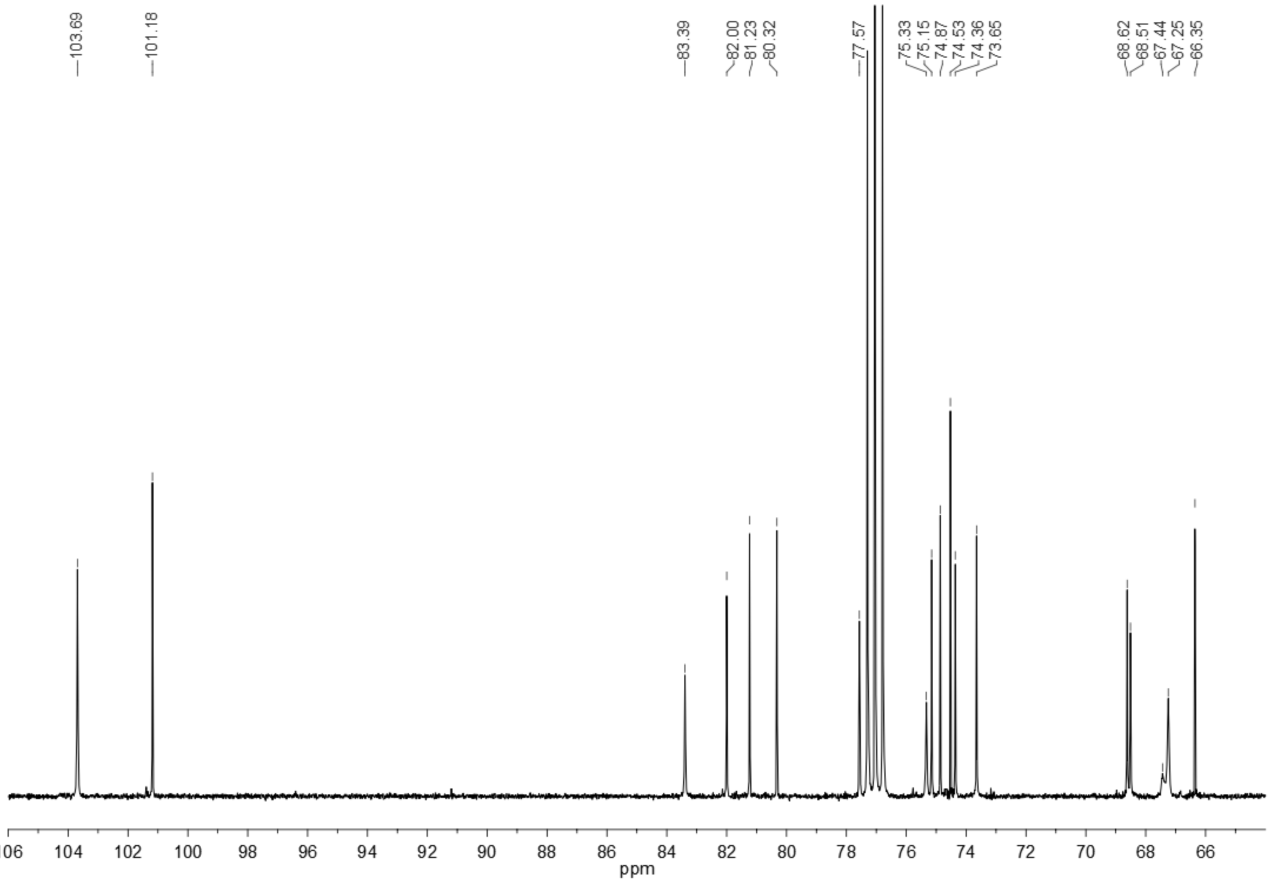

(16): ^{1}H NMR (500 MHz, $CDCl_3$)

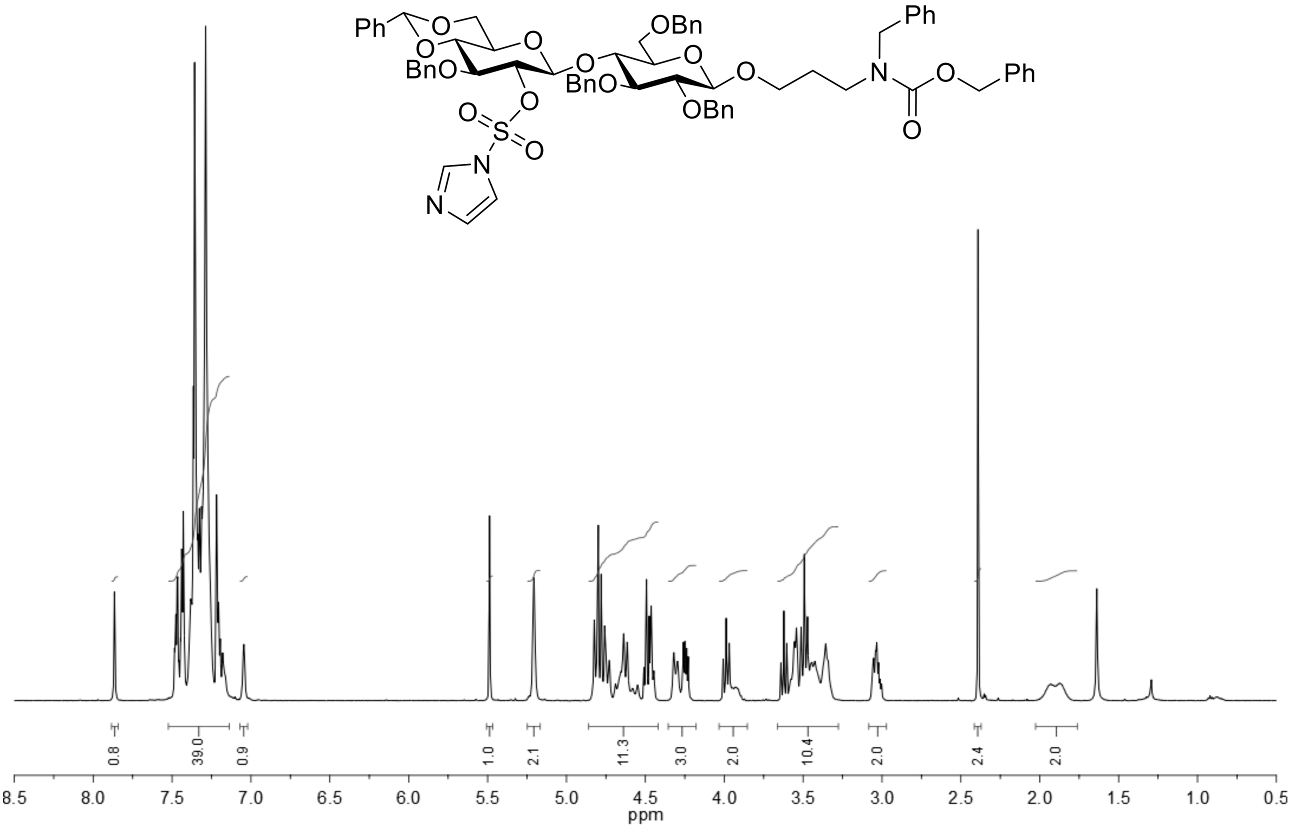

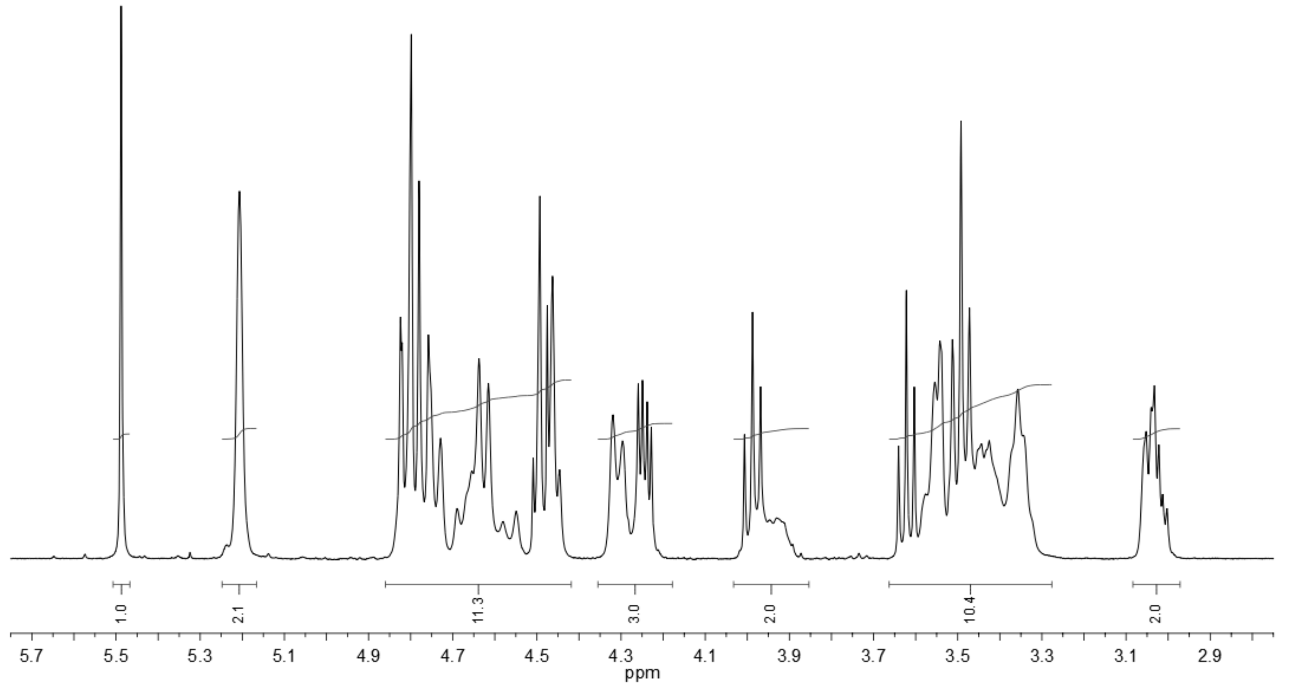

(16): ^{13}C NMR (126 MHz, CDCl$_3$)

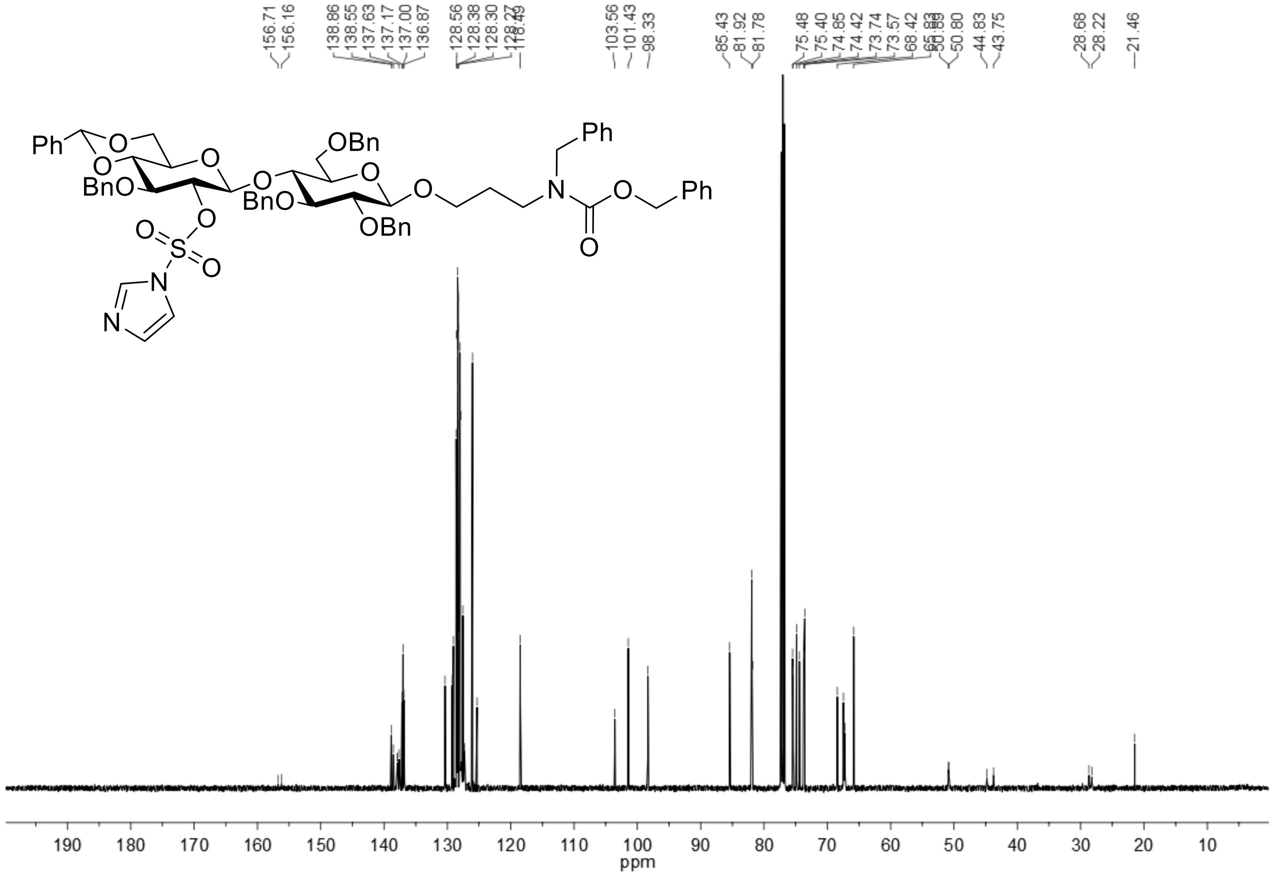

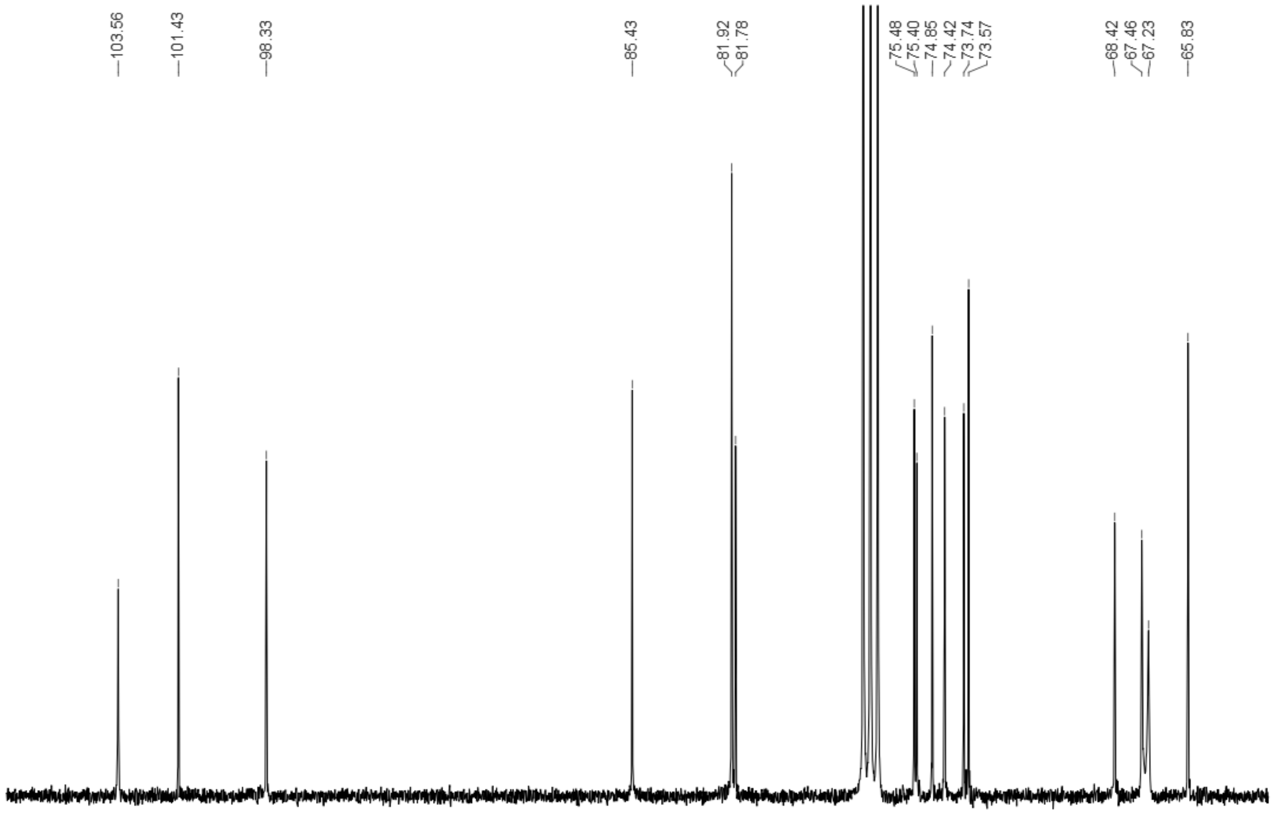

(17): ^{1}H NMR (500 MHz, CDCl$_3$)

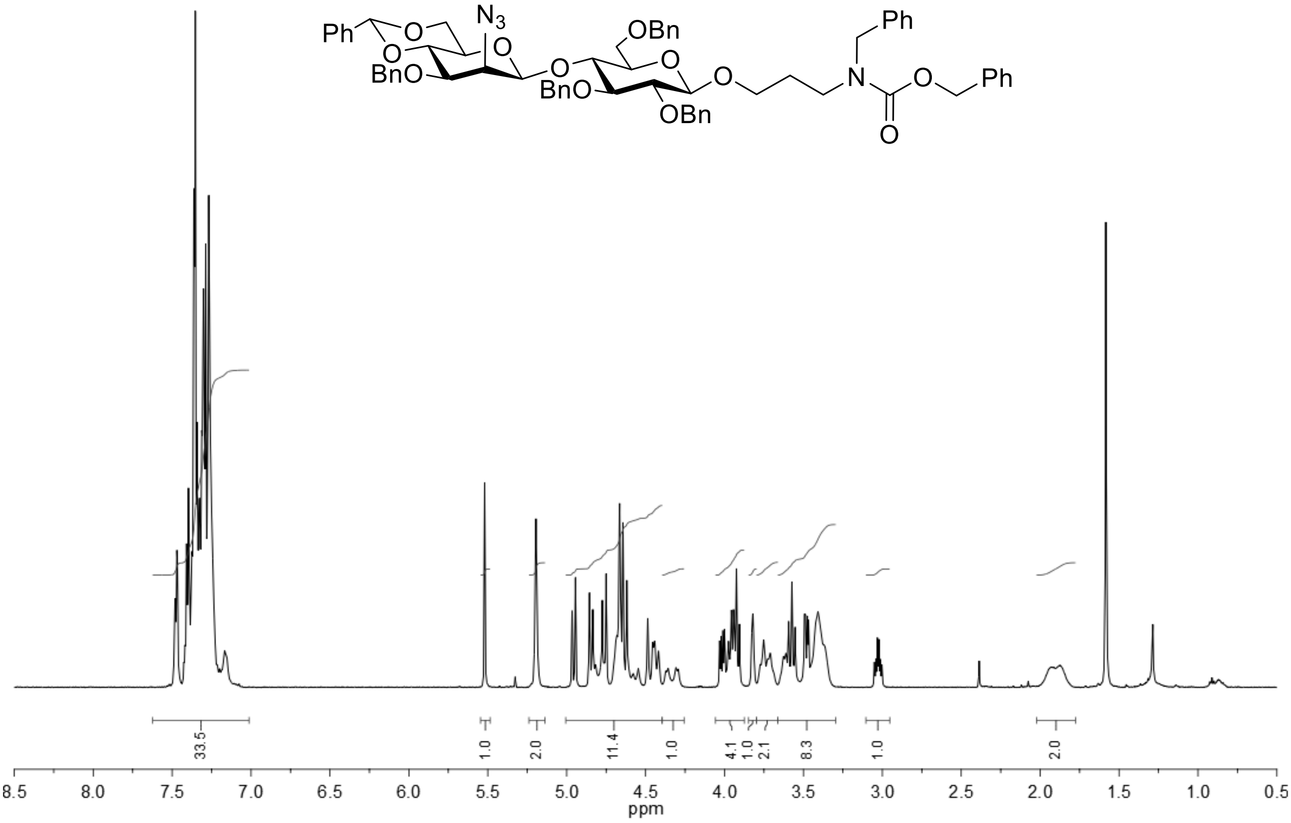

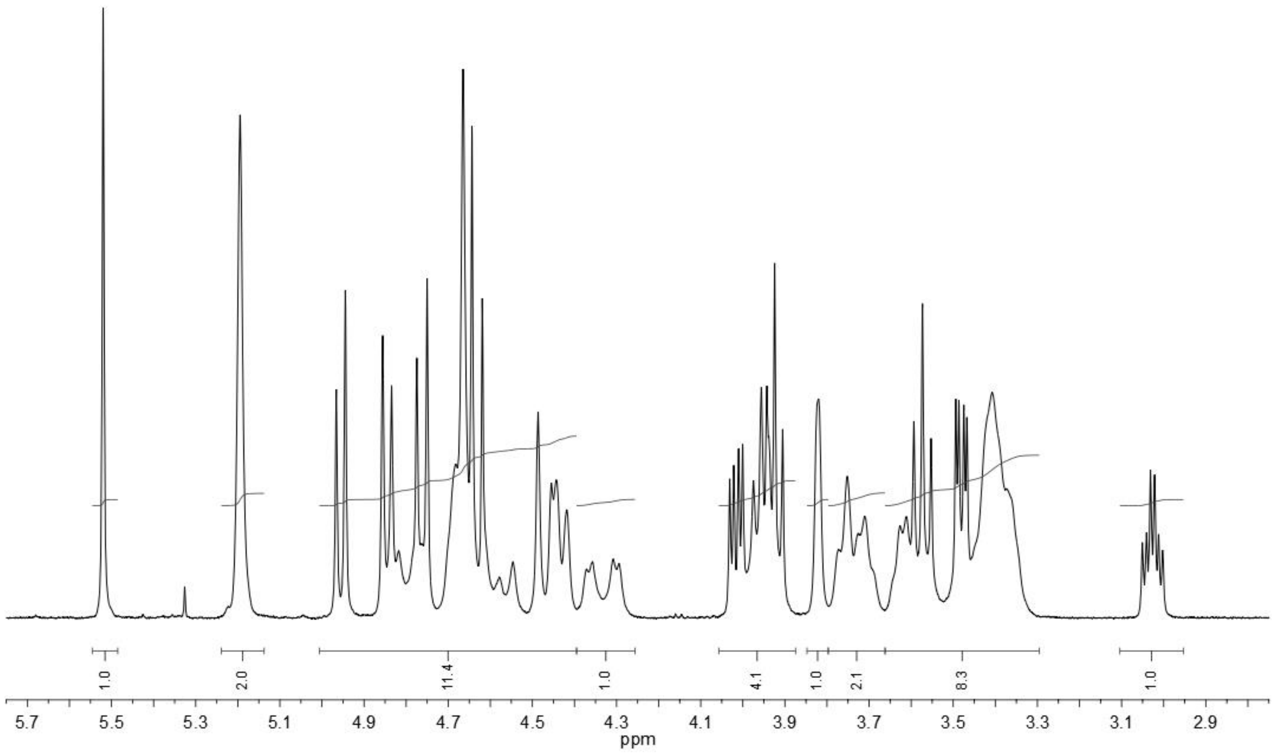

(17): ^{13}C NMR (126 MHz, $CDCl_3$)

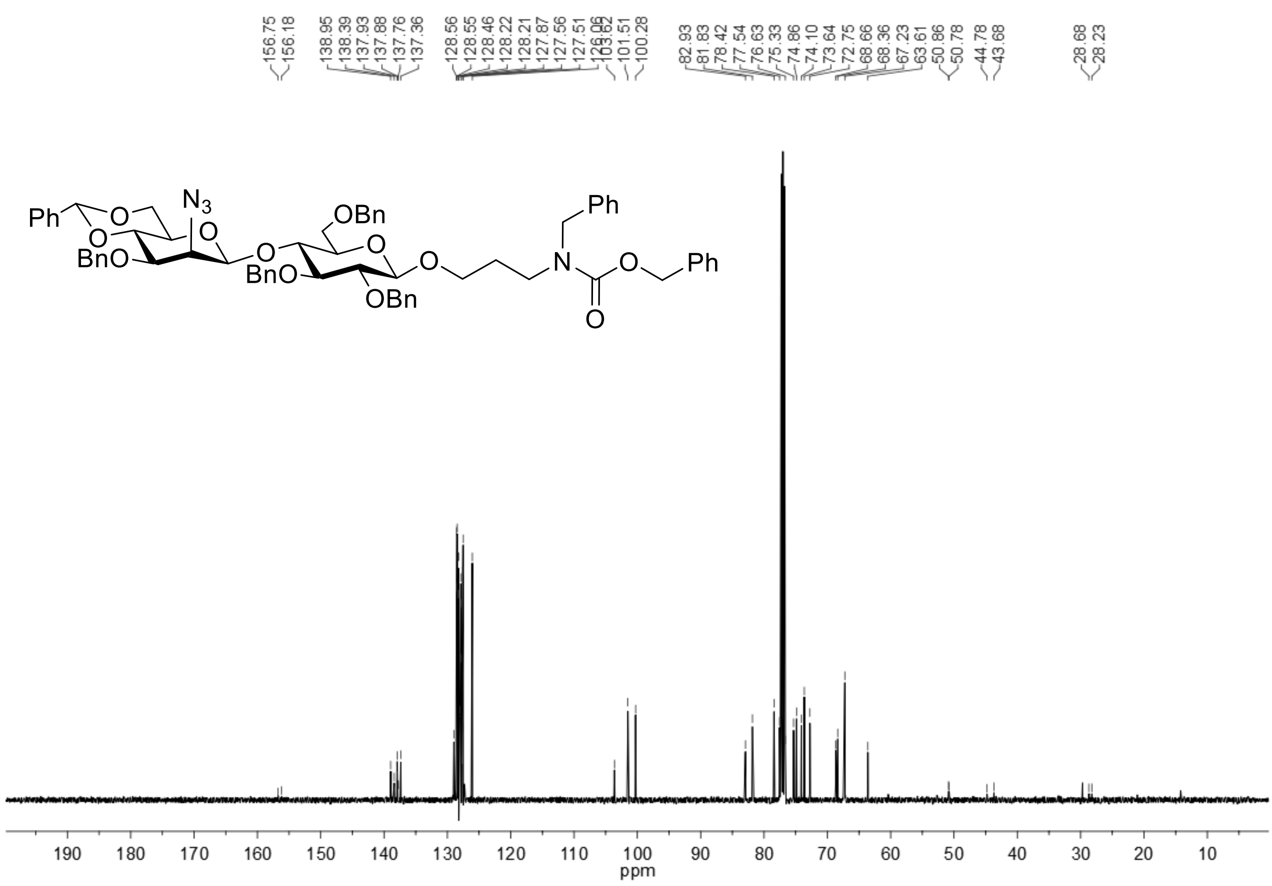

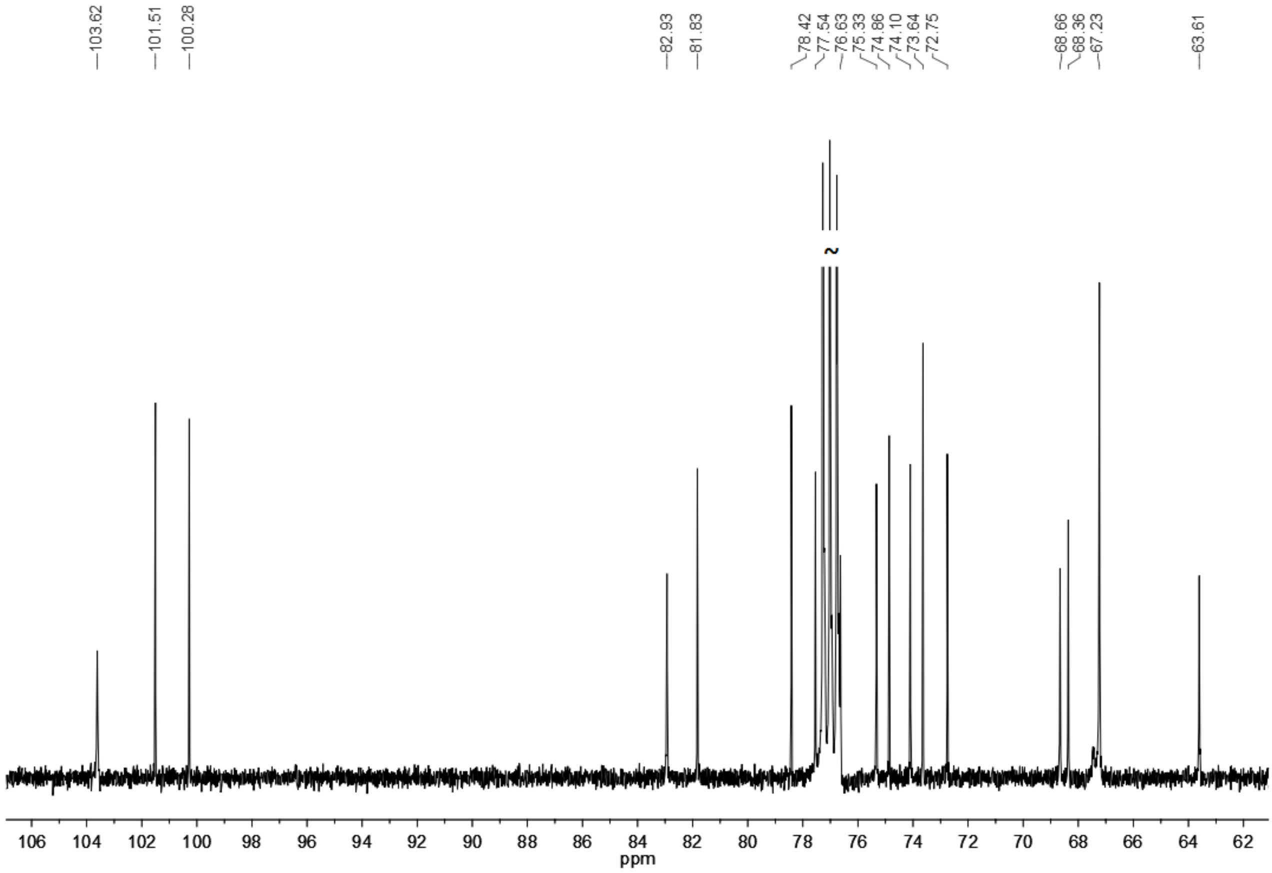

(18): ^{1}H NMR (500 MHz, CDCl$_3$)

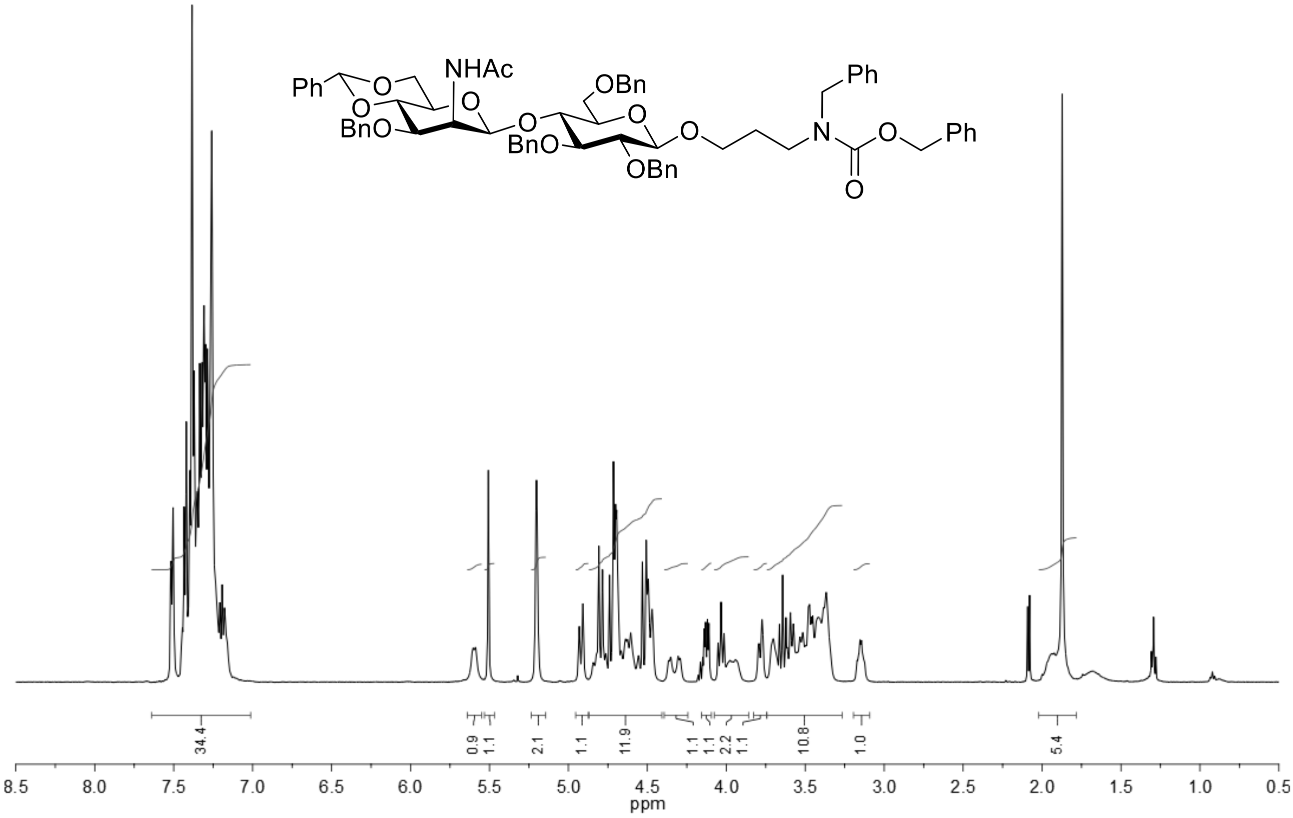

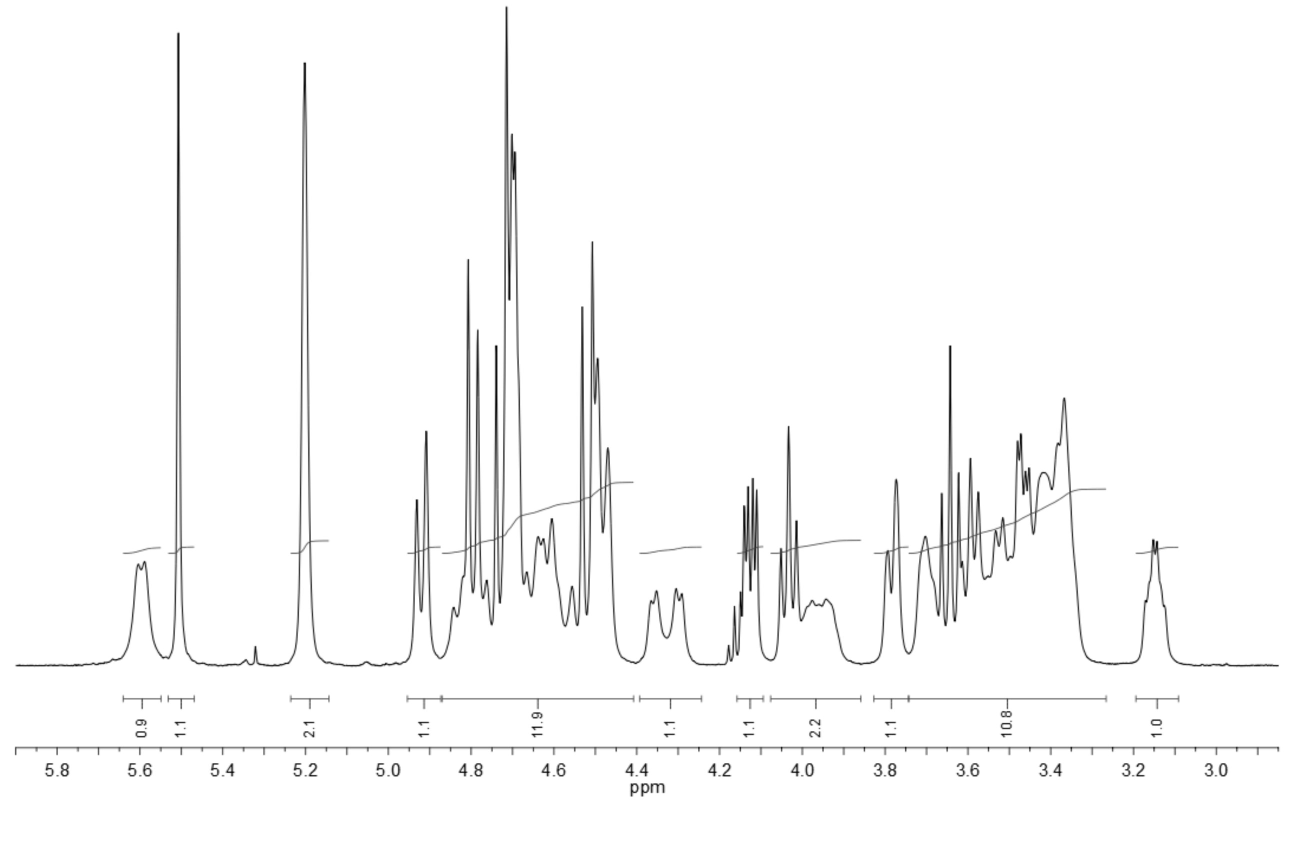

(18): ^{13}C NMR (126 MHz, CDCl$_3$)

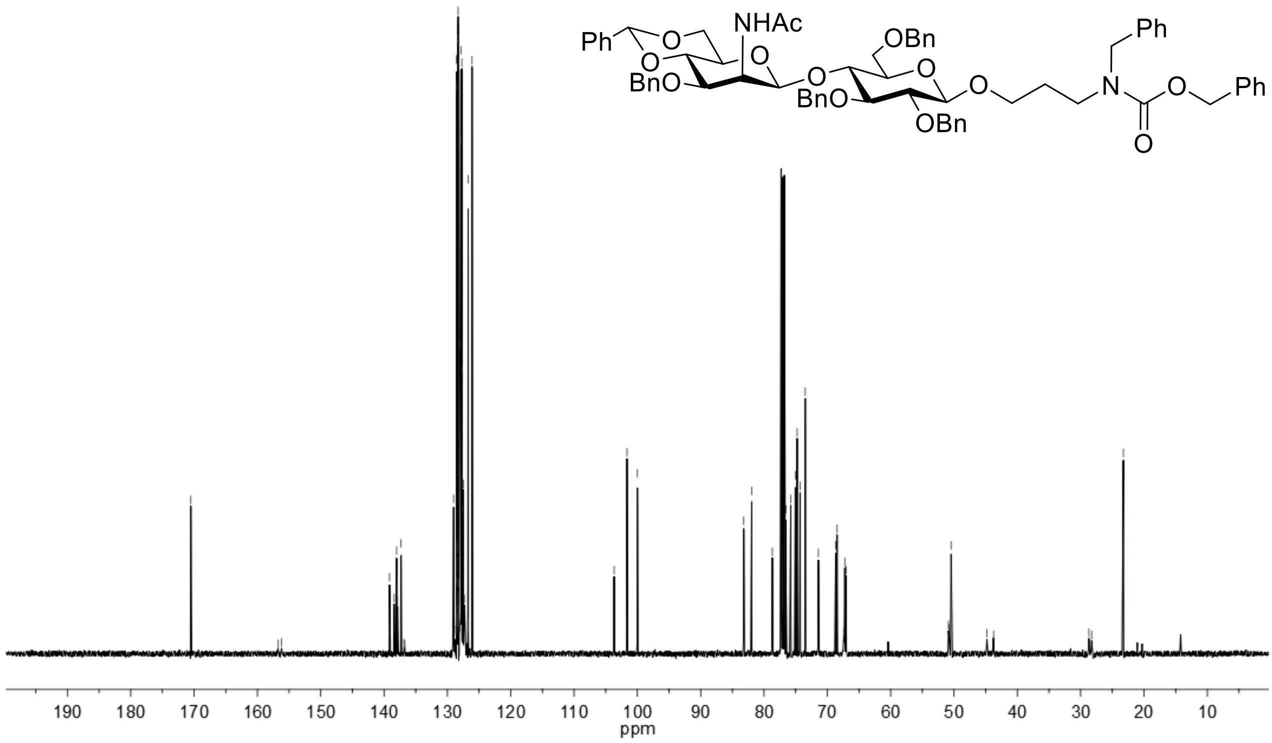

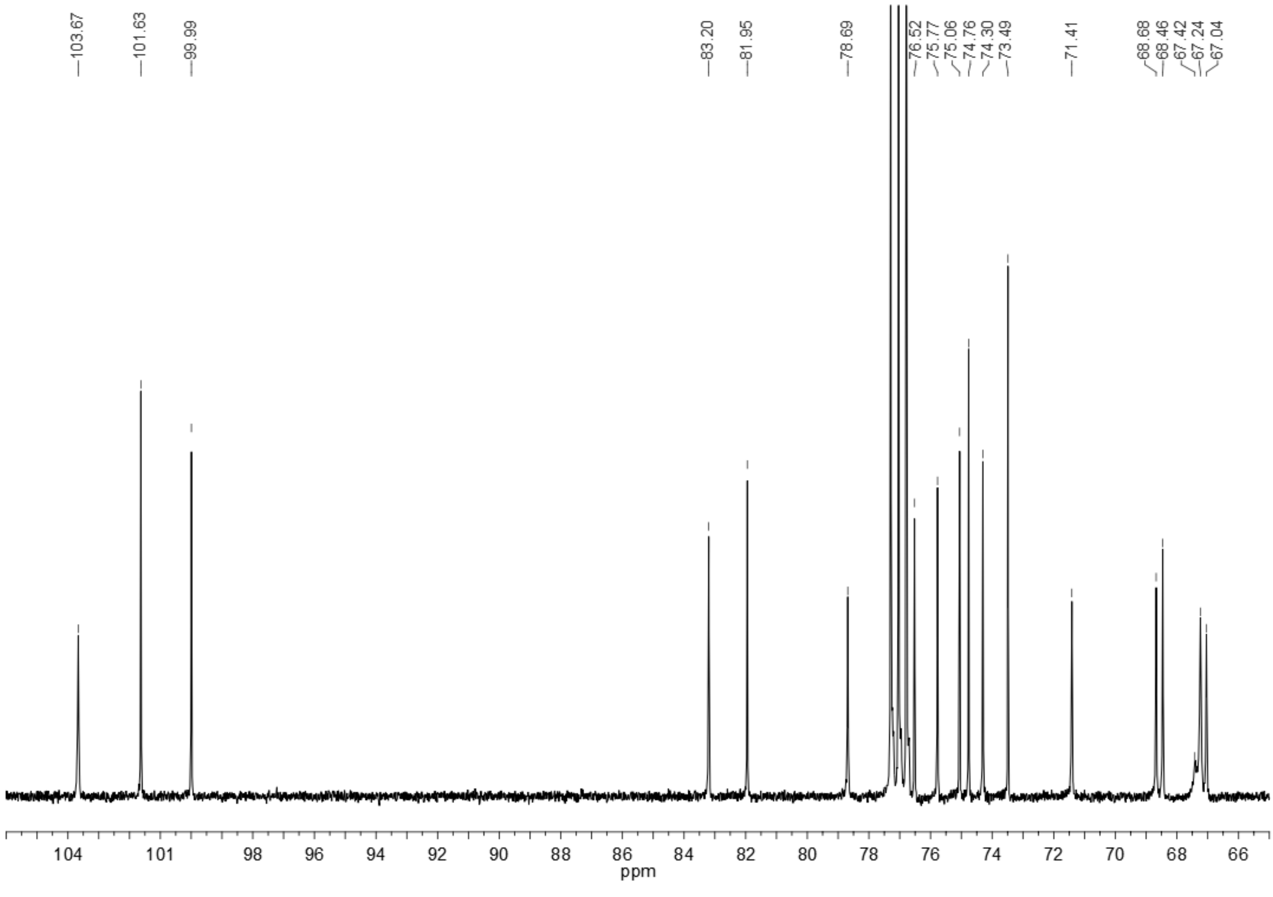

(1): ^{1}H NMR (500 MHz, D_2O)

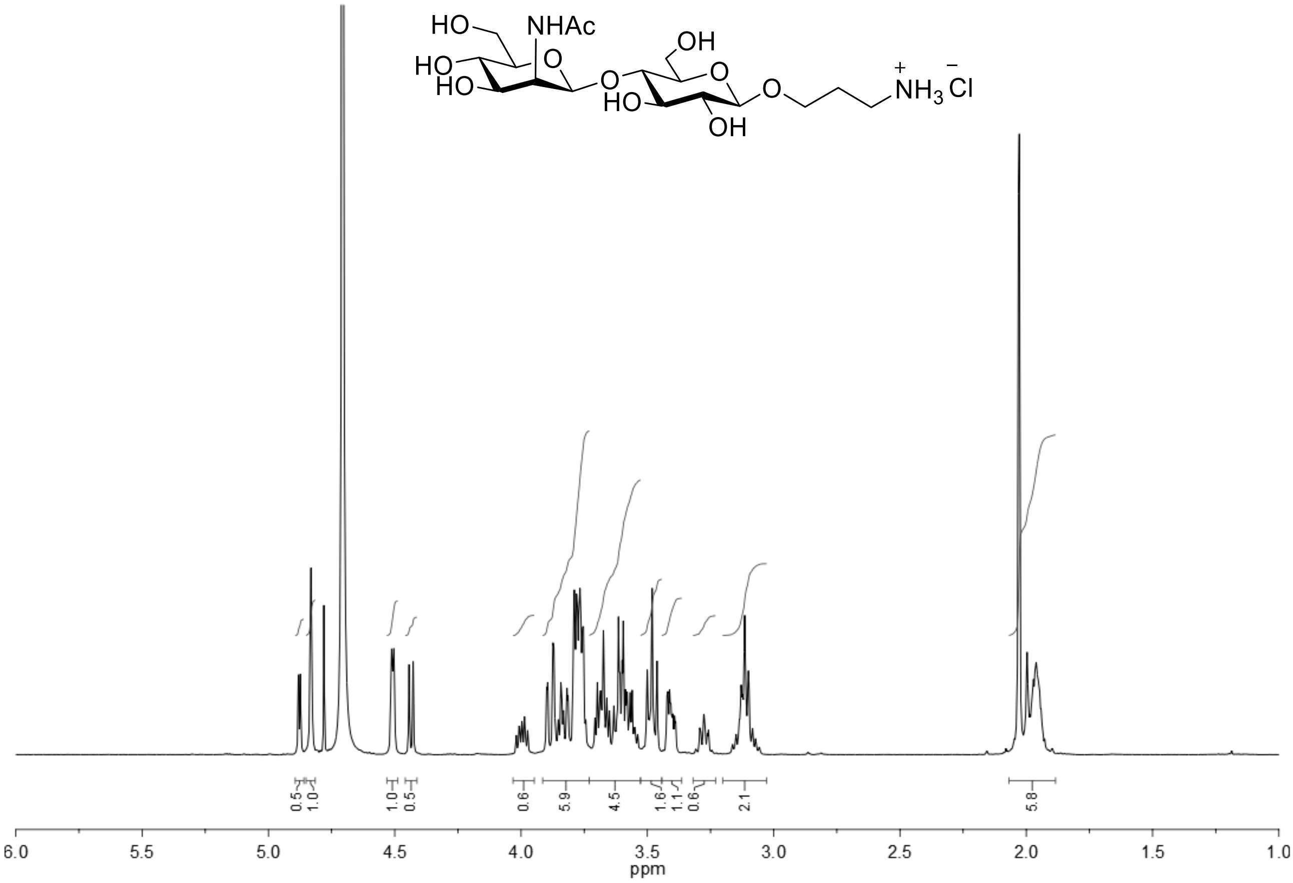

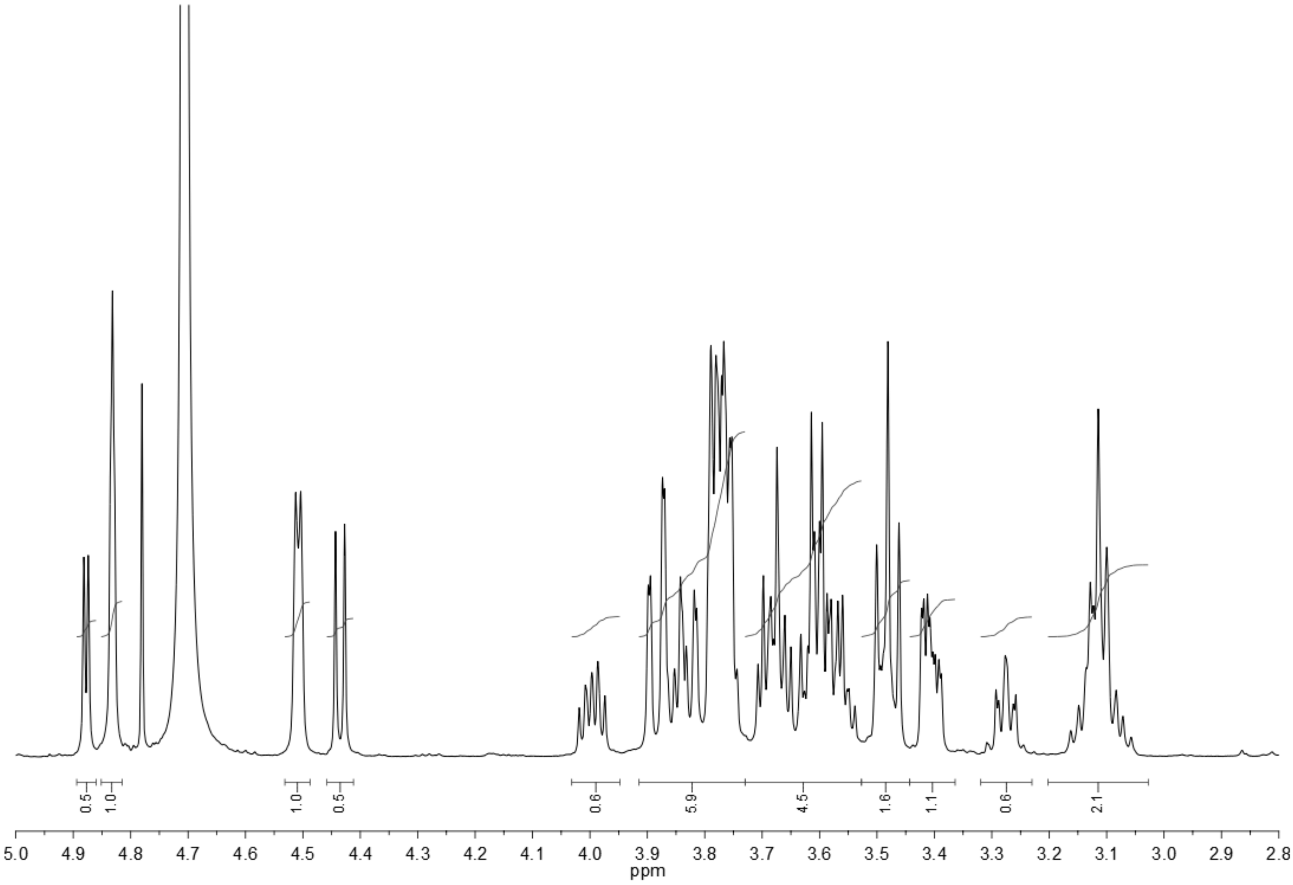

(1): ^{13}C NMR (126 MHz, D_2O)

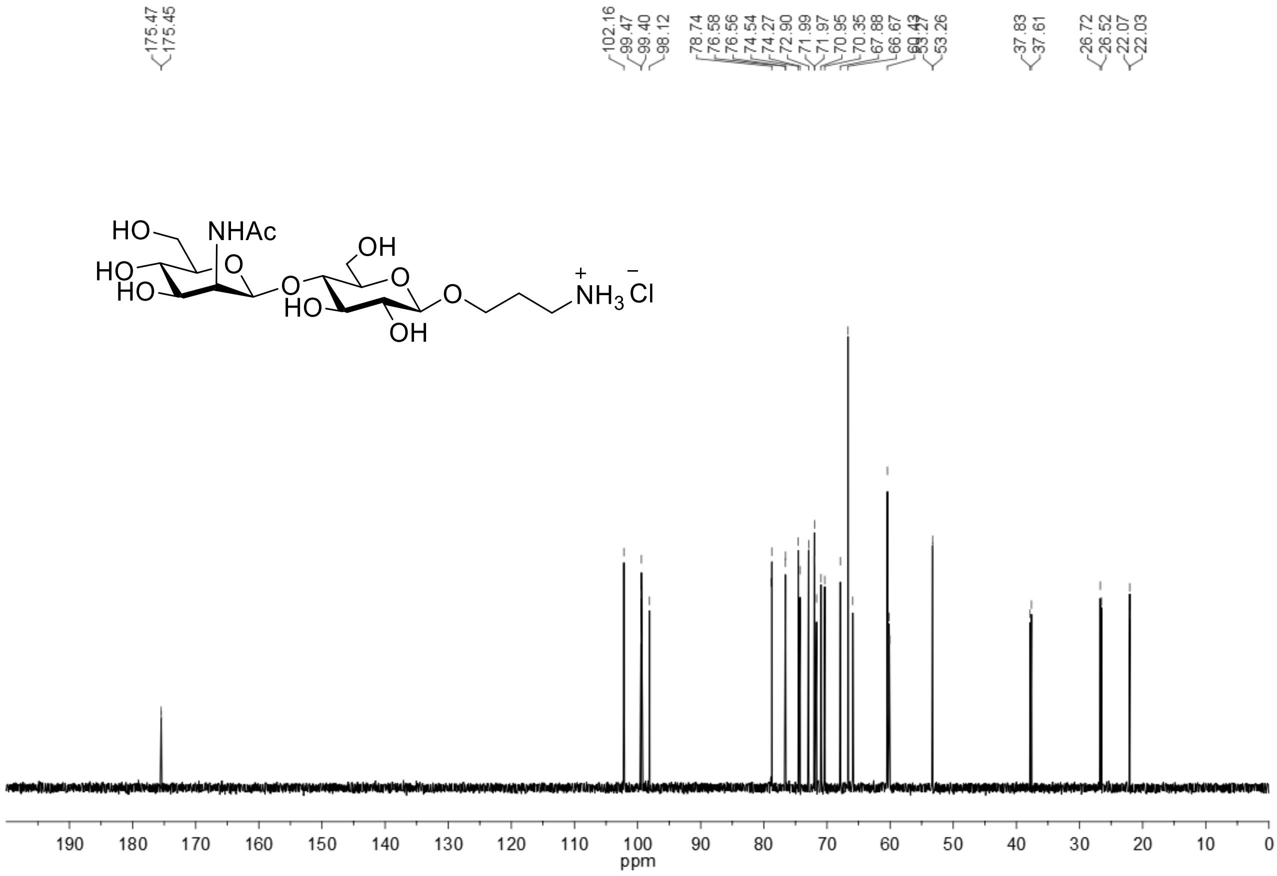

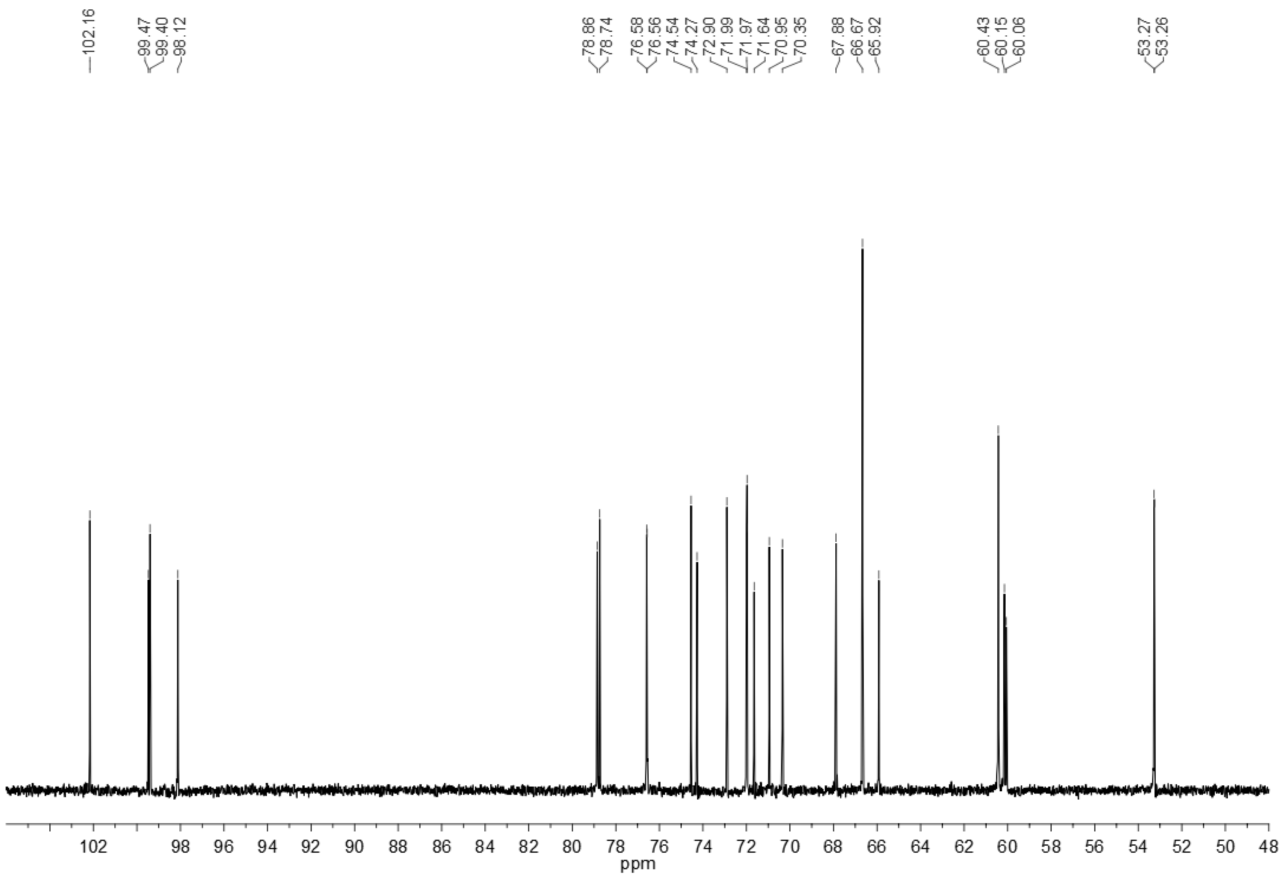

(19): ^{1}H NMR (500 MHz, $CDCl_3$)

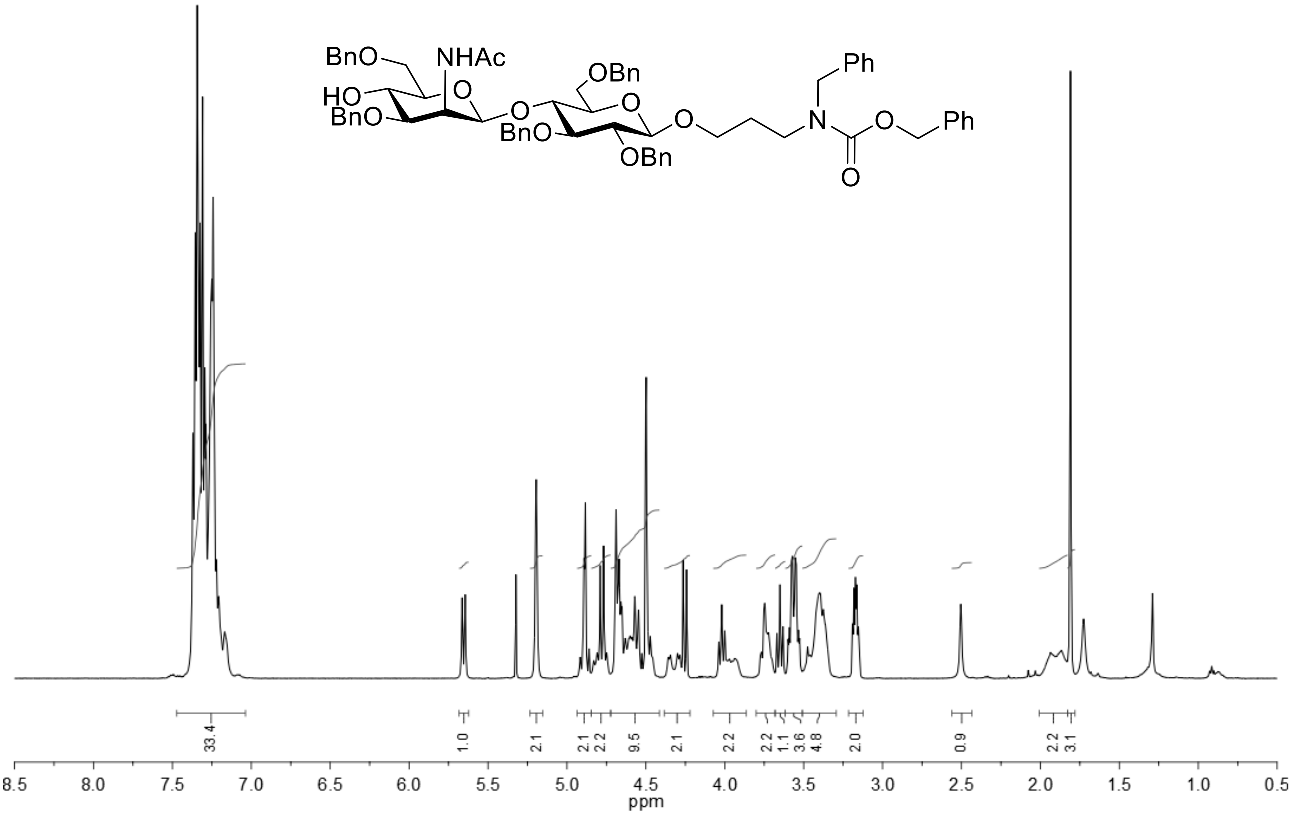

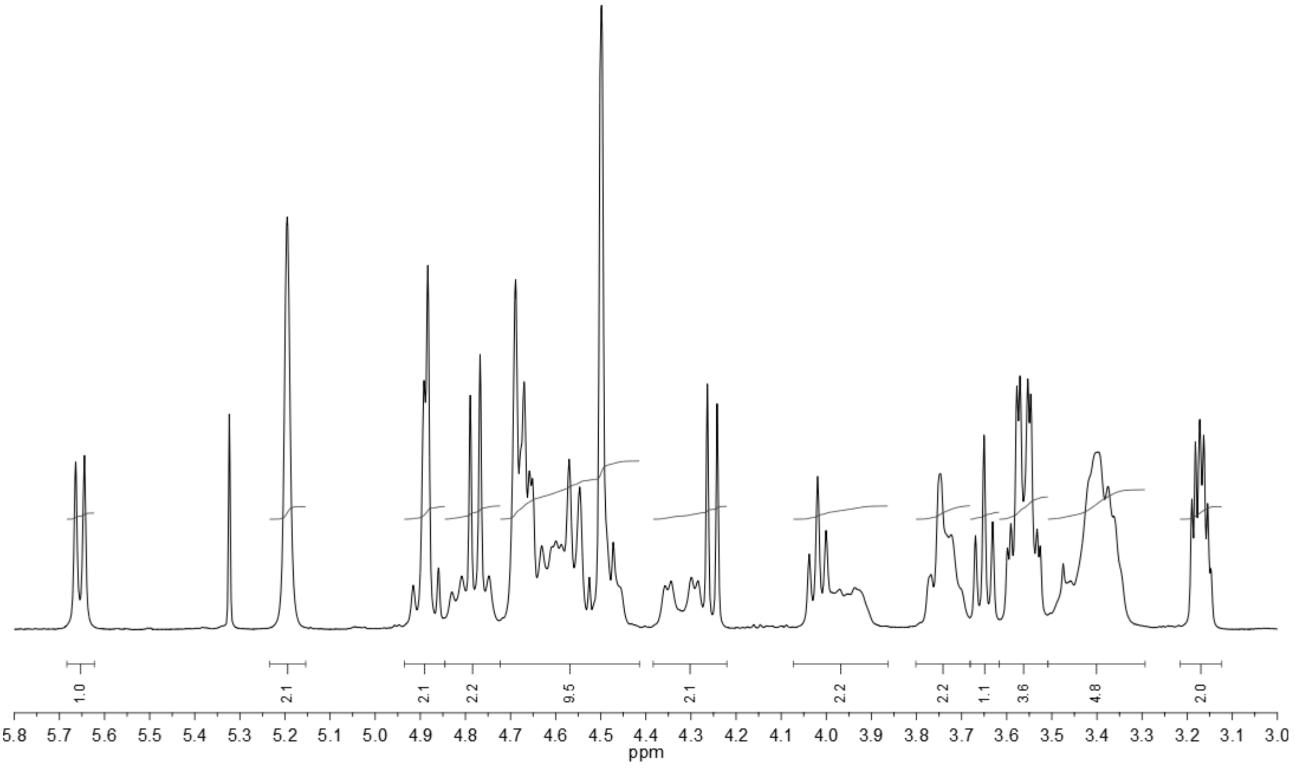

(19): ^{13}C NMR (126 MHz, $CDCl_3$)

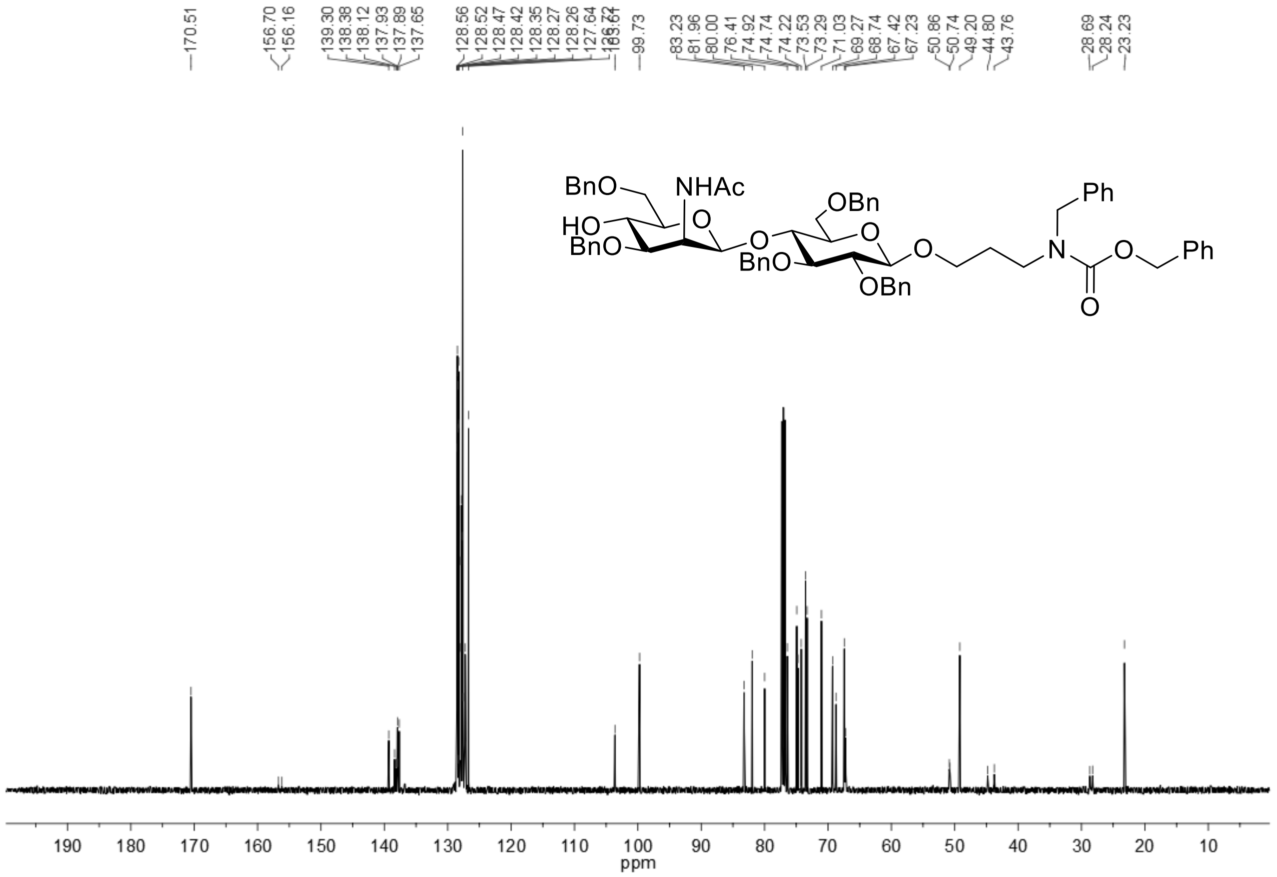

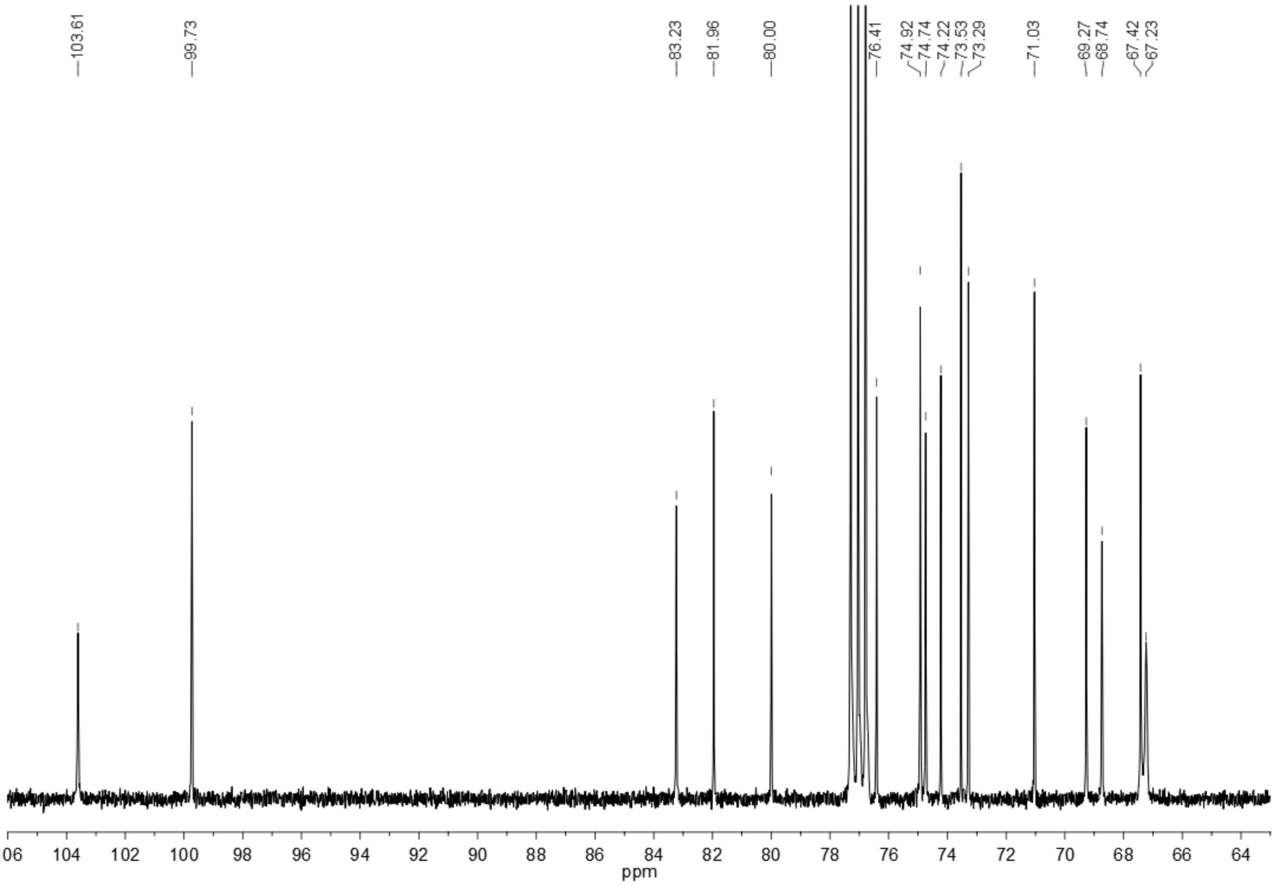

(20): ^{1}H NMR (500 MHz, $CDCl_3$)

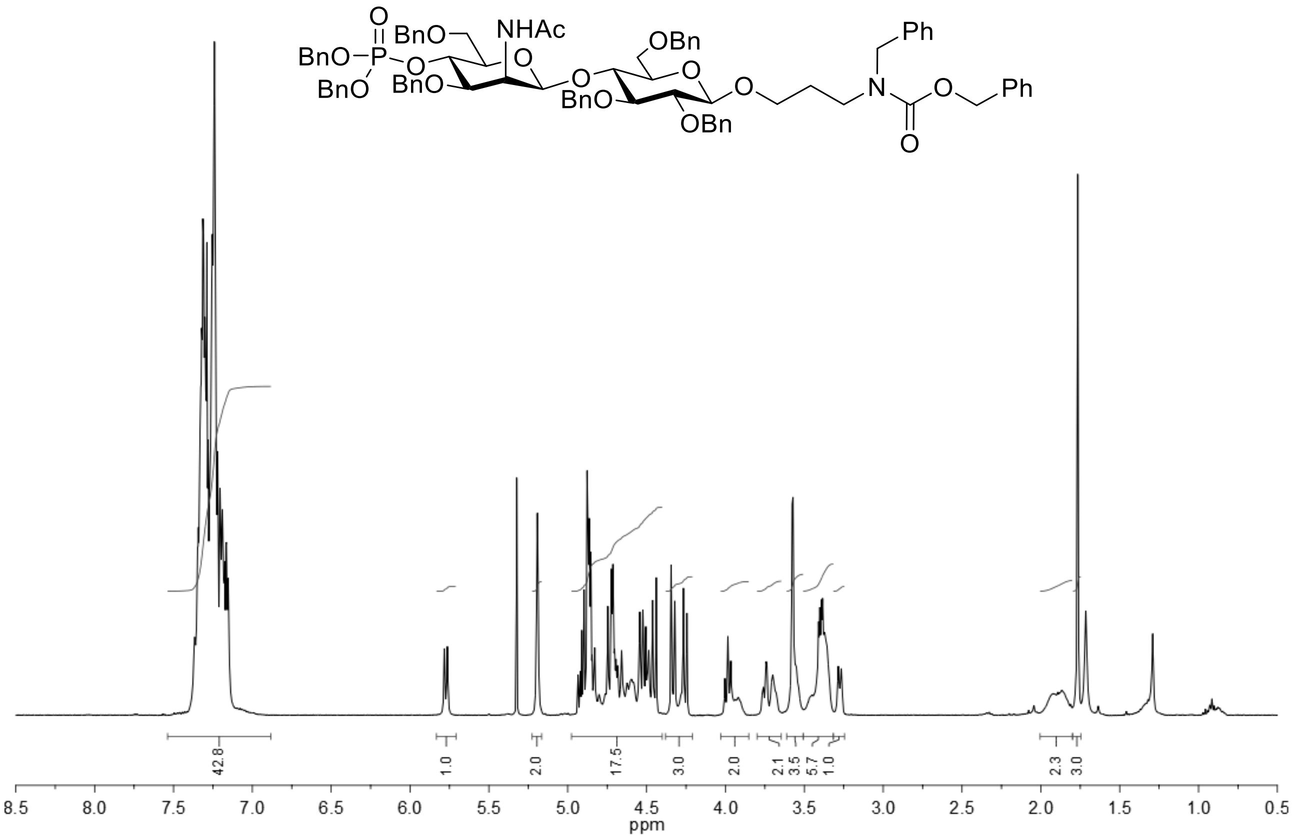

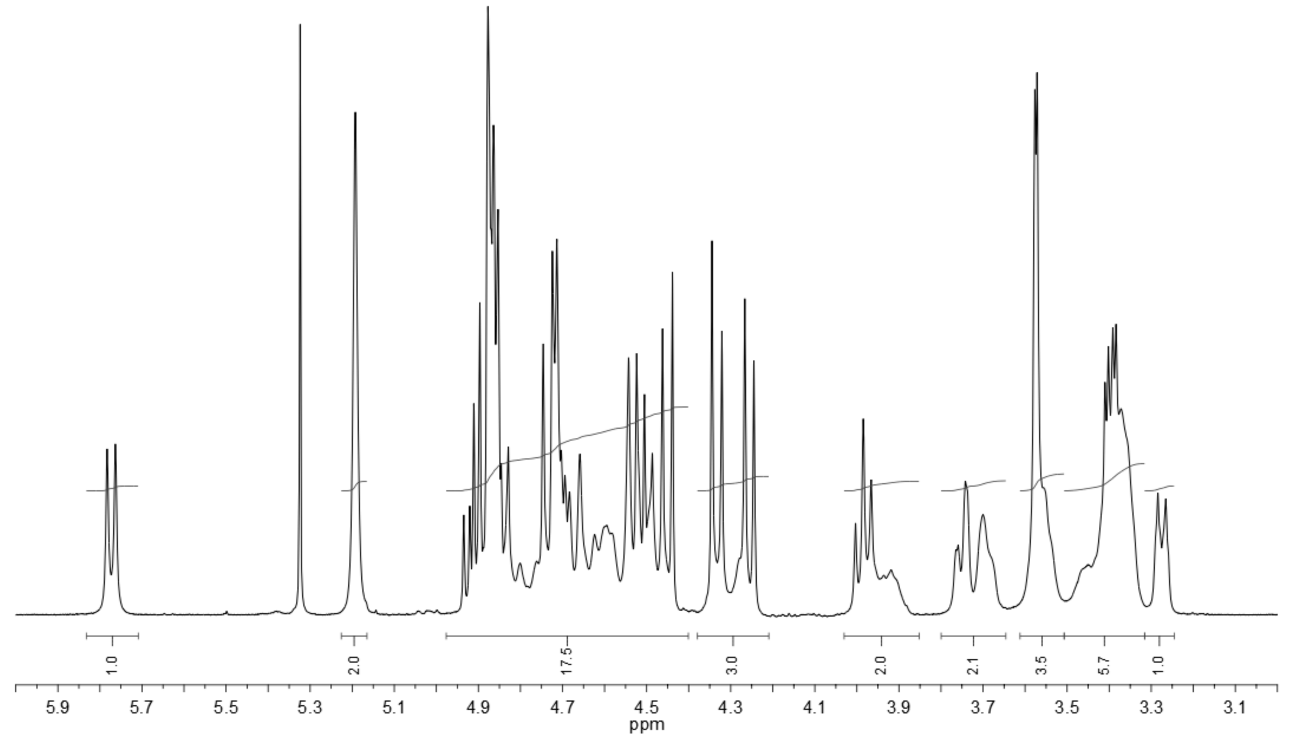

(20): ^{31}P NMR (202 MHz, $CDCl_3$)

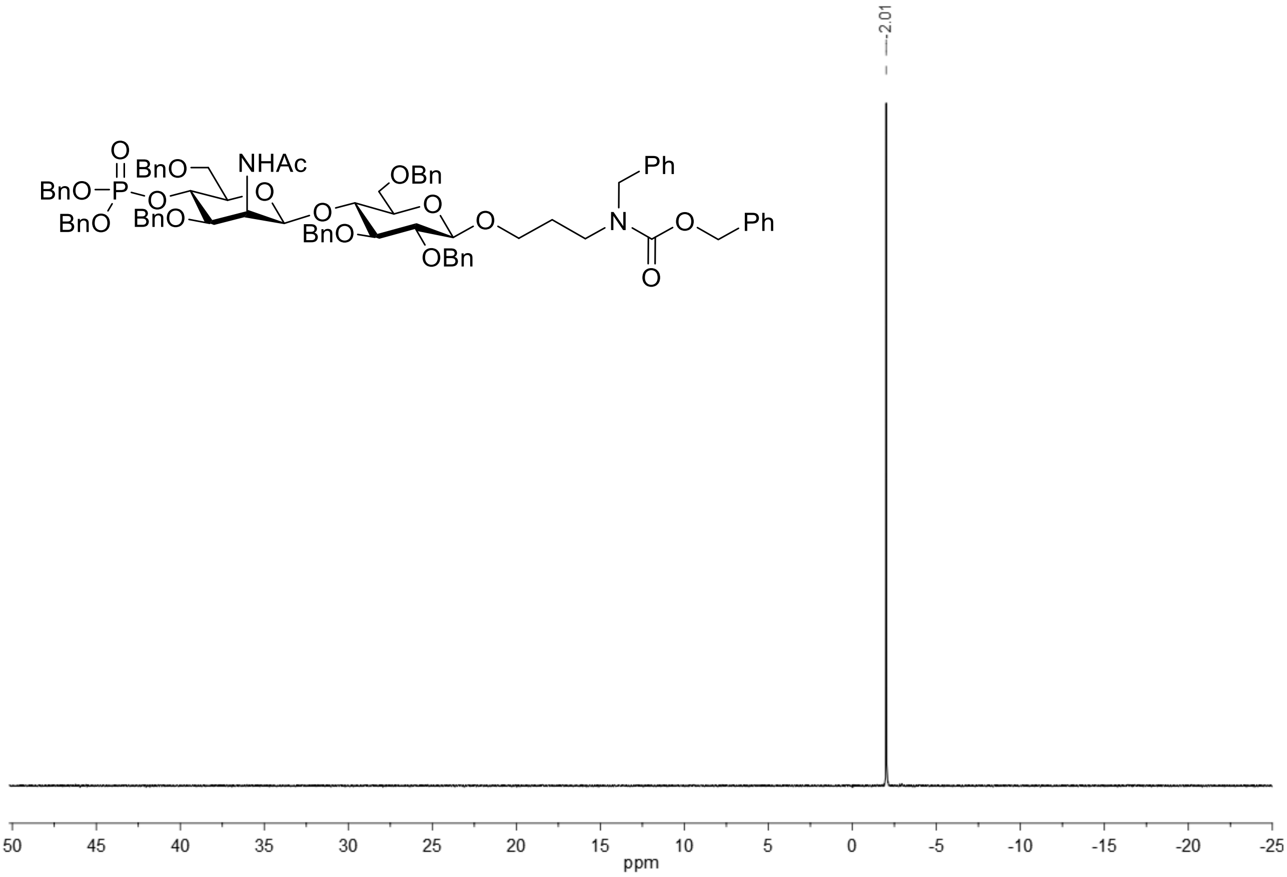

(20): ^{13}C NMR (126 MHz, CDCl$_3$)

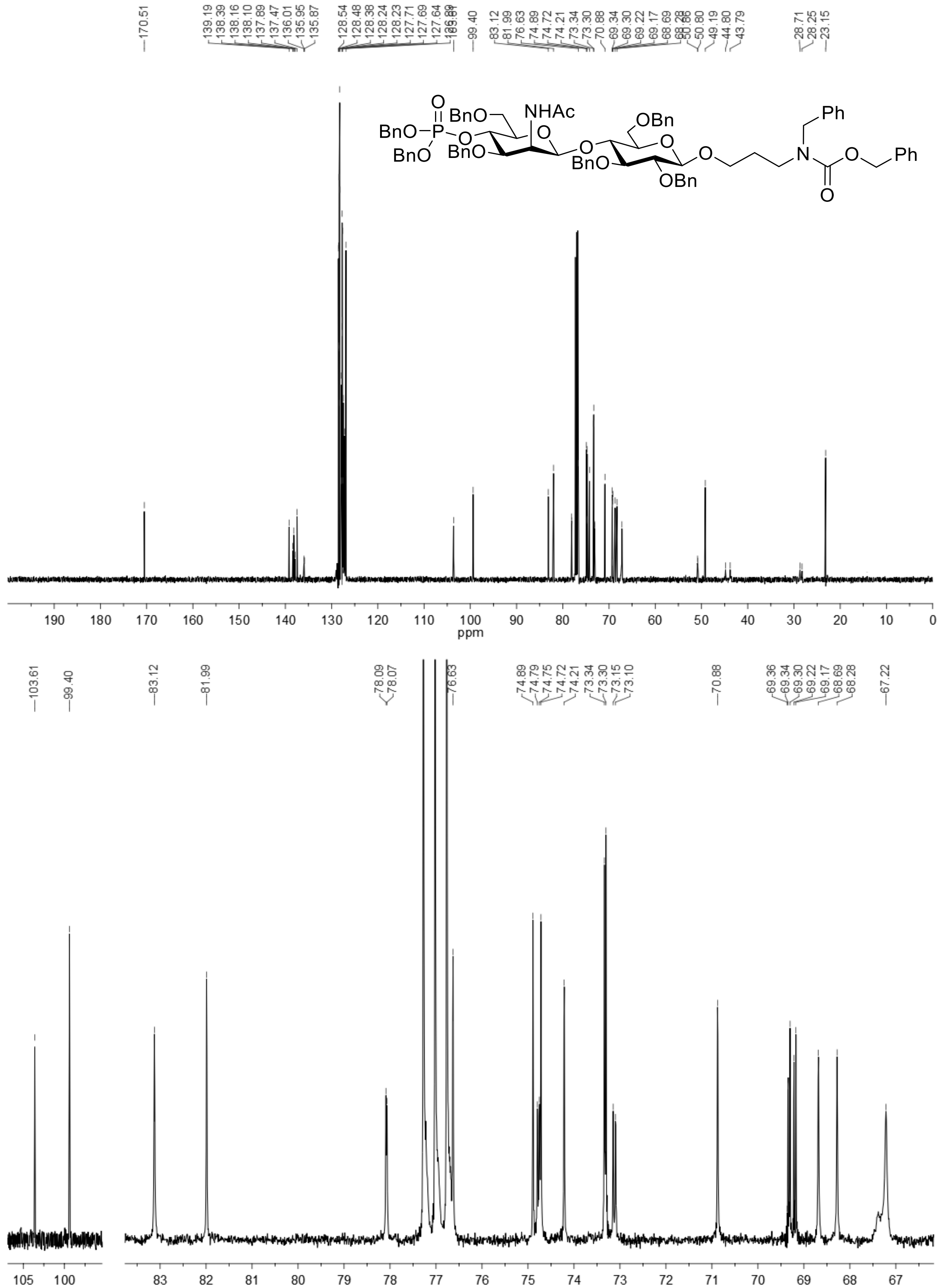

(2): ^{1}H NMR (500 MHz, D_2O)

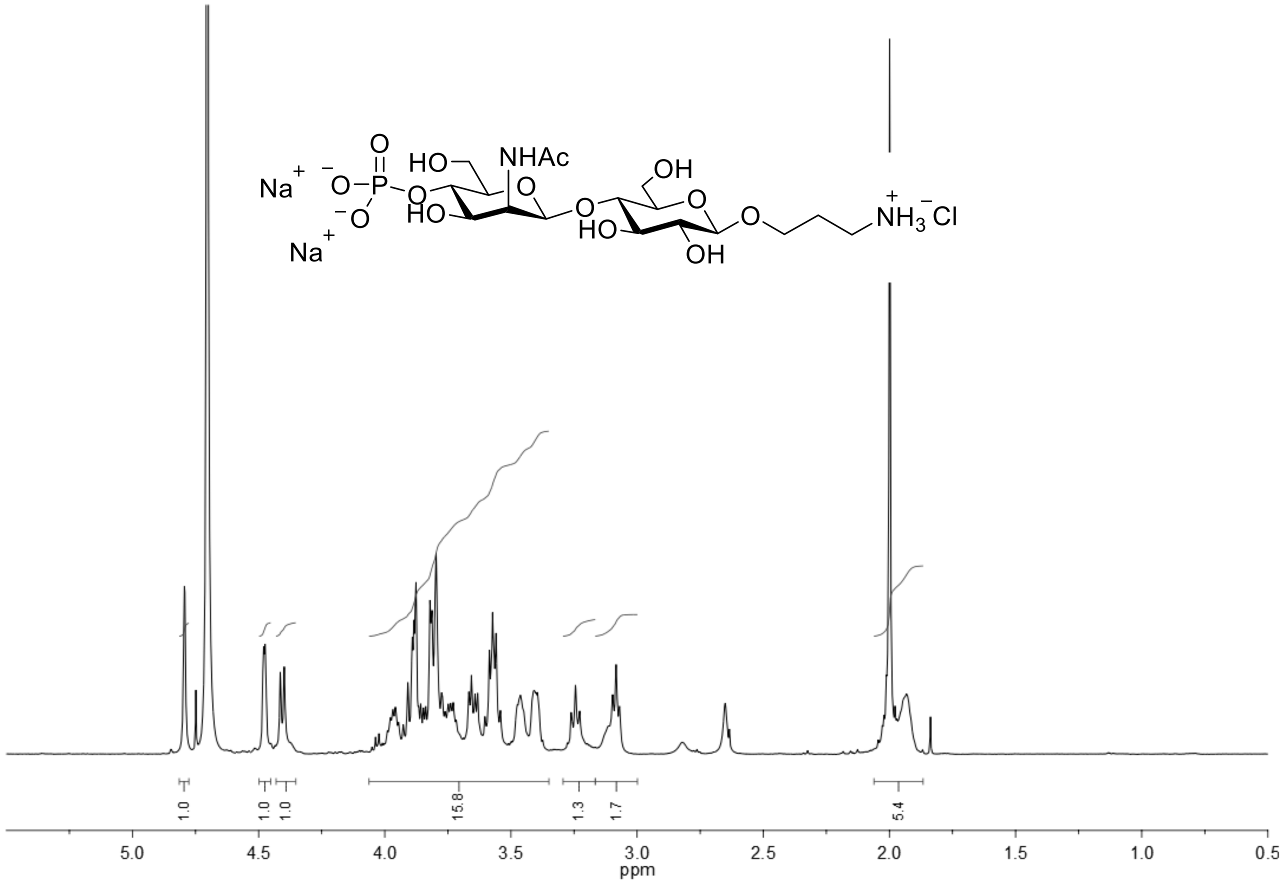

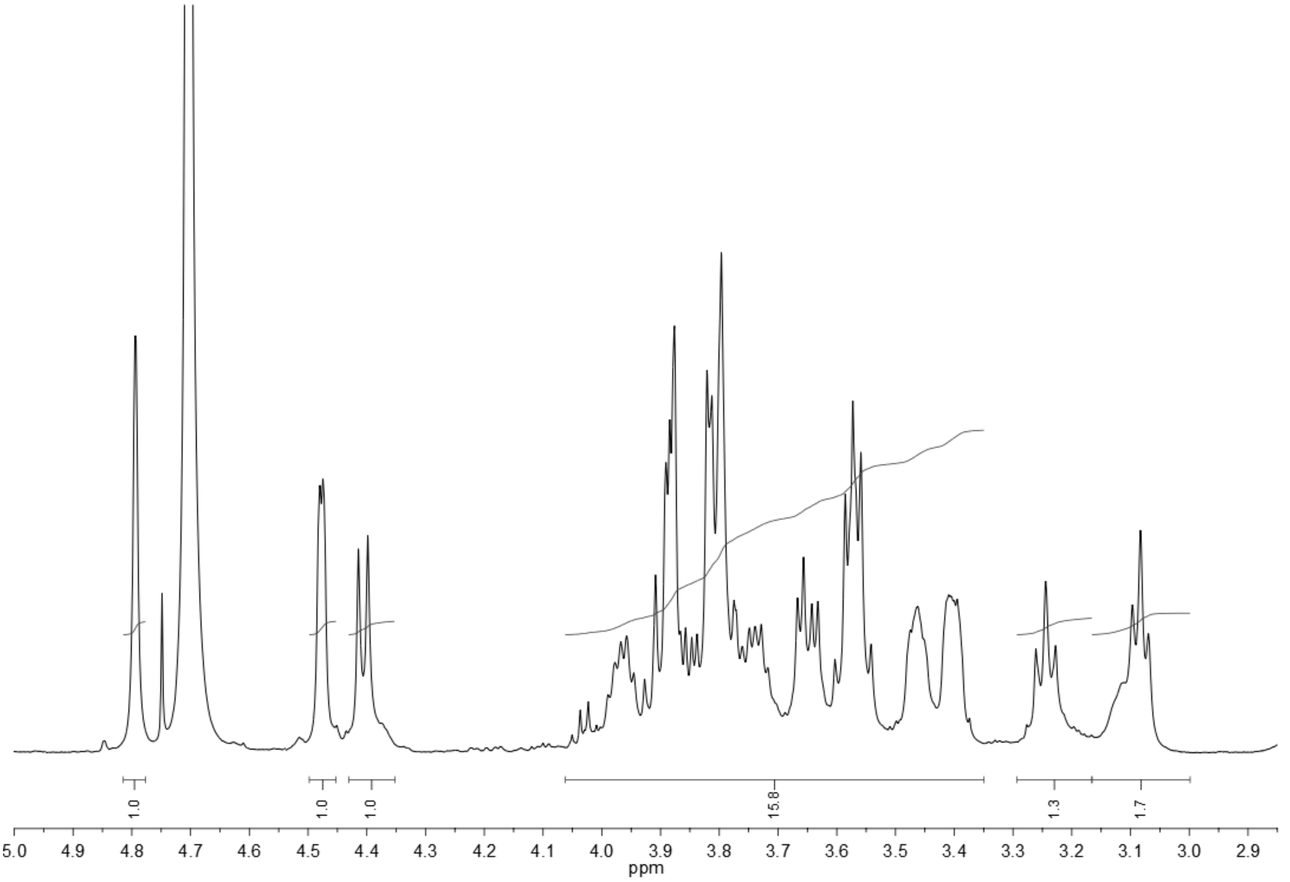

(2): ^{31}P NMR (202 MHz, D_2O)

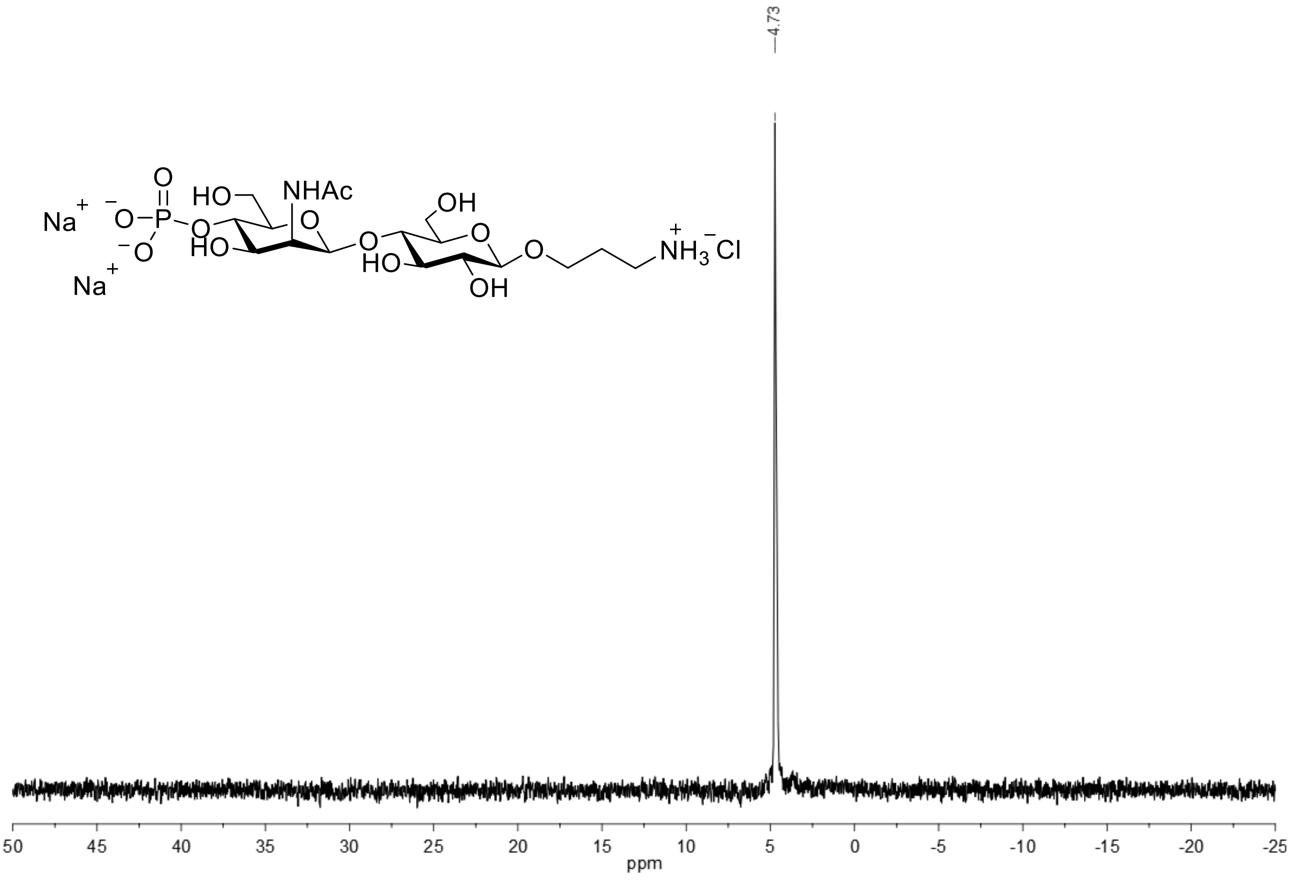

(2): ^{13}C NMR (126 MHz, D_2O)

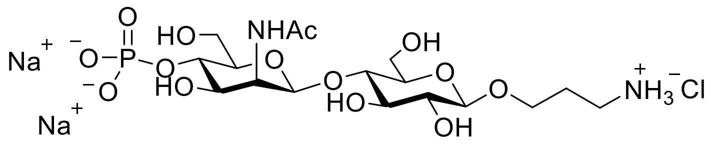

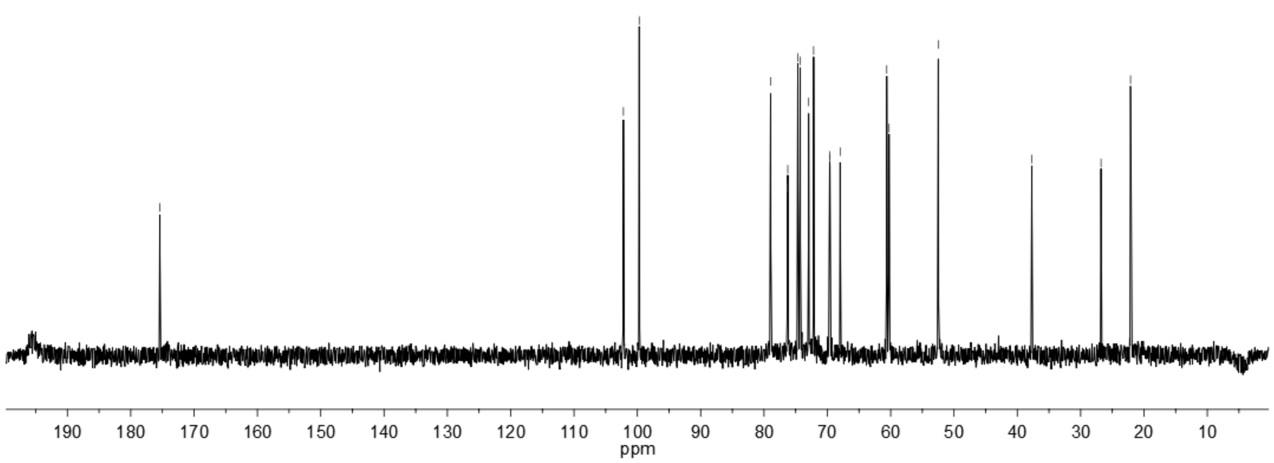

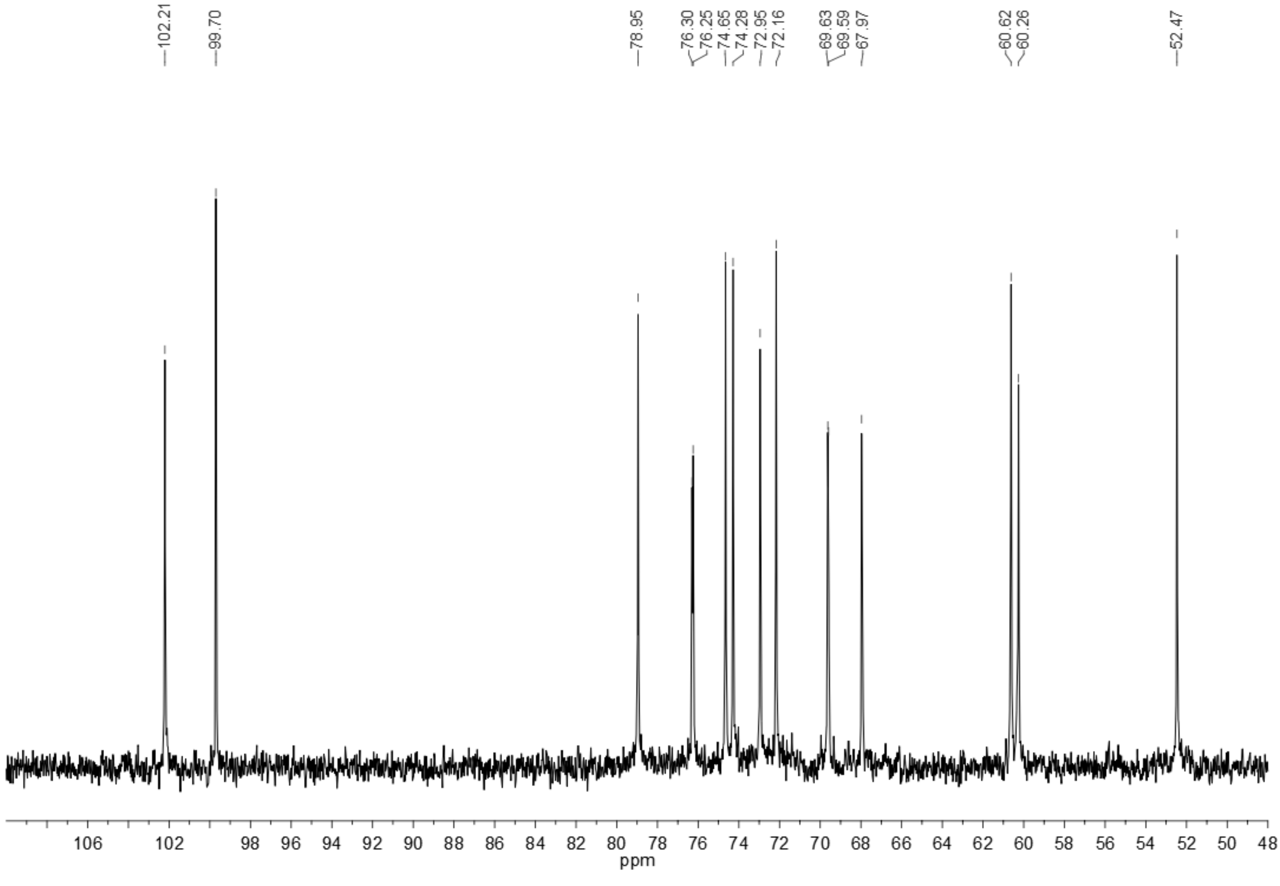

(21): ^{1}H NMR (500 MHz, CDCl$_3$)

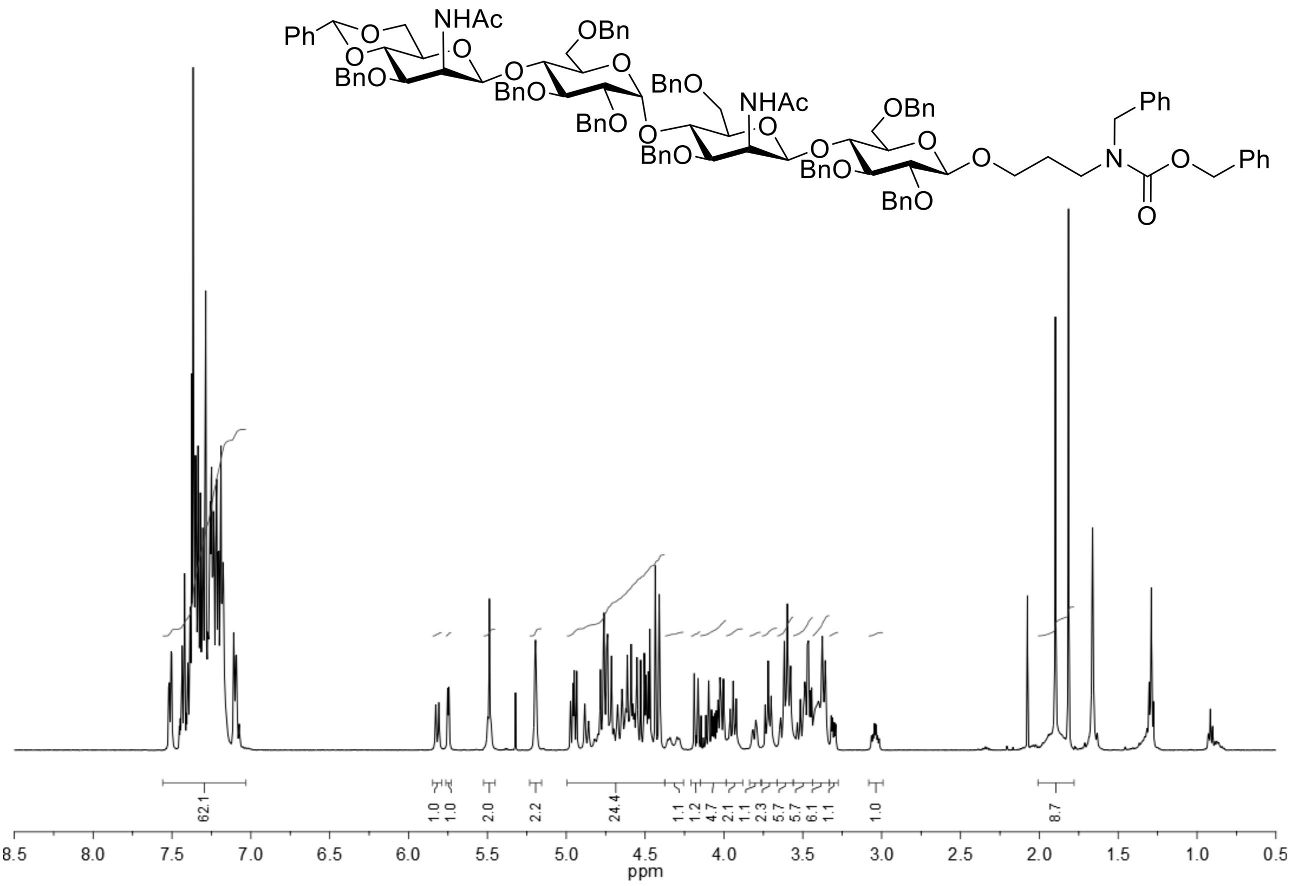

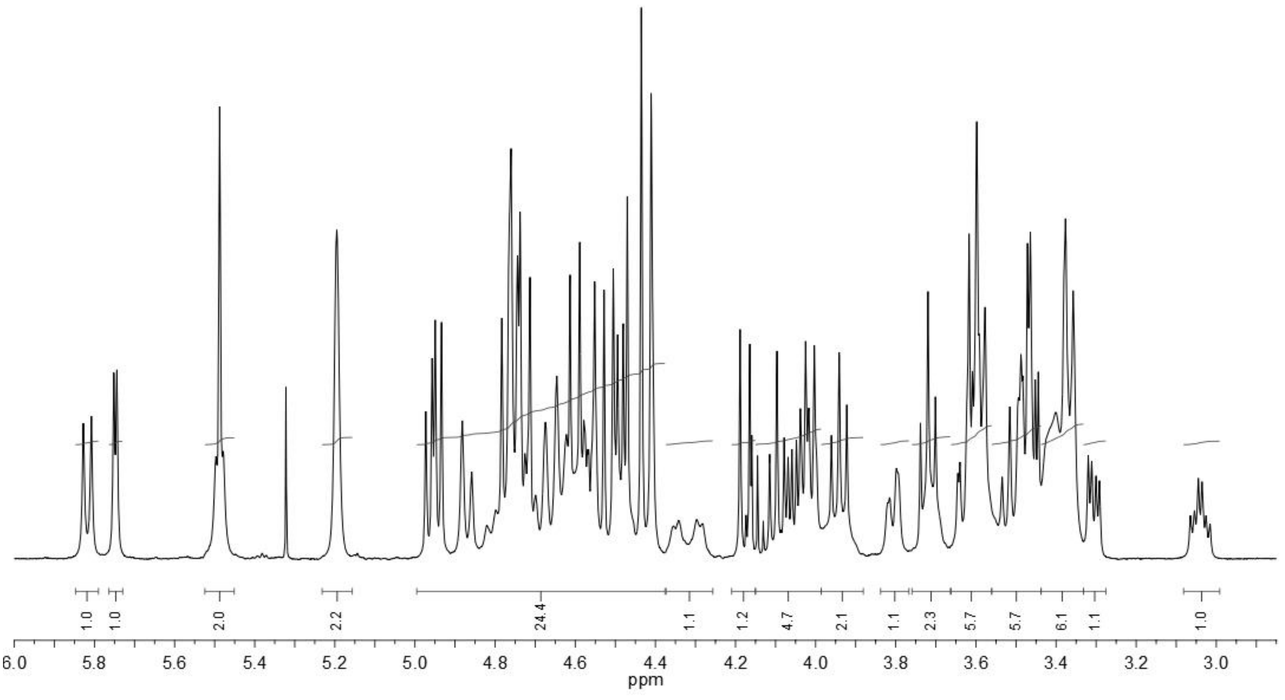

(21): ^{13}C NMR (126 MHz, $CDCl_3$)

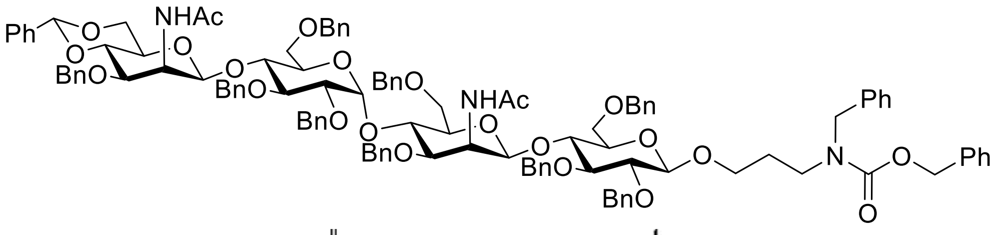

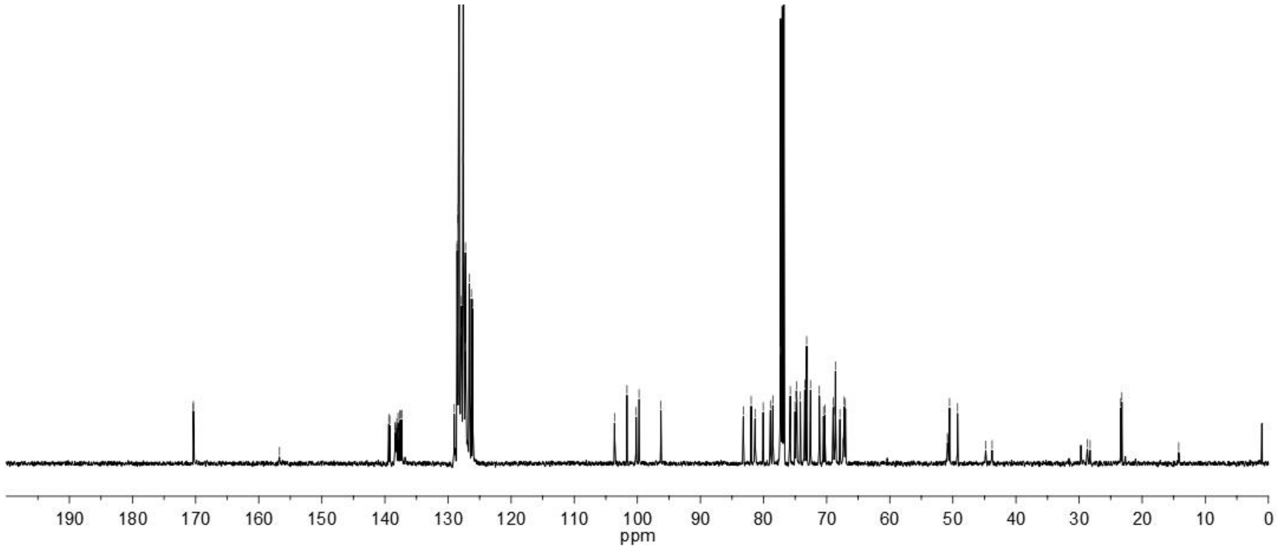

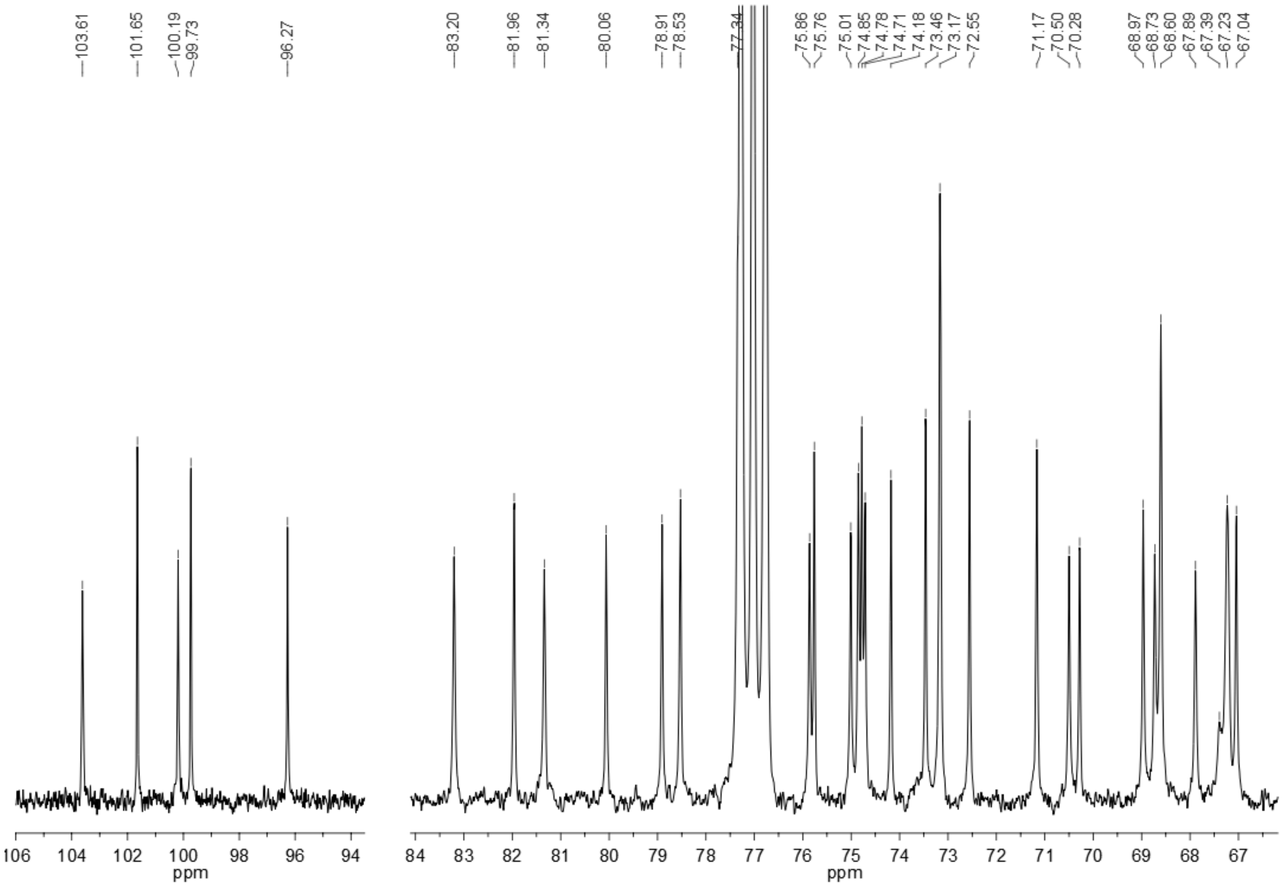

(6) ¹H NMR (500 MHz, D₂O)

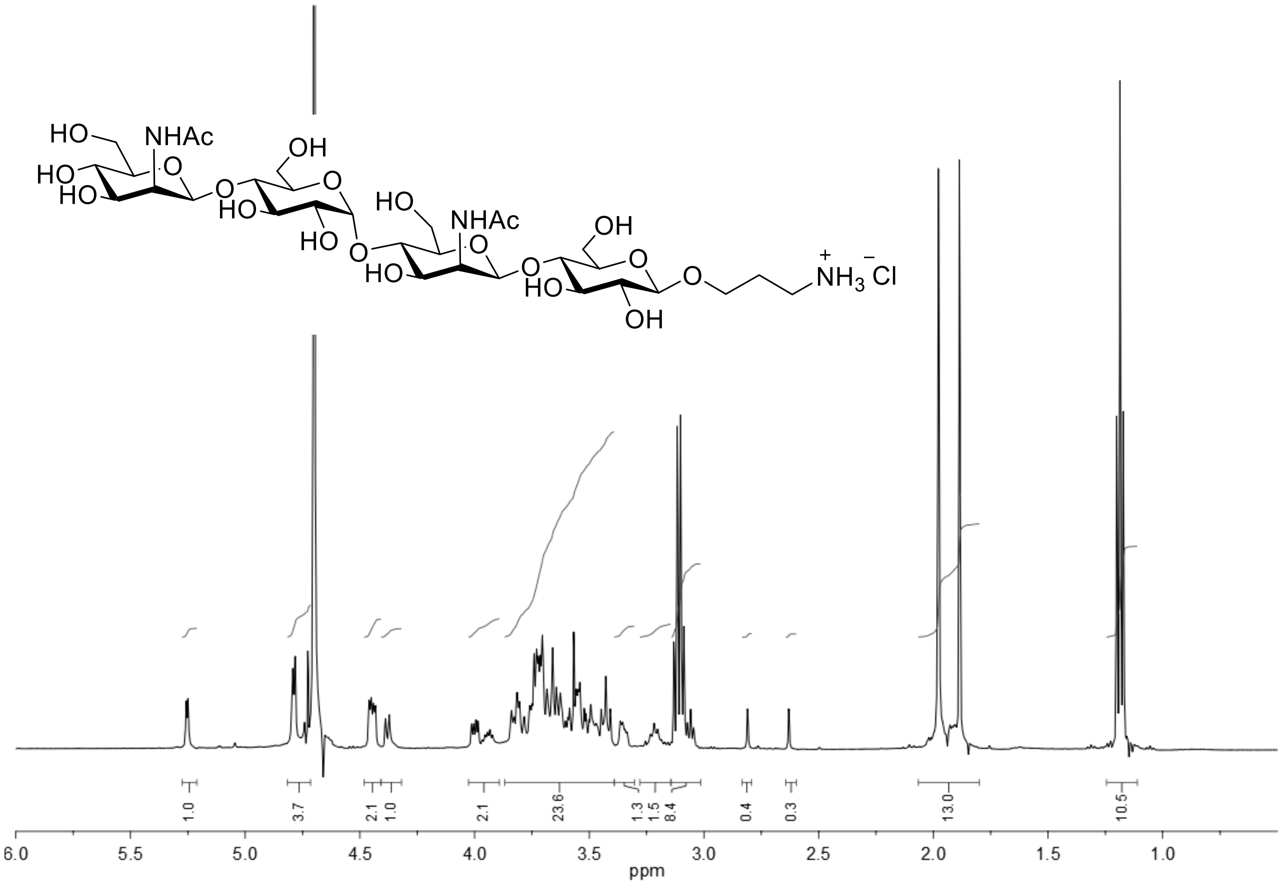

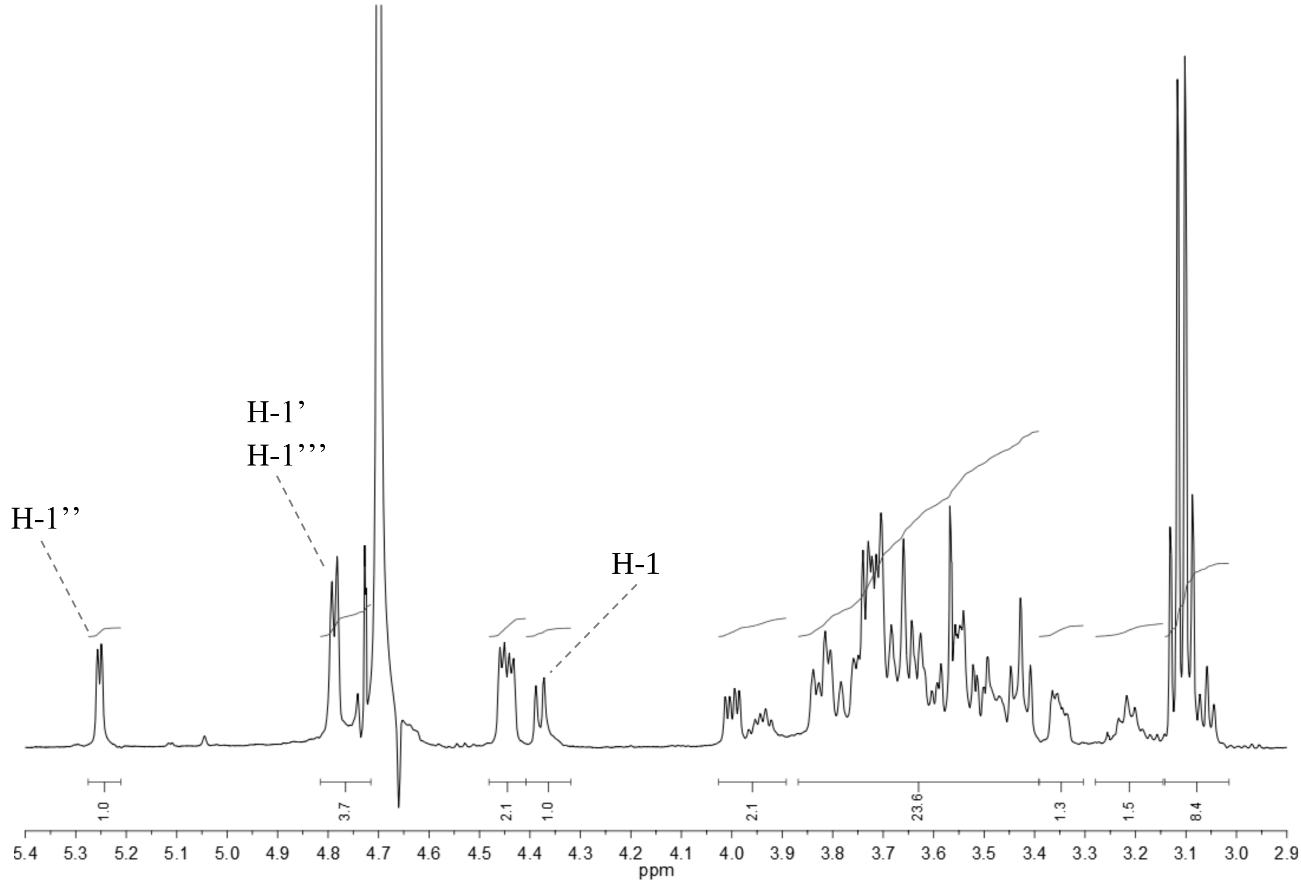

(6) ^{13}C NMR (126 MHz, D_2O)

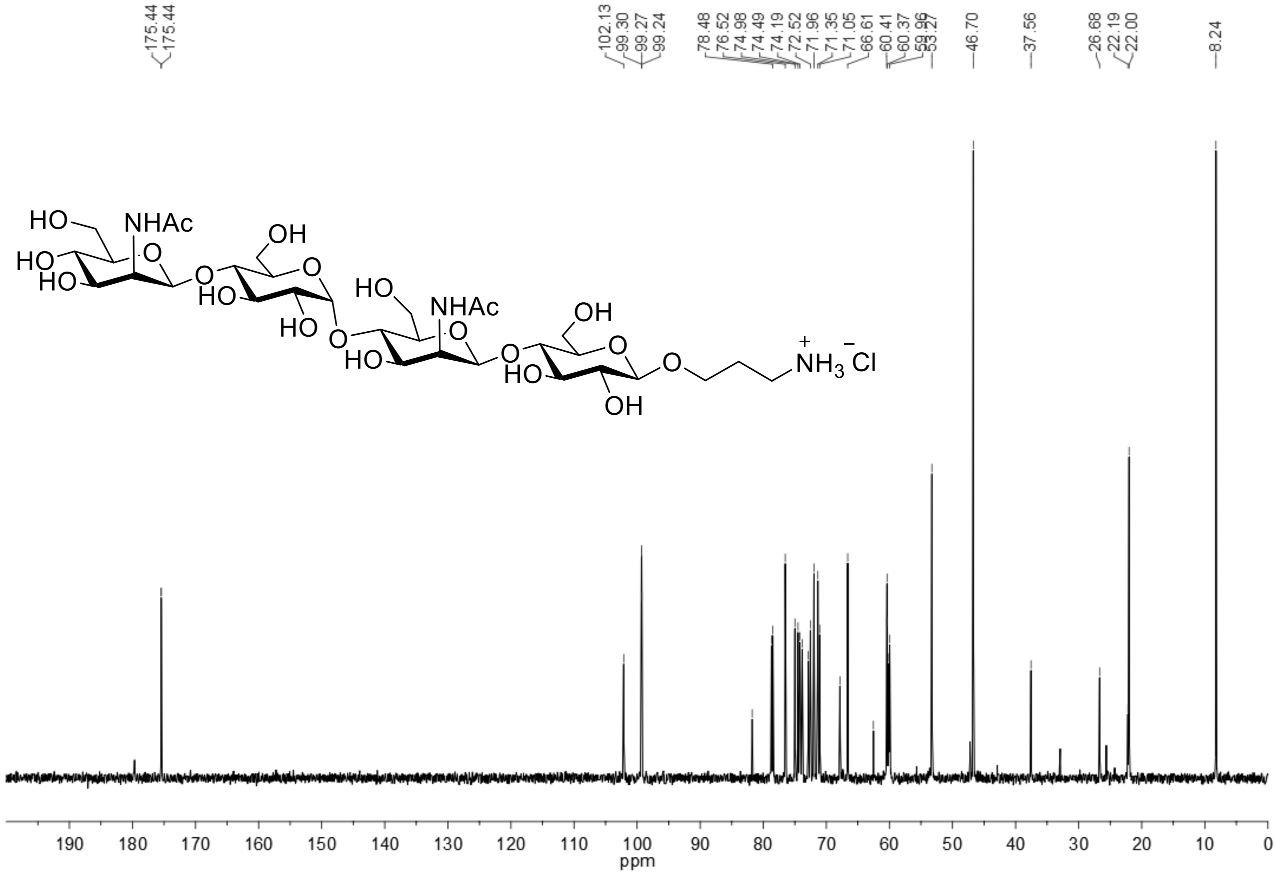

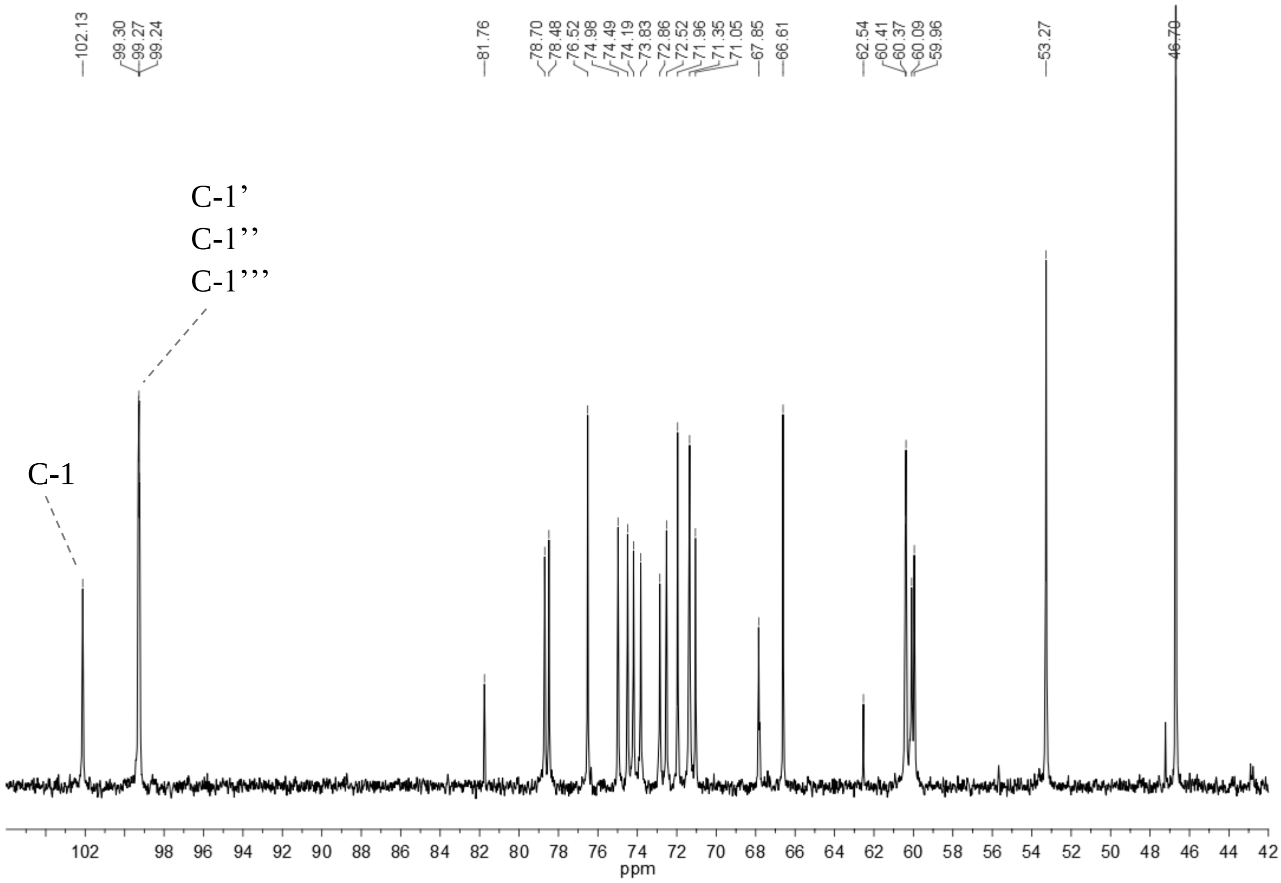

(22a): ^{1}H NMR (500 MHz, $CDCl_3$)

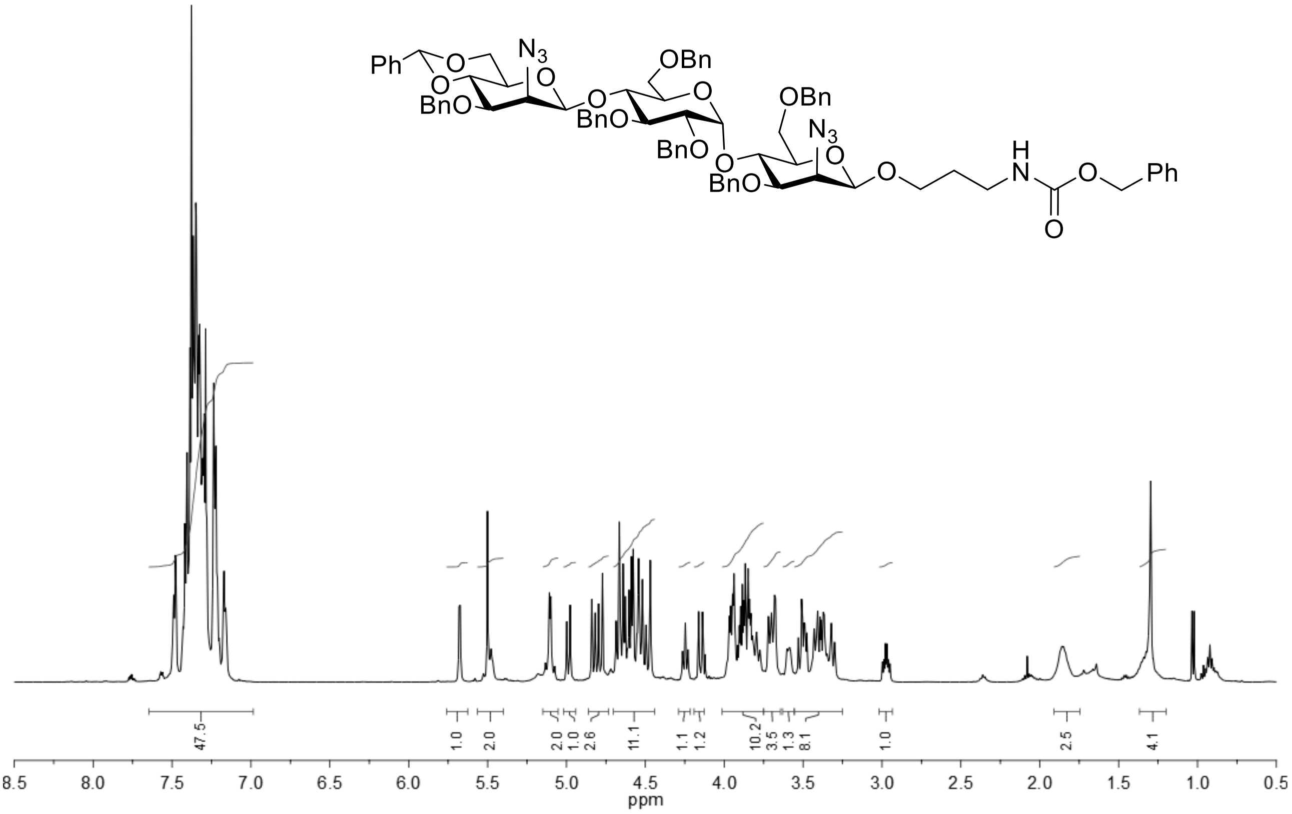

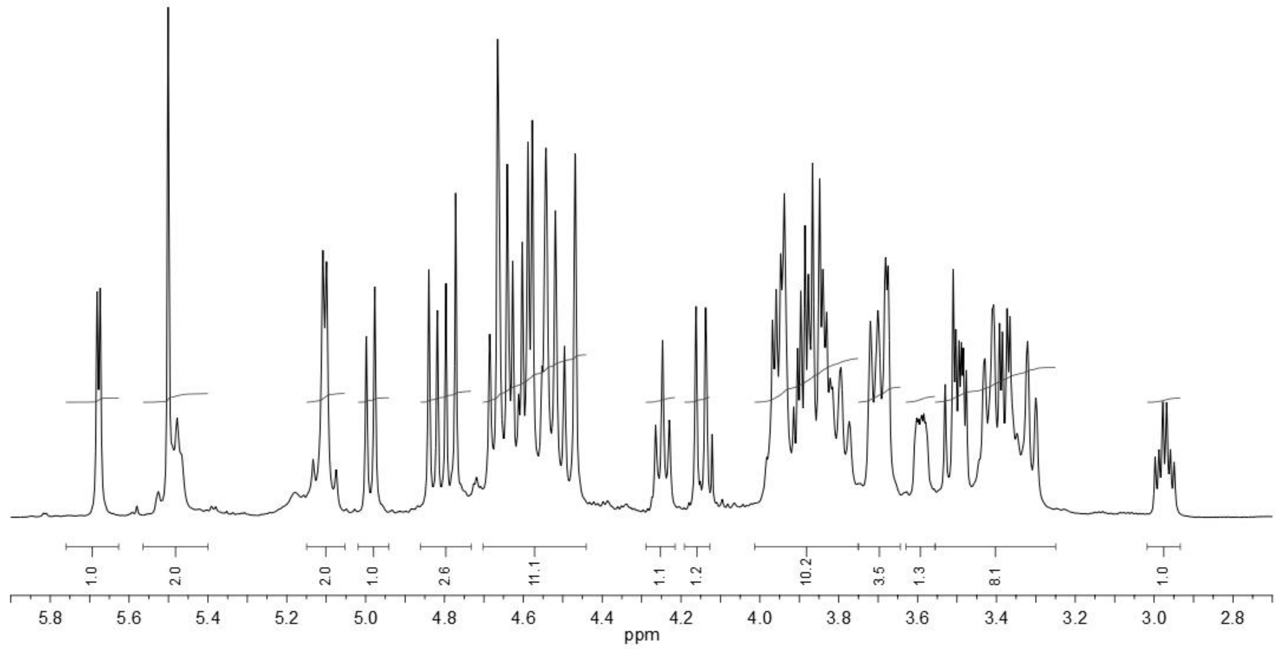

(22a): ^{13}C NMR (126 MHz, CDCl$_3$)

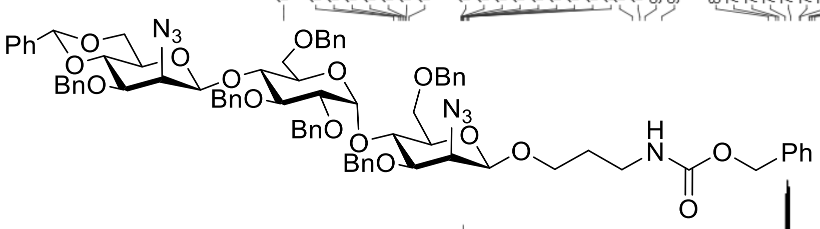

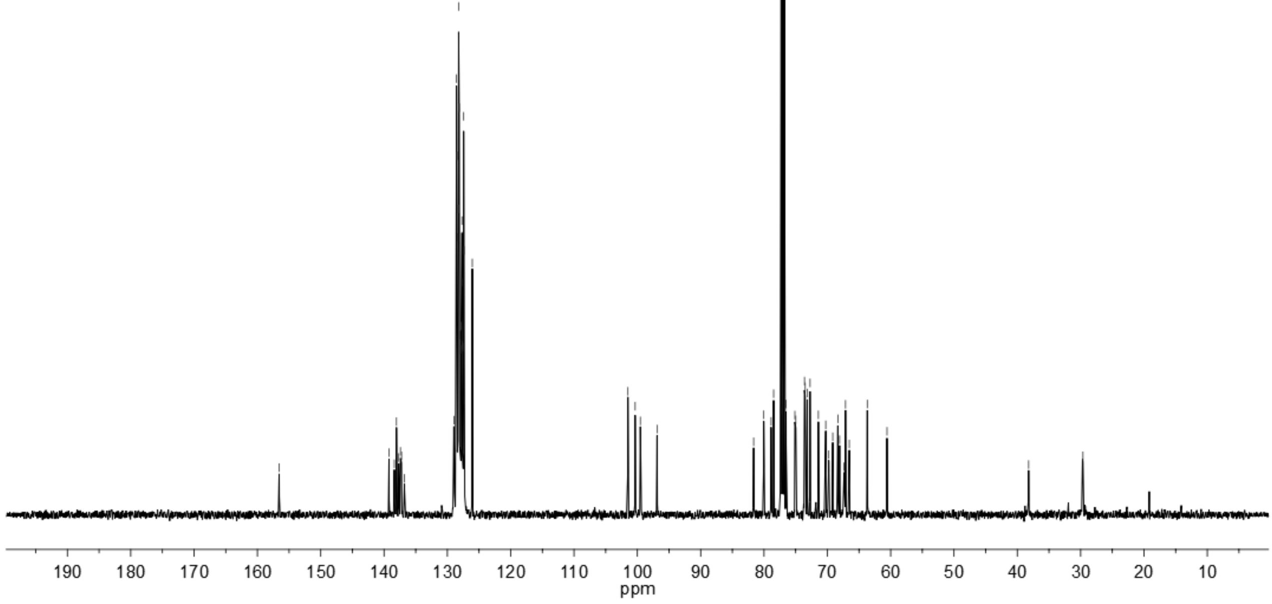

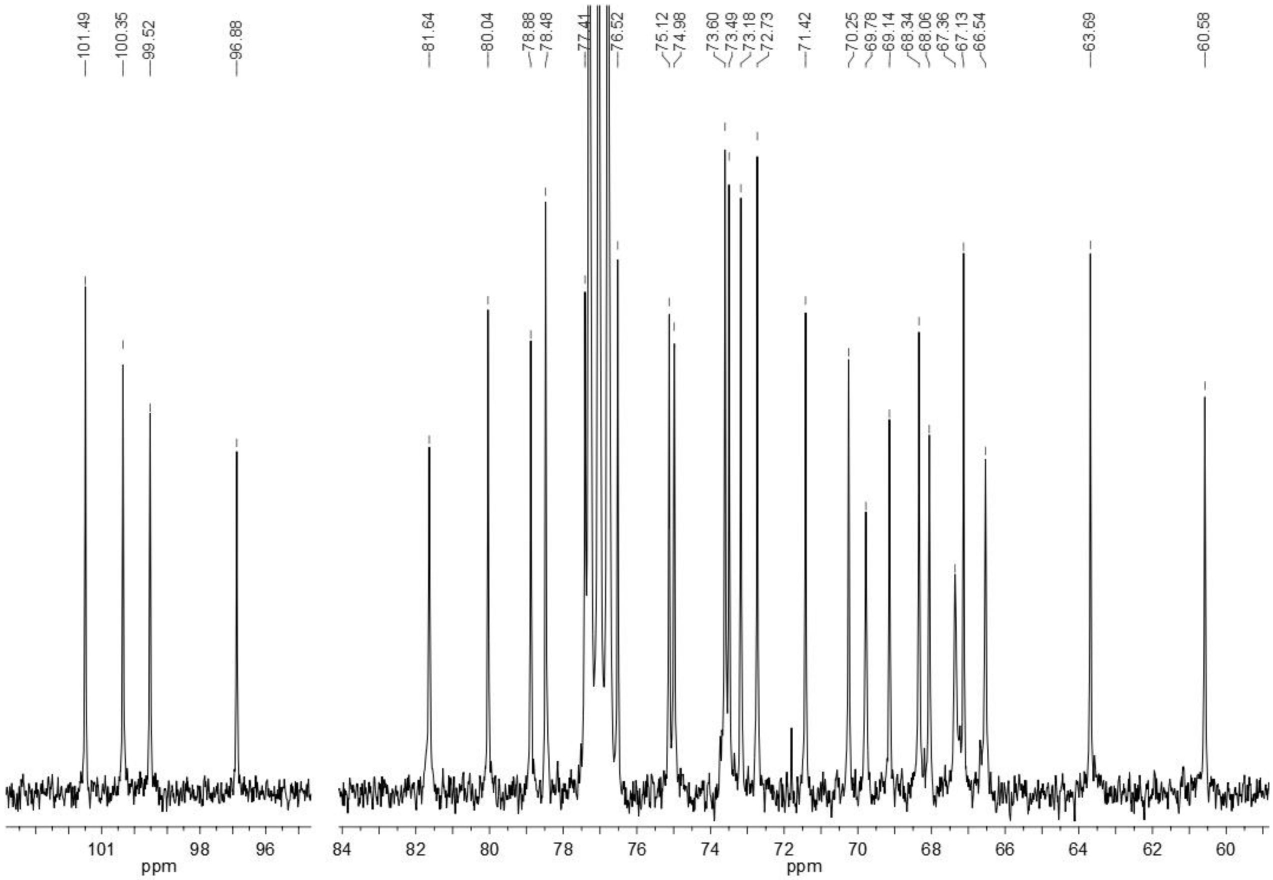

(22b): ^{1}H NMR (500 MHz, $CDCl_3$)

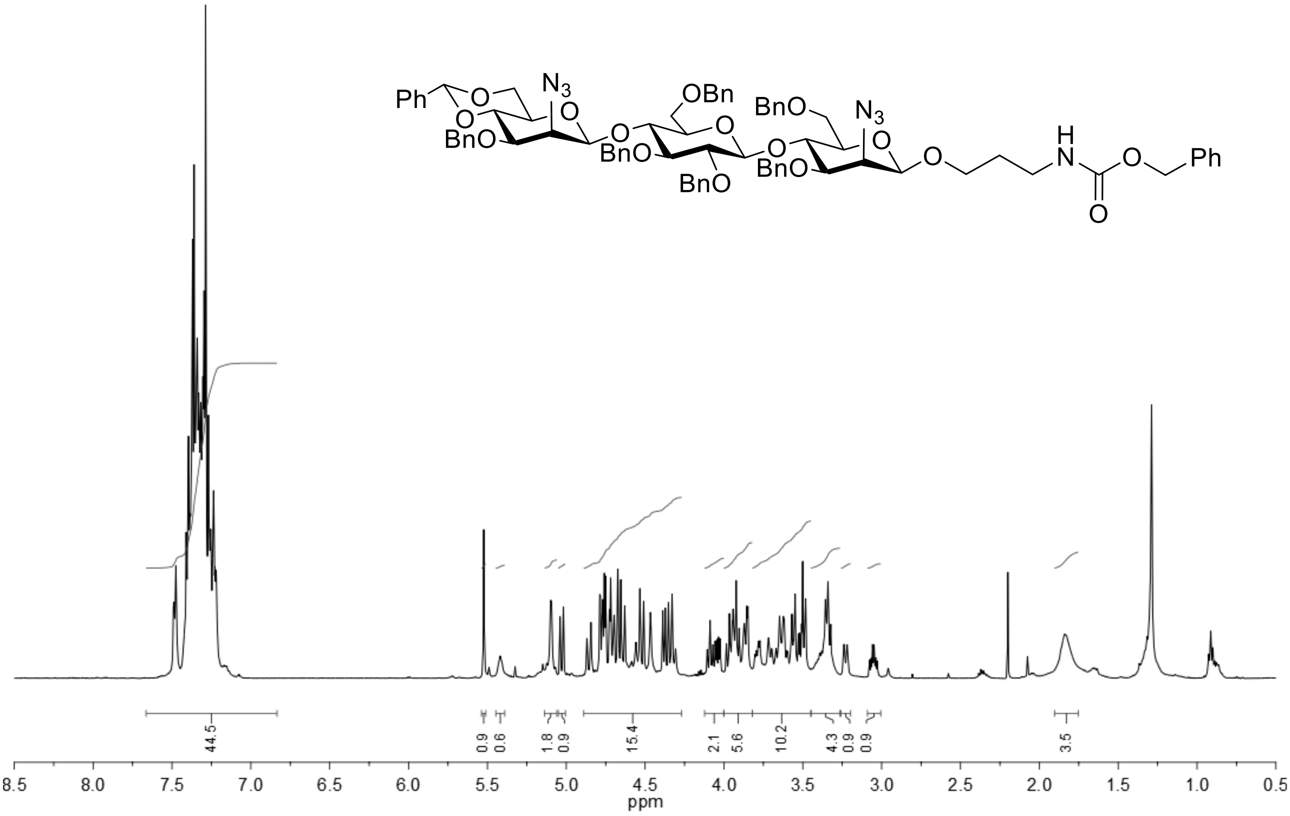

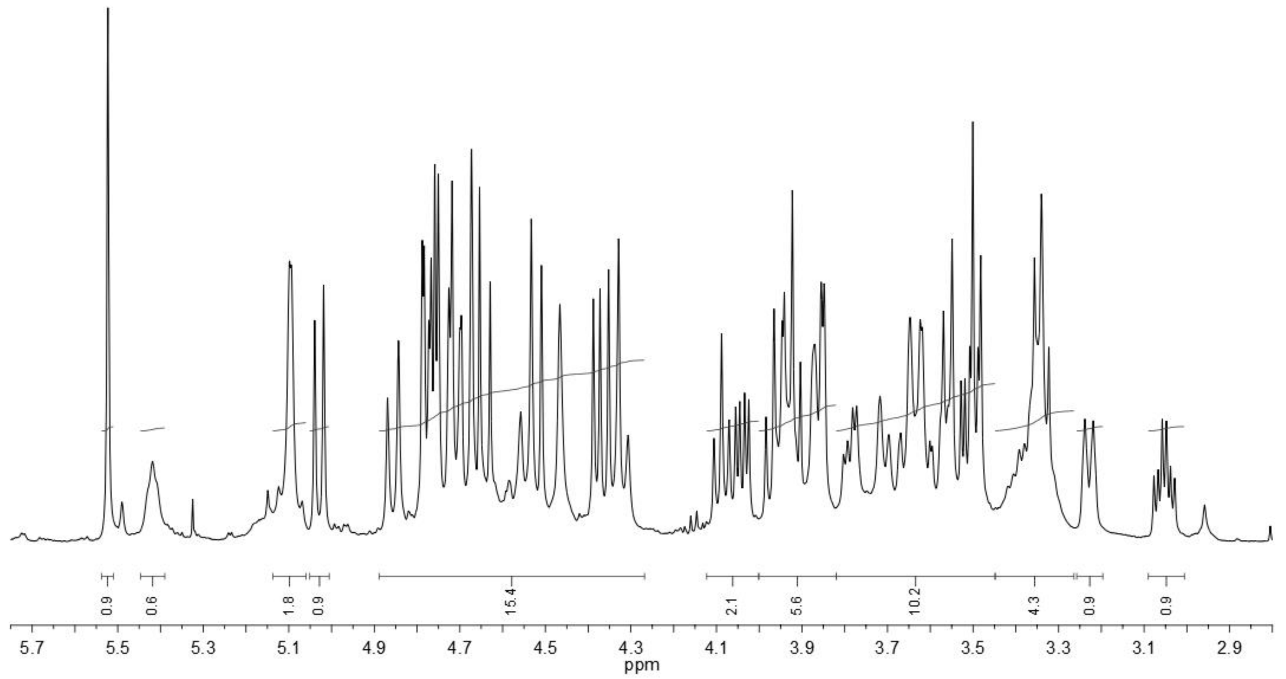

(22b): ^{13}C NMR (126 MHz, CDCl$_3$)

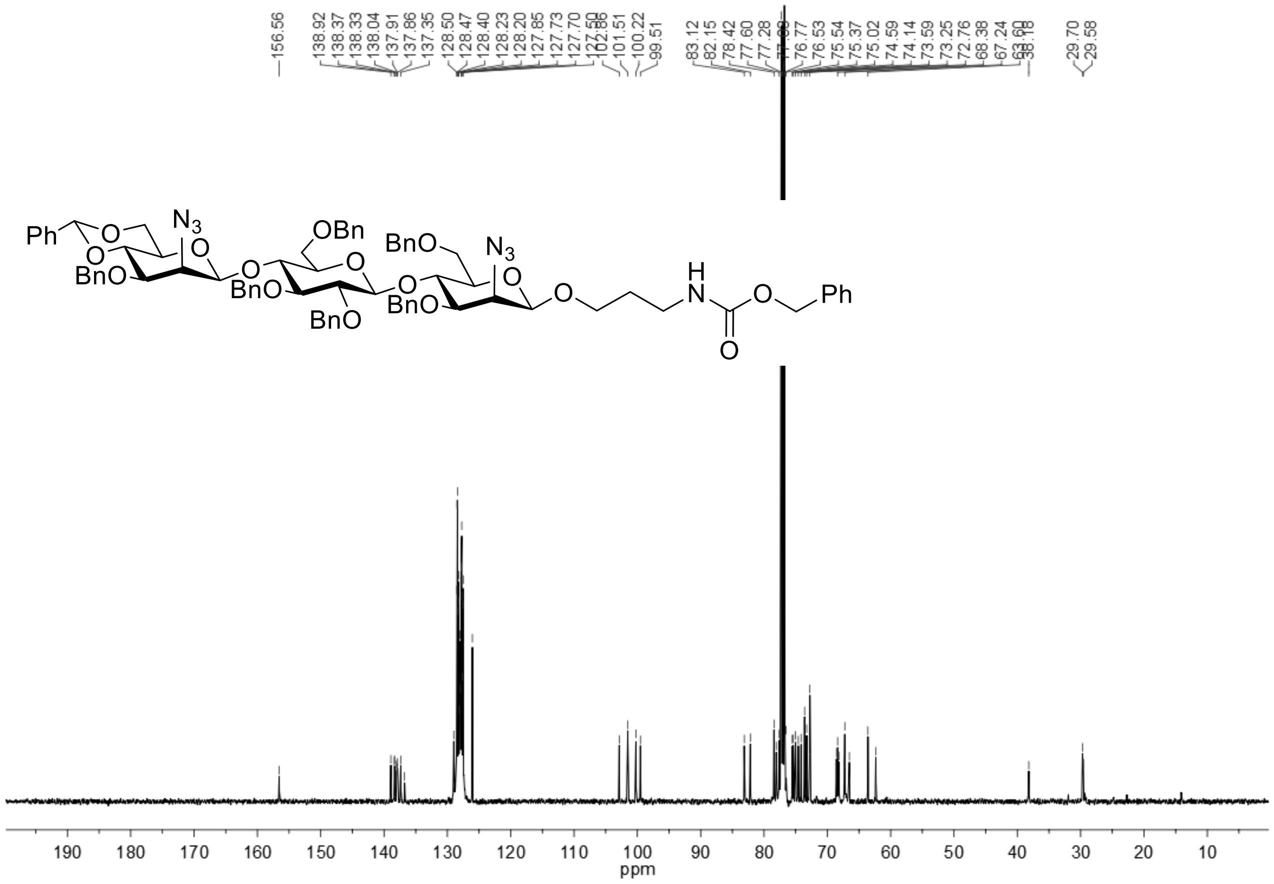

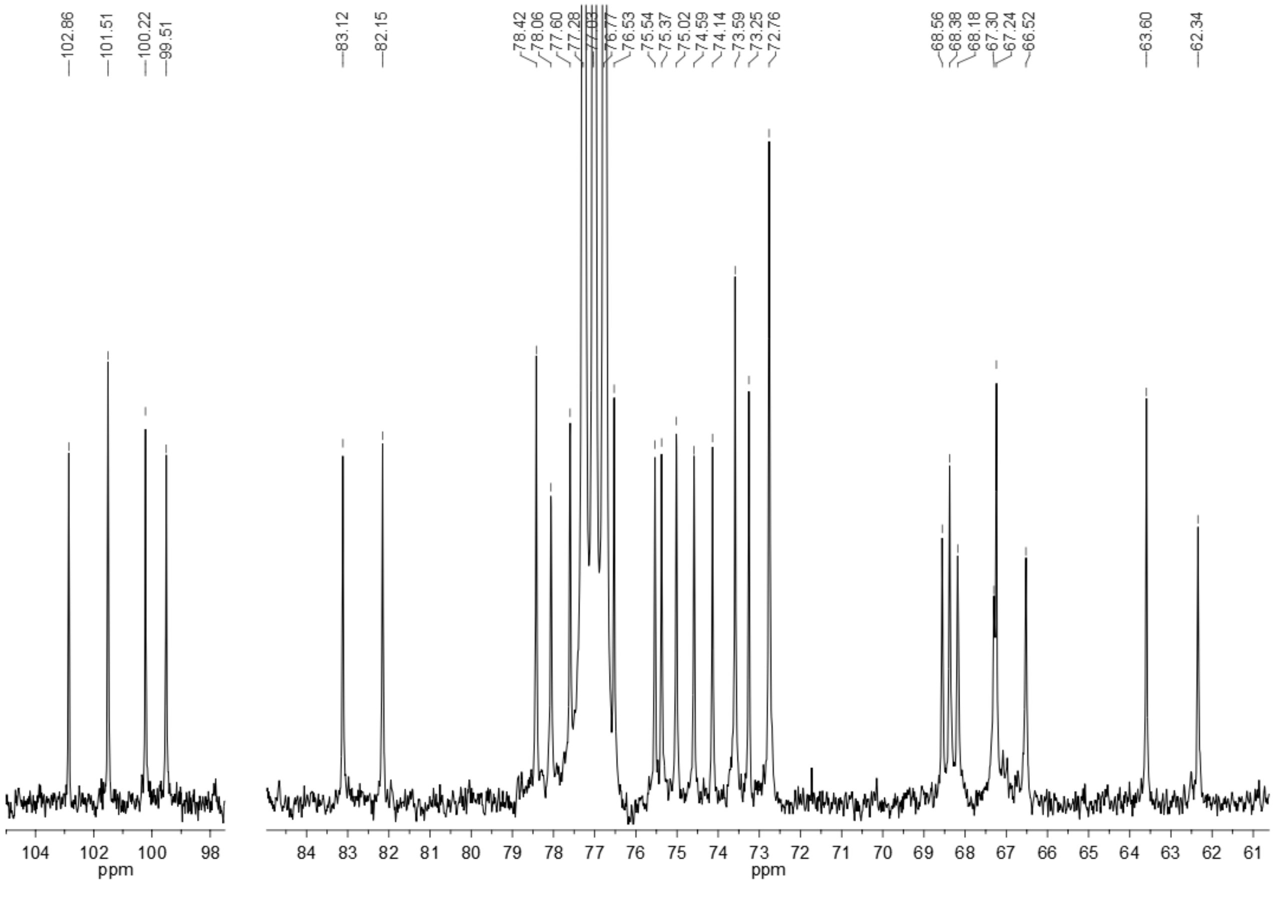

(23a): ^{1}H NMR (500 MHz, $CDCl_3$)

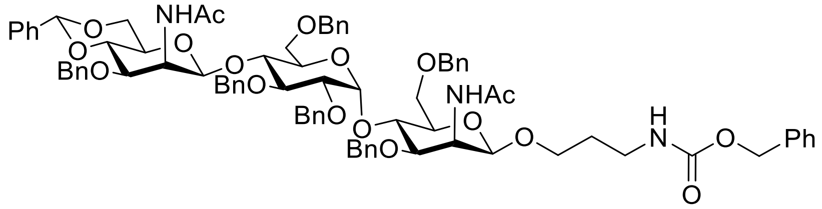

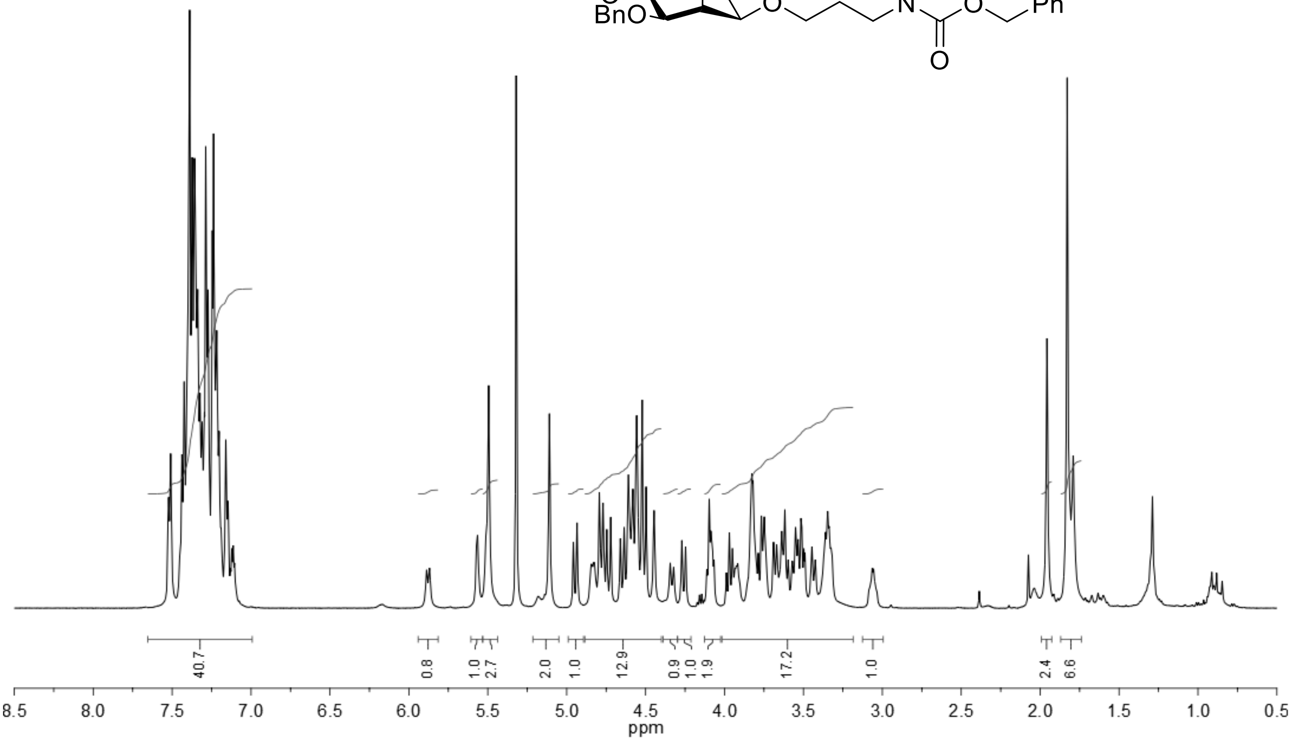

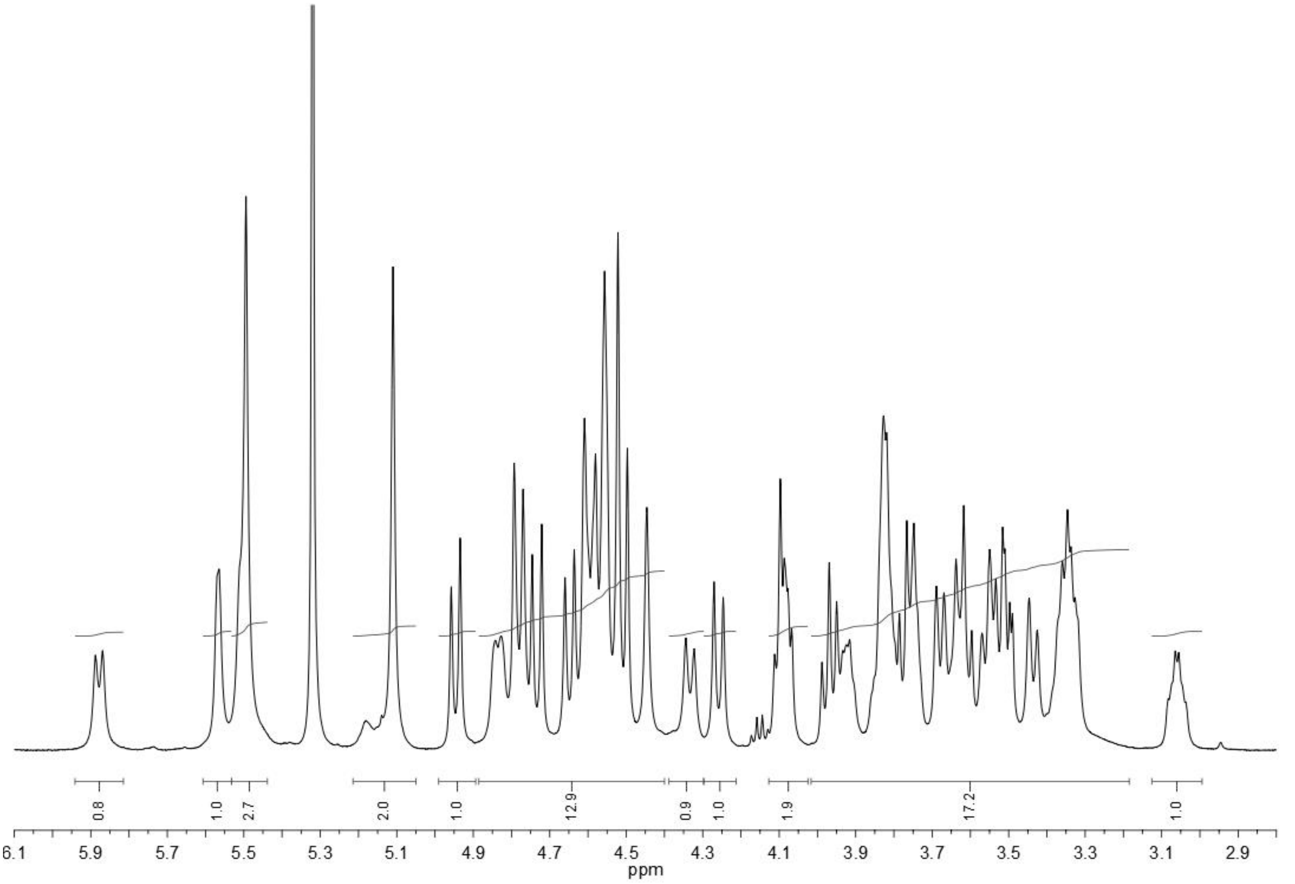

(23a): ^{13}C NMR (126 MHz, CDCl$_3$)

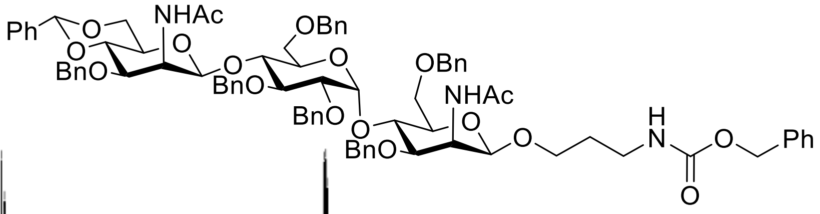

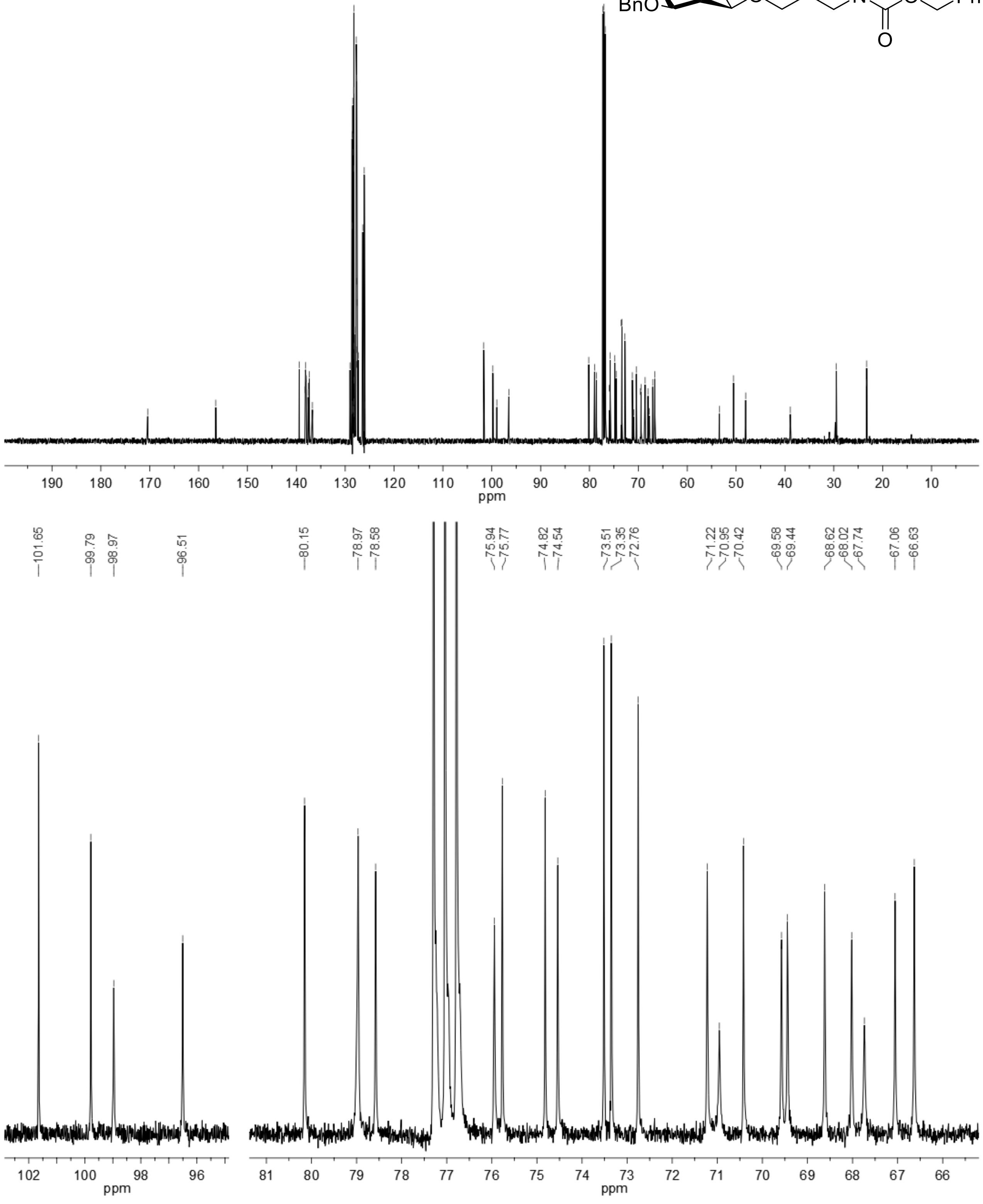

(3): ^{1}H NMR (500 MHz, D_2O)

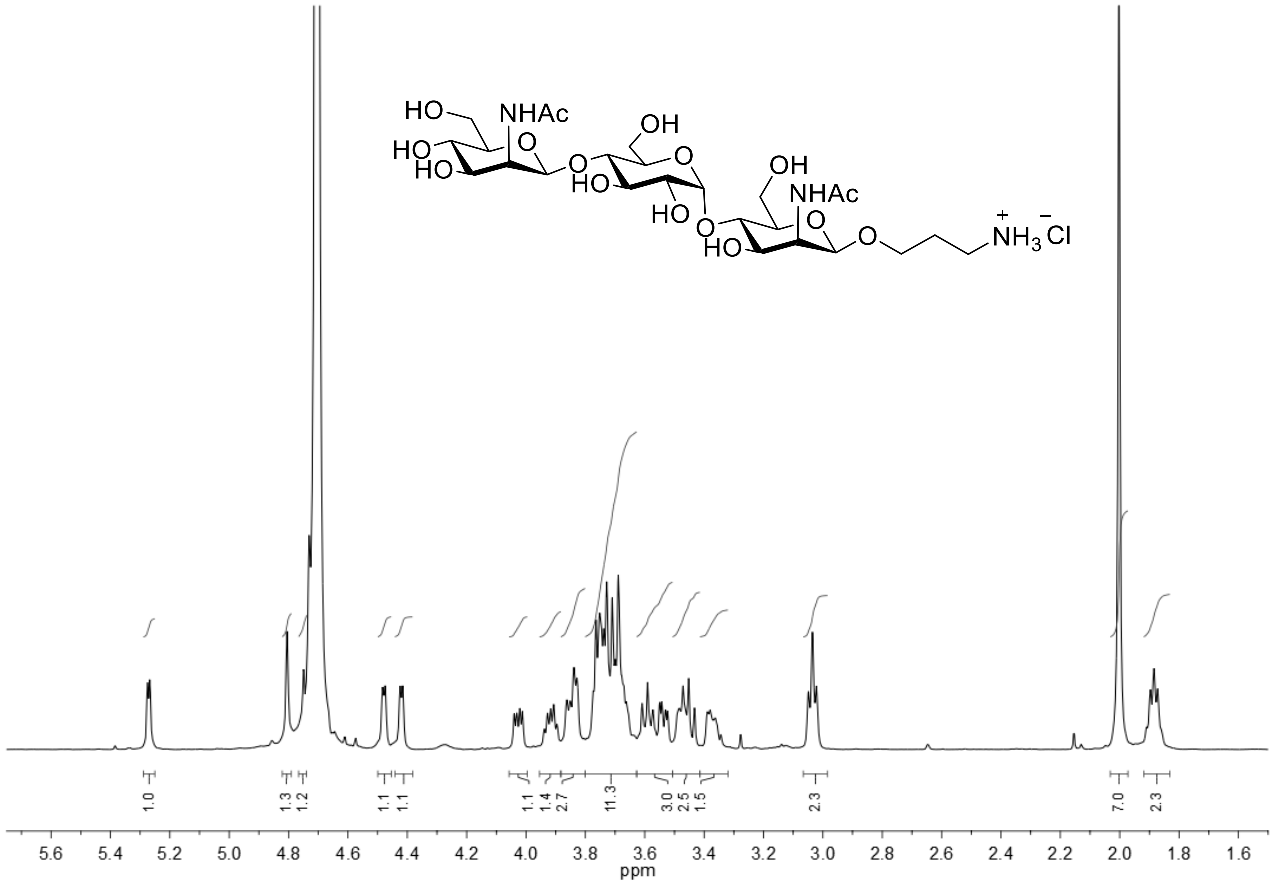

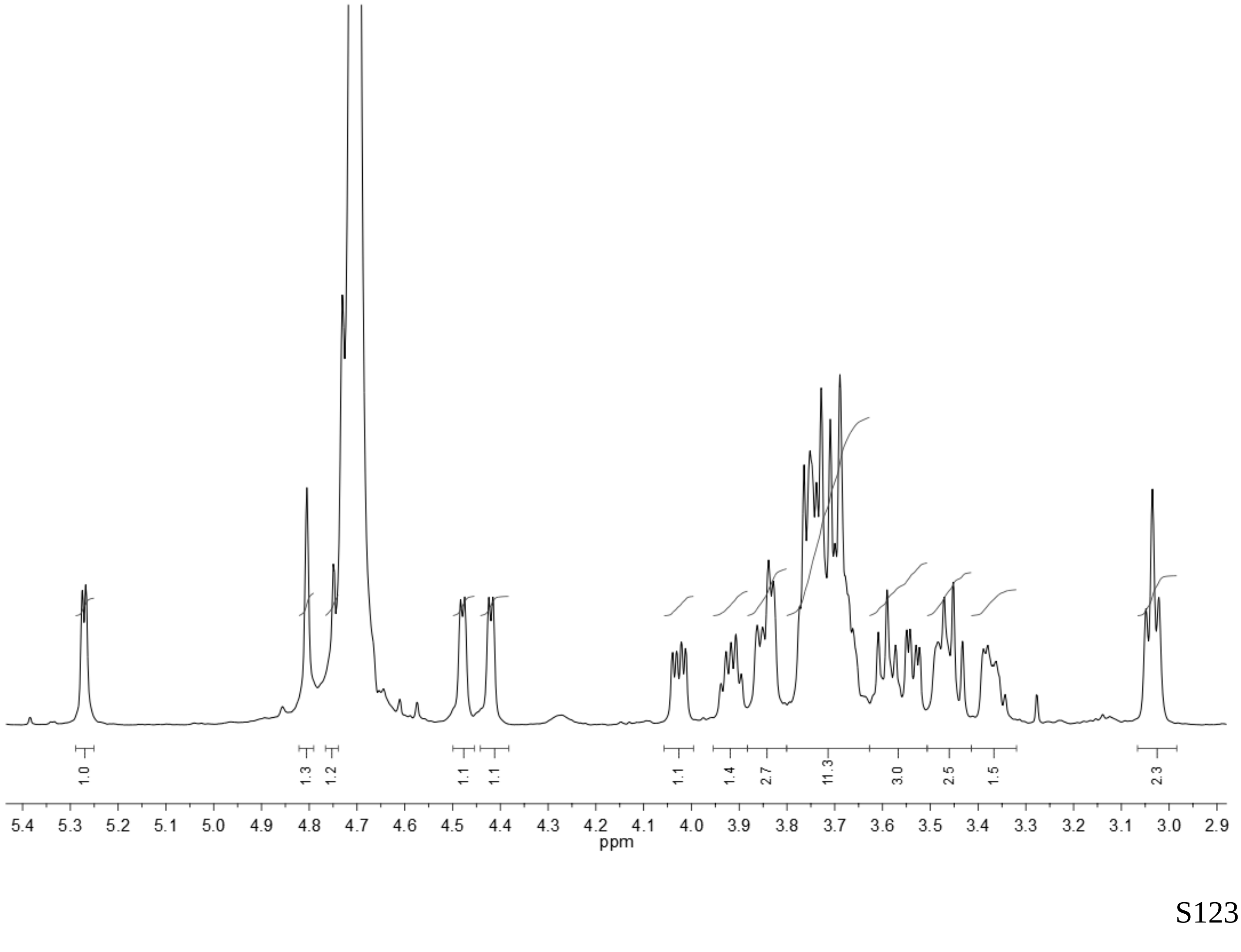

(3): ^{13}C NMR (126 MHz, D$_2$O)

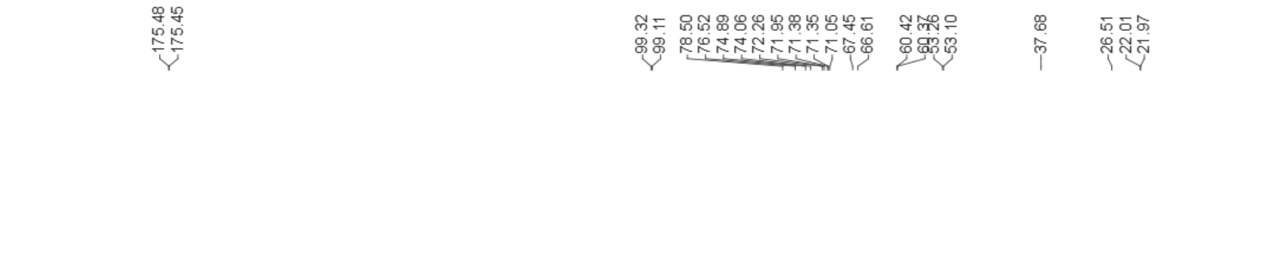

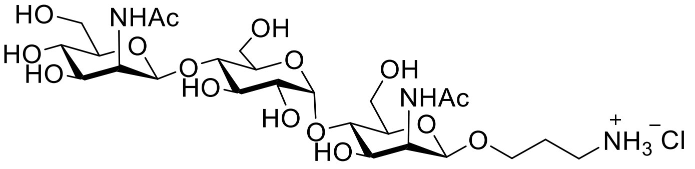

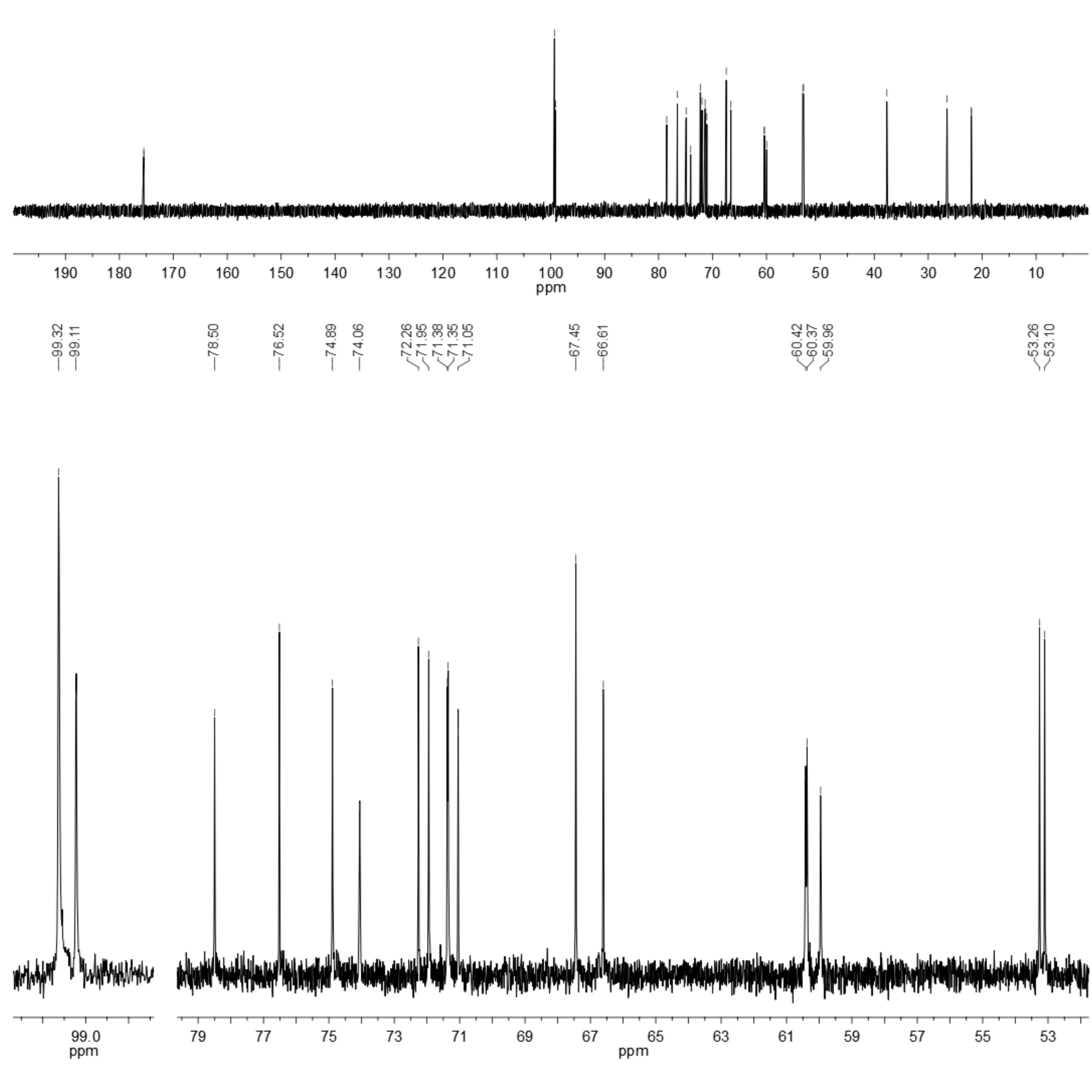

(23b): ^{1}H NMR (500 MHz, $CDCl_3$)

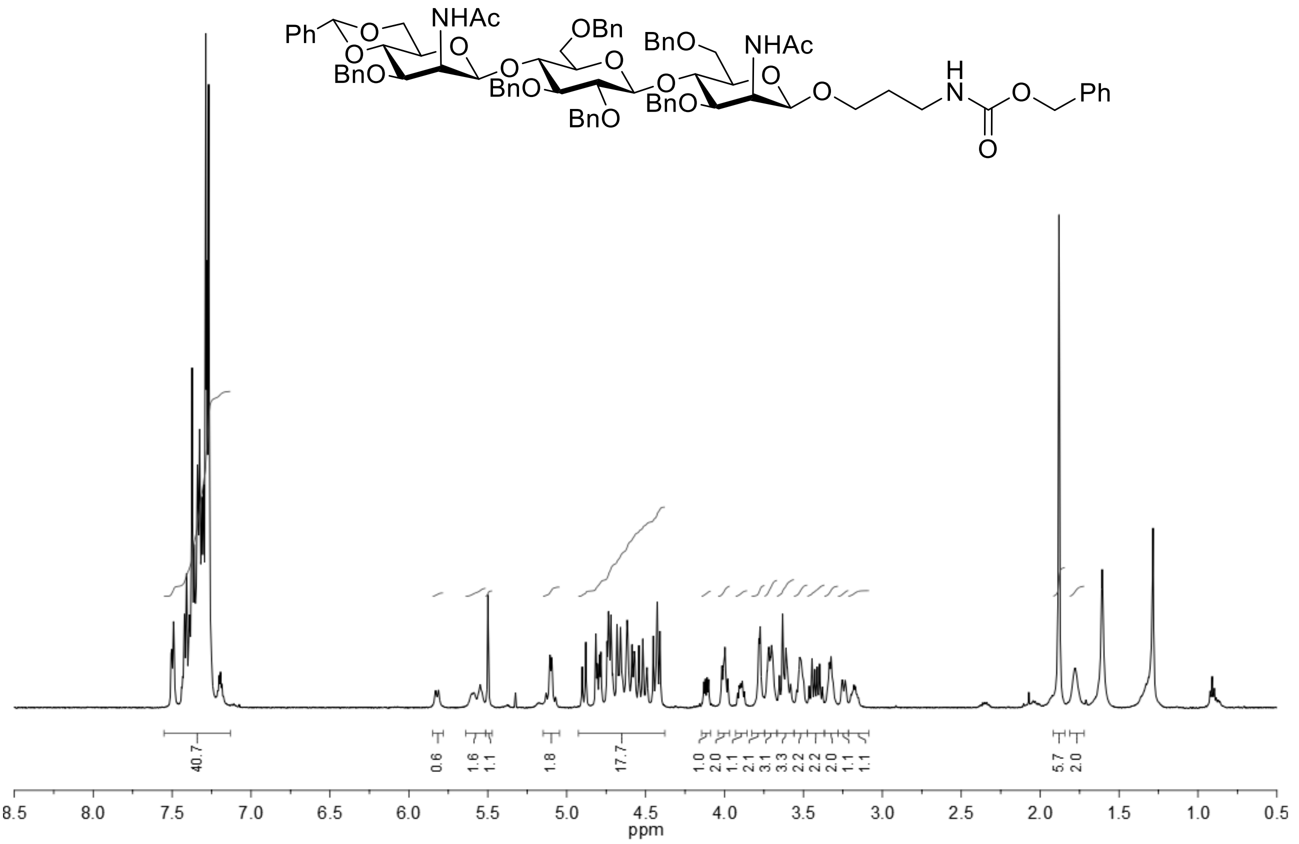

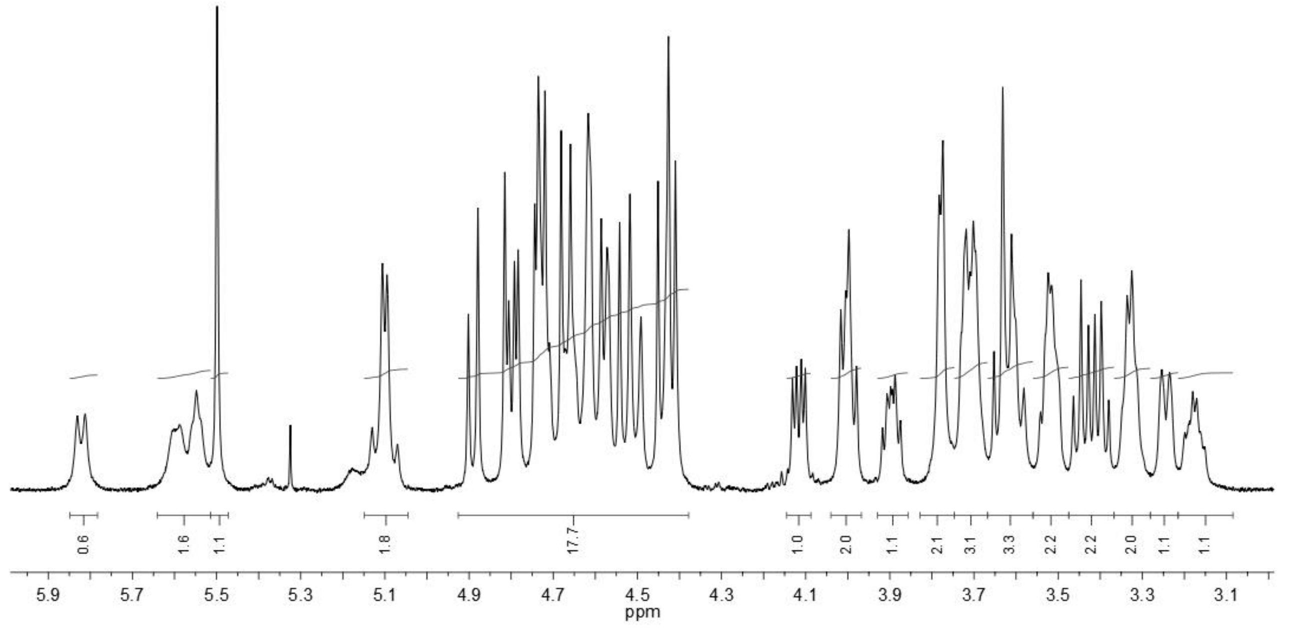

(23b): ^{13}C NMR (126 MHz, $CDCl_3$)

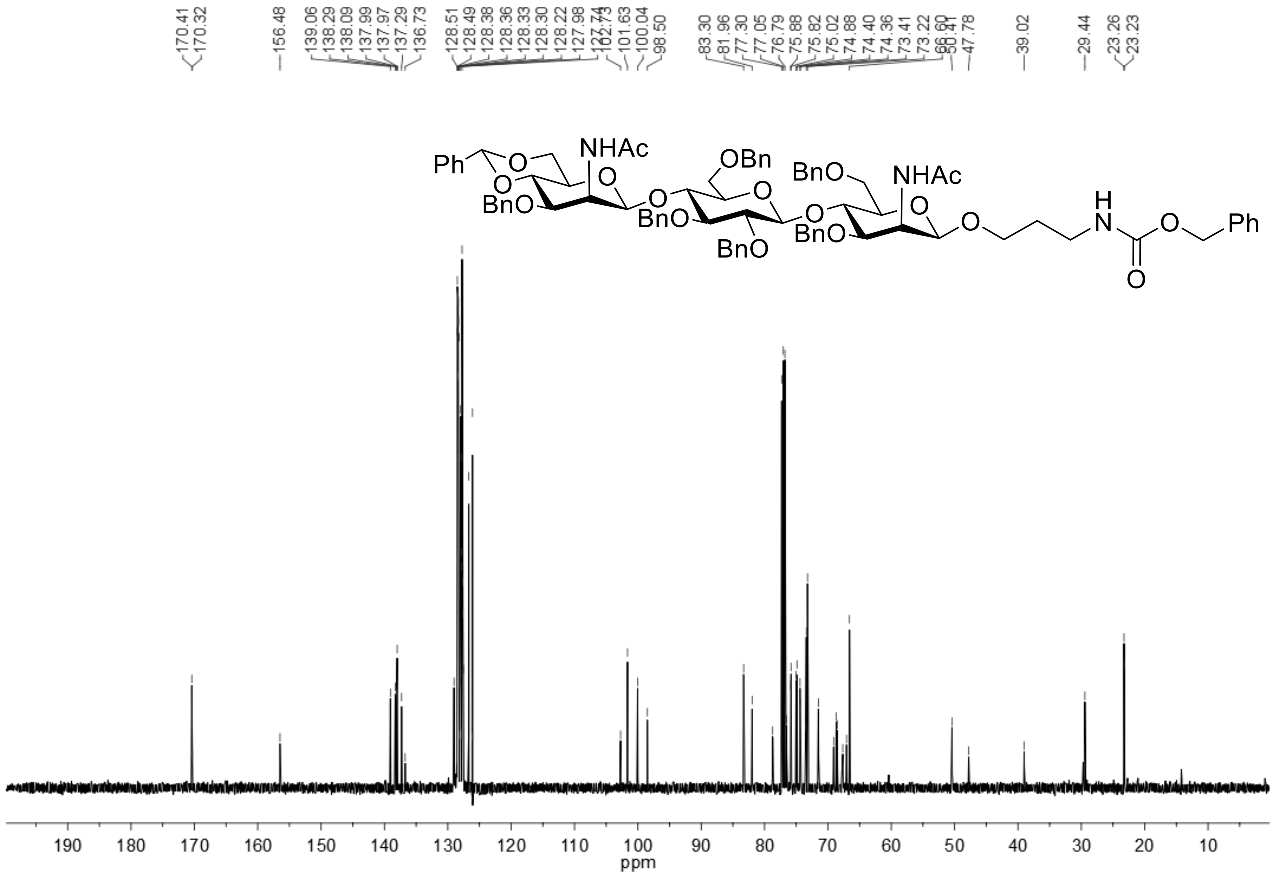

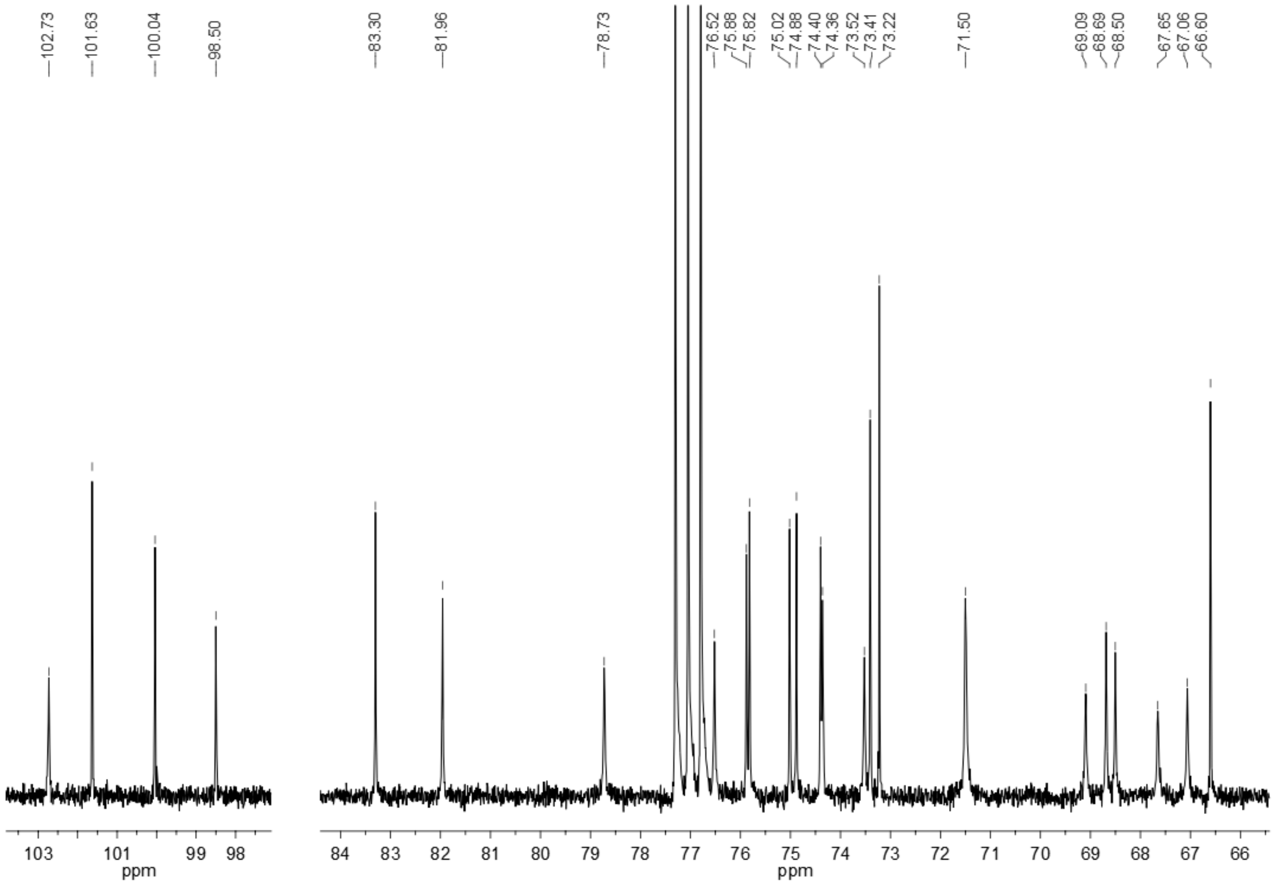

(4): ^{1}H NMR (500 MHz, D_2O)

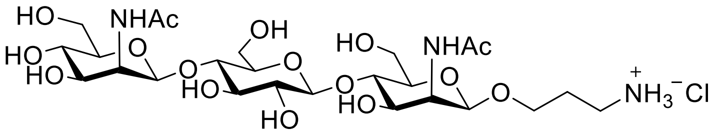

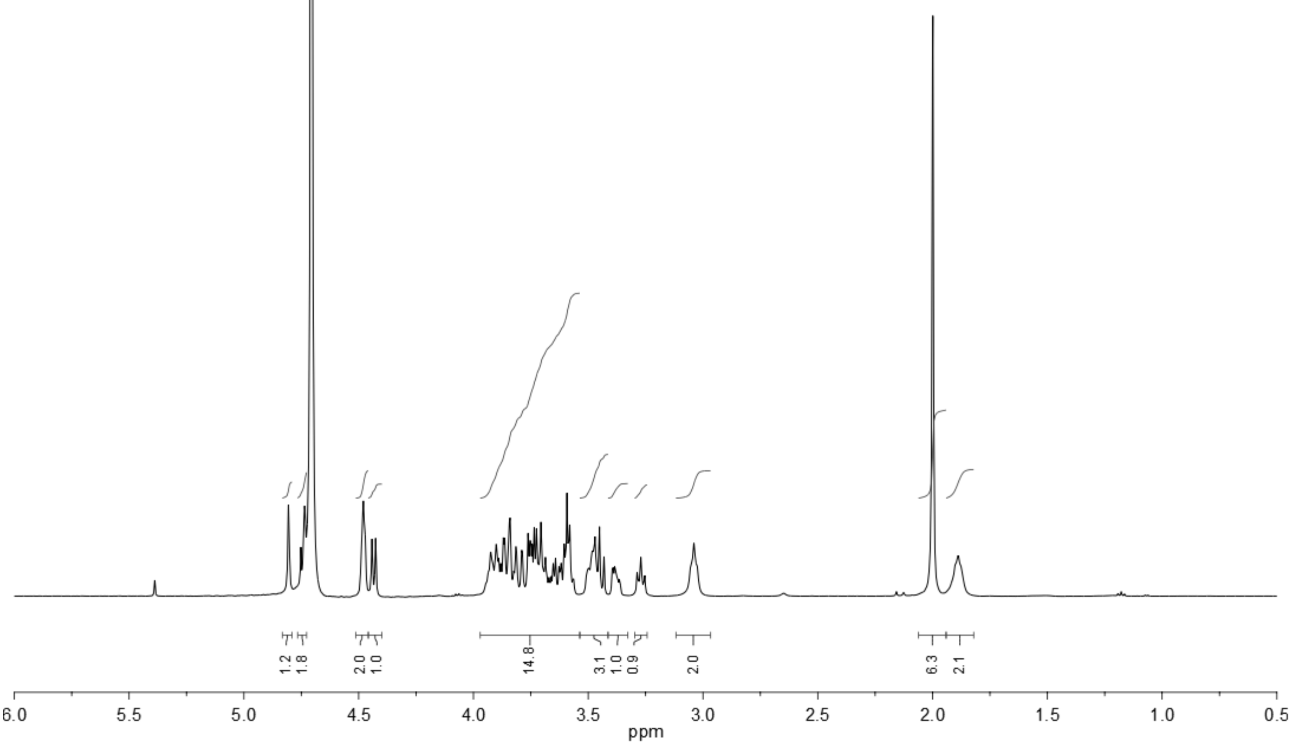

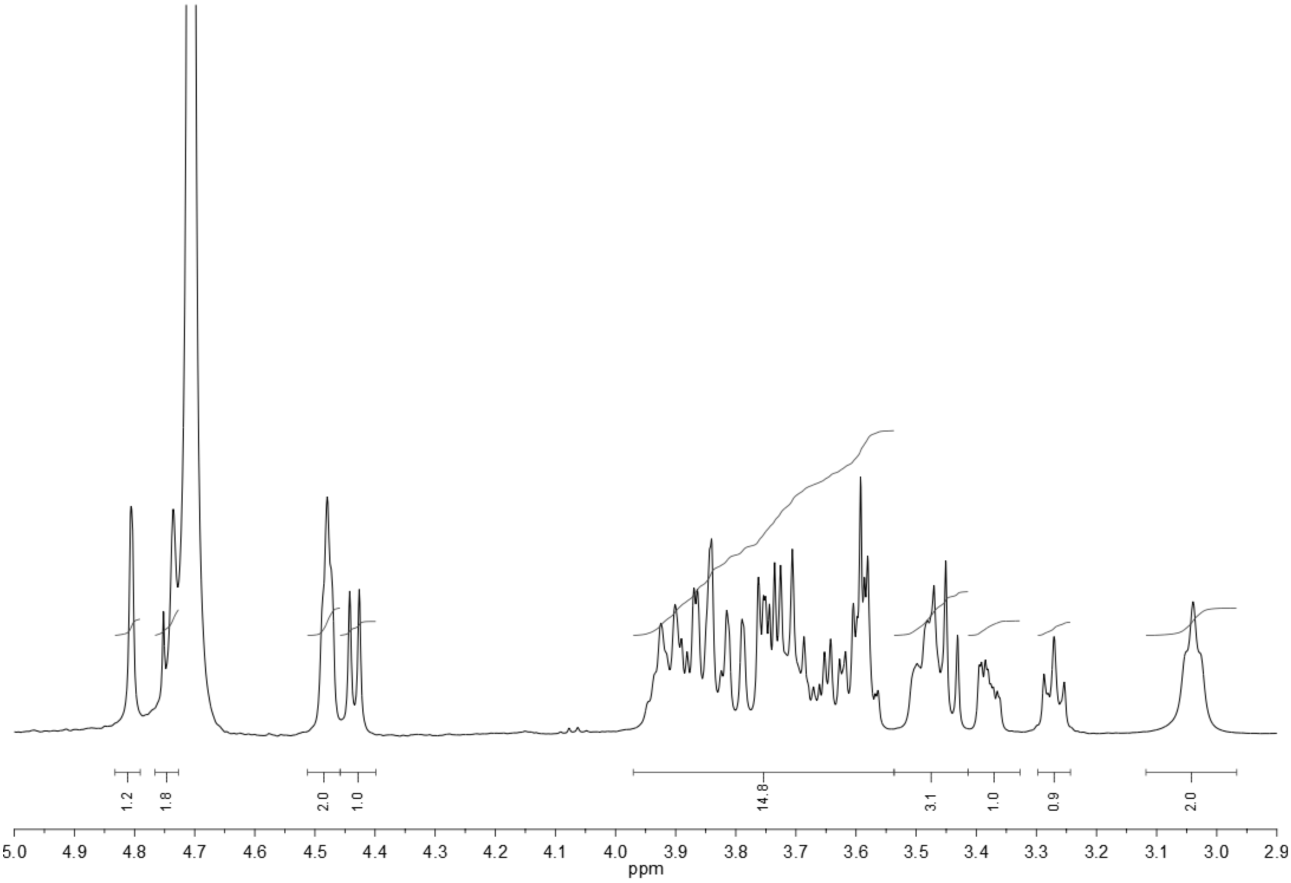

(4): ^{13}C NMR (126 MHz, D_2O)

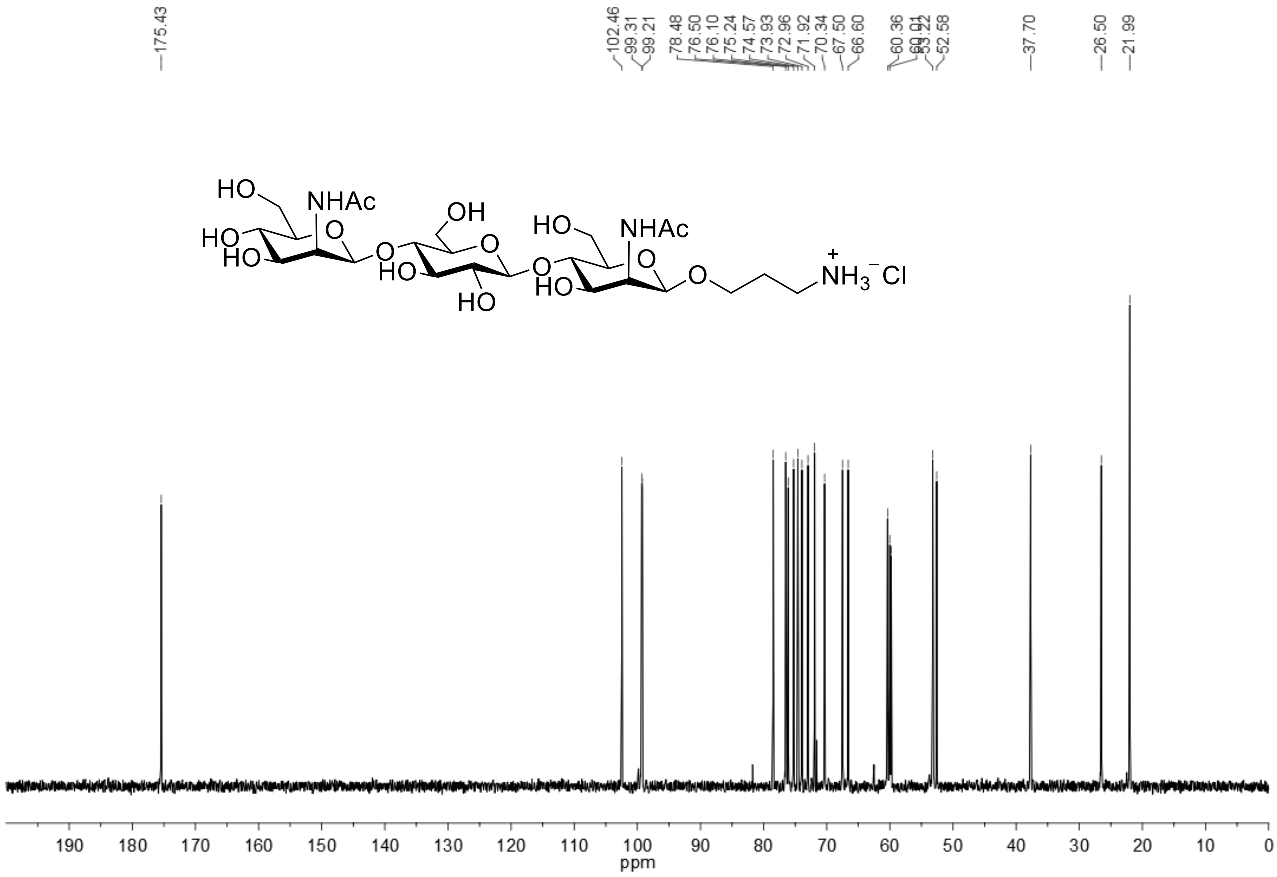

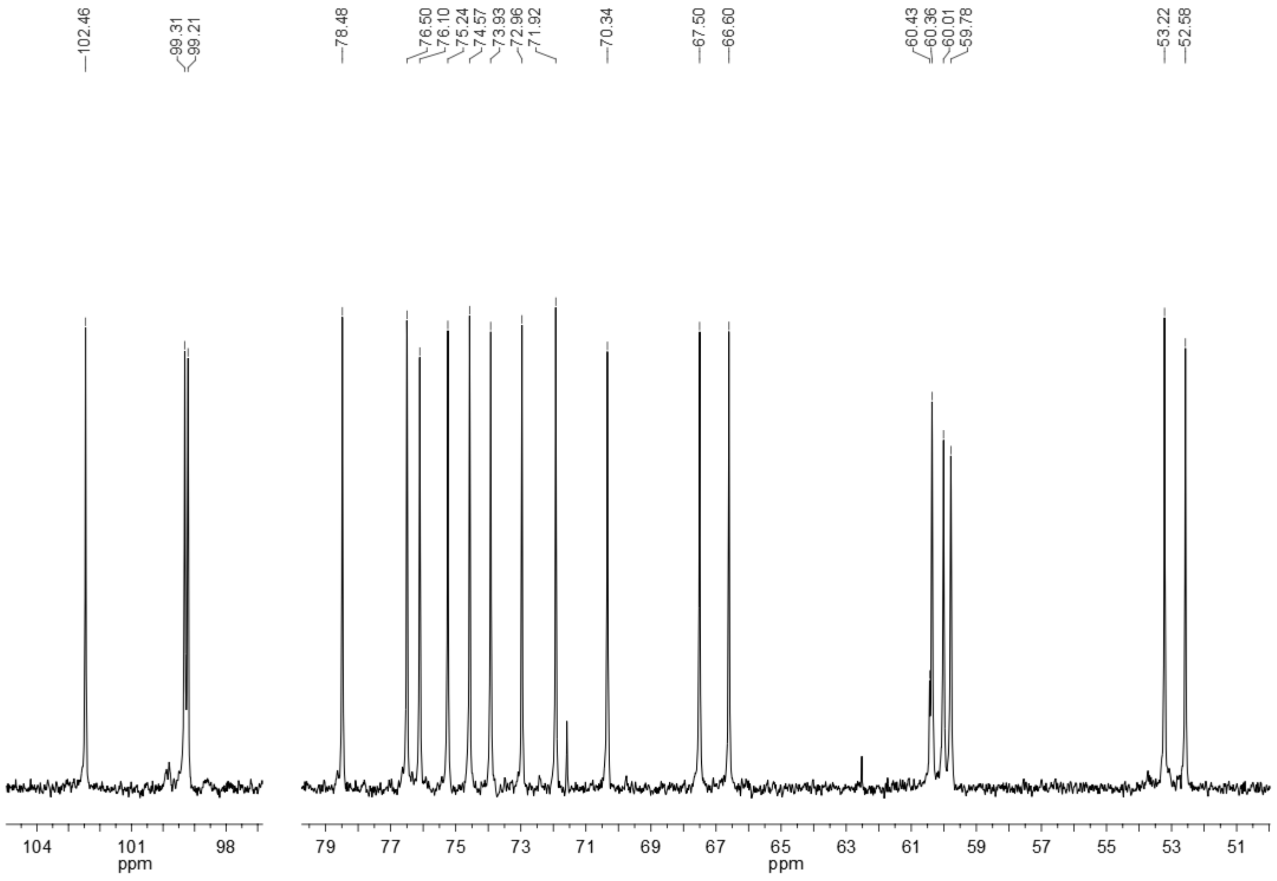

(24): ^{1}H NMR (500 MHz, CDCl$_3$)

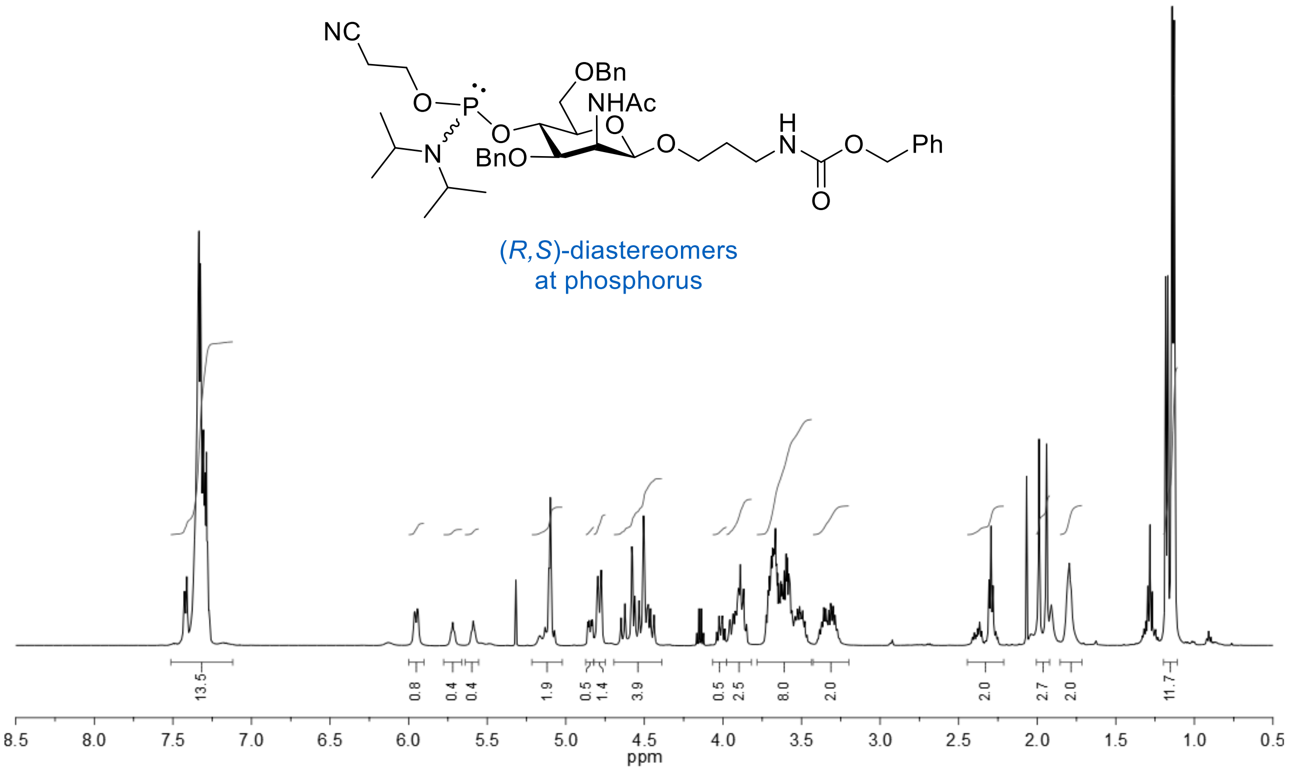

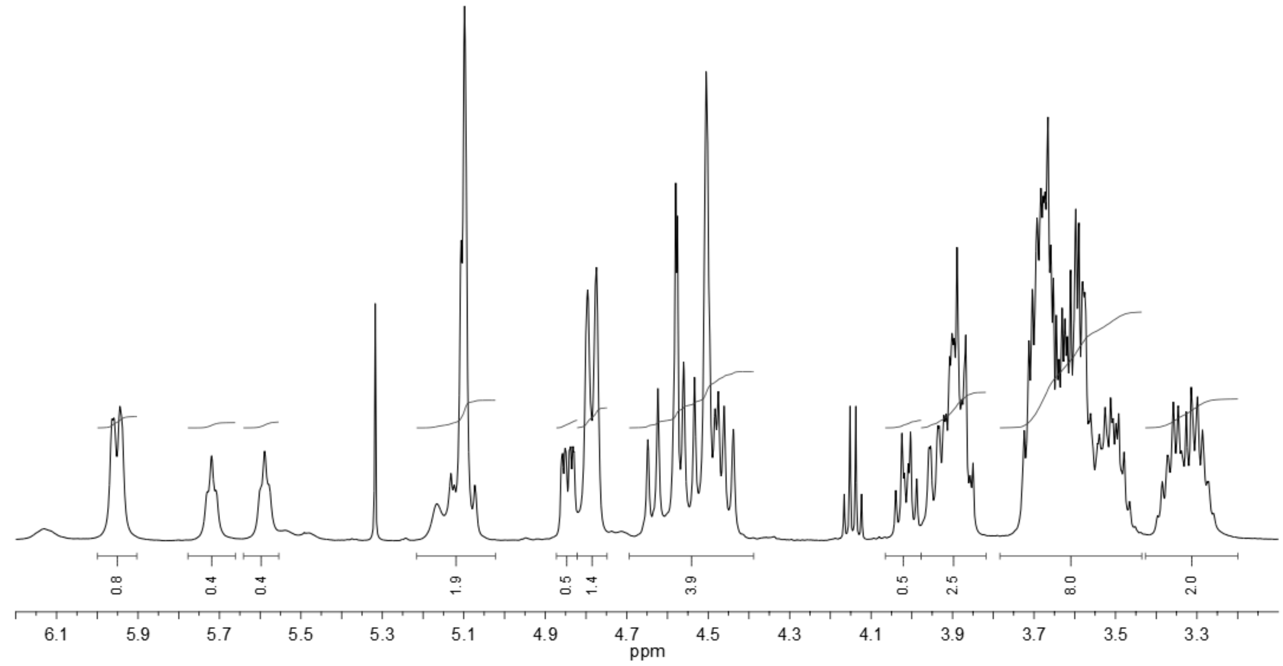

(24): ^{31}P NMR (202 MHz, CDCl$_3$)

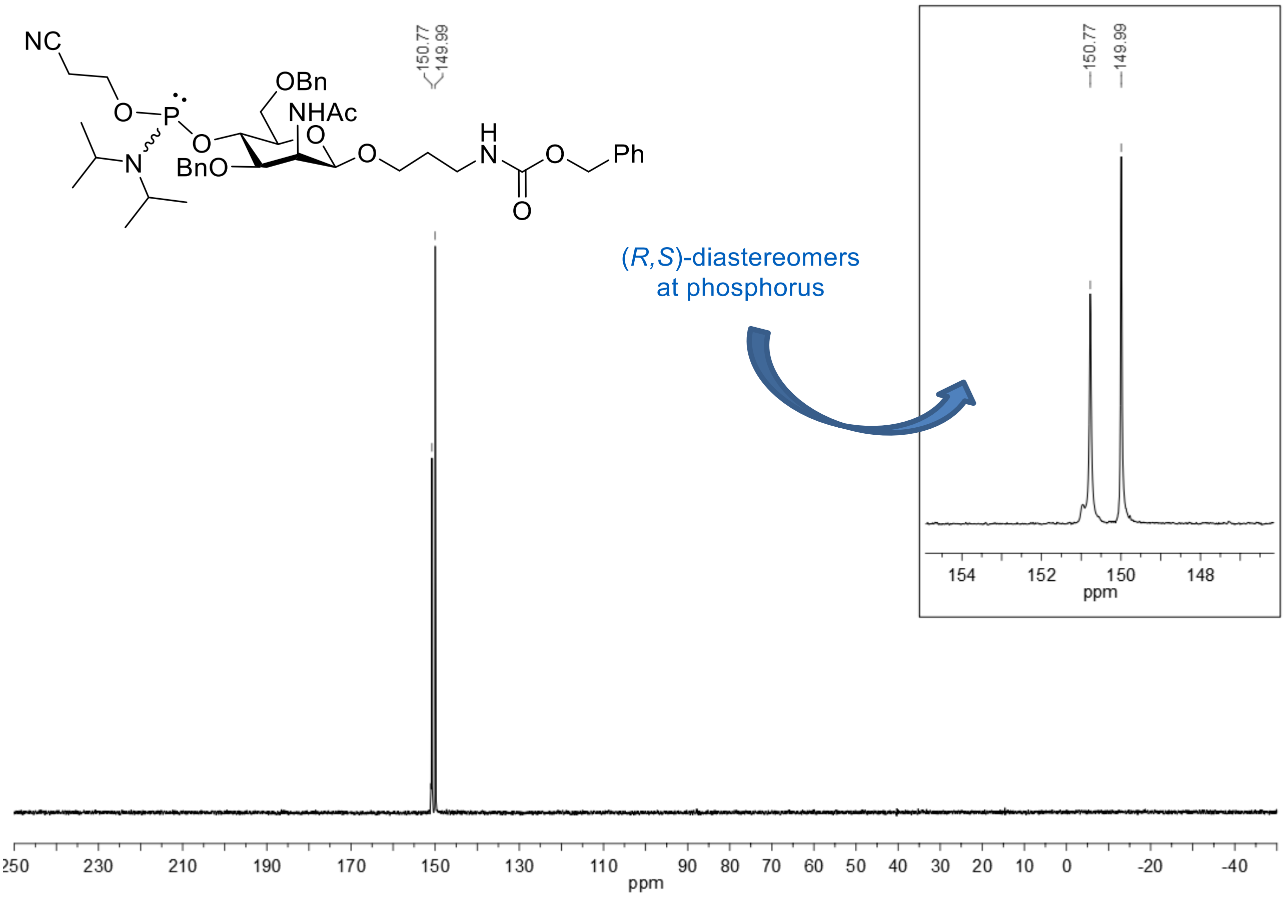

(24): ^{1}H-^{31}P HMQC NMR (CDCl$_3$)

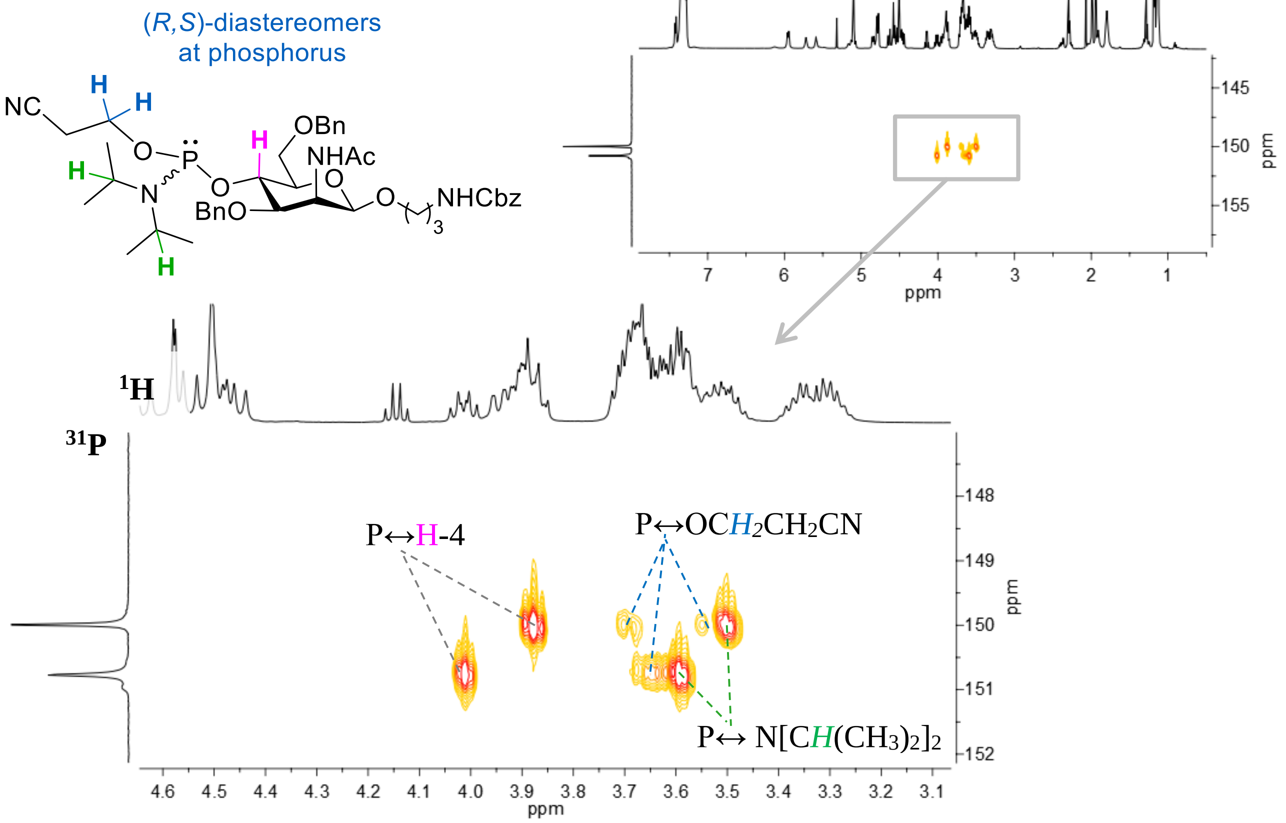

(24): ^{13}C NMR (126 MHz, CDCl$_3$)

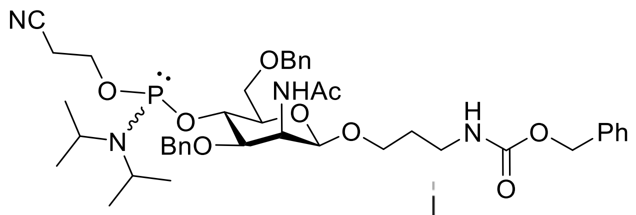

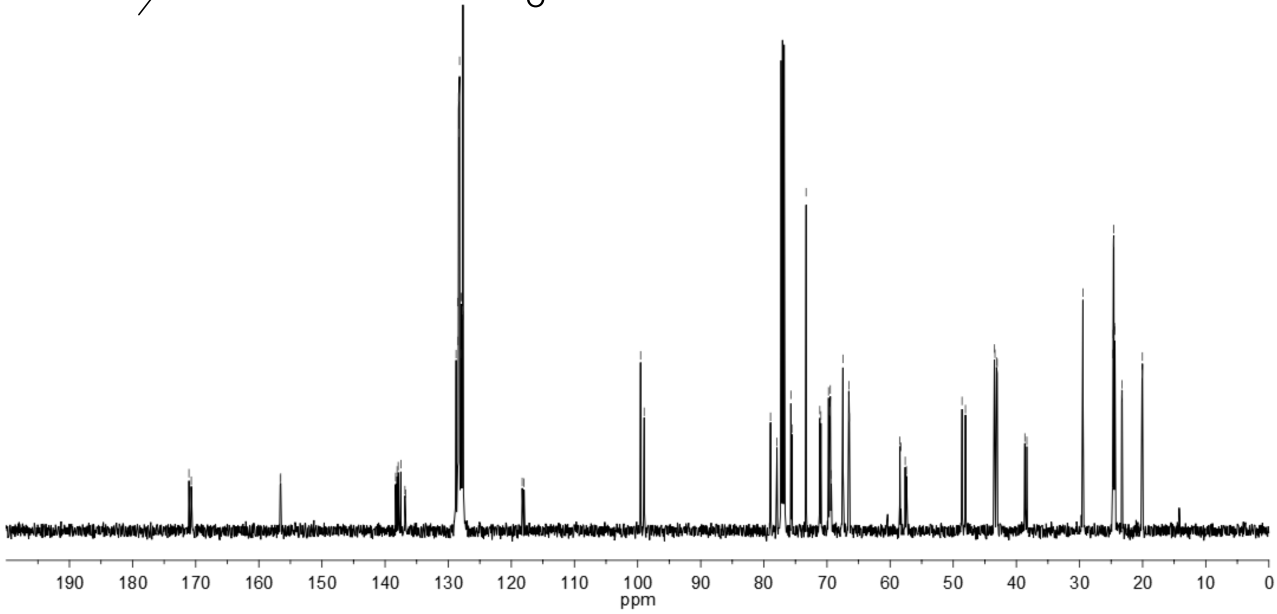

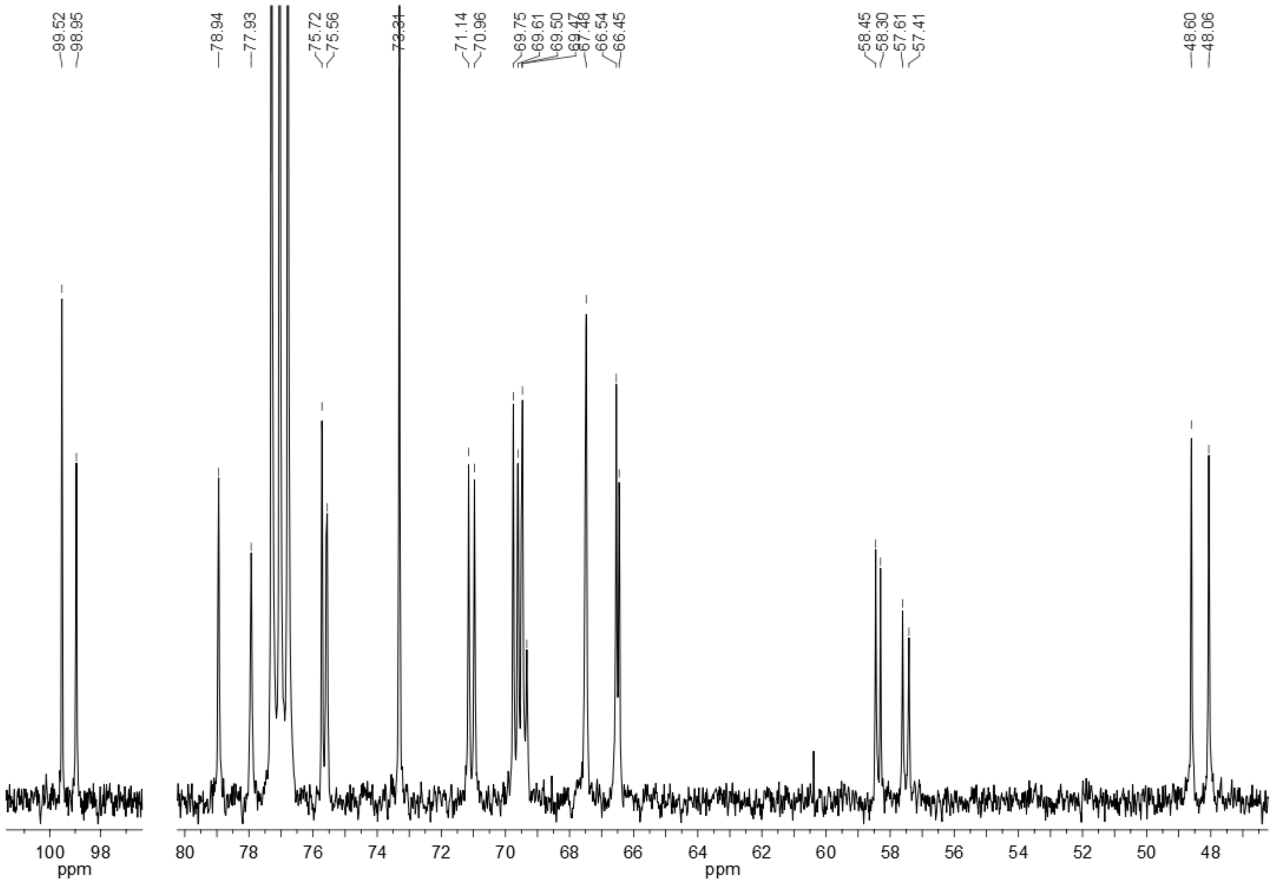

(25, crude): ^{3}H NMR (500 MHz, CDCl$_3$)

mixture of 4 diastereomers

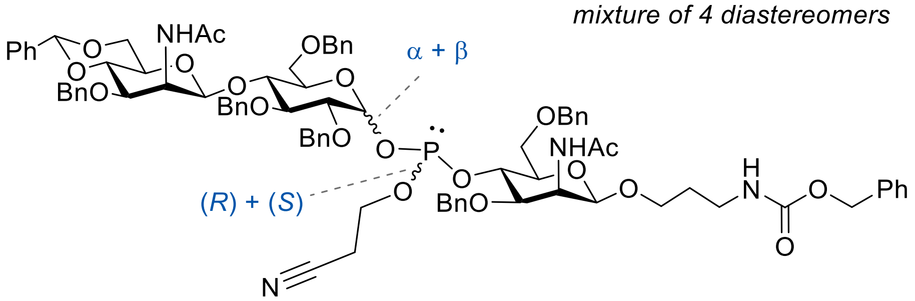

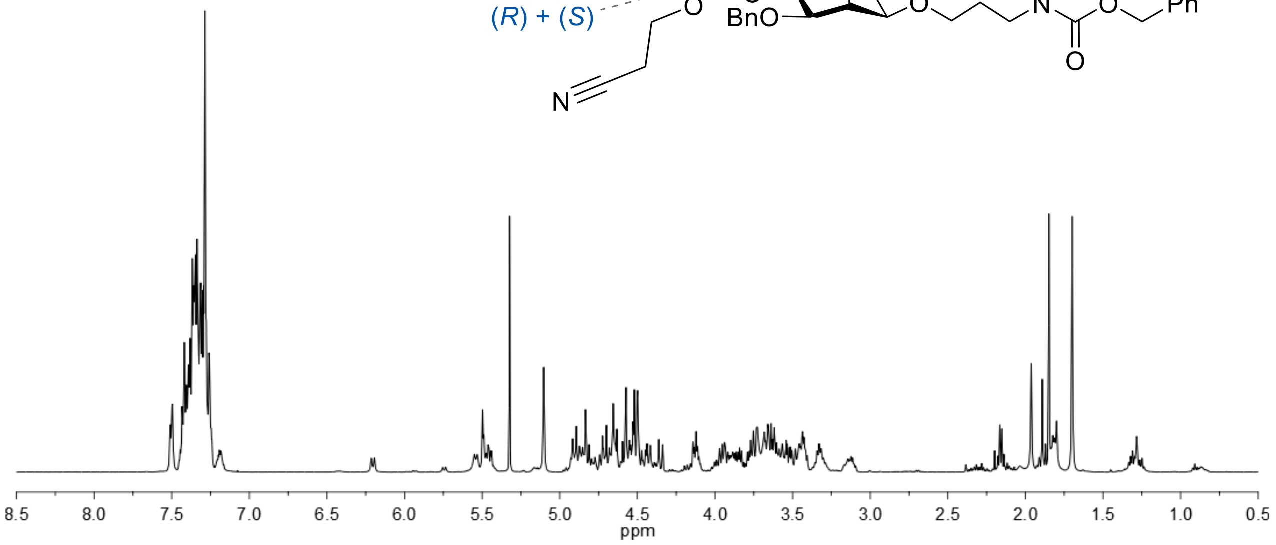

(25, crude): ^{13}C NMR (126 MHz, CDCl$_3$)

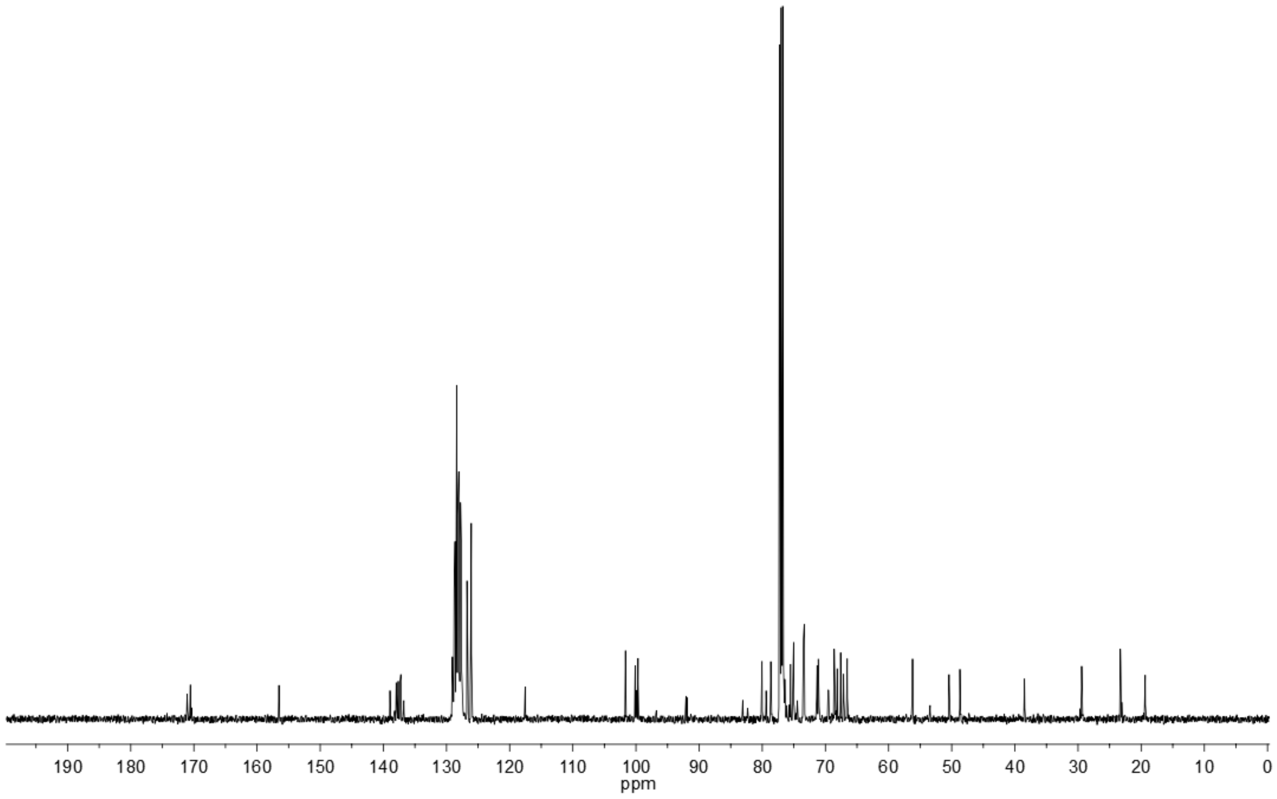

(25, crude): ^1H-^{13}C HSQC NMR ($CDCl_3$)

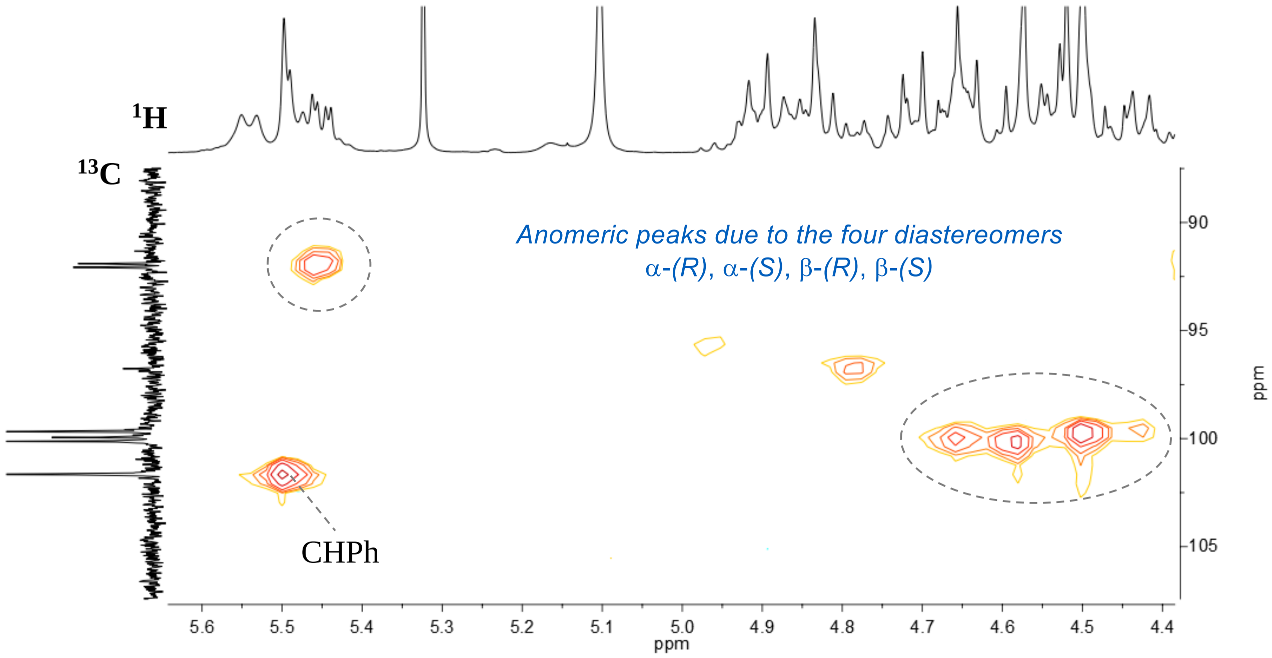

(25, crude): ^{31}P NMR (202 MHz, $CDCl_3$)

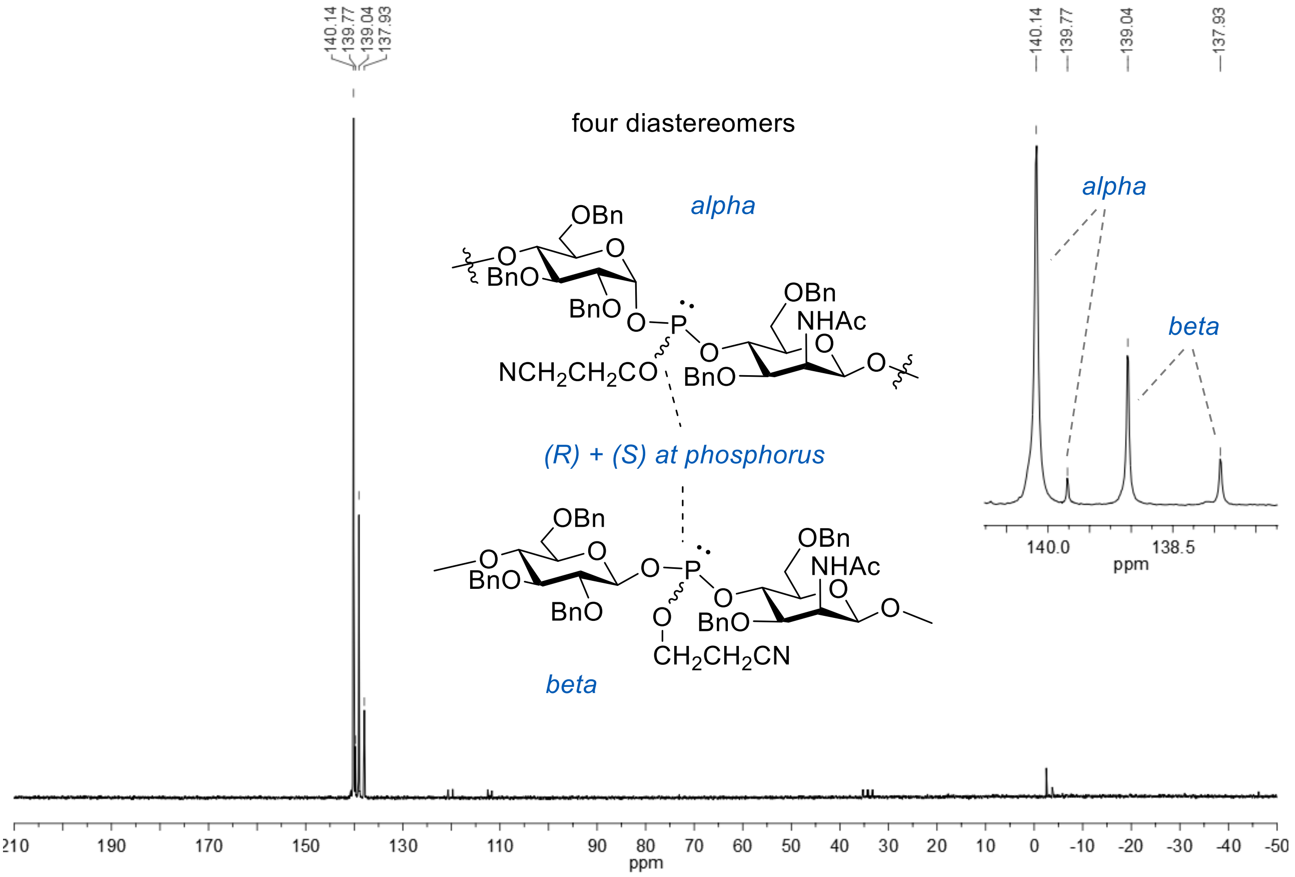

(25, crude): ^{1}H-^{31}P HMQC NMR ($CDCl_3$)

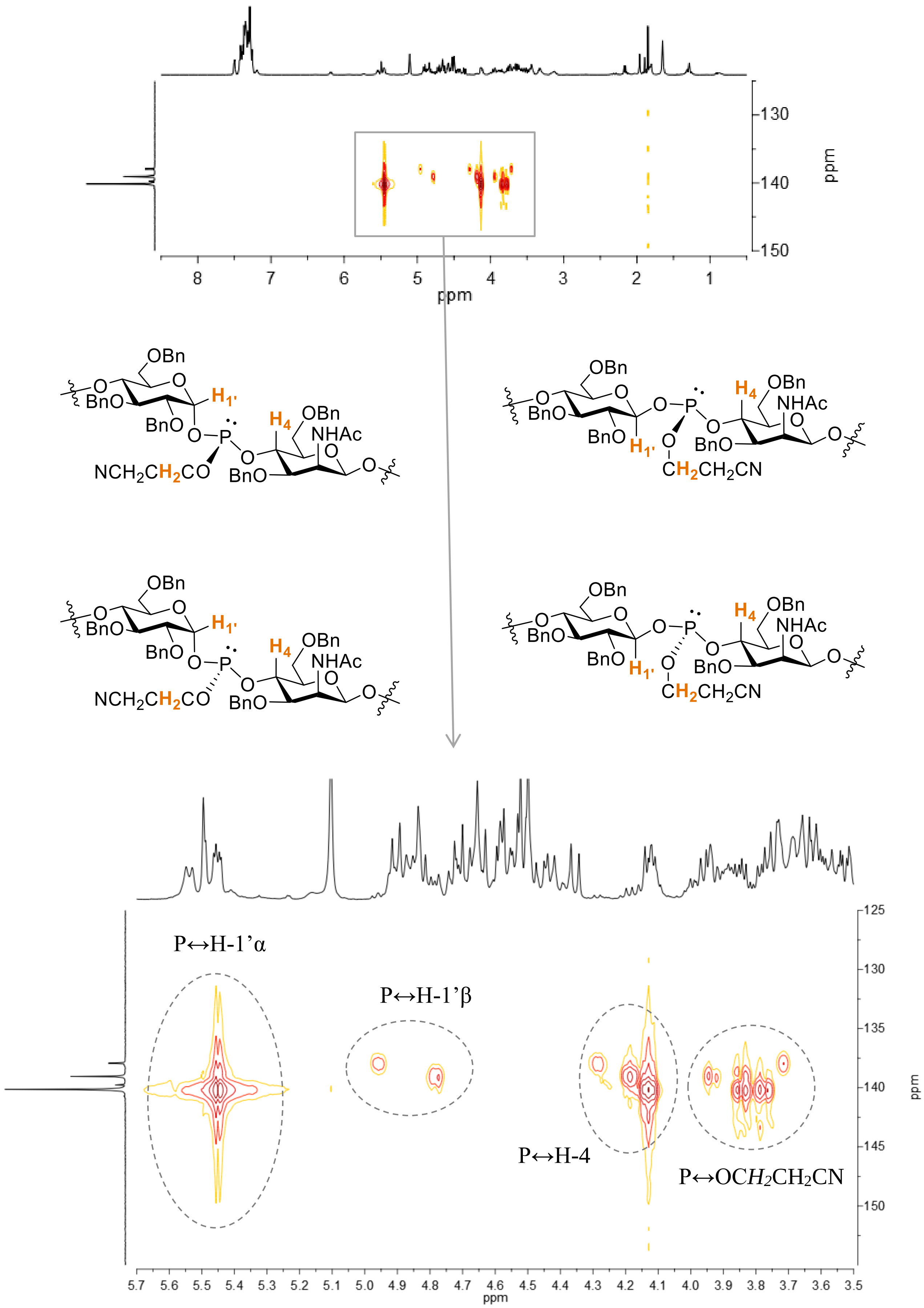

(**26α**): **^{1}H NMR (500 MHz, $CDCl_3$)**

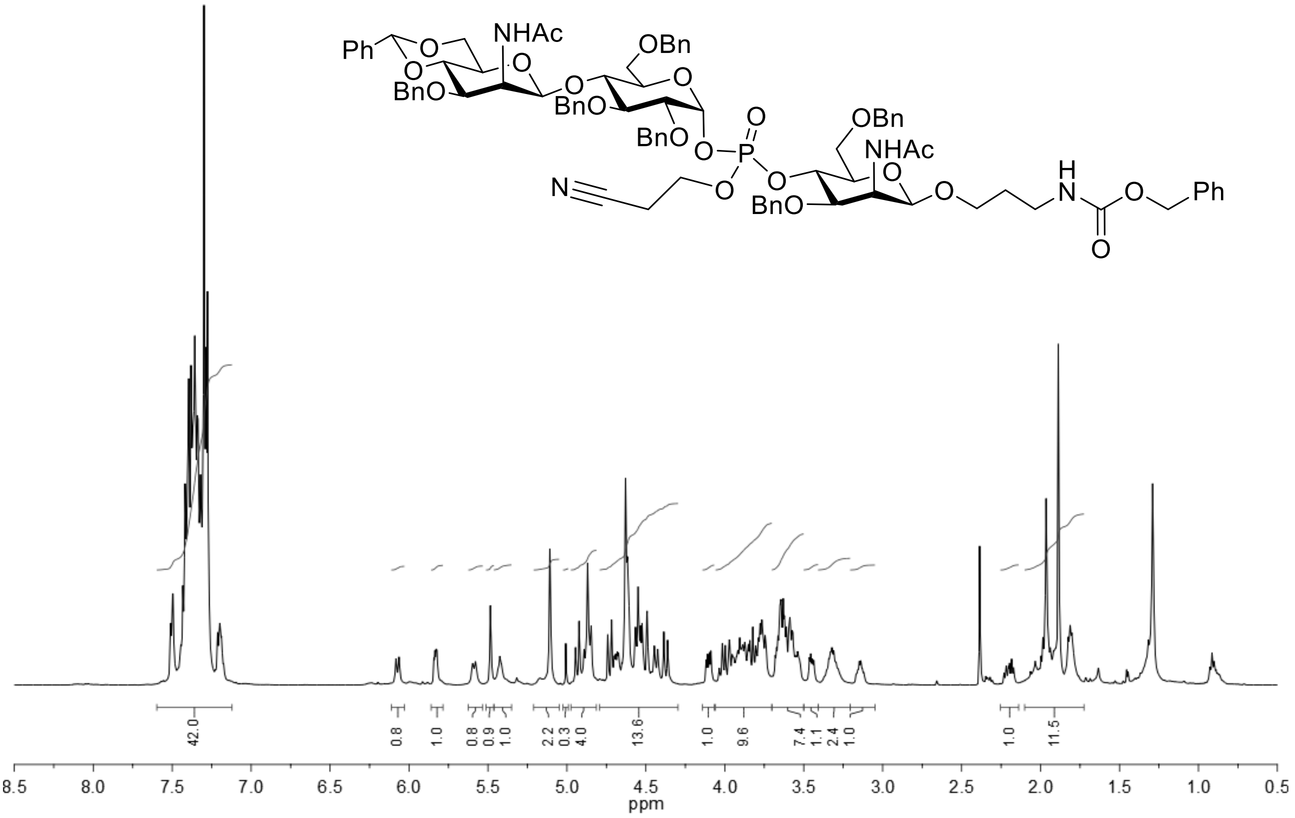

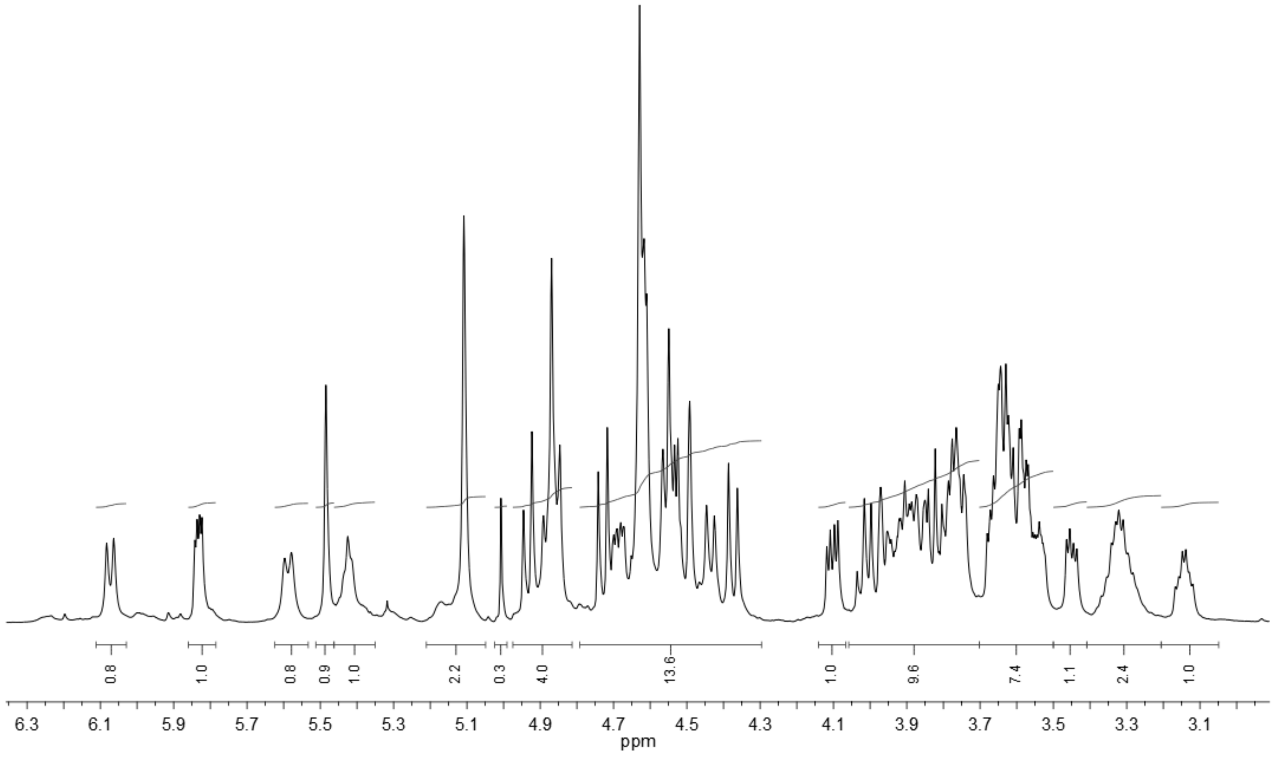

(26α): ^{31}P NMR (202 MHz, CDCl$_3$)

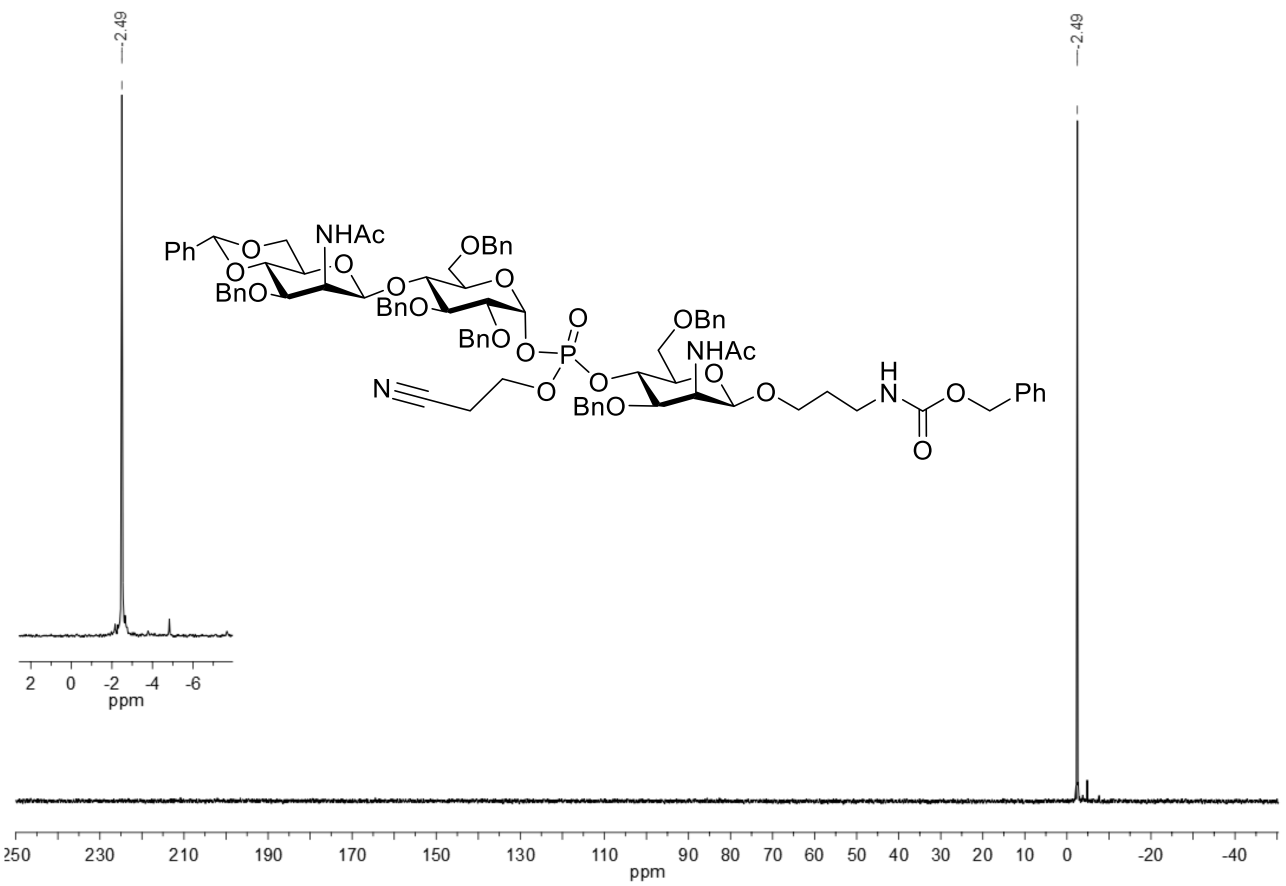

(26α): ^{1}H-^{31}P HMQC NMR (CDCl$_3$)

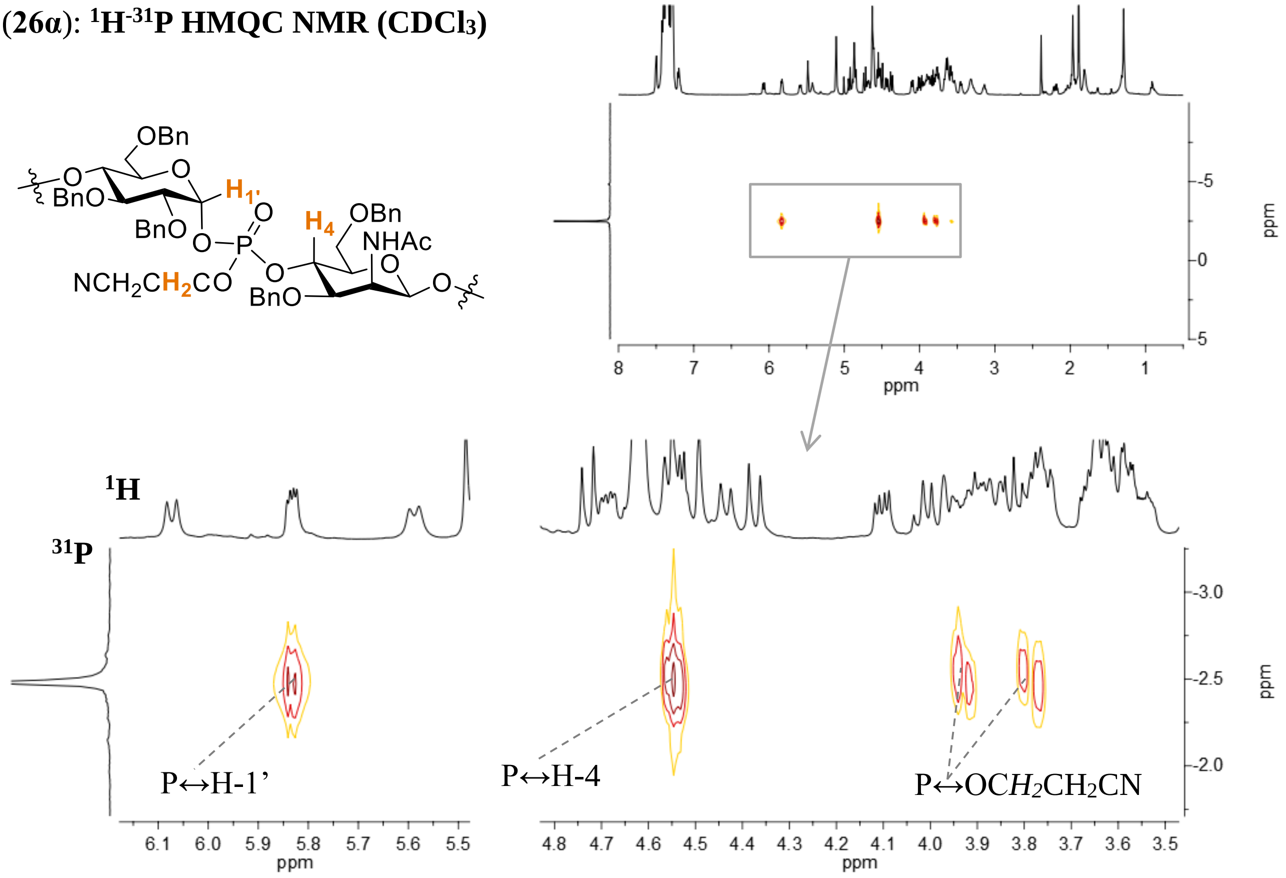

(26α): ^{13}C NMR (126 MHz, CDCl$_3$)

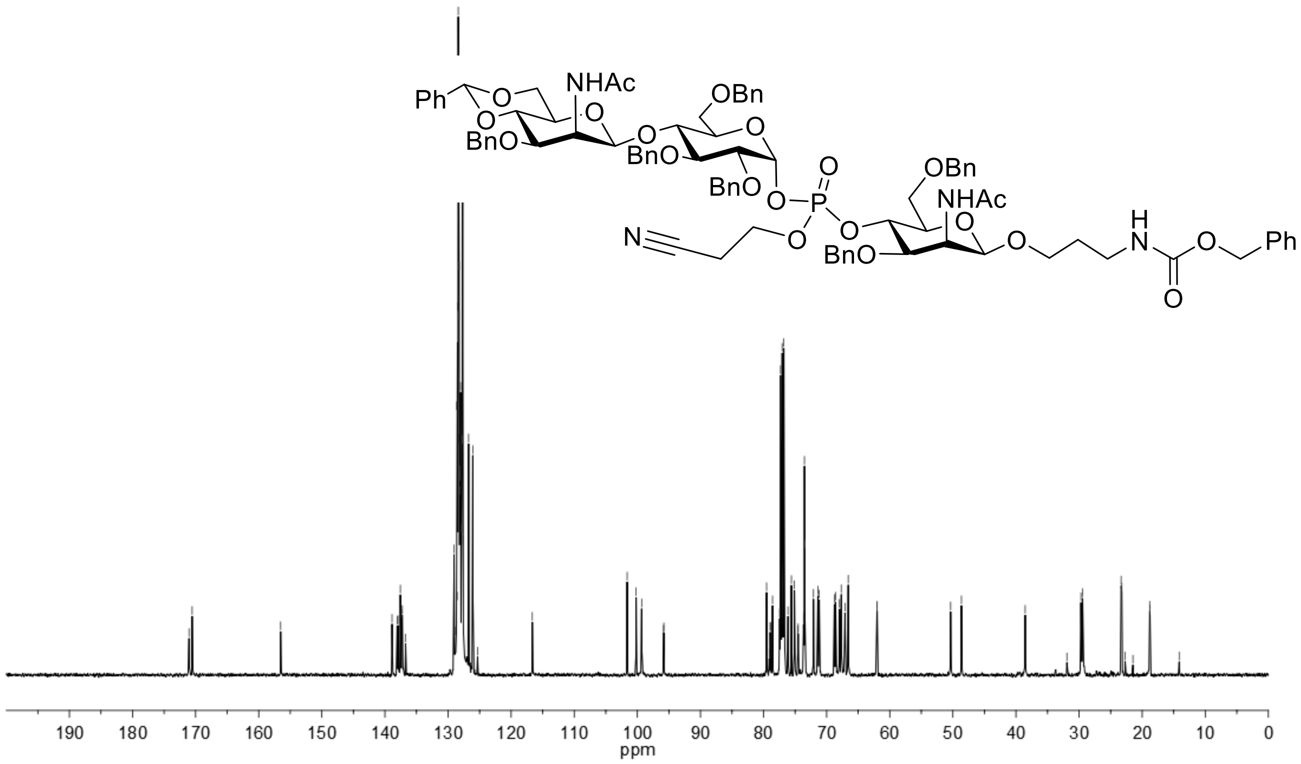

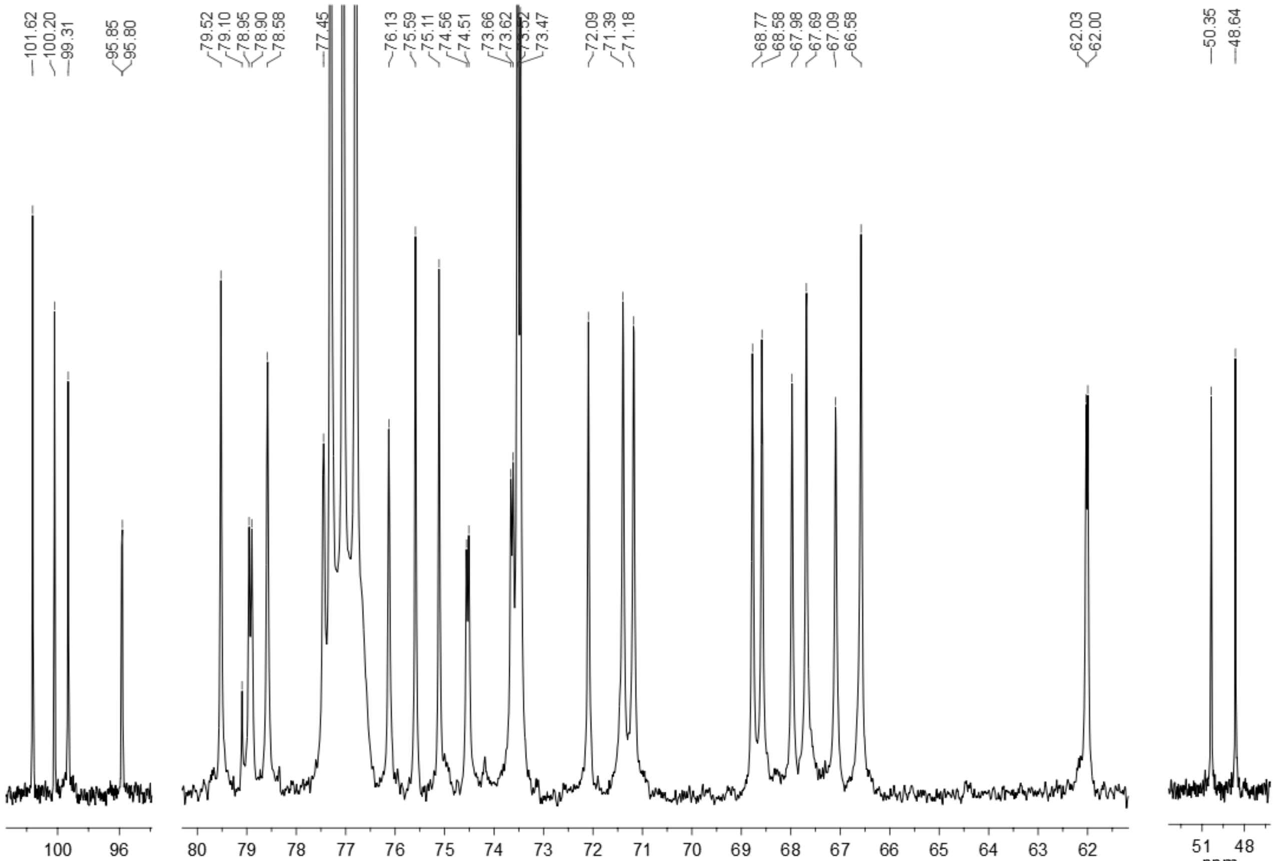

(27): ^{1}H NMR (500 MHz, CDCl$_3$)

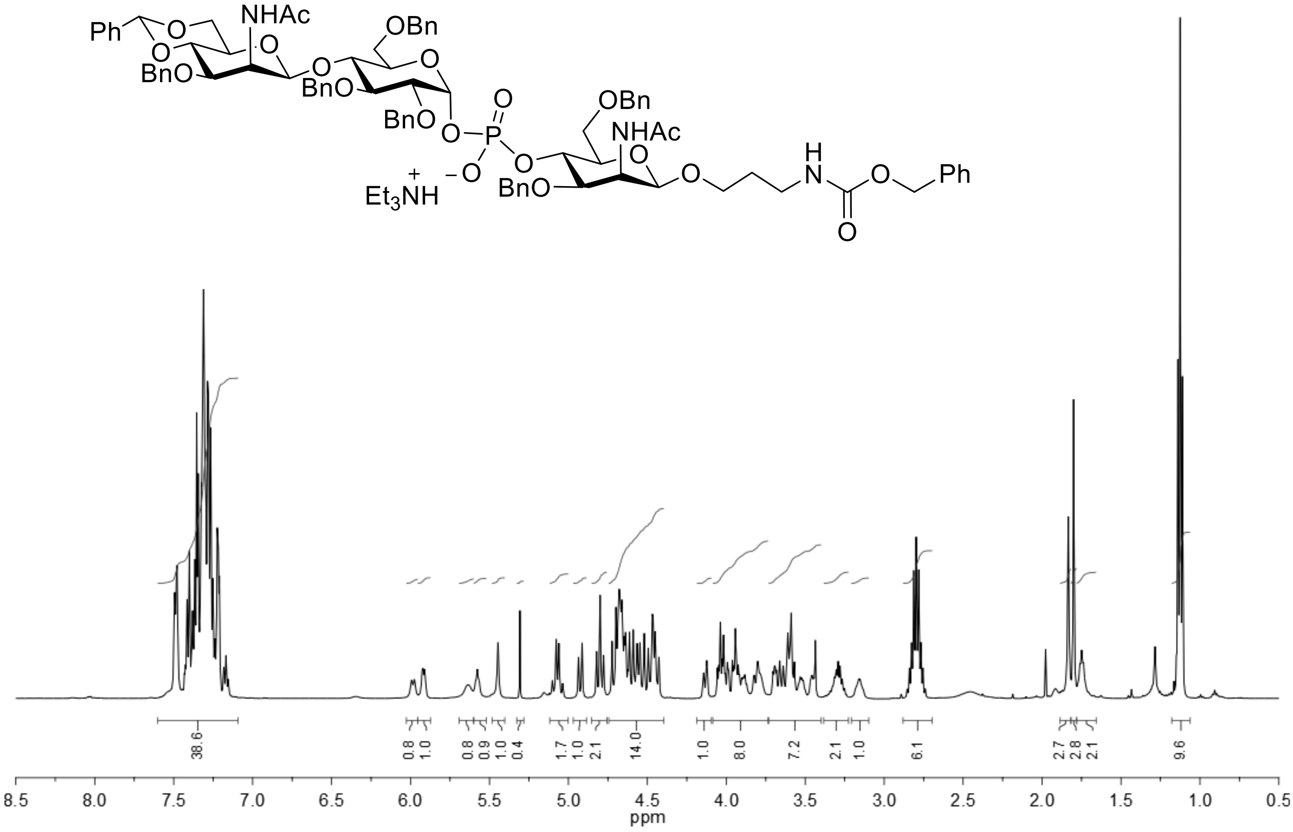

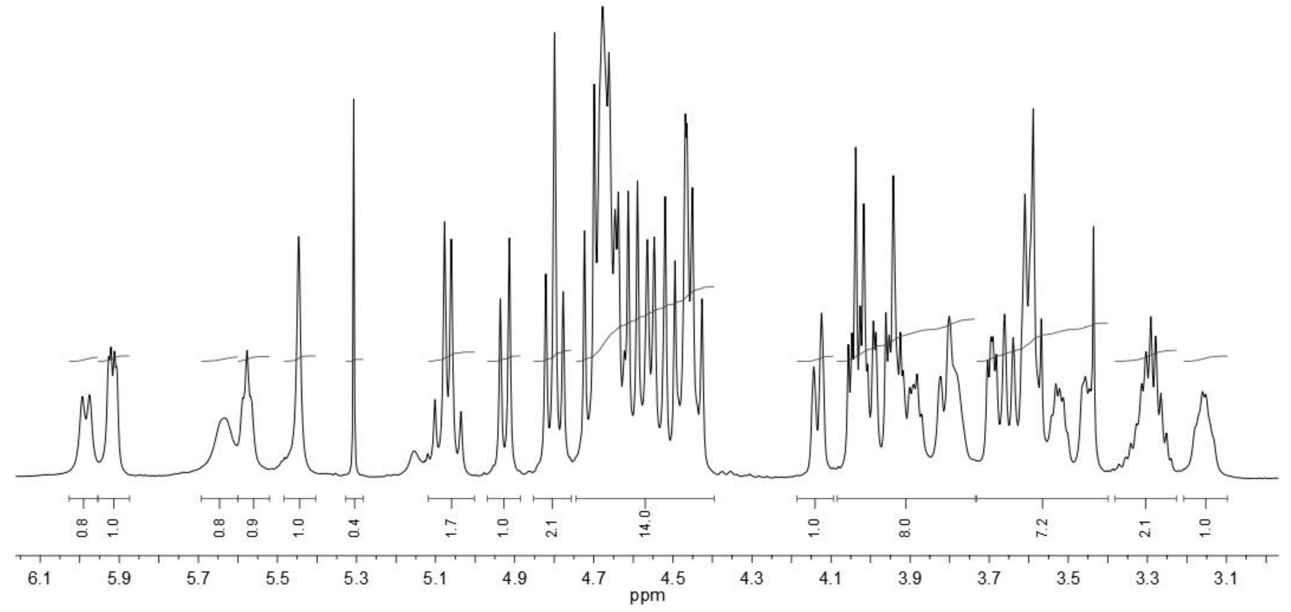

(27): ³¹P NMR (202 MHz, $CDCl_3$)

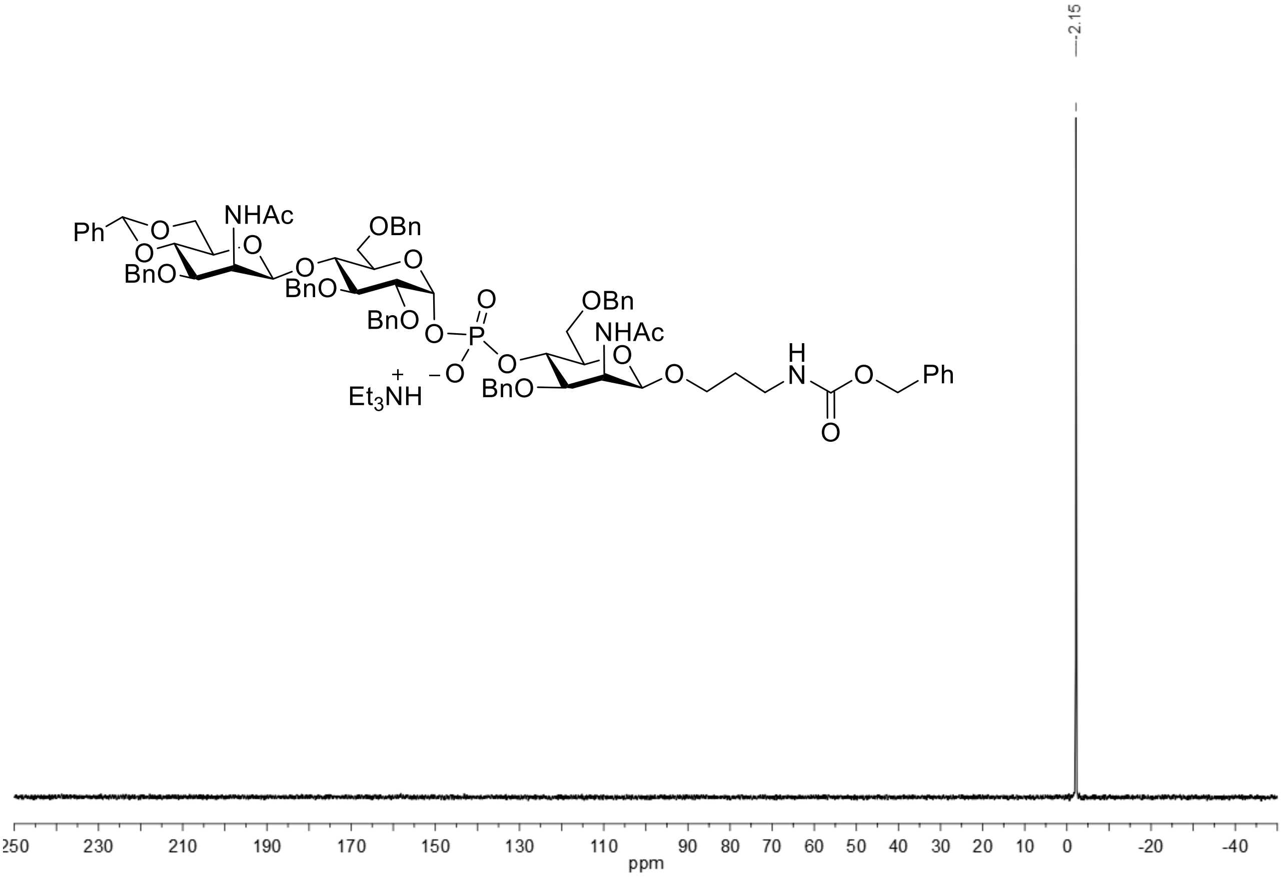

(27): ¹H-³¹P HMQC NMR ($CDCl_3$)

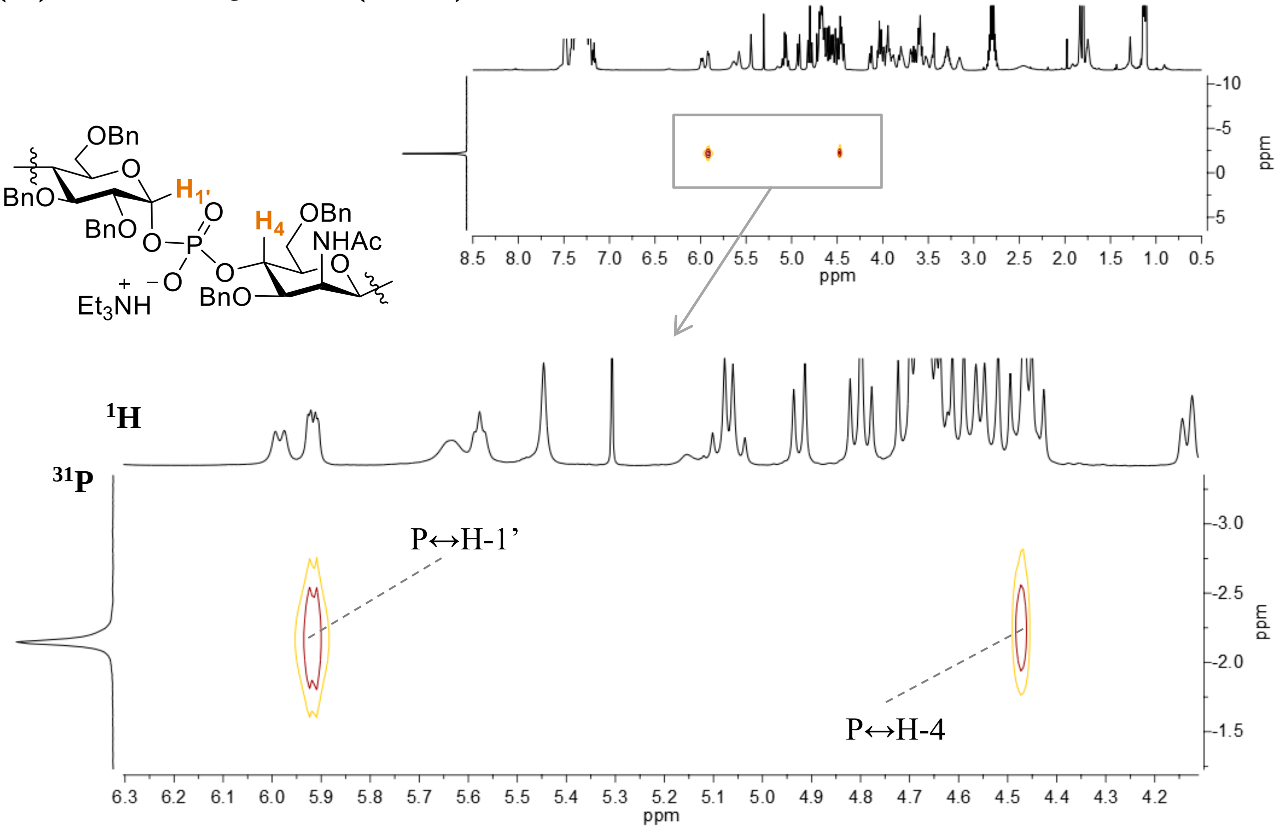

(27): ^{13}C NMR (126 MHz, CDCl$_3$)

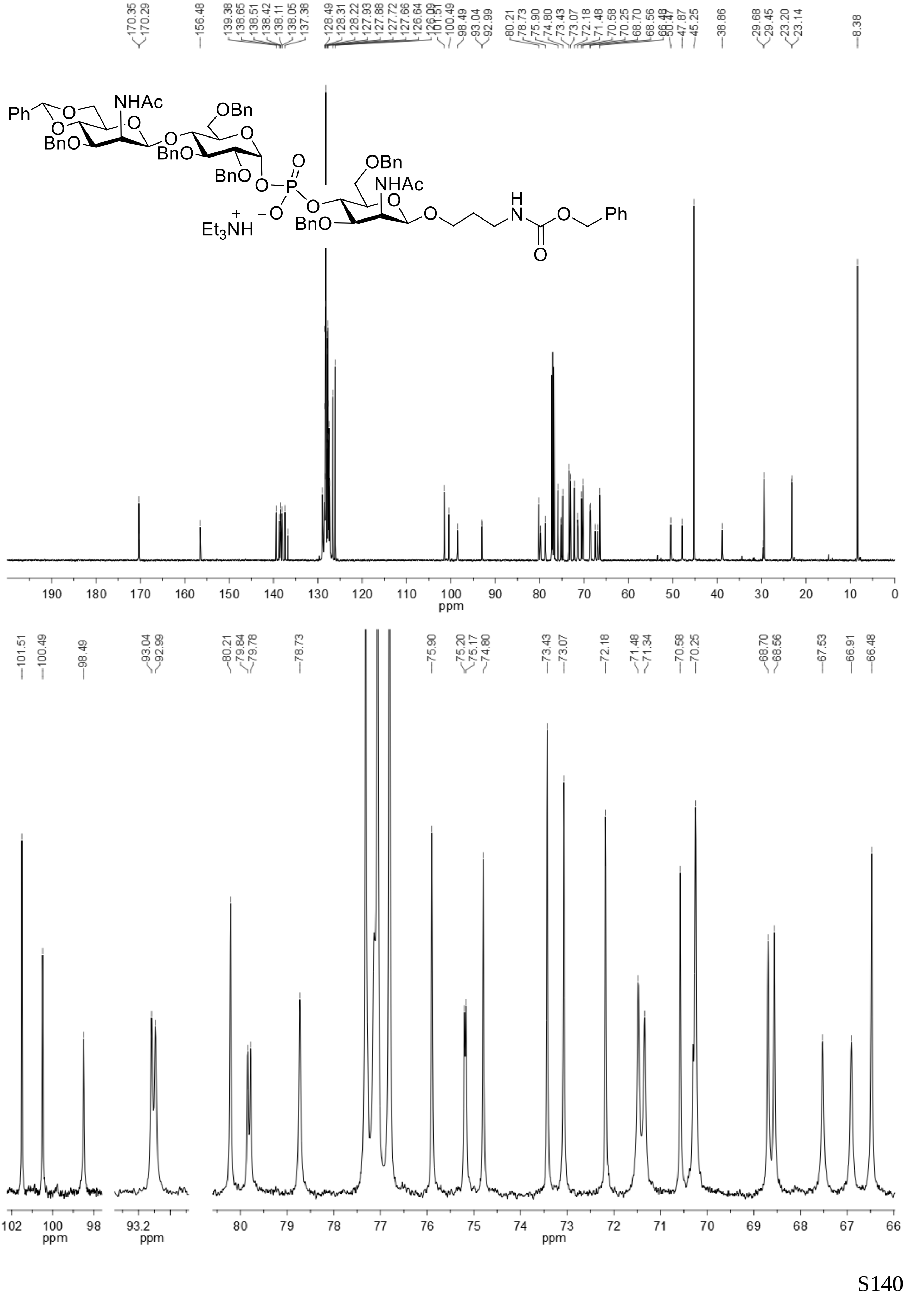

(5): ^{1}H NMR (500 MHz, D_2O)

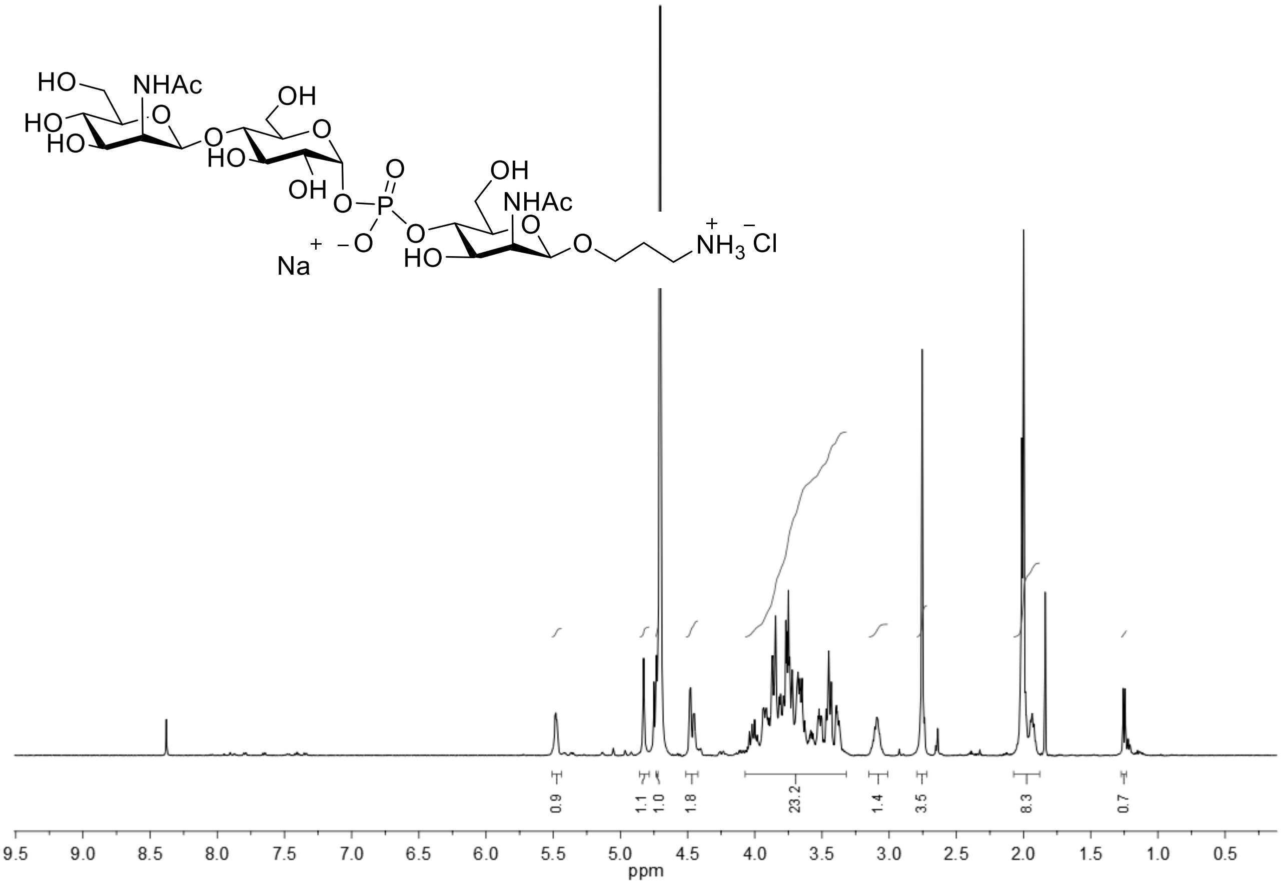

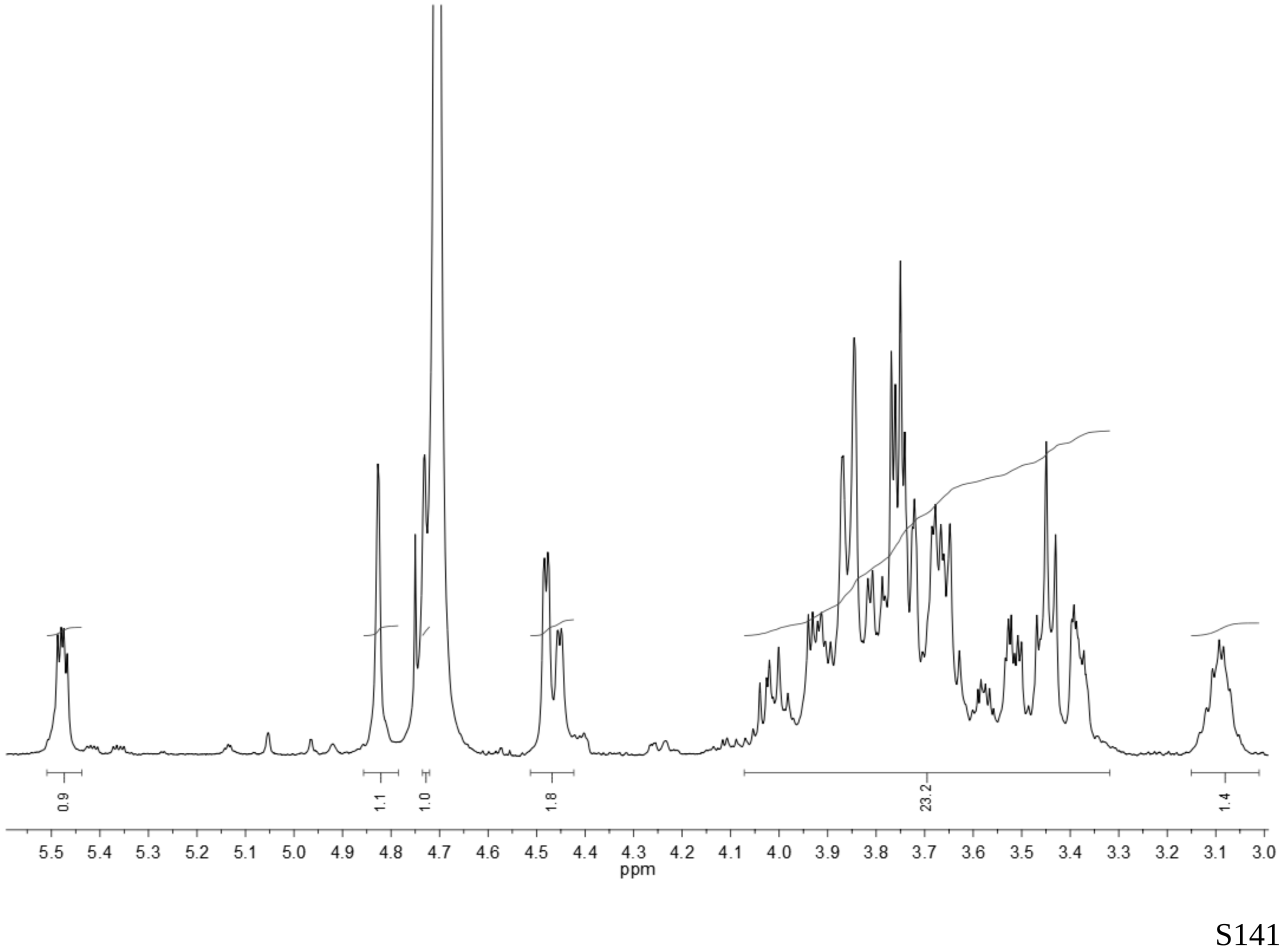

(5): ^{31}P NMR (202 MHz, D_2O)

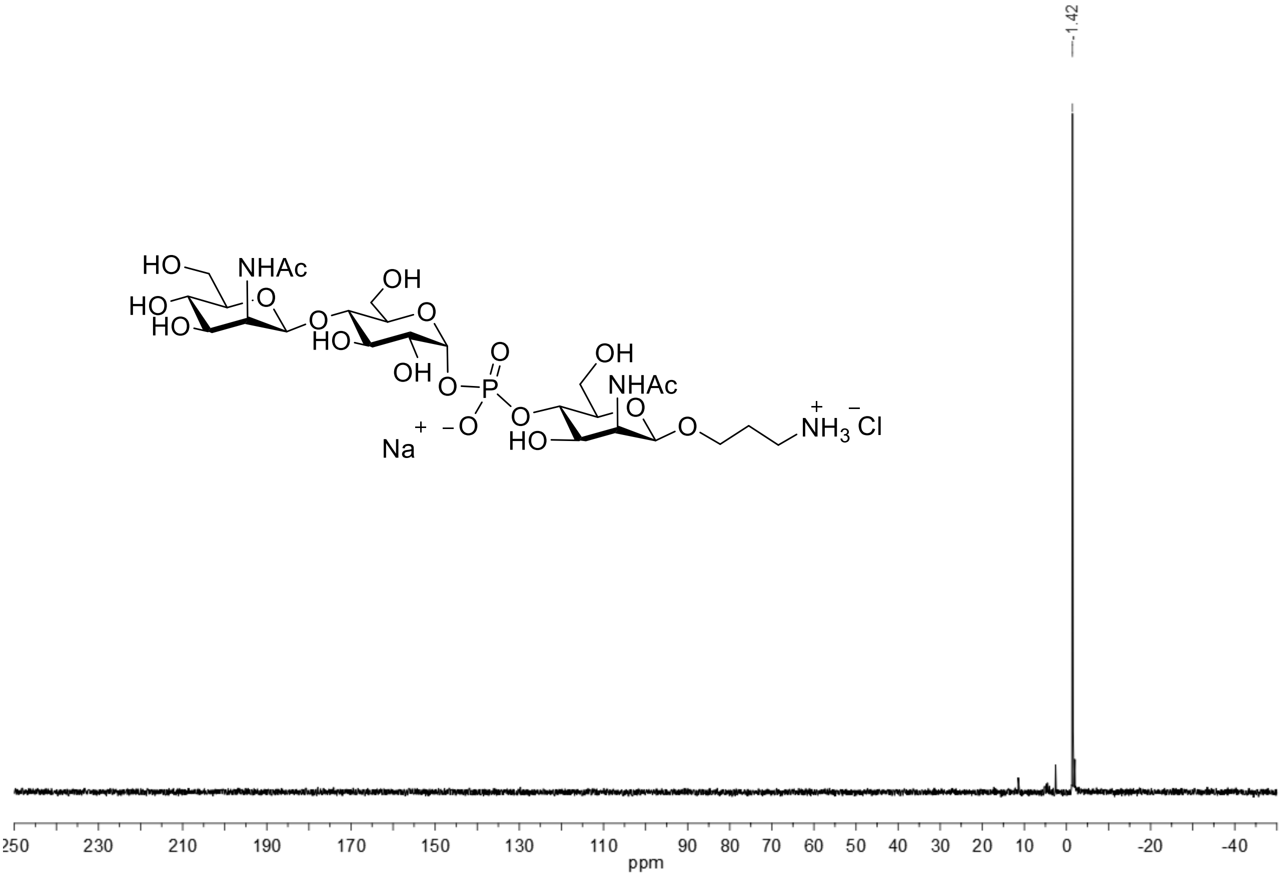

(5): ^{1}H-^{31}P HMQC NMR (D_2O)

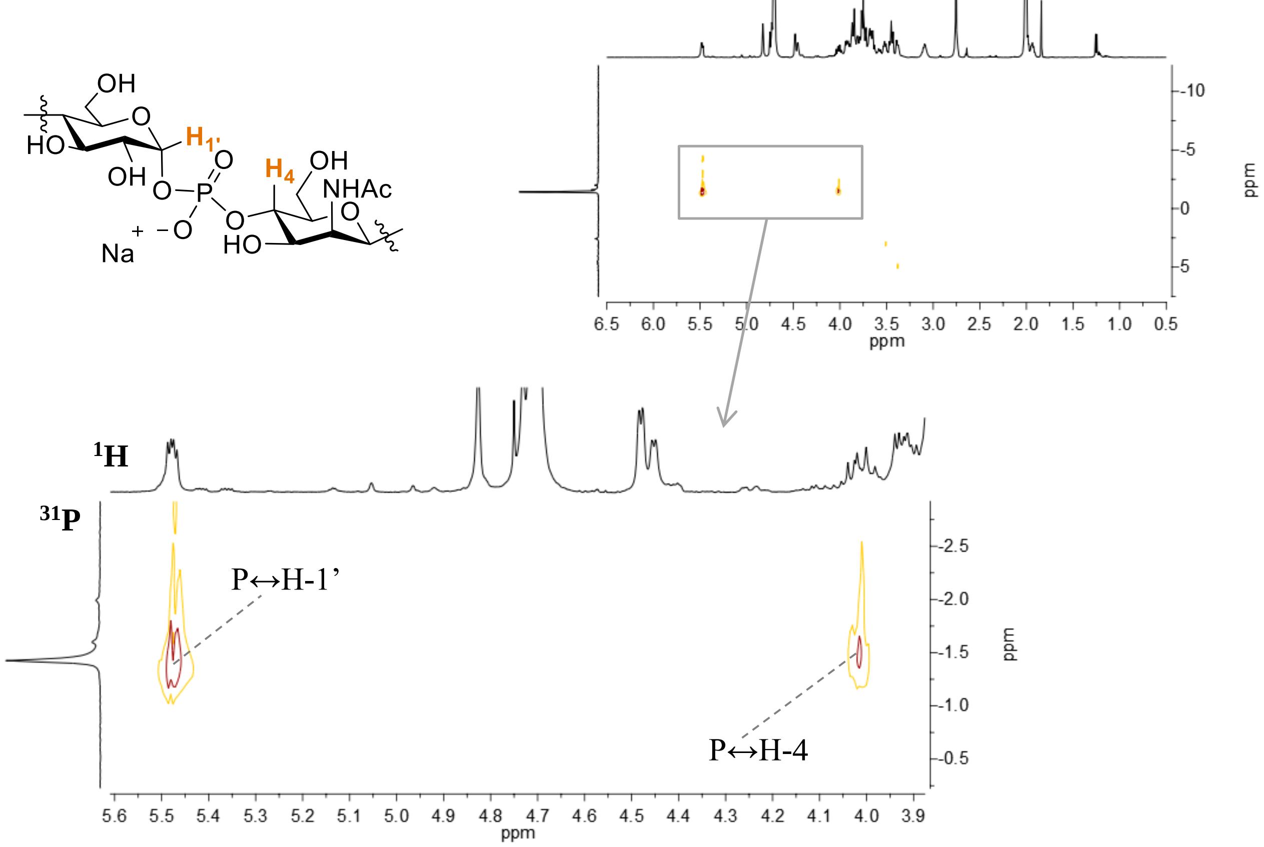

(5): ^{13}C NMR (126 MHz, D_2O)

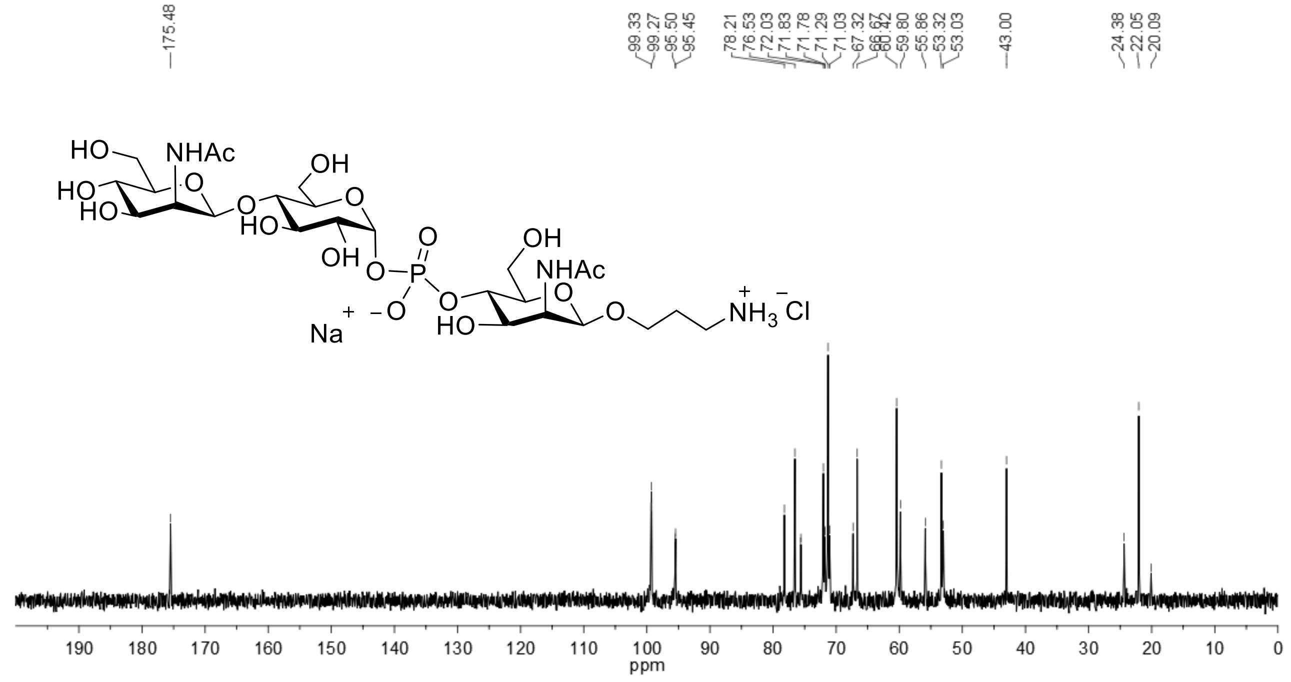

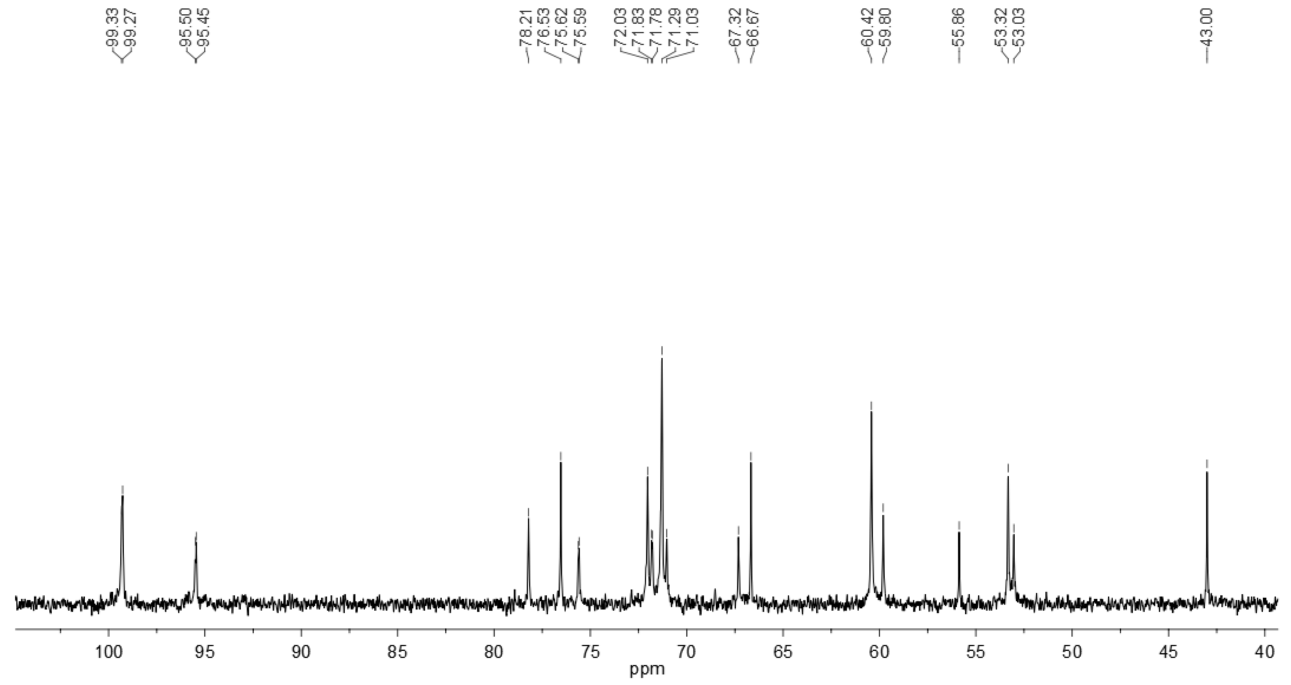

Supplement: Supplementary file 1 — cb1c00347_si_001.pdf [file cb1c00347_si_001.pdf]
